# Real-World Effectiveness of Beta-Blockers versus Other Antihypertensives in Reducing All-Cause Mortality and Cardiovascular Events

**DOI:** 10.1155/2022/6124559

**Published:** 2022-07-30

**Authors:** Caroline Foch, Arthur Allignol, Ulrike Hostalek, Emmanuelle Boutmy, Thilo Hohenberger

**Affiliations:** Merck KGaA, Frankfurter Str. 250, Darmstadt 64293, Germany

## Abstract

**Aim:**

The aim of the study was to compare the effectiveness of beta-blockers with other antihypertensive classes in reducing all-cause mortality, cardiovascular-related mortality and the risk of cerebrocardiovascular events.

**Methods:**

This noninterventional study was conducted within the UK Clinical Practice Research Datalink. Hypertensive patients who initiated antihypertensive monotherapy were allocated to one of five cohorts: beta-blockers; angiotensin-converting enzyme inhibitors (ACEi); angiotensin II receptor blockers (ARB); calcium channel blockers (CCB); and diuretics. Differences in outcomes were assessed using Cox proportional hazard models with competing risks.

**Results:**

A total of 44,404 patients were prescribed beta-blockers (75% atenolol), 132,545 ACEi, 12,018 ARB, 91,731 CCB, and 106,547 diuretics. At baseline, patients in the beta-blocker cohort presented more frequently with angina, arrhythmia, and atrial fibrillation. The risk of all-cause mortality was lower for those treated with ACEi, ARB, and CCB, and no difference was observed compared with diuretics (adjusted hazard ratio versus beta-blockers (98.75% CI), for ACEi 0.71 (0.61, 0.83), ARB 0.67 (0.51, 0.88), CCB 0.76 (0.66, 0.88), diuretics 1.06 (0.93, 1.22)). No differences were seen in the risk of cardiovascular mortality for patients treated with beta-blockers, ARB, CCB, and diuretics, while a lower risk in patients treated with ACEi was observed (ACEi 0.63 (0.43, 0.91), ARB 0.64 (0.32, 1.28), CCB 0.71 (0.49, 1.03), diuretics 0.97 (0.69, 1.37)).

**Conclusions:**

These data add to the limited pool of evidence from real-world studies exploring the effectiveness of beta-blockers versus other antihypertensive classes. Discrepancies to previously published studies might be partly explained by differences in the selected populations and in the follow-up time.

## 1. Introduction

Hypertension is a well-established risk factor for cardiovascular diseases such as myocardial infarction and stroke [1], and affects approximately 13% of people in the UK [2].

It is well known that reducing blood pressure (BP) with the use of antihypertensive drugs lowers the risk of cardiovascular disease and mortality [3]. Current options for antihypertensive therapy include the following five drug classes: beta-blockers; angiotensin II receptor blockers (ARB); angiotensin-converting enzyme inhibitors (ACEi); calcium channel blockers (CCB); and diuretics, including thiazides and thiazide-like diuretics [4].

Beta-blockers are recommended as a preferred treatment option for patients with hypertension and postmyocardial infarction, angina, heart failure, or for those requiring heart rate control by the European Society of Cardiology (ESC) and the European Society of Hypertension (ESH) [4]. However, some national guidelines, such as those from the National Institute for Health and Care Excellence (NICE) [5], do not recommend beta-blockers as a first-line therapy for hypertension.

Current guidelines rely on the available evidence from randomized controlled trials (RCTs), but they contain contrasting results regarding the efficacy of beta-blockers to treat hypertension. In a meta-analysis of RCTs including patients with hypertension, BP-lowering by all classes of antihypertensive drugs was accompanied by significant reductions in stroke and major cardiovascular events (composite of coronary heart disease, risk of heart failure, cardiovascular death, and all-cause death). This evidence suggests that the reduction of these events is due to BP-lowering rather than specific drug properties [6]. However, a Cochrane systematic review, which included 91,561 patients with hypertension, concluded that beta-blockers were inferior to other antihypertensive drug classes in preventing cardiovascular disease (fatal and nonfatal coronary heart disease, stroke, congestive heart failure, and transient ischemic attacks) and mortality outcomes [7]. It is worth noting, however, that these RCTs allowed the prescription of one or more additional drugs to achieve the BP target. Therefore, comparisons are often made between various combined treatments rather than two different drug classes.

Compared with clinical trials, which typically consider short treatment durations, using real-world data enables efficacy and safety outcomes to be monitored over a longer treatment period. This information is potentially more meaningful to healthcare practitioners as it is more reflective of clinical practice. Despite this, there are limited real-world data assessing the effectiveness of beta-blocker monotherapy versus each single antihypertensive class in routine clinical practice. For instance, although Bronsert et al. [8] observed that beta-blockers offer comparable BP reductions to other antihypertensive drug classes, the study did not compare the effectiveness of the different classes in reducing the risk of mortality or cerebrocardiovascular-related outcomes.

The primary aim of this noninterventional study was to compare the risk of all-cause mortality between patients with hypertension who initiated beta-blocker monotherapy versus other antihypertensive classes, including ACEi, ARB, CCB, and diuretics.

The secondary aims were to compare the risks of cerebrocardiovascular-related mortality, cardiovascular events (myocardial infarction), and cerebrovascular (stroke, ischemic, and hemorrhagic stroke) events between beta-blockers and the individual cohorts of monotherapy.

## 2. Material and Methods

This noninterventional study was conducted using routinely collected patient data from the UK Clinical Practice Research Datalink (CPRD) GOLD database [9] with a linkage to the Office for National Statistics (ONS) death registration data to collect the cause of death.

### 2.1. Data Source

CPRD is a large primary care database that collects anonymized electronic patient medical records from participating general practitioners (GPs), covering over 50 million patients in the UK [9]. The patients included in the CPRD database are considered representative of the general population, with similar distribution in terms of age, sex, and ethnicity [10]. GPs act as the first point of contact for any nonemergency health-related issues, such as hypertension, which may then be managed within primary care. Furthermore, it is only possible to be registered to one GP at a time. Therefore, patients can be followed over time in CPRD.

The ONS death registration data contain the dates and coded causes of death for the population of England and Wales from January 1998, based on the death certificate, and are considered the gold standard. Study approval was reviewed and granted by the CPRD Independent Scientific Advisory Committee (ISAC) of the Medicines and Healthcare products Regulatory Agency (MHRA) (protocol number 18_106R).

### 2.2. Study Population

The study population included all patients (≥18 years) in the CPRD database who were not prescribed an antihypertensive drug in the year prior to the index date and met the following criteria: had initiated an oral monotherapy with an antihypertensive drug between January 1, 2000, and December 31, 2015 (the first prescription being defined as the index date); had a diagnosis for hypertension at any time prior to the index date; had at least one year of medical history in their CPRD record prior to their index date. Patients' data were also required to meet the quality standards in the CPRD; patients had to be registered in a practice up to research standards, where the practice data are deemed to be of research quality, and the patients had to be flagged as acceptable (based on registration status, recording of events in the patient record as well as valid age and sex).

To avoid the inclusion of patients that initiated a combination treatment rather than a monotherapy at index, patients were requested to have no record of an antihypertensive drug prescription other than the index hypertensive class during the 14 days following the index date. For patients that fulfilled the inclusion and exclusion criteria several times over the inclusion period, only the first episode of an event was used.

### 2.3. Design

**Figure 1:**
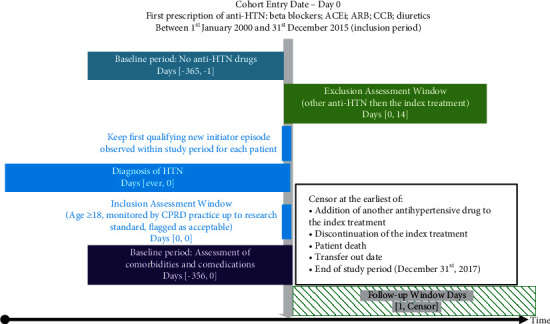
Study design.

The index date was defined as the date of initiation of one of the index treatments of interest. Patients were followed from the index date plus 1 day until the first occurrence of any one of the following events: change of therapy (discontinuation of the index treatment; addition of another antihypertensive drug to the index treatment); patient death; transfer-out date; or the end of the study period on 31 December 2017) (Figure 1).

Figure 1 is based on the framework for graphical depiction of longitudinal study designs in healthcare databases, originally published by Schneeweiss et al., 2019, under CC BY license [11].

ACEi, angiotensin-converting enzyme; ARB, angiotensin II receptor blocker; CCB, calcium channel blocker; CPRD, Clinical Practice Research Datalink; HTN, hypertension.

### 2.4. Exposure

The study population was categorized into one of five treatment cohorts depending on the antihypertensive class prescribed at the treatment initiation (index treatment): beta-blockers; ACEi; ARB; CCB; or diuretics. See Supplementary Table 1. Antihypertensive drugs were considered for each treatment of interest.

The duration of prescription was imputed based on a modified version of the method used in the Observational Medical Outcomes Partnership (OMOP) Common Data Model CPRD Mapping Specification [12]. If completed, the duration of the prescription corresponded to the “number of days of supply.” If this was not completed, the duration of the prescription was imputed with the most common value based on product code, number of tablets in a pack, quantity, and daily dose. If no value could be imputed, the value was fixed to 0.

To capture discontinuation or switch to a combination therapy, the following algorithm was implemented:

(1). The follow-up period of each patient was divided into 14-day segments.
(2). The presence or absence of an antihypertensive treatment in each segment was assessed. A treatment needed to be present for at least 7 days to be designated as “present” for that segment.
(3). Treatment discontinuation was defined as a gap of ≥90 days after the last 14-day segment where the treatment was present. A switch to an antihypertensive treatment within the same class was not classified as discontinuation.
(4). Combination therapy was defined as an exposure overlap of at least 7 days within a 14-day segment of two or more antihypertensive drugs of the same or different antihypertensive class.

### 2.5. Outcomes

The primary outcome of this study was all-cause death within the follow-up period. Death was defined via the CPRD algorithm that derived the date of death from the earliest event among the following three types of records: patient transfer-out date specified as “death”; information entered by the GP in the death administration structured data area using the earliest date of the death recorded by either the CPRD or ONS, and date of record of the information; and a READ code recorded indicating a death (statement of death, suicide). Exact agreement on the death date between CPRD and the ONS was 69.7% between 1998 and 2013, increasing from 53.4% in 1998 to 78.0% in 2013 [13]. Overall, most dates of death aligned within ±30 days, increasing from 80% to 98.8% between 1998 and 2013. Consequently, for censoring follow‐up and calculating mortality rates, CPRD data are likely to be sufficient as a delay in death recording of up to 1 month is unlikely to impact results significantly or to differ systematically between the cohorts [13].

Secondary outcomes included cerebrocardiovascular-related mortality as recorded in the ONS, defined as death due to ischemic heart diseases (international classification of diseases (ICD)-10: I20–I25; ICD-9: 410–414), cerebrovascular diseases (ICD-10: I60–I69; ICD-9: 430–438), and heart failure (ICD-10: I50; ICD-9: 428). For this outcome, the analysis was restricted to a sub-cohort of patients, registered in practices in England, who consented to the ONS linkage. Other secondary outcomes were myocardial infarction, and hemorrhagic and ischemic stroke defined by at least one diagnosis code recorded by the GP in CPRD within the follow-up periods.

### 2.6. Potential Confounders and Effect Modifiers

To consider potential imbalances between the cohorts, the models were adjusted on age at index year; sex; time from hypertension diagnosis; smoking status; body mass index (BMI); diastolic BP; systolic BP; angina; stroke; arrhythmia; chronic heart failure; myocardial infarction; peripheral vascular diseases; diabetes mellitus; dyslipidemia; and renal impairment. To account for missing data at baseline on biologic values such as blood pressure or body mass index, multiple imputation by chained equations (MICE) was performed. Smoking status was defined based on the algorithm developed by Booth et al. [14]. Clinical measurements such as BMI and BP were defined as the last recorded value within the year prior to the index date. Comorbidities and co-medications were defined by at least one recorded code for a disease (READ) or product code, respectively, recorded within the year prior to the index date. For diabetes mellitus and dyslipidemia, in addition to a READ code, a prescription of antidiabetic medication or a statin/fibrate, respectively, was considered as presence of disease. All code lists for exposure, covariates, and outcomes are available in Supplementary Tables 5–31. See Supplementary Tables 5–31. Code lists include: hypertension; beta-blockers; ACEi; ARB; diuretics; CCB; myocardial infarction; stroke; platelet aggregation inhibitors; anticoagulants; anti-inflammatory non-steroids; angina; anti-anginal; atrial fibrillation; arrhythmia; asthma; obstructive pulmonary disease; diabetes–diagnosis; diabetes–drugs; chronic heart failure; dyslipidemia–diagnosis; dyslipidemia–drugs; erectile dysfunction; renal; smoking–READ; smoking–drugs; peripheral vascular disease, respectively.

### 2.7. Data Analysis

Sample selection and variable creations were performed within the Instant Health Data (IHD) platform (Boston Health Analytics, Boston, MA); further analysis was performed using *R* version 3.5.1.

For the primary outcome, differences in all-cause mortality between cohorts were assessed using adjusted Cox proportional hazard models.

For secondary outcomes, differences were assessed using adjusted Cox proportional hazard models and Fine and Gray proportional sub-distribution hazard models, considering discontinuation as a competing event. A competing event is any event that prevents the observation of the event of interest. In the presence of competing events, the probability of experiencing the event of interest depends on the cause-specific hazard of the event of interest and the cause-specific hazard of the competing event [15].

Treatment effects can be assessed in terms of cause-specific hazard ratios (HRs), i.e., an increase (HR > 1) or decrease (HR < 1) in the instantaneous risk of experiencing the event of interest [15].

As the probability of experiencing the event of interest also depends on the competing event, an HR alone representing the event of interest is not enough to assess a treatment effect on probability [15]. Therefore, the sub-distribution hazard ratio (SHR) using the Fine and Gray model reflects the probability of an event, to be increased or decreased considering that other events might occur, and has a direct interpretation of the cumulative incidence function. For example, an SHR >1 leads to an increase in the probability of experiencing the event of interest over the entire follow-up period [15].

To account for the multiple comparisons, which included four comparisons, the estimates were given with associated 98.75% confidence intervals (CIs) due to a Bonferroni correction (1–(0.05/4)).

#### 2.7.1. Sensitivity Analysis

In a sensitivity analysis, a propensity score (PS) method with inverse probability of treatment weighting (IPTW) was applied to the primary and secondary outcomes. The PS was estimated using boosted regression trees. Stabilized weights for each subject were included in the outcome models. Covariate balance achieved by the PS was checked using standardized mean differences, and the distribution of propensity score for the cohorts was graphically assessed to identify the extent of overlap in PS. The results of this sensitivity analysis are available in Supplementary Tables 2–4 and are highlighted in Section 3 when discordant. See Supplementary Tables 2–4 for sensitivity analysis results for all-cause death and cardiovascular mortality with IPTW and the Fine and Gray model for estimating the incidence of cardiovascular mortality, myocardial infarction, and cerebrovascular outcome, respectively.

## 3. Results

### 3.1. Patients

A total of 44,404 patients were included in the beta-blocker cohort, 132,545 in the ACEi, 12,018 in the ARB, 91,731 in the CCB, and 106,547 in diuretic cohorts. See Supplementary Figure 1—patient attrition. Due to the absence of a diagnosis for hypertension, a higher number of patients were excluded from the beta-blocker cohort compared with the other cohorts.

Patients in the beta-blocker cohort were prescribed atenolol (75%), bisoprolol (11%), propranolol (8%), or an alternative (6%). The proportion of patients aged over 55 years was higher in the CCB (80.9%) and diuretic (79.2%) cohorts compared with the beta-blocker (58.6%), ARB (58.1%), and ACEi (48.9%) cohorts (Table 1). Additionally, a higher proportion of male patients was observed in the ACEi (58.1%) and ARB (56.8%) cohorts compared with the beta-blocker, CCB, and diuretics cohorts (50.1%, 51.9%, and 39.8%, respectively). At index, at least half of the patients were newly diagnosed with hypertension less than 1 month prior. Consequently, most patients (88.4–94.8%) presented with high systolic BP (≥140 mmHg) at baseline.

At baseline, patients in the beta-blocker cohort presented more frequently with angina, arrhythmia, and atrial fibrillation (beta-blocker versus all other cohorts: angina diagnosis 2.8% versus 0.3–0.6%; anti-anginal medication 6.6% versus 0.8–2.2%; arrhythmia 2.8% versus 0.6–0.8%; atrial fibrillation 2.4% versus 0.5–0.9%), but less frequently with diabetes mellitus and asthma compared with the ACEi and ARB cohorts (3.4% versus 11.0–15.9%, and 1.0% versus 6.4%, respectively (Table 1)). Follow-up durations varied across the cohorts, with the diuretic and beta-blocker cohorts having shorter median follow-up (3.6 months and 4.8 months, respectively) compared with ACEi, ARB, and CCB cohorts (7.2–13.2 months; Table 1).

### 3.2. Risk of All-Cause and Cardiovascular Mortality

**Figure 2:**
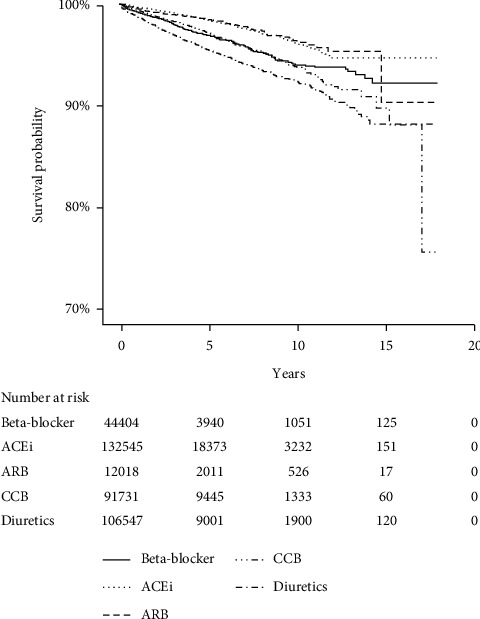
All-cause mortality Kaplan–Meier curves. ACEi, angiotensin-converting enzyme inhibitor; ARB, angiotensin II receptor blocker; CCB, calcium channel blocker.

**Figure 3:**
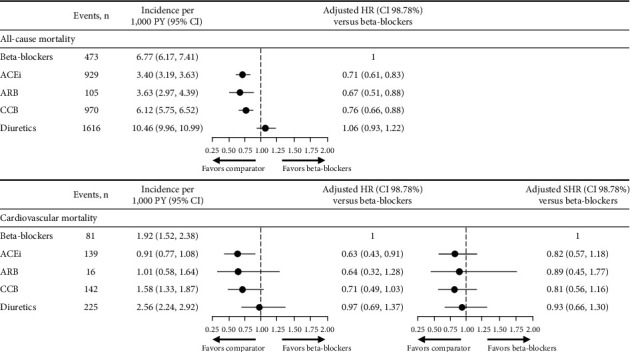
Risk of all-cause and cardiovascular mortality and Fine and Gray model for the event of cardiovascular mortality. Differences in risk of all-cause and cardiovascular mortality were assessed using adjusted Cox proportional hazard models (HR) and fine and gray proportional SHR, considering discontinuation as a competing event. Models were adjusted for age at index year; sex; time from hypertension diagnosis; smoking status; BMI; diastolic BP; systolic BP; angina; stroke; arrhythmia; chronic heart failure; myocardial infarction; peripheral vascular diseases; diabetes mellitus; dyslipidemia; and renal impairment. 98.7% CIs were generated using a Bonferroni correction (1–(0.05/4)) to account for multiple comparisons. ACEi, angiotensin-converting enzyme inhibitor; ARB, angiotensin II receptor blocker; BMI, body mass index; BP, blood pressure; CCB, calcium channel blocker; CI, confidence interval; HR, hazard ratio; PY, person years; SHR, sub-distribution hazard ratio.

The survival probabilities for all-cause mortality were displayed using the Kaplan–Meier estimator in Figure 2. No differences in the risk of all-cause mortality were observed between the beta-blocker and diuretics cohorts once adjusted for baseline confounders such as angina, diabetes mellitus, and BP. The risk of all-cause mortality was lower in patients treated with ACEi, ARB, and CCB compared with the beta-blocker cohort (Figure 3).

The risk of cardiovascular mortality was lower in patients treated with ACEi (adjusted hazard ratio (HR) (98.75% CI), 0.63 (0.43, 0.91)) compared with those receiving beta-blockers; there was no difference versus the other cohorts (Figure 3). The adjusted SHR did not show any differences in the risk of cardiovascular mortality with beta-blockers compared with ACEi, ARB, or CCB (Figure 3).

The HR reflects the probability of an event in the next instant, whereas the SHR reflects the probability of an event over the entire follow-up period. In this study, patients in the ACEi, ARB, and CCB cohorts had a longer follow-up compared with patients in the beta-blocker cohort. Therefore, the point estimate of the SHR was higher than the point estimate of the HR. Both adjusted HR and adjusted SHR point estimates indicated a trend toward an increased risk for all-cause and cardiovascular mortality with beta-blockers compared with ACEi, ARB, or CCB, but no differences compared with diuretics.

In contrast to the main analysis, the sensitivity analysis with IPTW showed an increased risk of all-cause mortality with diuretics (weighted HR versus beta-blockers for diuretics 1.32 (1.18, 1.49); Supplementary Tables 2–4) and did not show any difference between beta-blockers and CCB (weighted HR versus beta-blockers for CCB 0.96 (0.71, 1.30); Supplementary Tables 2–4).

### 3.3. Risk of Myocardial Infarction

**Figure 4:**
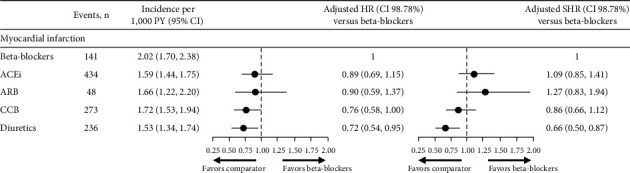
Risk of myocardial infarction and Fine and Gray model for the event of myocardial infarction. Differences in risk of myocardial infarction were assessed using adjusted Cox proportional hazard models (HR) and fine and gray proportional SHR, considering discontinuation as a competing event. Models were adjusted for age at index year; sex; time from hypertension diagnosis; smoking status; BMI; diastolic BP; systolic BP; angina; stroke; arrhythmia; chronic heart failure; myocardial infarction; peripheral vascular diseases; diabetes mellitus; dyslipidemia; and renal impairment. 98.7% CIs were generated using a Bonferroni correction (1–(0.05/4)) to account for multiple comparisons. ACEi, angiotensin-converting enzyme inhibitor; ARB, angiotensin II receptor blocker; BMI, body mass index; BP, blood pressure; CCB, calcium channel blocker; CI, confidence interval; HR, hazard ratio; PY, person years; SHR, sub-distribution hazard ratio.

There were no statistical differences in the risk of myocardial infarction in patients treated with ACEi and ARB compared with beta-blockers, and although this risk was lower for those treated with CCB, this was not significant. In the diuretic cohort, however, the risk of myocardial infarction was significantly lower in those treated with diuretics compared with beta-blockers (Figure 4). Similar results are observed with the adjusted SHR, which show a significant difference in the risk of myocardial infarction with those treated with diuretics, but not with CCB versus beta-blockers (Figure 4).

### 3.4. Risk of Stroke, Hemorrhagic and Ischemic Stroke

#### 3.4.1. Stroke

**Figure 5:**
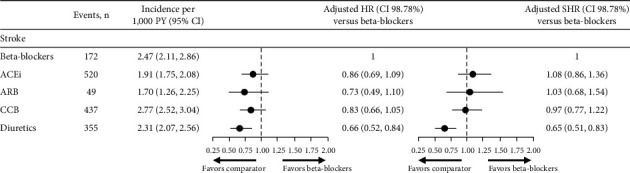
Risk of stroke and Fine and Gray model for the event of stroke. Differences in risk of stroke were assessed using adjusted Cox proportional hazard models (HR) and fine and gray proportional SHR, considering discontinuation as a competing event. Models were adjusted for age at index year; sex; time from hypertension diagnosis; smoking status; BMI; diastolic BP; systolic BP; angina; stroke; arrhythmia; chronic heart failure; myocardial infarction; peripheral vascular diseases; diabetes mellitus; dyslipidemia; and renal impairment. 98.7% CIs were generated using a Bonferroni correction (1–(0.05/4)) to account for multiple comparisons. ACEi, angiotensin-converting enzyme inhibitor; ARB, angiotensin II receptor blocker; BMI, body mass index; BP, blood pressure; CCB, calcium channel blocker; CI, confidence interval; HR, hazard ratio; PY, person years; SHR, sub-distribution hazard ratio.

Regarding the risk of stroke, there was no difference observed between the beta-blocker cohort and patients treated with ACEi, ARB, or CCB (adjusted HR (98.75% CI) for ACEi, 0.86 (0.69, 1.09), ARB, 0.73 (0.49, 1.10), and CCB, 0.83 (0.66, 1.05); Figure 5). The risk of stroke in patients treated with diuretics, however, was lower compared with the beta-blocker cohort (adjusted HR (98.75% CI) for diuretics 0.66 (0.52, 0.84); Figure 5). Although increased compared with the HR, the adjusted SHR drove similar conclusions for ACEi, ARB, and CCB cohorts. The point estimate of the SHR for diuretics was similar to the HR, indicating a lower risk of stroke compared with the beta-blocker cohort.

#### 3.4.2. Hemorrhagic Stroke

**Figure 6:**
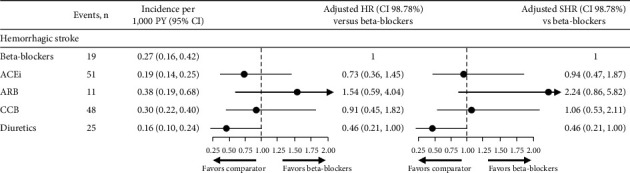
Risk of hemorrhagic stroke and Fine and Gray model for the event of hemorrhagic stroke. Differences in risk of hemorrhagic stroke were assessed using adjusted Cox proportional hazard models (HR) and fine and gray proportional SHR, considering discontinuation as a competing event. Models were adjusted for age at index year; sex; time from hypertension diagnosis; smoking status; BMI; diastolic BP; systolic BP; angina; stroke; arrhythmia; chronic heart failure; myocardial infarction; peripheral vascular diseases; diabetes mellitus; dyslipidemia; and renal impairment. 98.7% CIs were generated using a Bonferroni correction (1–(0.05/4)) to account for multiple comparisons. ACEi, angiotensin-converting enzyme inhibitor; ARB, angiotensin II receptor blocker; BMI, body mass index; BP, blood pressure; CCB, calcium channel blocker; CI, confidence interval; HR, hazard ratio; PY, person years; SHR, sub-distribution hazard ratio.

There was no difference in the risk of hemorrhagic stroke in the beta-blocker cohort compared with patients treated with ACEi, ARB, or CCB (Figure 6). There was also no significant difference in the risk of hemorrhagic stroke in patients treated with diuretics versus the beta-blocker cohort.

**Figure 7:**
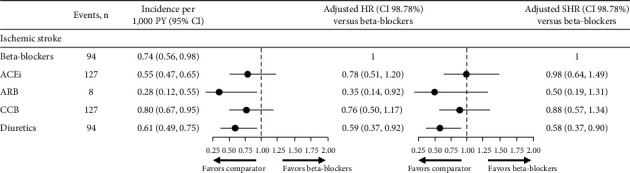
Risk of ischemic stroke and Fine and Gray model for the event of ischemic stroke. Differences in risk of ischemic stroke were assessed using adjusted Cox proportional hazard models (HR) and fine and gray proportional SHR, considering discontinuation as a competing event. Models were adjusted for age at index year; sex; time from hypertension diagnosis; smoking status; BMI; diastolic BP; systolic BP; angina; stroke; arrhythmia; chronic heart failure; myocardial infarction; peripheral vascular diseases; diabetes mellitus; dyslipidemia; and renal impairment. 98.7% CIs were generated using a Bonferroni correction (1–(0.05/4)) to account for multiple comparisons. ACEi, angiotensin-converting enzyme inhibitor; ARB, angiotensin II receptor blocker; BMI, body mass index; BP, blood pressure; CCB, calcium channel blocker; CI, confidence interval; HR, hazard ratio; PY, person years; SHR, sub-distribution hazard ratio.

No difference was observed in the risk of ischemic stroke between patients who received beta-blockers, ACEi, or CCB; however, this risk was lower in patients treated with ARB or diuretics (adjusted HR (98.75% CI), ARB 0.35 (0.14, 0.92), diuretics 0.59 (0.37, 0.92)) (Figure 7). In terms of the SHR, to the rates of each cerebrovascular outcome (stroke, hemorrhagic and ischemic stroke) were increased compared with the HR; however, the SHR for stroke for ACEi, ARB, and CCB drove a similar conclusion. The point estimate of SHR for diuretics was similar to the HR (Figure 7).

The cumulative incidence curves for the risk of cerebrocardiovascular-related mortality or cardiovascular events and cerebrovascular events are provided in Supplementary Figures 2–4. See Supplementary Figure 2 for cumulative incidence curves for cerebrocardiovascular mortality with only death from cerebrocardiovascular causes as event. Supplementary Figure 3 shows cumulative incidence curves for myocardial infarction. Supplementary Figure 4 shows cumulative incidence curves for stroke, hemorrhagic stroke, and ischemic stroke.

## 4. Discussion

In this noninterventional study, the risk of all-cause mortality was similar in patients treated with beta-blockers and diuretics, but lower in those treated with ACEi, ARB, or CCB. Additionally, the risk of cardiovascular mortality in patients treated with beta-blockers was comparable between all cohorts except for those who received ACEi, where the risk was lower. The sensitivity analysis with IPTW showed the same conclusions for ARB and ACEi and a comparable risk with CCB versus beta-blockers for all-cause mortality. Conversely, the sensitivity analysis with IPTW showed an increased risk of all-cause mortality with diuretics vs. beta-blockers. However, the propensity score method could have been affected by the extreme weights and moderate overlap across cohorts (Supplementary Figures 5–8; propensity score). In this case, the use of the IPTW method could produce less precise estimates than conventional adjustment [16]. After IPTW, the upper limit of the confidence for CCB was borderline significant (1.06), concordant with a trend towards a lower mortality.

This study builds on the limited real-world data assessing the effectiveness of antihypertensive monotherapy in reducing all-cause mortality, cerebrocardiovascular-related mortality, or cardiovascular and cerebrovascular events.

Other noninterventional studies have shown that beta-blockers offer comparable BP reductions to other antihypertensive drug classes [8]. Furthermore, a study looking into bisoprolol versus other antihypertensive classes to treat high BP found that bisoprolol had a similar antihypertensive effectiveness in terms of reducing BP compared with other antihypertensive agents (Merck data on file). Given the results of these studies, it was anticipated that these BP reductions would result in similar event rates across all five classes of antihypertensive monotherapies in the present study. One RCT demonstrated that a systolic/diastolic BP-lowering of 10/5 mmHg could prevent 8 deaths, 17 strokes, and 6 events of coronary heart disease for every 1,000 patients treated for 5 years, regardless of the therapeutic class administered [6]. Therefore, the reduction of these events is due to BP-lowering rather than specific drug properties.

There are also discrepancies between the results of the present study and those of meta-analyses in which beta-blocker-based therapy was demonstrated to be as effective as other classes of BP-lowering treatment at preventing all-cause mortality and myocardial infarction, and less effective at preventing stroke [6]. Furthermore, a network meta-analysis of clinical trials indicated that first-line antihypertension medications, including ACEi, dihydropyridine CCB, beta-blockers, ARB, and diuretics, were effective in reducing cardiovascular events compared with placebo; however, the differences between medication classes were generally small in terms of their associations with reducing cardiovascular events [17].

A study evaluating the effectiveness of bisoprolol in reducing the risk of mortality and cardiovascular outcomes in patients with hypertension showed a sustained benefit on survival, evident from 2 years after treatment initiation versus other beta-blockers. Additionally, there was a 5-year benefit with bisoprolol versus drugs other than beta-blockers [18].

These discrepancies could be explained by the difference in the selected populations and in the follow-up time. In clinical trials, the addition of another antihypertensive class to achieve BP targets might be planned in the protocol. However, in our study the population was censored at the addition of an antihypertensive class. Furthermore, the benefits of BP-lowering on all-cause mortality in hypertensive patients [6], as well as significant reduction in all major long-term events [4], may become apparent after several months of treatment. All patients in the RCT were treated for at least 1 year [7], whereas the median follow-up in the present study was only a few months, with beta-blocker and diuretic cohorts followed in median for less than 4.8 months and the other classes for less than a year. Therefore, this length of time may be insufficient to capture long-term effects related to a decrease in BP. This study is representative of the short-term cardiocerebrovascular effects of antihypertensive treatments.

In the present study, among the beta-blocker cohort of 44,404 patients, 75% were prescribed atenolol, 11% bisoprolol, and 8% propranolol. Guidelines such as those from NICE rely on the evidence from RCTs which mainly study atenolol [7]. Therefore, atenolol may be prescribed to patients with hypertension more frequently than other medications; the present study reflects this. More patients on highly beta-1-receptor-selective beta-blockers may have shown different results.

During the study period, prescription of the antihypertensives was consistent with NICE guidelines [5]. NICE guidelines recommend ACEi, ARB, or CCB as first-line treatment for hypertension, which is not the case for beta-blockers and diuretics. Beta-blockers were preferred in patients with a diagnosis of angina or arrhythmia, and ACEi and ARB were preferred in patients with metabolic syndrome such as diabetes mellitus or dyslipidemia, or in patients with asthma. These differences were adjusted for in the Cox proportional hazard and Fine and Gray proportional sub-distribution hazard models [5]. Due to this, the beta-blocker cohort presented different baseline characteristics. The adjusted model may have failed to compensate for some of these discrepancies; hence, biased estimates favoring the ACEi, ARB, and CCB cohorts were provided.

### 4.1. Strengths and Limitations

The strengths of the present study include the use of high-quality CPRD data with a breadth of coverage and size. A further strength of the methodology was the use of competing risk settings to consider different events such as discontinuation or death as competing events, compared with classical Cox models, which will consider single events in a cause-specific approach. The additional competing event analysis accounted for patient discontinuation and switching of drugs, which occurs in the real-world setting but not in RCTs. Furthermore, all models were adjusted for potential cofounders. This study also included a large sample size to assess real-world effectiveness of different antihypertensive drug classes. The study also adds to the limited real-world data assessing the effectiveness of beta-blockers only against each monotherapy antihypertensive class, particularly exploring the risk of mortality and cardiovascular and cerebrovascular events.

There were also several limitations to this study. First, this study may suffer from a selection bias. During the current study period, ACEi, ARB, or CCB was the preferred first-line treatment for hypertension [5] and beta-blockers were preferred in patients with increased sympathetic drive. Second, the short duration of follow-up was only representative of the short-term cardiocerebrovascular effects of antihypertensive treatments. The median follow-up was less than 1 year, and therefore, the study may have failed to capture long-term benefits associated with decreased BP. Third, as the inclusion period of this study started in 2000, the incidence of hypertension and other comorbidities was likely to be under-reported prior to the implementation of the Quality Outcomes Framework (QOF), an incentive scheme for general practitioners, in 2004 [19]. However, all the antihypertensive classes of interest were already marketed in 2000, suggesting that this potential bias is not expected to be different between the cohorts. Lastly, the effectiveness of the beta-blocker class is not homogenous [4] and refers to a mixed group of drugs with diverse properties such as cardioselectivity, sympathomimetic activity, and vasodilatation [7].

## 5. Conclusions

This real-world study assessed the relative effectiveness of antihypertensive monotherapies using a large UK database. All-cause mortality was lower in patients treated with ACEi, ARB, and CCB versus those treated with beta-blockers. However, the risk of cardiovascular mortality was similar for patients treated with beta-blockers, ARB, CCB, and diuretics, and lower in patients treated with ACEi. These data add to the limited pool of real-world studies comparing the long-term effectiveness of antihypertensive monotherapy drugs.

## Figures and Tables

**Table 1:** Patient baseline information and follow-up time per cohort.

	Beta-blockers *n* = 44,404	ACEi n = 132,545	ARB n = 12,018	CCB n = 91,731	Diuretics n = 106,547
Demographic characteristics					
Age (years)					
Median (p25; p75)	57 (49; 68)	54.0 (47; 64)	57.0 (49; 67)	64.0 (57; 72)	66.0 (56; 74)
<55, *n* (%)	18,363 (41.4%)	67,719 (51.1%)	5,039 (41.9%)	17,480 (19.1%)	22,160 (20.8%)
≥55, *n* (%)	26,041 (58.6%)	64,826 (48.9%)	6,979 (58.1%)	74,251 (80.9%)	84,387 (79.2%)
Sex					
Male, *n* (%)	22,250 (50.1%)	77,069 (58.1%)	6,822 (56.8%)	47,617 (51.9%)	42,396 (39.8%)

Clinical characteristics					
Time since HTN diagnosis (years)					
Median (p25; p75)	0.1 (0.0; 4.6)	0.0 (0.0; 0.1)	0.0 (0.0; 2.7)	0.0 (0.0; 0.1)	0.0 (0.0; 1.4)
Systolic BP (mmHg)					
Median (q1; q3)	163 (150; 180)	160 (150; 172)	160 (150; 176)	165 (155; 180)	167 (155; 180)
≥140	35,772 (88.4%)	118,820 (92.5%)	10,277 (90.8%)	83,312 (94.8%)	95,082 (94.6%)
Missing, *n* (%)	3,932 (8.9%)	4,071 (3.1%)	701 (5.8%)	3,809 (4.2%)	6,074 (5.7%)
Diastolic BP (mmHg)					
Median (p25; p75)	96 (88; 104)	96 (90; 102)	95 (88; 101)	94 (86; 100)	94 (86; 100)
≥90	29,546 (73.0%)	98,048 (76.3%)	8,278 (73.1%)	60,044 (68.3%)	70,566 (70.2%)
Missing, *n* (%)					
BMI (kg/m^2^)	3,946 (8.9%)	4,076 (3.1%)	694 (5.8%)	3,809 (4.2%)	6,080 (5.7%)
Median (p25; p75)	28.2 (25.1; 31.8)	29.6 (26.3; 33.5)	28.9 (25.8; 32.8)	28.2 (25.1; 31.8)	27.9 (24.9; 31.6)
<25, *n* (%)	3,975 (23.6%)	11,576 (15.8%)	1,072 (19.4%)	10,325 (23.9%)	11,659 (25.8%)
25–29, *n* (%)	6,727 (39.9%)	26,739 (36.6%)	2,118 (38.3%)	17,118 (39.6%)	17,987 (39.7%)
≥30, *n* (%)	6,148 (36.5%)	34,763 (47.6%)	2,344 (42.4%)	15,744 (36.5%)	15,631 (34.5%)
Missing, *n* (%)	27,554 (62.1%)	59,467 (44.9%)	6,484 (54.0%)	48,544 (52.9%)	61,269 (57.5%)
GFR					
Median (p25; p75)	74.2 (62.5; 86.2)	77.2 (61.1; 90.0)	73.9 (60.0; 87.0)	72.5 (60.0; 85.5)	70.4 (60.0; 82.5)
Missing, *n* (%)	24,317 (54.8%)	37,143 (28.0%)	5,086 (42.3%)	33,899 (37.0%)	51,210 (48.1%)

Smoking status					
Current smoker,					
n (%)	9,013 (20.3%)	27,749 (20.9%)	2,145 (17.8%)	16,635 (18.1%)	20,591 (19.3%)

Comorbidities and co-medications					
Disease of the circulatory system, n (%)					
Angina	1,257 (2.8%)	385 (0.3%)	50 (0.4%)	564 (0.6%)	479 (0.4%)
Arrhythmia	1,245 (2.8%)	787 (0.6%)	110 (0.9%)	701 (0.8%)	862 (0.8%)
Atrial fibrillation	1,071 (2.4%)	723 (0.5%)	103 (0.9%)	608 (0.7%)	805 (0.8%)
Chronic heart failure	57 (0.1%)	240 (0.2%)	17 (0.1%)	25 (0.0%)	412 (0.4%)
Myocardial infarction	406 (0.9%)	317 (0.2%)	14 (0.1%)	58 (0.1%)	95 (0.1%)
Peripheral vascular disease	96 (0.2%)	637 (0.5%)	57 (0.5%)	1,076 (1.2%)	585 (0.5%)
Stroke	325 (0.7%)	1,572 (1.2%)	86 (0.7%)	824 (0.9%)	887 (0.8%)
Hemorrhagic stroke	55 (0.1%)	231 (0.2%)	13 (0.1%)	181 (0.2%)	102 (0.1%)
Ischemic stroke	96 (0.2%)	533 (0.4%)	33 (0.3%)	244 (0.3%)	253 (0.2%)

Nutritional and metabolic diseases, n (%)					
Diabetes mellitus	1,521 (3.4%)	21,066 (15.9%)	1,323 (11.0%)	3,710 (4.0%)	3,182 (3.0%)
Dyslipidemia	1,801 (4.1%)	8,365 (6.3%)	766 (6.4%)	4,778 (5.2%)	4,251 (4.0%)
Diseases of the respiratory system, n (%)					
Asthma	440 (1.0%)	8,504 (6.4%)	765 (6.4%)	6,030 (6.6%)	6,059 (5.7%)
Diseases of the genitourinary system, n (%)					
Erectile dysfunction	349 (0.8%)	2,298 (1.7%)	209 (1.7%)	1,241 (1.4%)	927 (0.9%)
Renal impairment	263 (0.6%)	3,485 (2.6%)	280 (2.3%)	1,699 (1.9%)	824 (0.8%)

Co-medication at baseline, n (%)					
Anticoagulants	806 (1.8%)	1,634 (1.2%)	194 (1.6%)	1,255 (1.4%)	479 (0.4%)
Anti-anginals	2,929 (6.6%)	1,927 (1.5%)	219 (1.8%)	2,025 (2.2%)	862 (0.8%)
NSAID	9,682 (21.8%)	27,903 (21.1%)	2,570 (21.4%)	19,723 (21.5%)	805 (0.8%)
Platelet aggregation inhibitors	7,183 (16.2%)	15,583 (11.8%)	1,506 (12.5%)	11,020 (12.0%)	412 (0.4%)
Follow-up time (years)					
Median (q1; q3)	0.4 (0.1; 1.9)	0.8 (0.2; 2.9)	1.1 (0.2; 3.4)	0.6 (0.2; 2.5)	0.3 (0.1; 1.7)
Min; max	0.0; 18.0	0.0; 17.9	0.0; 17.9	0.0; 17.9	0.0; 17.9

ACEi, angiotensin-converting enzyme inhibitors; ARB, angiotensin II receptor blockers; bpm, beats per minute; BMI, body mass index; CCB, calcium channel blockers; GFR, glomerular filtration rate; HTN, hypertension; p25, 25th percentile; p75, 75th percentile; NSAID, nonsteroidal anti-inflammatory drug.

## Data Availability

The datasets generated and/or analyzed during the current study are not publicly available, in order to protect subject identification and privacy. Furthermore, restrictions apply to the availability of these data, which were used under license for the current study.

## Additional Points

What is already known about this topic? (i). Reducing blood pressure using antihypertensive drugs lowers the risk of cardiovascular disease and mortality. However, there is limited real-world evidence comparing the effectiveness of single antihypertensive classes in preventing all-cause mortality. What does this article add? (i) The study of a large UK patient database adds to the limited pool of real-world evidence demonstrating the effectiveness of beta-blockers and other long-term antihypertensive monotherapies on cardiovascular outcomes and all-cause mortality. (ii) The study findings are potentially more meaningful to healthcare practitioners than randomized clinical trial data as they better reflect hypertension management in routine clinical practice.

## Supplementary Materials

Supplementary Materials Supplementary Table 1. Antihypertensive drugs considered for each treatment of interest. Supplementary Tables 5–31. All code lists for exposure, covariates, and outcomes. Supplementary Figure 1. Patient attrition. Supplementary Table 2. Sensitivity analysis results for all-cause death and cardiovascular mortality with IPTW and Fine and Gray model for the event of cardiovascular mortality. Supplementary Table 3. Sensitivity analysis results for myocardial infarction with IPTW and fine and gray model. Supplementary Table 4. Sensitivity analysis results for cerebrovascular outcome with IPTW and fine and gray model. Supplementary Figure 2. Cumulative incidence curves for cerebrocardiovascular mortality with only death from cerebrocardiovascular causes as event. Supplementary Figure 3. Cumulative incidence curves for myocardial infarction. Supplementary Figure 4. Cumulative incidence curves for stroke, hemorrhagic stroke and ischemic stroke.
