# Supplementary material for: Real-World Effectiveness of Beta-Blockers versus Other Antihypertensives in Reducing All-Cause Mortality and Cardiovascular Events
Source: Int J Clin Pract. 2022 Jul 30;2022:6124559. doi: 10.1155/2022/6124559 (PMC9356871; doi:10.1155/2022/6124559)

**Supplementary file: Discussion (Section 4):**

**Supplementary Figures 5–8. Propensity score**

**Supplementary Figure 5. Propensity score distribution between beta-blockers and ACEi**


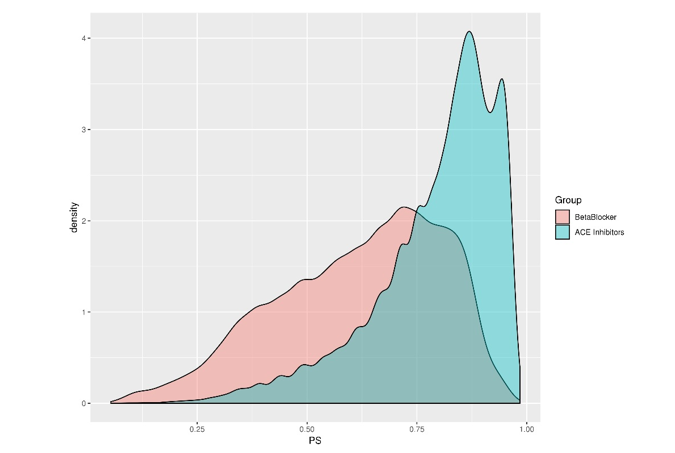


**Supplementary Figure 6. Propensity score distribution between beta-blockers and ARB**


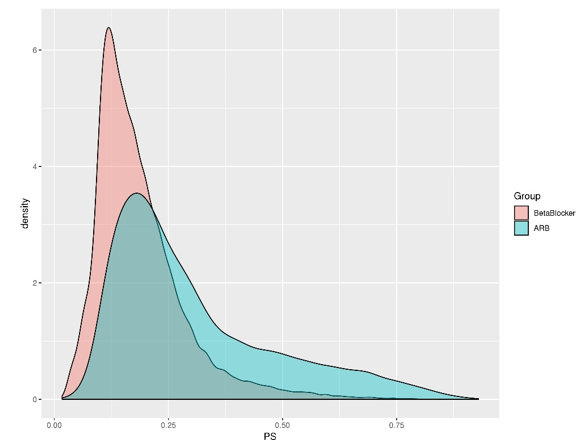


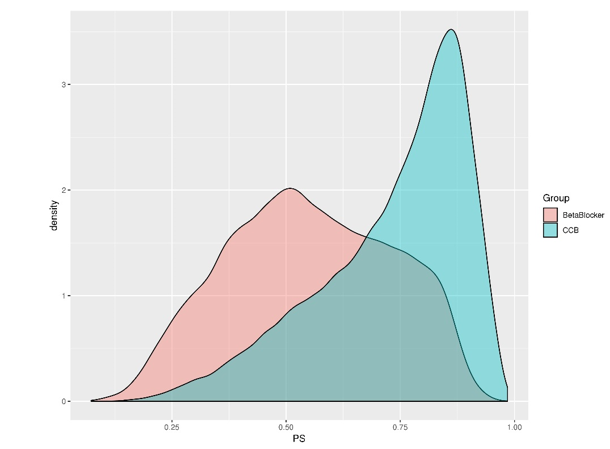


**Supplementary Figure 7. Propensity score distribution between beta-blockers and CCB**

**Supplementary Figure 8. Propensity score distribution between beta-blockers and diuretics**


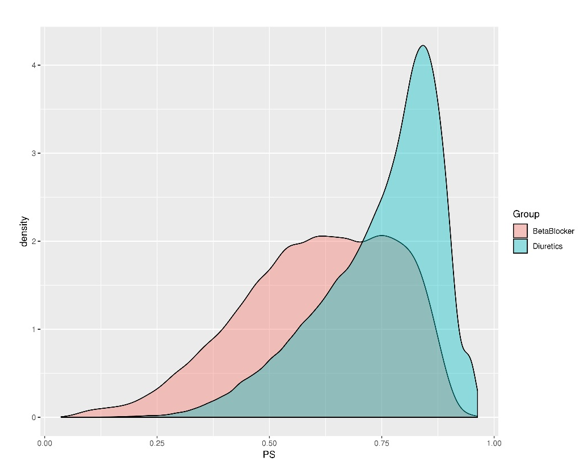

Supplement: Supplementary Materials — Supplementary Table 1. Antihypertensive drugs considered for each treatment of interest. Supplementary Tables 5–31. All code lists for exposure, covariates, and outcomes. Supplementary Figure 1. Patient attrition. Supplementary Table 2. Sensitivity analysis results for all-cause death and cardiovascular mortality with IPTW and Fine and Gray model for the event of cardiovascular mortality. Supplementary Table 3. Sensitivity analysis results for myocardial infarction with IPTW and fine and gray model. Supplementary Table 4. Sensitivity analysis results for cerebrovascular outcome with IPTW and fine and gray model. Supplementary Figure 2. Cumulative incidence curves for cerebrocardiovascular mortality with only death from cerebrocardiovascular causes as event. Supplementary Figure 3. Cumulative incidence curves for myocardial infarction. Supplementary Figure 4. Cumulative incidence curves for stroke, hemorrhagic stroke and ischemic stroke. [file 6124559.f1.zip › 6124559.f1/Supplementary file_Discussion (Section 4).docx]
