# Supplementary material for: Real-World Effectiveness of Beta-Blockers versus Other Antihypertensives in Reducing All-Cause Mortality and Cardiovascular Events
Source: Int J Clin Pract. 2022 Jul 30;2022:6124559. doi: 10.1155/2022/6124559 (PMC9356871; doi:10.1155/2022/6124559)
Supplement: Supplementary Materials — Supplementary Table 1. Antihypertensive drugs considered for each treatment of interest. Supplementary Tables 5–31. All code lists for exposure, covariates, and outcomes. Supplementary Figure 1. Patient attrition. Supplementary Table 2. Sensitivity analysis results for all-cause death and cardiovascular mortality with IPTW and Fine and Gray model for the event of cardiovascular mortality. Supplementary Table 3. Sensitivity analysis results for myocardial infarction with IPTW and fine and gray model. Supplementary Table 4. Sensitivity analysis results for cerebrovascular outcome with IPTW and fine and gray model. Supplementary Figure 2. Cumulative incidence curves for cerebrocardiovascular mortality with only death from cerebrocardiovascular causes as event. Supplementary Figure 3. Cumulative incidence curves for myocardial infarction. Supplementary Figure 4. Cumulative incidence curves for stroke, hemorrhagic stroke and ischemic stroke. [file 6124559.f1.zip › 6124559.f1/Supplementary file_Methods (Section 2.4).docx]

**Supplementary file: Methods (Section 2.4)**

**Supplementary Table 1: Antihypertensive drugs considered for each treatment of interest**

| Cohort | Antihypertensive drugs considered |
| --- | --- |
| Beta-blockers | acebutolol, atenolol, betaxolol, bisoprolol, carteolol, carvedilol, celiprolol, labetalol, metoprolol, nadolol, nebivolol, oxprenolol, pindolol, propranolol, timolol |
| ACEi | captopril, cilazapril, enalapril, fosinopril, imidapril, lisinopril, moexipril, perindopril, quinapril, ramipril, trandolapril |
| ARB | azilsartan, candesartan, eprosartan, irbesartan, losartan, olmesartan, telmisartan, valsartan |
| CCB | amlodipine, diltiazem, felodipine, isradipine, lacidipine, lercanidipine, mibefradil, nicardipine, nifedipine, nimodipine, nisoldipine, verapamil |
| Diuretics | amiloride, bendroflumethiazide, bumetanide, chlorothiazide, chlortalidone, clopamide, cyclopenthiazide, eplerenone, etacrynic acid, furosemide, hydrochlorothiazide, hydroflumethiazide, indapamide, mefruside, methyciothiazide, metolazone, piretanide,  polythiazide, spironolactone, torasemide, triamterene, xipamide |

ACEi, angiotensin-converting enzyme inhibitor; ARB, angiotensin II receptor blockers; CCB, calcium channel blockers
