# Supplementary material for: Real-World Effectiveness of Beta-Blockers versus Other Antihypertensives in Reducing All-Cause Mortality and Cardiovascular Events
Source: Int J Clin Pract. 2022 Jul 30;2022:6124559. doi: 10.1155/2022/6124559 (PMC9356871; doi:10.1155/2022/6124559)
Supplement: Supplementary Materials — Supplementary Table 1. Antihypertensive drugs considered for each treatment of interest. Supplementary Tables 5–31. All code lists for exposure, covariates, and outcomes. Supplementary Figure 1. Patient attrition. Supplementary Table 2. Sensitivity analysis results for all-cause death and cardiovascular mortality with IPTW and Fine and Gray model for the event of cardiovascular mortality. Supplementary Table 3. Sensitivity analysis results for myocardial infarction with IPTW and fine and gray model. Supplementary Table 4. Sensitivity analysis results for cerebrovascular outcome with IPTW and fine and gray model. Supplementary Figure 2. Cumulative incidence curves for cerebrocardiovascular mortality with only death from cerebrocardiovascular causes as event. Supplementary Figure 3. Cumulative incidence curves for myocardial infarction. Supplementary Figure 4. Cumulative incidence curves for stroke, hemorrhagic stroke and ischemic stroke. [file 6124559.f1.zip › 6124559.f1/Supplementary file_Methods (Section 2.6).docx]

**Supplementary file: Methods (Section 2.6)**

**Supplementary Tables 5–32. All code lists for exposure, covariates and outcomes**

**Supplementary Table 5. Code list for hypertension**

| **Hypertension** |  |  |
| --- | --- | --- |
| **medcode** | **readcode** | **readterm** |
| 32976 | 6146200 | Hypertension induced by oral contraceptive pill |
| 109611 | 661M600 | Hypertension self-management plan agreed |
| 110631 | 661N600 | Hypertension self-management plan review |
| 16565 | 6627.00 | Good hypertension control |
| 27511 | 6628.00 | Poor hypertension control |
| 18590 | 662b.00 | Moderate hypertension control |
| 21826 | 662F.00 | Hypertension treatm. started |
| 13188 | 662G.00 | Hypertensive treatm.changed |
| 12948 | 662H.00 | Hypertension treatm.stopped |
| 3425 | 662O.00 | On treatment for hypertension |
| 85944 | 7Q01.00 | High cost hypertension drugs |
| 101649 | 7Q01y00 | Other specified high cost hypertension drugs |
| 18057 | 8B26.00 | Antihypertensive therapy |
| 11056 | 8BL0.00 | Patient on maximal tolerated antihypertensive therapy |
| 12680 | 8CR4.00 | Hypertension clinical management plan |
| 22333 | 8I3N.00 | Hypertension treatment refused |
| 204 | G2...00 | Hypertensive disease |
| 8732 | G2...11 | BP - hypertensive disease |
| 799 | G20..00 | Essential hypertension |
| 351 | G20..11 | High blood pressure |
| 107704 | G20..12 | Primary hypertension |
| 15377 | G200.00 | Malignant essential hypertension |
| 1894 | G201.00 | Benign essential hypertension |
| 4372 | G202.00 | Systolic hypertension |
| 83473 | G203.00 | Diastolic hypertension |
| 10818 | G20z.00 | Essential hypertension NOS |
| 3712 | G20z.11 | Hypertension NOS |
| 7329 | G24..00 | Secondary hypertension |
| 31755 | G240.00 | Secondary malignant hypertension |
| 59383 | G240000 | Secondary malignant renovascular hypertension |
| 73293 | G240z00 | Secondary malignant hypertension NOS |
| 57288 | G241.00 | Secondary benign hypertension |
| 25371 | G241000 | Secondary benign renovascular hypertension |
| 51635 | G241z00 | Secondary benign hypertension NOS |
| 34744 | G244.00 | Hypertension secondary to endocrine disorders |
| 16059 | G24z.00 | Secondary hypertension NOS |
| 31387 | G24z000 | Secondary renovascular hypertension NOS |
| 31341 | G24z100 | Hypertension secondary to drug |
| 42229 | G24zz00 | Secondary hypertension NOS |
| 105371 | G25..00 | Stage 1 hypertension (NICE - Nat Ins for Hth Clin Excl 2011) |
| 105316 | G25..11 | Stage 1 hypertension |
| 105989 | G26..00 | Severe hypertension (Nat Inst for Health Clinical Ex 2011) |
| 105487 | G26..11 | Severe hypertension |
| 105480 | G27..00 | Hypertension resistant to drug therapy |
| 105274 | G28..00 | Stage 2 hypertension (NICE - Nat Ins for Hth Clin Excl 2011) |
| 18765 | G2y..00 | Other specified hypertensive disease |
| 7057 | G2z..00 | Hypertensive disease NOS |
| 69753 | Gyu2.00 | [X]Hypertensive diseases |
| 102458 | Gyu2000 | [X]Other secondary hypertension |
| 97533 | Gyu2100 | [X]Hypertension secondary to other renal disorders |

**Supplementary Table 6. Code list for beta-blockers**

| **Beta-blockers** | |  |  |  |  |
| --- | --- | --- | --- | --- | --- |
| **Prod**  **code** | **Gemscriptc**  **ode** | **Product**  **name** | **Drug**  **Substance**  **name** | **To include** | **combination therapy to exclude** |
| 8113 | 59925020 | Acebutolol 200mg capsules | Acebutolol hydrochloride | 1 |  |
| 8023 | 52703020 | Sectral 400mg tablets (Sanofi) | Acebutolol hydrochloride | 1 |  |
| 65438 | 71696020 | Acebutolol 100mg capsules (A A H Pharmaceuticals Ltd) | Acebutolol hydrochloride | 1 |  |
| 12296 | 52701020 | Sectral 100mg capsules (Sanofi) | Acebutolol hydrochloride | 1 |  |
| 8172 | 59924020 | Acebutolol 100mg capsules | Acebutolol hydrochloride | 1 |  |
| 8555 | 52702020 | Sectral 200mg capsules (Sanofi) | Acebutolol hydrochloride | 1 |  |
| 7620 | 59926020 | Acebutolol 400mg tablets | Acebutolol hydrochloride | 1 |  |
| 45309 | 71703020 | Acebutolol 400mg tablets (A A H Pharmaceuticals Ltd) | Acebutolol hydrochloride | 1 |  |
| 14126 | 59935020 | Acebutolol 200mg / Hydrochlorothiazide 12.5mg tablets | Acebutolol hydrochloride/Hydrochlorothiazide | 0 | 1 |
| 8189 | 51499020 | Secadrex 200mg/12.5mg tablets (Sanofi) | Acebutolol hydrochloride/Hydrochlorothiazide | 0 | 1 |
| 65027 | 44190021 | Bisoprolol 5mg / Aspirin 100mg capsules | Aspirin/Bisoprolol fumarate | 1 | 0 |
| 61573 | 30311020 | Atenolol 25mg/5ml oral solution | Atenolol | 1 |  |
| 53826 | 1006020 | Atenolol 25mg tablets (Boston Healthcare Ltd) | Atenolol | 1 |  |
| 44858 | 56835020 | Atenolol 25mg tablets (Actavis UK Ltd) | Atenolol | 1 |  |
| 51998 | 1011020 | Atenolol 25mg tablets (Strides Shasun (UK) Ltd) | Atenolol | 1 |  |
| 34976 | 59302020 | Atenolol 25mg tablets (Tillomed Laboratories Ltd) | Atenolol | 1 |  |
| 33650 | 60232020 | Atenolol 50mg tablets (Mylan) | Atenolol | 1 |  |
| 53204 | 973020 | Atenolol 50mg tablets (Alliance Healthcare (Distribution) Ltd) | Atenolol | 1 |  |
| 33850 | 56836020 | Atenolol 50mg tablets (Actavis UK Ltd) | Atenolol | 1 |  |
| 19172 | 57790020 | Atenolol 25mg tablets (IVAX Pharmaceuticals UK Ltd) | Atenolol | 1 |  |
| 13394 | 51861020 | Tenormin 25mg/5ml syrup (AstraZeneca UK Ltd) | Atenolol | 1 |  |
| 21133 | 86681020 | Atenamin 50mg Tablet (OPD Pharm) | Atenolol | 1 |  |
| 30636 | 72687020 | Vasaten 50mg Tablet (Shire Pharmaceuticals Ltd) | Atenolol | 1 |  |
| 46908 | 56095020 | Atenolol 100mg tablets (Kent Pharmaceuticals Ltd) | Atenolol | 1 |  |
| 33085 | 51023020 | Atenolol 100mg tablets (A A H Pharmaceuticals Ltd) | Atenolol | 1 |  |
| 59982 | 1012020 | Atenolol 25mg tablets (Accord Healthcare Ltd) | Atenolol | 1 |  |
| 34882 | 52909020 | Atenolol 50mg Tablet (Berk Pharmaceuticals Ltd) | Atenolol | 1 |  |
| 34365 | 53708020 | Atenolol 50mg tablets (Teva UK Ltd) | Atenolol | 1 |  |
| 31536 | 56093020 | Atenolol 25mg tablets (Kent Pharmaceuticals Ltd) | Atenolol | 1 |  |
| 66548 | 16660021 | Atenolol 50mg tablets (DE Pharmaceuticals) | Atenolol | 1 |  |
| 59695 | 980020 | Atenolol 50mg tablets (Boston Healthcare Ltd) | Atenolol | 1 |  |
| 34585 | 51440020 | Atenolol 25mg tablets (Sandoz Ltd) | Atenolol | 1 |  |
| 20502 | 72697020 | Atenix 100 tablets (Ashbourne Pharmaceuticals Ltd) | Atenolol | 1 |  |
| 34265 | 51438020 | Atenolol 50mg tablets (Sandoz Ltd) | Atenolol | 1 |  |
| 2587 | 51860020 | Tenormin 100mg tablets (AstraZeneca UK Ltd) | Atenolol | 1 |  |
| 5 | 60153020 | Atenolol 50mg tablets | Atenolol | 1 |  |
| 20728 | 86680020 | Atenamin 25mg Tablet (OPD Pharm) | Atenolol | 1 |  |
| 54542 | 1008020 | Atenolol 25mg tablets (Zanza Laboratories Ltd) | Atenolol | 1 |  |
| 33092 | 51022020 | Atenolol 50mg tablets (A A H Pharmaceuticals Ltd) | Atenolol | 1 |  |
| 19191 | 53707020 | Atenolol 100mg tablets (Teva UK Ltd) | Atenolol | 1 |  |
| 53802 | 1007020 | Atenolol 25mg tablets (Sigma Pharmaceuticals Plc) | Atenolol | 1 |  |
| 33184 | 52914020 | Atenolol 100mg tablets (Wockhardt UK Ltd) | Atenolol | 1 |  |
| 29368 | 53709020 | Atenolol 25mg tablets (Teva UK Ltd) | Atenolol | 1 |  |
| 57817 | 74385020 | Atenolol 50mg tablets (Zentiva) | Atenolol | 1 |  |
| 19182 | 55394020 | Atenolol 50mg tablets (IVAX Pharmaceuticals UK Ltd) | Atenolol | 1 |  |
| 17322 | 72698020 | Atenix 25 tablets (Ashbourne Pharmaceuticals Ltd) | Atenolol | 1 |  |
| 55778 | 983020 | Atenolol 50mg tablets (Phoenix Healthcare Distribution Ltd) | Atenolol | 1 |  |
| 54752 | 984020 | Atenolol 50mg tablets (Strides Shasun (UK) Ltd) | Atenolol | 1 |  |
| 34695 | 56094020 | Atenolol 50mg tablets (Kent Pharmaceuticals Ltd) | Atenolol | 1 |  |
| 10191 | 72696020 | Atenix 50 tablets (Ashbourne Pharmaceuticals Ltd) | Atenolol | 1 |  |
| 69526 | 30663020 | Atenolol 50mg/5ml oral solution | Atenolol | 1 |  |
| 26211 | 68815020 | Antipressan 25mg tablets (Teva UK Ltd) | Atenolol | 1 |  |
| 24 | 60152020 | Atenolol 100mg tablets | Atenolol | 1 |  |
| 47870 | 70250020 | Atenolol 25mg tablets (Almus Pharmaceuticals Ltd) | Atenolol | 1 |  |
| 24195 | 68814020 | Antipressan 100mg tablets (Teva UK Ltd) | Atenolol | 1 |  |
| 49953 | 1009020 | Atenolol 25mg tablets (Bristol Laboratories Ltd) | Atenolol | 1 |  |
| 24191 | 68813020 | Antipressan 50mg tablets (Teva UK Ltd) | Atenolol | 1 |  |
| 34492 | 60231020 | Atenolol 25mg tablets (Mylan) | Atenolol | 1 |  |
| 2590 | 72526020 | Tenormin 25mg tablets (AstraZeneca UK Ltd) | Atenolol | 1 |  |
| 26 | 67920020 | Atenolol 25mg tablets | Atenolol | 1 |  |
| 36261 | 59303020 | Atenolol 50mg tablets (Tillomed Laboratories Ltd) | Atenolol | 1 |  |
| 6066 | 67919020 | Atenolol 25mg/5ml oral solution sugar free | Atenolol | 1 |  |
| 53414 | 985020 | Atenolol 50mg tablets (Accord Healthcare Ltd) | Atenolol | 1 |  |
| 34575 | 52915020 | Atenolol 25mg tablets (Wockhardt UK Ltd) | Atenolol | 1 |  |
| 18950 | 72693020 | Totamol 25mg Tablet (C P Pharmaceuticals Ltd) | Atenolol | 1 |  |
| 33657 | 51024020 | Atenolol 25mg tablets (A A H Pharmaceuticals Ltd) | Atenolol | 1 |  |
| 52500 | 70253020 | Atenolol 50mg tablets (Almus Pharmaceuticals Ltd) | Atenolol | 1 |  |
| 34443 | 52913020 | Atenolol 50mg tablets (Wockhardt UK Ltd) | Atenolol | 1 |  |
| 31934 | 55395020 | Atenolol 100mg tablets (IVAX Pharmaceuticals UK Ltd) | Atenolol | 1 |  |
| 29398 | 86682020 | Atenamin 100mg Tablet (OPD Pharm) | Atenolol | 1 |  |
| 50702 | 998020 | Atenolol 25mg tablets (Alliance Healthcare (Distribution) Ltd) | Atenolol | 1 |  |
| 34754 | 51439020 | Atenolol 100mg tablets (Sandoz Ltd) | Atenolol | 1 |  |
| 64973 | 52168021 | Atenolol 50mg tablets (Sigma Pharmaceuticals Plc) | Atenolol | 1 |  |
| 15176 | 72691020 | Totamol 50mg Tablet (C P Pharmaceuticals Ltd) | Atenolol | 1 |  |
| 71026 | 70257020 | Atenolol 100mg tablets (Almus Pharmaceuticals Ltd) | Atenolol | 1 |  |
| 62325 | 47104020 | Atenolol 25mg tablets (Waymade Healthcare Plc) | Atenolol | 1 |  |
| 33079 | 60233020 | Atenolol 100mg tablets (Mylan) | Atenolol | 1 |  |
| 2432 | 54818020 | Tenormin LS 50mg tablets (AstraZeneca UK Ltd) | Atenolol | 1 |  |
| 15730 | 72692020 | Totamol 100mg Tablet (C P Pharmaceuticals Ltd) | Atenolol | 1 |  |
| 70135 | 16662021 | Atenolol 25mg tablets (DE Pharmaceuticals) | Atenolol | 1 |  |
| 51643 | 1013020 | Atenolol 25mg/5ml oral solution sugar free (Alliance Healthcare (Distribution) Ltd) | Atenolol | 1 |  |
| 52310 | 39793020 | Atenolol 25mg tablets (Crescent Pharma Ltd) | Atenolol | 1 |  |
| 46931 | 56837020 | Atenolol 100mg tablets (Actavis UK Ltd) | Atenolol | 1 |  |
| 56445 | 77037020 | Atenolol 25mg/5ml oral solution sugar free (A A H Pharmaceuticals Ltd) | Atenolol | 1 |  |
| 53215 | 982020 | Atenolol 50mg tablets (Bristol Laboratories Ltd) | Atenolol | 1 |  |
| 7543 | 56162020 | Kalten capsules (M & A Pharmachem Ltd) | Atenolol/Amiloride hydrochloride/Hydrochlorothiazide | 0 | 1 |
| 4983 | 68844020 | Atenolol with amiloride and hydrochlorothiazide capsules | Atenolol/Amiloride Hydrochloride/Hydrochlorothiazide | 0 | 1 |
| 3526 | 69296020 | Amiloride with atenolol with hydrochlorothiazide capsules | Atenolol/Amiloride Hydrochloride/Hydrochlorothiazide | 0 | 1 |
| 28177 | 68841020 | Hydrochlorothiazide with atenolol and amiloride Capsule | Atenolol/Amiloride Hydrochloride/Hydrochlorothiazide | 0 | 1 |
| 9178 | 83986020 | Atenolol 25mg / Bendroflumethiazide 1.25mg capsules | Atenolol/Bendroflumethiazide | 0 | 1 |
| 18743 | 83989020 | Tenben 25mg/1.25mg capsules (Galen Ltd) | Atenolol/Bendroflumethiazide | 0 | 1 |
| 1288 | 51857020 | Tenoret 50mg/12.5mg tablets (AstraZeneca UK Ltd) | Atenolol/Chlortalidone | 0 | 1 |
| 5721 | 73883020 | Co-tenidone 100mg/25mg tablets | Atenolol/Chlortalidone | 0 | 1 |
| 34449 | 60352020 | Co-tenidone 50mg/12.5mg tablets (Mylan) | Atenolol/Chlortalidone | 0 | 1 |
| 46952 | 56911020 | Co-tenidone 100mg/25mg tablets (Actavis UK Ltd) | Atenolol/Chlortalidone | 0 | 1 |
| 34034 | 55247020 | Co-tenidone 50mg/12.5mg tablets (IVAX Pharmaceuticals UK Ltd) | Atenolol/Chlortalidone | 0 | 1 |
| 62537 | 21452021 | Co-tenidone 100mg/25mg tablets (DE Pharmaceuticals) | Atenolol/Chlortalidone | 0 | 1 |
| 24280 | 80150020 | Totaretic 100mg+25mg Tablet (C P Pharmaceuticals Ltd) | Atenolol/Chlortalidone | 0 | 1 |
| 31708 | 56910020 | Co-tenidone 50mg/12.5mg tablets (Actavis UK Ltd) | Atenolol/Chlortalidone | 0 | 1 |
| 31470 | 57164020 | Tenchlor 50mg/12.5mg tablets (Teva UK Ltd) | Atenolol/Chlortalidone | 0 | 1 |
| 32094 | 53521020 | Co-tenidone 50mg/12.5mg tablets (A A H Pharmaceuticals Ltd) | Atenolol/Chlortalidone | 0 | 1 |
| 26741 | 80149020 | Totaretic 50mg+12.5mg Tablet (C P Pharmaceuticals Ltd) | Atenolol/Chlortalidone | 0 | 1 |
| 1124 | 54815020 | Tenoretic 100mg/25mg tablets (AstraZeneca UK Ltd) | Atenolol/Chlortalidone | 0 | 1 |
| 26248 | 57165020 | Tenchlor 100mg/25mg tablets (Teva UK Ltd) | Atenolol/Chlortalidone | 0 | 1 |
| 34825 | 54653020 | Co-tenidone 50mg/12.5mg tablets (Teva UK Ltd) | Atenolol/Chlortalidone | 0 | 1 |
| 41572 | 54654020 | Co-tenidone 100mg/25mg tablets (Teva UK Ltd) | Atenolol/Chlortalidone | 0 | 1 |
| 34899 | 53522020 | Co-tenidone 100mg/25mg tablets (A A H Pharmaceuticals Ltd) | Atenolol/Chlortalidone | 0 | 1 |
| 13526 | 63763020 | Atenix Co 100 tablets (Ashbourne Pharmaceuticals Ltd) | Atenolol/Chlortalidone | 0 | 1 |
| 37725 | 60353020 | Co-tenidone 100mg/25mg tablets (Mylan) | Atenolol/Chlortalidone | 0 | 1 |
| 34012 | 55246020 | Co-tenidone 100mg/25mg tablets (IVAX Pharmaceuticals UK Ltd) | Atenolol/Chlortalidone | 0 | 1 |
| 9783 | 73882020 | Co-tenidone 50mg/12.5mg tablets | Atenolol/Chlortalidone | 0 | 1 |
| 21873 | 63762020 | Atenix Co 50 tablets (Ashbourne Pharmaceuticals Ltd) | Atenolol/Chlortalidone | 0 | 1 |
| 4542 | 68847020 | Atenolol 50mg / Nifedipine 20mg modified-release capsules | Atenolol/Nifedipine | 0 | 1 |
| 52728 | 1077020 | Beta-Adalat modified-release capsules (Lexon (UK) Ltd) | Atenolol/Nifedipine | 0 | 1 |
| 61719 | 1072020 | Beta-Adalat modified-release capsules (Waymade Healthcare Plc) | Atenolol/Nifedipine | 0 | 1 |
| 8642 | 68652020 | Tenif 50mg/20mg modified-release capsules (AstraZeneca UK Ltd) | Atenolol/Nifedipine | 0 | 1 |
| 68020 | 1075020 | Beta-Adalat modified-release capsules (Sigma Pharmaceuticals Plc) | Atenolol/Nifedipine | 0 | 1 |
| 1684 | 68629020 | Beta-Adalat modified-release capsules (Bayer Plc) | Atenolol/Nifedipine | 0 | 1 |
| 12141 | 64189020 | Betaxolol 20mg tablets | Betaxolol hydrochloride | 1 |  |
| 12519 | 64192020 | Kerlone 20mg tablets (Sanofi-Synthelabo Ltd) | Betaxolol hydrochloride | 1 |  |
| 43251 | 75323020 | Bisoprolol 1.25mg tablets (Mylan) | Bisoprolol fumarate | 1 |  |
| 58763 | 47116020 | Bisoprolol 2.5mg tablets (Waymade Healthcare Plc) | Bisoprolol fumarate | 1 |  |
| 63535 | 1085020 | Bisoprolol 5mg tablets (Relonchem Ltd) | Bisoprolol fumarate | 1 |  |
| 17615 | 83190020 | Cardicor 5mg tablets (Merck Serono Ltd) | Bisoprolol fumarate | 1 |  |
| 52611 | 31454020 | Bisoprolol 10mg/5ml oral solution | Bisoprolol fumarate | 1 |  |
| 33909 | 90617020 | Congescor 1.25mg tablets (Tillomed Laboratories Ltd) | Bisoprolol fumarate | 1 |  |
| 19178 | 62996020 | Bisoprolol 10mg tablets (Ranbaxy (UK) Ltd) | Bisoprolol fumarate | 1 |  |
| 56240 | 40506020 | Bisoprolol 3.75mg tablets (Sandoz Ltd) | Bisoprolol fumarate | 1 |  |
| 472 | 68438020 | Bisoprolol 5mg tablets | Bisoprolol fumarate | 1 |  |
| 55791 | 40507020 | Bisoprolol 3.75mg tablets (Actavis UK Ltd) | Bisoprolol fumarate | 1 |  |
| 58498 | 15612021 | Bisoprolol 2.5mg tablets (Medreich Plc) | Bisoprolol fumarate | 1 |  |
| 62407 | 94413020 | Bisoprolol oral solution | Bisoprolol Fumarate | 1 |  |
| 52686 | 31458020 | Bisoprolol 2.5mg/5ml oral solution | Bisoprolol fumarate | 1 |  |
| 822 | 91125020 | Bisoprolol 1.5mg/5ml oral suspension | Bisoprolol Fumarate | 1 |  |
| 63493 | 77726020 | Bisoprolol 2.5mg tablets (Actavis UK Ltd) | Bisoprolol fumarate | 1 |  |
| 52548 | 78457020 | Bisoprolol 1.25mg tablets (Almus Pharmaceuticals Ltd) | Bisoprolol fumarate | 1 |  |
| 3588 | 68353020 | Monocor 5mg tablets (Wyeth Pharmaceuticals) | Bisoprolol fumarate | 1 |  |
| 594 | 83178020 | Bisoprolol 2.5mg tablets | Bisoprolol fumarate | 1 |  |
| 53664 | 40505020 | Bisoprolol 2.5mg tablets (Sandoz Ltd) | Bisoprolol fumarate | 1 |  |
| 61651 | 1149020 | Bisoprolol 7.5mg tablets (Almus Pharmaceuticals Ltd) | Bisoprolol fumarate | 1 |  |
| 10892 | 68369020 | Emcor 10mg tablets (Merck Serono Ltd) | Bisoprolol fumarate | 1 |  |
| 14058 | 83182020 | Cardicor 1.25mg tablets (Merck Serono Ltd) | Bisoprolol fumarate | 1 |  |
| 38991 | 75624020 | Bisoprolol 7.5mg tablets (A A H Pharmaceuticals Ltd) | Bisoprolol fumarate | 1 |  |
| 24083 | 62681020 | Bisoprolol 5mg tablets (Teva UK Ltd) | Bisoprolol fumarate | 1 |  |
| 50514 | 1122020 | Bisoprolol 2.5mg tablets (Chanelle Medical UK Ltd) | Bisoprolol fumarate | 1 |  |
| 34821 | 62677020 | Bisoprolol 10mg tablets (Mylan) | Bisoprolol fumarate | 1 |  |
| 47041 | 75328020 | Bisoprolol 2.5mg tablets (Mylan) | Bisoprolol fumarate | 1 |  |
| 18185 | 83191020 | Cardicor 7.5mg tablets (Merck Serono Ltd) | Bisoprolol fumarate | 1 |  |
| 53885 | 74139020 | Bisoprolol 1.25mg tablets (A A H Pharmaceuticals Ltd) | Bisoprolol fumarate | 1 |  |
| 59148 | 41897020 | Bisoprolol 2.5mg tablets (Zentiva) | Bisoprolol fumarate | 1 |  |
| 58974 | 1116020 | Bisoprolol 2.5mg tablets (Alliance Healthcare (Distribution) Ltd) | Bisoprolol fumarate | 1 |  |
| 19200 | 63135020 | Bisoprolol 5mg tablets (IVAX Pharmaceuticals UK Ltd) | Bisoprolol fumarate | 1 |  |
| 69156 | 15613021 | Bisoprolol 3.75mg tablets (Medreich Plc) | Bisoprolol fumarate | 1 |  |
| 55929 | 45144020 | Bisoprolol 5mg tablets (Accord Healthcare Ltd) | Bisoprolol fumarate | 1 |  |
| 61340 | 38485020 | Bisoprolol 5mg tablets (DE Pharmaceuticals) | Bisoprolol fumarate | 1 |  |
| 59495 | 43811020 | Bisoprolol 1.25mg tablets (Teva UK Ltd) | Bisoprolol fumarate | 1 |  |
| 63850 | 43812020 | Bisoprolol 2.5mg tablets (Teva UK Ltd) | Bisoprolol fumarate | 1 |  |
| 61564 | 47117020 | Bisoprolol 3.75mg tablets (Waymade Healthcare Plc) | Bisoprolol fumarate | 1 |  |
| 37118 | 73449020 | Bisoprolol 2.5mg tablets (A A H Pharmaceuticals Ltd) | Bisoprolol fumarate | 1 |  |
| 7553 | 90711020 | Bisoprolol 5mg/5ml oral suspension | Bisoprolol fumarate | 1 |  |
| 39846 | 84559020 | Vivacor 5mg tablets (Lexon (UK) Ltd) | Bisoprolol fumarate | 1 |  |
| 50224 | 41307020 | Congescor 2.5mg tablets (Teva UK Ltd) | Bisoprolol fumarate | 1 |  |
| 55298 | 1097020 | Bisoprolol 10mg tablets (Sigma Pharmaceuticals Plc) | Bisoprolol fumarate | 1 |  |
| 57176 | 45145020 | Bisoprolol 10mg tablets (Accord Healthcare Ltd) | Bisoprolol fumarate | 1 |  |
| 57934 | 67815020 | Bisoprolol 5mg tablets (Sandoz Ltd) | Bisoprolol fumarate | 1 |  |
| 64538 | 77787020 | Bisoprolol 3.75mg tablets (Teva UK Ltd) | Bisoprolol fumarate | 1 |  |
| 599 | 68440020 | Bisoprolol 1.25mg tablets | Bisoprolol fumarate | 1 |  |
| 57626 | 19127020 | Bisoprolol 1.25mg/5ml oral solution | Bisoprolol fumarate | 1 |  |
| 1290 | 68439020 | Bisoprolol 10mg tablets | Bisoprolol fumarate | 1 |  |
| 59969 | 1090020 | Bisoprolol 5mg tablets (Almus Pharmaceuticals Ltd) | Bisoprolol fumarate | 1 |  |
| 59037 | 62942020 | Bisoprolol 5mg tablets (A A H Pharmaceuticals Ltd) | Bisoprolol fumarate | 1 |  |
| 43564 | 62726020 | Bisoprolol 5mg Tablet (PLIVA Pharma Ltd) | Bisoprolol fumarate | 1 |  |
| 4771 | 68375020 | Emcor LS 5mg tablets (Merck Serono Ltd) | Bisoprolol fumarate | 1 |  |
| 54479 | 1103020 | Bisoprolol 1.25mg tablets (Alliance Healthcare (Distribution) Ltd) | Bisoprolol fumarate | 1 |  |
| 21905 | 86502020 | Bipranix 10mg tablets (Ashbourne Pharmaceuticals Ltd) | Bisoprolol fumarate | 1 |  |
| 58982 | 38487020 | Bisoprolol 10mg tablets (Medreich Plc) | Bisoprolol fumarate | 1 |  |
| 58109 | 19125020 | Bisoprolol 1.25mg/5ml oral suspension | Bisoprolol fumarate | 1 |  |
| 7091 | 83179020 | Bisoprolol 3.75mg tablets | Bisoprolol fumarate | 1 |  |
| 52635 | 1082020 | Bisoprolol 5mg tablets (Alliance Healthcare (Distribution) Ltd) | Bisoprolol fumarate | 1 |  |
| 39646 | 96562020 | Bisoprolol 0.625mg/5ml oral solution | Bisoprolol Fumarate | 1 |  |
| 44000 | 98730020 | Bisoprolol 2.5mg/5ml oral suspension | Bisoprolol fumarate | 1 |  |
| 14030 | 83183020 | Cardicor 2.5mg tablets (Merck Serono Ltd) | Bisoprolol fumarate | 1 |  |
| 19858 | 83192020 | Cardicor 10mg tablets (Merck Serono Ltd) | Bisoprolol fumarate | 1 |  |
| 50403 | 73571020 | Bisoprolol 1.25mg Tablet (Teva UK Ltd) | Bisoprolol fumarate | 1 |  |
| 5713 | 83180020 | Bisoprolol 7.5mg tablets | Bisoprolol fumarate | 1 |  |
| 60761 | 15611021 | Bisoprolol 1.25mg tablets (Medreich Plc) | Bisoprolol fumarate | 1 |  |
| 60896 | 38486020 | Bisoprolol 5mg tablets (Medreich Plc) | Bisoprolol fumarate | 1 |  |
| 61115 | 19119020 | Bisoprolol 5mg/5ml oral solution | Bisoprolol fumarate | 1 |  |
| 56768 | 77972020 | Bisoprolol 2.5mg tablets (Niche Generics Ltd) | Bisoprolol fumarate | 1 |  |
| 64850 | 38489020 | Bisoprolol 2.5mg tablets (DE Pharmaceuticals) | Bisoprolol fumarate | 1 |  |
| 19853 | 83184020 | Cardicor 3.75mg tablets (Merck Serono Ltd) | Bisoprolol fumarate | 1 |  |
| 64784 | 63960020 | Bisoprolol 5mg tablets (Niche Generics Ltd) | Bisoprolol fumarate | 1 |  |
| 62361 | 1109020 | Bisoprolol 1.25mg tablets (Chanelle Medical UK Ltd) | Bisoprolol fumarate | 1 |  |
| 33839 | 62689020 | Bisoprolol 10mg tablets (Actavis UK Ltd) | Bisoprolol fumarate | 1 |  |
| 57023 | 1125020 | Bisoprolol 2.5mg tablets (Almus Pharmaceuticals Ltd) | Bisoprolol fumarate | 1 |  |
| 50300 | 41306020 | Congescor 1.25mg tablets (Teva UK Ltd) | Bisoprolol fumarate | 1 |  |
| 65821 | 35421020 | Bisoprolol 7.5mg/5ml oral suspension | Bisoprolol fumarate | 1 |  |
| 41591 | 62682020 | Bisoprolol 10mg tablets (Teva UK Ltd) | Bisoprolol fumarate | 1 |  |
| 51528 | 40504020 | Bisoprolol 1.25mg tablets (Actavis UK Ltd) | Bisoprolol fumarate | 1 |  |
| 53334 | 62943020 | Bisoprolol 10mg tablets (A A H Pharmaceuticals Ltd) | Bisoprolol fumarate | 1 |  |
| 56486 | 1094020 | Monocor 10mg tablets (Dowelhurst Ltd) | Bisoprolol fumarate | 1 |  |
| 21966 | 86500020 | Bipranix 5mg tablets (Ashbourne Pharmaceuticals Ltd) | Bisoprolol fumarate | 1 |  |
| 58973 | 63965020 | Bisoprolol 10mg tablets (Niche Generics Ltd) | Bisoprolol fumarate | 1 |  |
| 37837 | 73574020 | Bisoprolol 2.5mg Tablet (Teva UK Ltd) | Bisoprolol fumarate | 1 |  |
| 53916 | 78463020 | Bisoprolol 2.5mg tablets (Almus Pharmaceuticals Ltd) | Bisoprolol fumarate | 1 |  |
| 32630 | 77372020 | Vivacor 10mg tablets (Lexon (UK) Ltd) | Bisoprolol fumarate | 1 |  |
| 34963 | 62688020 | Bisoprolol 5mg tablets (Actavis UK Ltd) | Bisoprolol fumarate | 1 |  |
| 57578 | 1123020 | Cardicor 2.5mg tablets (Necessity Supplies Ltd) | Bisoprolol fumarate | 1 |  |
| 60502 | 38490020 | Bisoprolol 3.75mg tablets (DE Pharmaceuticals) | Bisoprolol fumarate | 1 |  |
| 56459 | 45146020 | Bisoprolol 2.5mg tablets (Accord Healthcare Ltd) | Bisoprolol fumarate | 1 |  |
| 65805 | 47115020 | Bisoprolol 1.25mg tablets (Waymade Healthcare Plc) | Bisoprolol fumarate | 1 |  |
| 32114 | 62676020 | Bisoprolol 5mg tablets (Mylan) | Bisoprolol fumarate | 1 |  |
| 5968 | 68354020 | Monocor 10mg tablets (Wyeth Pharmaceuticals) | Bisoprolol fumarate | 1 |  |
| 58455 | 40508020 | Bisoprolol 7.5mg tablets (Sandoz Ltd) | Bisoprolol fumarate | 1 |  |
| 32552 | 90619020 | Congescor 2.5mg tablets (Tillomed Laboratories Ltd) | Bisoprolol fumarate | 1 |  |
| 58511 | 40503020 | Bisoprolol 1.25mg tablets (Sandoz Ltd) | Bisoprolol fumarate | 1 |  |
| 67124 | 44188021 | Bisoprolol 10mg / Aspirin 75mg capsules | Bisoprolol fumarate/Aspirin | 1 | 0 |
| 17462 | 75023020 | Bisoprolol 10mg / Hydrochlorothiazide 6.25mg tablets | Bisoprolol fumarate/Hydrochlorothiazide | 0 | 1 |
| 17149 | 75020020 | Monozide 10 tablets (Wyeth Pharmaceuticals) | Bisoprolol fumarate/Hydrochlorothiazide | 0 | 1 |
| 29827 | 71512020 | Carteolol HCl 10mg tablets | Carteolol Hydrochloride | 1 |  |
| 28700 | 71407020 | Cartrol 10mg Tablet (Novartis Consumer Health UK Ltd) | Carteolol Hydrochloride | 1 |  |
| 34741 | 67913020 | Carvedilol 3.125mg tablets (IVAX Pharmaceuticals UK Ltd) | Carvedilol | 1 |  |
| 63422 | 47121020 | Carvedilol 12.5mg tablets (Waymade Healthcare Plc) | Carvedilol | 1 |  |
| 47107 | 567021 | Carvedilol 5mg/5ml oral suspension | Carvedilol | 1 |  |
| 34501 | 67708020 | Carvedilol 12.5mg tablets (Actavis UK Ltd) | Carvedilol | 1 |  |
| 18414 | 72241020 | Eucardic 12.5mg tablets (Roche Products Ltd) | Carvedilol | 1 |  |
| 46936 | 67784020 | Carvedilol 3.125mg tablets (A A H Pharmaceuticals Ltd) | Carvedilol | 1 |  |
| 49142 | 31922020 | Carvedilol 3.125mg/5ml oral suspension | Carvedilol | 1 |  |
| 7049 | 72237020 | Carvedilol 25mg tablets | Carvedilol | 1 |  |
| 46935 | 67701020 | Carvedilol 3.125mg tablets (Actavis UK Ltd) | Carvedilol | 1 |  |
| 4410 | 83758020 | Carvedilol 6.25mg tablets | Carvedilol | 1 |  |
| 54106 | 35298020 | Carvedilol 1.5mg/5ml oral suspension | Carvedilol | 1 |  |
| 14146 | 83760020 | Eucardic 6.25mg tablets (Roche Products Ltd) | Carvedilol | 1 |  |
| 67661 | 1216020 | Carvedilol 6.25mg tablets (Sigma Pharmaceuticals Plc) | Carvedilol | 1 |  |
| 14117 | 72243020 | Eucardic 3.125mg tablets (Roche Products Ltd) | Carvedilol | 1 |  |
| 19202 | 67720020 | Carvedilol 6.25mg tablets (Teva UK Ltd) | Carvedilol | 1 |  |
| 19437 | 72242020 | Eucardic 25mg tablets (Roche Products Ltd) | Carvedilol | 1 |  |
| 34740 | 67705020 | Carvedilol 6.25mg tablets (Actavis UK Ltd) | Carvedilol | 1 |  |
| 59549 | 19333020 | Carvedilol 5mg/5ml oral suspension | Carvedilol | 1 |  |
| 61663 | 67717020 | Carvedilol 3.125mg tablets (Teva UK Ltd) | Carvedilol | 1 |  |
| 33374 | 69739020 | Carvedilol 12.5mg tablets (Genus Pharmaceuticals Ltd) | Carvedilol | 1 |  |
| 817 | 72238020 | Carvedilol 3.125mg tablets | Carvedilol | 1 |  |
| 2629 | 72236020 | Carvedilol 12.5mg tablets | Carvedilol | 1 |  |
| 42795 | 61054020 | Celiprolol 200mg tablets (Mylan) | Celiprolol hydrochloride | 1 |  |
| 8262 | 68837020 | Celiprolol 200mg tablets | Celiprolol hydrochloride | 1 |  |
| 35940 | 91237020 | Celectol 400mg tablets (Zentiva) | Celiprolol hydrochloride | 1 |  |
| 56485 | 1155020 | Celectol 200mg tablets (Waymade Healthcare Plc) | Celiprolol hydrochloride | 1 |  |
| 41740 | 63783020 | Celiprolol 200mg tablets (Teva UK Ltd) | Celiprolol hydrochloride | 1 |  |
| 57573 | 1153020 | Celectol 200mg tablets (Dowelhurst Ltd) | Celiprolol hydrochloride | 1 |  |
| 4265 | 68965020 | Celectol 200mg Tablet (Pantheon Healthcare Ltd) | Celiprolol hydrochloride | 1 |  |
| 7974 | 68838020 | Celiprolol 400mg tablets | Celiprolol hydrochloride | 1 |  |
| 16776 | 68966020 | Celectol 400mg Tablet (Pantheon Healthcare Ltd) | Celiprolol hydrochloride | 1 |  |
| 35054 | 91235020 | Celectol 200mg tablets (Zentiva) | Celiprolol hydrochloride | 1 |  |
| 67292 | 1157020 | Celectol 200mg tablets (Sigma Pharmaceuticals Plc) | Celiprolol hydrochloride | 1 |  |
| 581 | 67597020 | Atenolol 50mg with Chlortalidone 12.5mg tablets | Chlortalidone/Atenolol | 0 | 1 |
| 16786 | 61276020 | Chlortalidone 25mg with Atenolol 100mg tablets | Chlortalidone/Atenolol | 0 | 1 |
| 19055 | 61275020 | Chlortalidone 12.5mg with Atenolol 50mg tablets | Chlortalidone/Atenolol | 0 | 1 |
| 1788 | 67598020 | Atenolol 100mg with Chlortalidone 25mg tablets | Chlortalidone/Atenolol | 0 | 1 |
| 14057 | 65806020 | Pindolol 10mg / Clopamide 5mg tablets | Clopamide/Pindolol | 0 | 1 |
| 9143 | 52285020 | Viskaldix tablets (AMCo) | Clopamide/Pindolol | 0 | 1 |
| 35778 | 58483020 | Labrocol 100mg Tablet (Lagap) | Labetalol hydrochloride | 1 |  |
| 40240 | 49924020 | Labetalol 400mg tablets (A A H Pharmaceuticals Ltd) | Labetalol hydrochloride | 1 |  |
| 34177 | 49922020 | Labetalol 100mg tablets (A A H Pharmaceuticals Ltd) | Labetalol hydrochloride | 1 |  |
| 1295 | 68333020 | Labetalol 400mg tablets | Labetalol hydrochloride | 1 |  |
| 22793 | 58484020 | Labrocol 200mg Tablet (Lagap) | Labetalol hydrochloride | 1 |  |
| 63736 | 14465021 | Labetalol 100mg tablets (Waymade Healthcare Plc) | Labetalol hydrochloride | 1 |  |
| 62638 | 57012020 | Labetalol 100mg tablets (Actavis UK Ltd) | Labetalol hydrochloride | 1 |  |
| 1597 | 63899020 | Labetalol 100mg tablets | Labetalol hydrochloride | 1 |  |
| 41827 | 60515020 | Labetalol 100mg tablets (Mylan) | Labetalol hydrochloride | 1 |  |
| 47674 | 49918020 | Labetalol 200mg Tablet (C P Pharmaceuticals Ltd) | Labetalol hydrochloride | 1 |  |
| 4725 | 68332020 | Labetalol 50mg tablets | Labetalol hydrochloride | 1 |  |
| 45250 | 66745020 | Labetalol 400mg tablets (Sandoz Ltd) | Labetalol hydrochloride | 1 |  |
| 44083 | 57013020 | Labetalol 200mg tablets (Actavis UK Ltd) | Labetalol hydrochloride | 1 |  |
| 47673 | 54575020 | Labetalol 400mg Tablet (Approved Prescription Services Ltd) | Labetalol hydrochloride | 1 |  |
| 8707 | 54881020 | Trandate 200mg tablets (Focus Pharmaceuticals Ltd) | Labetalol hydrochloride | 1 |  |
| 2775 | 63900020 | Labetalol 200mg tablets | Labetalol hydrochloride | 1 |  |
| 34171 | 49917020 | Labetalol 100mg Tablet (C P Pharmaceuticals Ltd) | Labetalol hydrochloride | 1 |  |
| 8807 | 52545020 | Trandate 400mg tablets (Focus Pharmaceuticals Ltd) | Labetalol hydrochloride | 1 |  |
| 30770 | 49923020 | Labetalol 200mg tablets (A A H Pharmaceuticals Ltd) | Labetalol hydrochloride | 1 |  |
| 9016 | 54880020 | Trandate 100mg tablets (Focus Pharmaceuticals Ltd) | Labetalol hydrochloride | 1 |  |
| 34188 | 51424020 | Labetalol 200mg Tablet (Celltech Pharma Europe Ltd) | Labetalol hydrochloride | 1 |  |
| 16645 | 58485020 | Labrocol 400mg Tablet (Lagap) | Labetalol hydrochloride | 1 |  |
| 9273 | 54879020 | Trandate 50mg tablets (Focus Pharmaceuticals Ltd) | Labetalol hydrochloride | 1 |  |
| 29998 | 69479020 | Metoros 190mg Tablet (Novartis Pharmaceuticals UK Ltd) | Metoprolol | 1 |  |
| 27719 | 69482020 | Metoros ls 95mg Tablet (Geigy Pharmaceuticals) | Metoprolol | 1 |  |
| 40167 | 54126020 | Metoprolol 100mg tablets (IVAX Pharmaceuticals UK Ltd) | Metoprolol tartrate | 1 |  |
| 34509 | 60551020 | Metoprolol 100mg tablets (Mylan) | Metoprolol tartrate | 1 |  |
| 29762 | 63850020 | Mepranix 50mg Tablet (Ashbourne Pharmaceuticals Ltd) | Metoprolol tartrate | 1 |  |
| 34094 | 50072020 | Metoprolol 50mg tablets (A A H Pharmaceuticals Ltd) | Metoprolol tartrate | 1 |  |
| 70116 | 20537020 | Metoprolol 50mg/5ml oral solution | Metoprolol tartrate | 1 |  |
| 3474 | 53660020 | Betaloc-SA 200mg tablets (AstraZeneca UK Ltd) | Metoprolol tartrate | 1 |  |
| 20082 | 50243020 | Lopresor SR 200mg tablets (Recordati Pharmaceuticals Ltd) | Metoprolol tartrate | 1 |  |
| 8068 | 64670020 | Metoprolol 200mg modified-release tablets | Metoprolol tartrate | 1 |  |
| 11793 | 67160020 | Metoprolol 50mg/5ml oral suspension | Metoprolol tartrate | 1 |  |
| 10429 | 50246020 | Lopresor 50mg Tablet (Novartis Pharmaceuticals UK Ltd) | Metoprolol tartrate | 1 |  |
| 34854 | 57038020 | Metoprolol 100mg tablets (Actavis UK Ltd) | Metoprolol tartrate | 1 |  |
| 8071 | 48396020 | Betaloc 50mg tablets (AstraZeneca UK Ltd) | Metoprolol tartrate | 1 |  |
| 34925 | 65979020 | Metoprolol 50mg tablets (Sandoz Ltd) | Metoprolol tartrate | 1 |  |
| 63724 | 47106020 | Metoprolol 100mg tablets (Waymade Healthcare Plc) | Metoprolol tartrate | 1 |  |
| 66670 | 717020 | Metoprolol 100mg tablets (Alliance Healthcare (Distribution) Ltd) | Metoprolol tartrate | 1 |  |
| 739 | 64666020 | Metoprolol 50mg tablets | Metoprolol tartrate | 1 |  |
| 3344 | 48397020 | Betaloc 100mg tablets (AstraZeneca UK Ltd) | Metoprolol tartrate | 1 |  |
| 34430 | 57037020 | Metoprolol 50mg tablets (Actavis UK Ltd) | Metoprolol tartrate | 1 |  |
| 47536 | 770021 | Metoprolol tartrate 12.5mg/5ml Oral suspension | Metoprolol Tartrate | 1 |  |
| 55979 | 20535020 | Metoprolol 25mg/5ml oral suspension | Metoprolol tartrate | 1 |  |
| 46614 | 195021 | Lopresor 50mg tablets (Recordati Pharmaceuticals Ltd) | Metoprolol tartrate | 1 |  |
| 34584 | 54125020 | Metoprolol 50mg tablets (IVAX Pharmaceuticals UK Ltd) | Metoprolol tartrate | 1 |  |
| 45289 | 91796020 | Metoprolol tartrate Oral solution | Metoprolol Tartrate | 1 |  |
| 51447 | 20527020 | Metoprolol 12.5mg/5ml oral suspension | Metoprolol tartrate | 1 |  |
| 753 | 64667020 | Metoprolol 100mg tablets | Metoprolol tartrate | 1 |  |
| 34125 | 50073020 | Metoprolol 100mg tablets (A A H Pharmaceuticals Ltd) | Metoprolol tartrate | 1 |  |
| 68881 | 98589020 | Metoprolol 12.5mg capsules | Metoprolol tartrate | 1 |  |
| 30400 | 63851020 | Mepranix 100mg Tablet (Ashbourne Pharmaceuticals Ltd) | Metoprolol tartrate | 1 |  |
| 32836 | 60550020 | Metoprolol 50mg tablets (Mylan) | Metoprolol tartrate | 1 |  |
| 71098 | 45140020 | Metoprolol 50mg tablets (Accord Healthcare Ltd) | Metoprolol tartrate | 1 |  |
| 34407 | 56767020 | Metoprolol 50mg tablets (Teva UK Ltd) | Metoprolol tartrate | 1 |  |
| 34890 | 53461020 | Metoprolol 50mg Tablet (Berk Pharmaceuticals Ltd) | Metoprolol tartrate | 1 |  |
| 34092 | 56768020 | Metoprolol 100mg tablets (Teva UK Ltd) | Metoprolol tartrate | 1 |  |
| 46740 | 197021 | Lopresor 100mg tablets (Recordati Pharmaceuticals Ltd) | Metoprolol tartrate | 1 |  |
| 65227 | 20525020 | Metoprolol 12.5mg/5ml oral solution | Metoprolol tartrate | 1 |  |
| 13499 | 50247020 | Lopresor 100mg Tablet (Novartis Pharmaceuticals UK Ltd) | Metoprolol tartrate | 1 |  |
| 57240 | 20539020 | Metoprolol 50mg/5ml oral suspension (Special Order) | Metoprolol tartrate | 1 |  |
| 8147 | 50252020 | Lopresoretic Tablet (Novartis Pharmaceuticals UK Ltd) | Metoprolol Tartrate/Chlortalidone | 0 | 1 |
| 15488 | 68972020 | Metoprolol tartrate with chlortalidone Tablet | Metoprolol Tartrate/Chlortalidone | 0 | 1 |
| 7066 | 68976020 | Metoprolol 100mg / Hydrochlorothiazide 12.5mg tablets | Metoprolol tartrate/Hydrochlorothiazide | 0 | 1 |
| 33659 | 68979020 | Hydrochlorothiazide with metoprolol tartrate 25mg with 200mg Modified-release tablet | Metoprolol Tartrate/Hydrochlorothiazide | 0 | 1 |
| 29427 | 68980020 | Hydrochlorothiazide with metoprolol tartrate 12.5mg with 100mg tablet | Metoprolol Tartrate/Hydrochlorothiazide | 0 | 1 |
| 20093 | 68975020 | Metoprolol 200mg modified-release / Hydrochlorothiazide 25mg tablets | Metoprolol tartrate/Hydrochlorothiazide | 0 | 1 |
| 18287 | 53671020 | Co-Betaloc SA tablets (Pfizer Ltd) | Metoprolol tartrate/Hydrochlorothiazide | 0 | 1 |
| 10627 | 48722020 | Co-Betaloc tablets (Pfizer Ltd) | Metoprolol tartrate/Hydrochlorothiazide | 0 | 1 |
| 10716 | 54539020 | Corgard 80mg tablets (Sanofi) | Nadolol | 1 |  |
| 13415 | 54538020 | Corgard 40mg tablets (Sanofi-Synthelabo Ltd) | Nadolol | 1 |  |
| 8935 | 64895020 | Nadolol 40mg tablets | Nadolol | 1 |  |
| 66779 | 36922020 | Nadolol 30mg/5ml oral suspension | Nadolol | 1 |  |
| 66464 | 31704020 | Nadolol 40mg/5ml oral solution | Nadolol | 1 |  |
| 2499 | 64896020 | Nadolol 80mg tablets | Nadolol | 1 |  |
| 67424 | 36157020 | Nadolol 80mg/5ml oral suspension | Nadolol | 1 |  |
| 11338 | 67627020 | Bendroflumethiazide 5mg with Nadolol 40mg tablets | Nadolol/Bendroflumethiazide | 0 | 1 |
| 23134 | 64899020 | Nadolol 40mg / Bendroflumethiazide 5mg tablets | Nadolol/Bendroflumethiazide | 0 | 1 |
| 14438 | 54543020 | Corgaretic 80mg tablets (Sanofi-Synthelabo Ltd) | Nadolol/Bendroflumethiazide | 0 | 1 |
| 27946 | 64900020 | Nadolol 80mg / Bendroflumethiazide 5mg tablets | Nadolol/Bendroflumethiazide | 0 | 1 |
| 69334 | 67628020 | Bendroflumethiazide 5mg with Nadolol 80mg tablets | Nadolol/Bendroflumethiazide | 0 | 1 |
| 5330 | 48767020 | Corgaretic 40mg tablets (Sanofi-Synthelabo Ltd) | Nadolol/Bendroflumethiazide | 0 | 1 |
| 59961 | 21461021 | Nebivolol 10mg tablets | Nebivolol hydrochloride | 1 |  |
| 7528 | 80460020 | Nebilet 5mg tablets (A. Menarini Farmaceutica Internazionale SRL) | Nebivolol hydrochloride | 1 |  |
| 47300 | 76819020 | Nebivolol 2.5mg tablets (Glenmark Pharmaceuticals Europe Ltd) | Nebivolol hydrochloride | 1 |  |
| 40761 | 97367020 | Nebivolol 2.5mg tablets | Nebivolol hydrochloride | 1 |  |
| 751 | 80458020 | Nebivolol 5mg tablets | Nebivolol hydrochloride | 1 |  |
| 67595 | 25449021 | Nebivolol 5mg tablets (Almus Pharmaceuticals Ltd) | Nebivolol hydrochloride | 1 |  |
| 64703 | 1227020 | Nebivolol 5mg tablets (Glenmark Pharmaceuticals Europe Ltd) | Nebivolol hydrochloride | 1 |  |
| 71032 | 40509020 | Nebivolol 5mg tablets (Sandoz Ltd) | Nebivolol hydrochloride | 1 |  |
| 66559 | 1220020 | Nebilet 5mg tablets (Waymade Healthcare Plc) | Nebivolol hydrochloride | 1 |  |
| 68677 | 77740020 | Nebivolol 5mg tablets (Actavis UK Ltd) | Nebivolol hydrochloride | 1 |  |
| 69115 | 75760020 | Nebivolol 5mg tablets (A A H Pharmaceuticals Ltd) | Nebivolol hydrochloride | 1 |  |
| 44808 | 77127020 | Nebivolol 2.5mg tablets (A A H Pharmaceuticals Ltd) | Nebivolol hydrochloride | 1 |  |
| 54487 | 44459020 | Nebivolol 2.5mg tablets (Sigma Pharmaceuticals Plc) | Nebivolol hydrochloride | 1 |  |
| 15117 | 68850020 | Nifedipine with atenolol 20mg + 50mg Capsule | Nifedipine/Atenolol | 0 | 1 |
| 2361 | 54886020 | Trasicor 80mg Tablet (Novartis Pharmaceuticals UK Ltd) | Oxprenolol hydrochloride | 1 |  |
| 3748 | 59365020 | Oxprenolol 160mg Tablet | Oxprenolol Hydrochloride | 1 |  |
| 7474 | 54884020 | Trasicor 20mg Tablet (Novartis Pharmaceuticals UK Ltd) | Oxprenolol hydrochloride | 1 |  |
| 27357 | 49156020 | Oxprenolol 40mg Tablet (Actavis UK Ltd) | Oxprenolol hydrochloride | 1 |  |
| 29180 | 90895020 | Trasicor 80mg tablets (Amdipharm Plc) | Oxprenolol hydrochloride | 1 |  |
| 35062 | 90891020 | Trasicor 20mg tablets (Amdipharm Plc) | Oxprenolol hydrochloride | 1 |  |
| 29230 | 59496020 | Slow-pren 160mg Tablet (IVAX Pharmaceuticals UK Ltd) | Oxprenolol hydrochloride | 1 |  |
| 10777 | 54889020 | Trasicor 160mg Tablet (Novartis Pharmaceuticals UK Ltd) | Oxprenolol Hydrochloride | 1 |  |
| 21885 | 74896020 | Oxyprenix SR 160mg tablets | Oxprenolol Hydrochloride | 1 |  |
| 4025 | 51584020 | Slow-Trasicor 160mg tablets (AMCo) | Oxprenolol hydrochloride | 1 |  |
| 24094 | 90893020 | Trasicor 40mg tablets (Amdipharm Plc) | Oxprenolol hydrochloride | 1 |  |
| 1334 | 65274020 | Oxprenolol 160mg modified-release tablets | Oxprenolol hydrochloride | 1 |  |
| 3516 | 59360020 | Oxprenolol 20mg tablets | Oxprenolol hydrochloride | 1 |  |
| 2780 | 59362020 | Oxprenolol 80mg tablets | Oxprenolol hydrochloride | 1 |  |
| 25644 | 57667020 | Apsolox 80mg Tablet (Approved Prescription Services Ltd) | Oxprenolol hydrochloride | 1 |  |
| 33569 | 49177020 | Oxprenolol sr 160mg Modified-release tablet (Hillcross Pharmaceuticals Ltd) | Oxprenolol hydrochloride | 1 |  |
| 8290 | 54885020 | Trasicor 40mg Tablet (Novartis Pharmaceuticals UK Ltd) | Oxprenolol hydrochloride | 1 |  |
| 1333 | 59361020 | Oxprenolol 40mg tablets | Oxprenolol hydrochloride | 1 |  |
| 8673 | 73918020 | Oxprenolol with cyclopenthiazide 160mg+0.25mg Modified-release tablet | Oxprenolol Hydrochloride/Cyclopenthiazide | 0 | 1 |
| 13871 | 73921020 | Co-prenozide 160mg/0.25mg modified-release tablets | Oxprenolol hydrochloride/Cyclopenthiazide | 0 | 1 |
| 52145 | 61691020 | Cyclopenthiazide 0.25mg with oxprenolol 160mg modified-release tablets | Oxprenolol Hydrochloride/Cyclopenthiazide | 0 | 1 |
| 4429 | 51994020 | Trasidrex modified-release tablets (Mercury Pharma Group Ltd) | Oxprenolol hydrochloride/Cyclopenthiazide | 0 | 1 |
| 26529 | 62151020 | Furosemide with penbutolol Tablet | Penbutolol/Furosemide | 0 | 1 |
| 24832 | 52999020 | Lasipressin Tablet (Hoechst UK Ltd) | Penbutolol/Furosemide | 0 | 1 |
| 4588 | 52291020 | Visken 5mg Tablet (Sovereign Medical Ltd) | Pindolol | 1 |  |
| 20012 | 52292020 | Visken 15mg Tablet (Sovereign Medical Ltd) | Pindolol | 1 |  |
| 5284 | 65802020 | Pindolol 5mg tablets | Pindolol | 1 |  |
| 55853 | 60484020 | Pindolol 15mg Tablet (Hillcross Pharmaceuticals Ltd) | Pindolol | 1 |  |
| 14673 | 65803020 | Pindolol 15mg tablets | Pindolol | 1 |  |
| 35695 | 78143020 | Visken 5mg tablets (AMCo) | Pindolol | 1 |  |
| 32787 | 78135020 | Visken 15mg tablets (AMCo) | Pindolol | 1 |  |
| 25462 | 61503020 | Clopamide 5mg with Pindolol 10mg tablets | Pindolol/Clopamide | 0 | 1 |
| 5478 | 59376020 | Propranolol 10mg/5ml oral solution sugar free | Propranolol hydrochloride | 1 |  |
| 58491 | 924020 | Propranolol 40mg tablets (Alliance Healthcare (Distribution) Ltd) | Propranolol hydrochloride | 1 |  |
| 34378 | 50435020 | Propranolol 10mg tablets (A A H Pharmaceuticals Ltd) | Propranolol hydrochloride | 1 |  |
| 55416 | 71580020 | Propranolol 40mg tablets (Almus Pharmaceuticals Ltd) | Propranolol hydrochloride | 1 |  |
| 64160 | 15600021 | Propranolol 5mg/5ml oral solution sugar free (AM Distributions (Yorkshire) Ltd) | Propranolol hydrochloride | 1 |  |
| 58297 | 918020 | Propranolol 10mg tablets (Kent Pharmaceuticals Ltd) | Propranolol hydrochloride | 1 |  |
| 31776 | 63160020 | Propranolol 40mg tablets (Mylan) | Propranolol hydrochloride | 1 |  |
| 45877 | 99275020 | Beta-Prograne 160mg modified-release capsules (Teva UK Ltd) | Propranolol hydrochloride | 1 |  |
| 1048 | 54495020 | Inderal 80mg tablets (AstraZeneca UK Ltd) | Propranolol hydrochloride | 1 |  |
| 34783 | 50422020 | Propranolol 10mg tablets (Actavis UK Ltd) | Propranolol hydrochloride | 1 |  |
| 48682 | 183020 | Propranolol 50mg/5ml oral solution sugar free | Propranolol hydrochloride | 1 |  |
| 3167 | 59374020 | Propranolol 160mg tablets | Propranolol hydrochloride | 1 |  |
| 70680 | 34208020 | Propranolol 6mg/5ml oral suspension | Propranolol hydrochloride | 1 |  |
| 71173 | 57246021 | Propranolol 80mg modified-release capsules (Mawdsley-Brooks & Company Ltd) | Propranolol hydrochloride | 1 |  |
| 14808 | 55786020 | Bedranol SR 80mg capsules (Sandoz Ltd) | Propranolol hydrochloride | 1 |  |
| 9185 | 75255020 | Propranolol 80mg/5ml oral solution | Propranolol Hydrochloride | 1 |  |
| 71150 | 34202020 | Propranolol 5mg/5ml oral solution | Propranolol hydrochloride | 1 |  |
| 53177 | 92021020 | Propranolol oral solution | Propranolol Hydrochloride | 1 |  |
| 34208 | 51777020 | Propranolol SR 160mg Modified-release capsule (C P Pharmaceuticals Ltd) | Propranolol hydrochloride | 1 |  |
| 29763 | 63927020 | Propanix 160mg Tablet (Ashbourne Pharmaceuticals Ltd) | Propranolol hydrochloride | 1 |  |
| 36603 | 50847020 | Propranolol SR 160mg Modified-release capsule (Hillcross Pharmaceuticals Ltd) | Propranolol hydrochloride | 1 |  |
| 45297 | 64022020 | Propranolol 40mg tablets (IVAX Pharmaceuticals UK Ltd) | Propranolol hydrochloride | 1 |  |
| 34214 | 50424020 | Propranolol 160mg tablets (Actavis UK Ltd) | Propranolol hydrochloride | 1 |  |
| 31833 | 59468020 | Angilol 80mg Tablet (DDSA Pharmaceuticals Ltd) | Propranolol hydrochloride | 1 |  |
| 33602 | 85990020 | Slo-Pro 160mg capsules (Mylan) | Propranolol hydrochloride | 1 |  |
| 41555 | 50436020 | Propranolol 40mg tablets (A A H Pharmaceuticals Ltd) | Propranolol hydrochloride | 1 |  |
| 25359 | 79243020 | Rapranol SR 160mg capsules (Ranbaxy (UK) Ltd) | Propranolol hydrochloride | 1 |  |
| 297 | 59368020 | Propranolol 10mg tablets | Propranolol hydrochloride | 1 |  |
| 40241 | 56193020 | Propranolol LA 160mg Capsule (Approved Prescription Services Ltd) | Propranolol hydrochloride | 1 |  |
| 33644 | 50437020 | Propranolol 80mg tablets (A A H Pharmaceuticals Ltd) | Propranolol hydrochloride | 1 |  |
| 33836 | 57662020 | Apsolol 160mg Tablet (Approved Prescription Services Ltd) | Propranolol hydrochloride | 1 |  |
| 56173 | 955020 | Half Beta-Prograne 80mg modified-release capsules (Actavis UK Ltd) | Propranolol hydrochloride | 1 |  |
| 28128 | 54244020 | Propranolol 80mg Modified-release capsule (Actavis UK Ltd) | Propranolol hydrochloride | 1 |  |
| 1050 | 54494020 | Inderal 40mg tablets (AstraZeneca UK Ltd) | Propranolol hydrochloride | 1 |  |
| 33376 | 78494020 | Probeta LA 160mg Capsule (Trinity Pharmaceuticals Ltd) | Propranolol hydrochloride | 1 |  |
| 55949 | 20600020 | Propranolol 40mg/5ml oral solution | Propranolol hydrochloride | 1 |  |
| 23326 | 71607020 | Betadur cr 160mg Modified-release capsule (Monmouth Pharmaceuticals Ltd) | Propranolol hydrochloride | 1 |  |
| 59597 | 75862020 | Propranolol 160mg modified-release capsules (A A H Pharmaceuticals Ltd) | Propranolol hydrochloride | 1 |  |
| 3005 | 49929020 | Inderal LA 160mg capsules (AstraZeneca UK Ltd) | Propranolol hydrochloride | 1 |  |
| 28996 | 87099020 | Bedranol SR 160mg capsules (Sandoz Ltd) | Propranolol hydrochloride | 1 |  |
| 1448 | 66308020 | Propranolol 160mg modified-release capsules | Propranolol hydrochloride | 1 |  |
| 3827 | 64050020 | Propanix 40mg Tablet (Ashbourne Pharmaceuticals Ltd) | Propranolol hydrochloride | 1 |  |
| 57567 | 34143020 | Propranolol 10mg/5ml oral suspension | Propranolol hydrochloride | 1 |  |
| 57063 | 957020 | Bedranol SR 80mg capsules (Almus Pharmaceuticals Ltd) | Propranolol hydrochloride | 1 |  |
| 45765 | 96602020 | Syprol 40mg/5ml oral solution (Rosemont Pharmaceuticals Ltd) | Propranolol hydrochloride | 1 |  |
| 21839 | 54823020 | Berkolol 80mg Tablet (Berk Pharmaceuticals Ltd) | Propranolol hydrochloride | 1 |  |
| 31214 | 63161020 | Propranolol 80mg tablets (Mylan) | Propranolol hydrochloride | 1 |  |
| 8978 | 63928020 | Propanix 160mg Modified-release capsule (Ashbourne Pharmaceuticals Ltd) | Propranolol hydrochloride | 1 |  |
| 54623 | 99279020 | Beta-Prograne 160mg modified-release capsules (Actavis UK Ltd) | Propranolol hydrochloride | 1 |  |
| 27700 | 50423020 | Propranolol 40mg tablets (Actavis UK Ltd) | Propranolol hydrochloride | 1 |  |
| 45494 | 71577020 | Propranolol 10mg tablets (Almus Pharmaceuticals Ltd) | Propranolol hydrochloride | 1 |  |
| 32162 | 59465020 | Propranolol 80mg Modified-release capsule (Lagap) | Propranolol hydrochloride | 1 |  |
| 45343 | 51778020 | Propranolol SR 80mg Modified-release capsule (C P Pharmaceuticals Ltd) | Propranolol hydrochloride | 1 |  |
| 3087 | 75253020 | Propranolol 40mg/5ml oral solution sugar free | Propranolol hydrochloride | 1 |  |
| 54297 | 34198020 | Propranolol 50mg/5ml oral solution | Propranolol hydrochloride | 1 |  |
| 769 | 66307020 | Propranolol 80mg modified-release capsules | Propranolol hydrochloride | 1 |  |
| 707 | 59369020 | Propranolol 40mg tablets | Propranolol hydrochloride | 1 |  |
| 47543 | 627021 | Half Beta-Prograne 80mg modified-release capsules (Actavis UK Ltd) | Propranolol hydrochloride | 1 |  |
| 55849 | 63164020 | Propranolol 160mg tablets (Mylan) | Propranolol hydrochloride | 1 |  |
| 21838 | 64051020 | Propanix 80mg Tablet (Ashbourne Pharmaceuticals Ltd) | Propranolol hydrochloride | 1 |  |
| 34949 | 54245020 | Propranolol 160mg Modified-release capsule (Actavis UK Ltd) | Propranolol hydrochloride | 1 |  |
| 59415 | 20140021 | Propranolol 40mg tablets (Accord Healthcare Ltd) | Propranolol hydrochloride | 1 |  |
| 17082 | 79593020 | Syprol 5mg/5ml oral solution (Rosemont Pharmaceuticals Ltd) | Propranolol hydrochloride | 1 |  |
| 26895 | 79594020 | Syprol 10mg/5ml oral solution (Rosemont Pharmaceuticals Ltd) | Propranolol hydrochloride | 1 |  |
| 15619 | 57243020 | Half-betadur cr 80mg Capsule (Monmouth Pharmaceuticals Ltd) | Propranolol hydrochloride | 1 |  |
| 25367 | 84837020 | Rapranol SR 80mg capsules (Ranbaxy (UK) Ltd) | Propranolol hydrochloride | 1 |  |
| 42152 | 79595020 | Syprol 50mg/5ml oral solution (Rosemont Pharmaceuticals Ltd) | Propranolol hydrochloride | 1 |  |
| 46363 | 99271020 | Half Beta-Prograne 80mg modified-release capsules (Teva UK Ltd) | Propranolol hydrochloride | 1 |  |
| 58407 | 53353020 | Propranolol 80mg tablets (Teva UK Ltd) | Propranolol hydrochloride | 1 |  |
| 39233 | 68806020 | Propranolol 80mg modified-release capsules (Teva UK Ltd) | Propranolol hydrochloride | 1 |  |
| 65986 | 920020 | Propranolol 10mg tablets (Alliance Healthcare (Distribution) Ltd) | Propranolol hydrochloride | 1 |  |
| 38433 | 55581020 | Propranolol 50mg/5ml Oral solution (Rosemont Pharmaceuticals Ltd) | Propranolol Hydrochloride | 1 |  |
| 27964 | 57658020 | Apsolol 40mg Tablet (Approved Prescription Services Ltd) | Propranolol hydrochloride | 1 |  |
| 22208 | 84849020 | Half propanix la 80mg Modified-release capsule (Ashbourne Pharmaceuticals Ltd) | Propranolol hydrochloride | 1 |  |
| 66555 | 922020 | Propranolol 10mg tablets (Boston Healthcare Ltd) | Propranolol hydrochloride | 1 |  |
| 60565 | 70592020 | Propranolol 40mg tablets (Ranbaxy (UK) Ltd) | Propranolol hydrochloride | 1 |  |
| 34884 | 66703020 | Propranolol 160mg Modified-release capsule (Sandoz Ltd) | Propranolol hydrochloride | 1 |  |
| 47907 | 538021 | Bedranol SR 160mg capsules (Almus Pharmaceuticals Ltd) | Propranolol hydrochloride | 1 |  |
| 36576 | 63159020 | Propranolol 10mg tablets (Mylan) | Propranolol hydrochloride | 1 |  |
| 21866 | 54822020 | Berkolol 40mg Tablet (Berk Pharmaceuticals Ltd) | Propranolol hydrochloride | 1 |  |
| 8331 | 54498020 | Inderal 160mg Tablet (AstraZeneca UK Ltd) | Propranolol hydrochloride | 1 |  |
| 34867 | 56034020 | Propranolol 80mg Capsule (IVAX Pharmaceuticals UK Ltd) | Propranolol hydrochloride | 1 |  |
| 69661 | 34181020 | Propranolol 3mg/5ml oral solution | Propranolol hydrochloride | 1 |  |
| 34804 | 53351020 | Propranolol 10mg tablets (Teva UK Ltd) | Propranolol hydrochloride | 1 |  |
| 2414 | 54493020 | Inderal 10mg tablets (AstraZeneca UK Ltd) | Propranolol hydrochloride | 1 |  |
| 26229 | 56515020 | Beta-Prograne 160mg modified-release capsules (Tillomed Laboratories Ltd) | Propranolol hydrochloride | 1 |  |
| 220 | 59375020 | Propranolol 5mg/5ml oral solution | Propranolol Hydrochloride | 1 |  |
| 940 | 59370020 | Propranolol 80mg tablets | Propranolol hydrochloride | 1 |  |
| 14552 | 64049020 | Propanix 10mg Tablet (Ashbourne Pharmaceuticals Ltd) | Propranolol hydrochloride | 1 |  |
| 28048 | 59466020 | Angilol 10mg Tablet (DDSA Pharmaceuticals Ltd) | Propranolol hydrochloride | 1 |  |
| 61727 | 20139021 | Propranolol 10mg tablets (Accord Healthcare Ltd) | Propranolol hydrochloride | 1 |  |
| 11711 | 75254020 | Propranolol 50mg/5ml oral solution | Propranolol Hydrochloride | 1 |  |
| 23587 | 66314020 | Sloprolol 160mg Capsule (C P Pharmaceuticals Ltd) | Propranolol hydrochloride | 1 |  |
| 52777 | 925020 | Propranolol 40mg tablets (Kent Pharmaceuticals Ltd) | Propranolol hydrochloride | 1 |  |
| 62711 | 20143021 | Propranolol 80mg modified-release capsules (Waymade Healthcare Plc) | Propranolol hydrochloride | 1 |  |
| 26255 | 78284020 | Lopranol la 160mg Capsule (Opus Pharmaceuticals Ltd) | Propranolol hydrochloride | 1 |  |
| 60934 | 948020 | Propranolol 80mg modified-release capsules (Kent Pharmaceuticals Ltd) | Propranolol hydrochloride | 1 |  |
| 65435 | 47095020 | Propranolol 10mg tablets (Waymade Healthcare Plc) | Propranolol hydrochloride | 1 |  |
| 34868 | 53352020 | Propranolol 40mg tablets (Teva UK Ltd) | Propranolol hydrochloride | 1 |  |
| 47833 | 536021 | Bedranol SR 80mg capsules (Almus Pharmaceuticals Ltd) | Propranolol hydrochloride | 1 |  |
| 35938 | 64558020 | Propranolol 80mg modified-release capsules (A A H Pharmaceuticals Ltd) | Propranolol hydrochloride | 1 |  |
| 70681 | 34163020 | Propranolol 3mg/5ml oral suspension | Propranolol hydrochloride | 1 |  |
| 56764 | 47096020 | Propranolol 40mg tablets (Waymade Healthcare Plc) | Propranolol hydrochloride | 1 |  |
| 57342 | 928020 | Propranolol 40mg tablets (Phoenix Healthcare Distribution Ltd) | Propranolol hydrochloride | 1 |  |
| 49863 | 182020 | Propranolol 5mg/5ml oral solution sugar free | Propranolol hydrochloride | 1 |  |
| 26228 | 85670020 | Propanix LA 160mg Modified-release capsule (Ashbourne Pharmaceuticals Ltd) | Propranolol hydrochloride | 1 |  |
| 34945 | 59464020 | Propranolol 160mg Modified-release capsule (Lagap) | Propranolol hydrochloride | 1 |  |
| 52136 | 69495020 | Bedranol sr 160mg Capsule (Lagap) | Propranolol hydrochloride | 1 |  |
| 52609 | 942020 | Inderal LA 160mg capsules (Sigma Pharmaceuticals Plc) | Propranolol hydrochloride | 1 |  |
| 55228 | 927020 | Propranolol 40mg tablets (Boston Healthcare Ltd) | Propranolol hydrochloride | 1 |  |
| 34185 | 56194020 | Propranolol LA 80mg Modified-release capsule (Approved Prescription Services Ltd) | Propranolol hydrochloride | 1 |  |
| 1006 | 53253020 | Half Inderal LA 80mg capsules (AstraZeneca UK Ltd) | Propranolol hydrochloride | 1 |  |
| 68400 | 53253021 | Propranolol 160mg tablets (DE Pharmaceuticals) | Propranolol hydrochloride | 1 |  |
| 20468 | 72042020 | Half Beta-Prograne 80mg modified-release capsules (Tillomed Laboratories Ltd) | Propranolol hydrochloride | 1 |  |
| 24218 | 54826020 | Berkolol 160mg Tablet (Berk Pharmaceuticals Ltd) | Propranolol hydrochloride | 1 |  |
| 12495 | 54821020 | Berkolol 10mg Tablet (Berk Pharmaceuticals Ltd) | Propranolol hydrochloride | 1 |  |
| 43525 | 64019020 | Propranolol 10mg tablets (IVAX Pharmaceuticals UK Ltd) | Propranolol hydrochloride | 1 |  |
| 28788 | 85953020 | Half propatard la 80mg Modified-release capsule (Galen Ltd) | Propranolol hydrochloride | 1 |  |
| 12054 | 69745020 | Propranolol 80mg / Bendroflumethiazide 2.5mg capsules | Propranolol hydrochloride/Bendroflumethiazide | 0 | 1 |
| 4796 | 54256020 | Inderetic 80mg/2.5mg capsules (AstraZeneca UK Ltd) | Propranolol hydrochloride/Bendroflumethiazide | 0 | 1 |
| 23131 | 67621020 | Bendroflumethiazide 5mg with Propanolol 160mg modified-release capsules | Propranolol Hydrochloride/Bendroflumethiazide | 0 | 1 |
| 22912 | 67620020 | Bendroflumethiazide 2.5mg with Propanolol 80mg capsules | Propranolol Hydrochloride/Bendroflumethiazide | 0 | 1 |
| 8369 | 54259020 | Inderex 160mg/5mg modified-release capsules (AstraZeneca UK Ltd) | Propranolol hydrochloride/Bendroflumethiazide | 0 | 1 |
| 8987 | 69746020 | Propranolol 160mg modified-release / Bendroflumethiazide 5mg capsules | Propranolol hydrochloride/Bendroflumethiazide | 0 | 1 |
| 17783 | 57624020 | Spiroprop Tablet (Pharmacia Ltd) | Propranolol Hydrochloride/Spironolactone | 0 | 1 |
| 3691 | 66694020 | Sotalol 160mg with hydrochlorothiazide 25mg tablet | Sotalol Hydrochloride/Hydrochlorothiazide | 0 | 1 |
| 8061 | 66693020 | Sotalol 80mg with hydrochlorothiazide 12.5mg tablet | Sotalol Hydrochloride/Hydrochlorothiazide | 0 | 1 |
| 15042 | 66704020 | Tolerzide Tablet (Bristol-Myers Squibb Pharmaceuticals Ltd) | Sotalol Hydrochloride/Hydrochlorothiazide | 0 | 1 |
| 12456 | 51646020 | Sotazide Tablet (Bristol-Myers Squibb Pharmaceuticals Ltd) | Sotalol Hydrochloride/Hydrochlorothiazide | 0 | 1 |
| 7852 | 54409020 | Blocadren 10mg Tablet (Merck Sharp & Dohme Ltd) | Timolol maleate | 1 |  |
| 7853 | 69193020 | Timolol 10mg tablets | Timolol maleate | 1 |  |
| 12037 | 48400020 | Betim 10mg Tablet (ICN Pharmaceuticals France S.A.) | Timolol maleate | 1 |  |
| 29610 | 88268020 | Betim 10mg tablets (Meda Pharmaceuticals Ltd) | Timolol maleate | 1 |  |
| 21182 | 69280020 | Hydrochlorothiazide with timolol and amiloride 25mg with 10mg with 2.5mg Tablet | Timolol Maleate/Amiloride Hydrochloride/Hydrochlorothiazide | 0 | 1 |
| 25730 | 69274020 | Timolol maleate with amiloride and hydrochlorothiazide Tablet | Timolol Maleate/Amiloride Hydrochloride/Hydrochlorothiazide | 0 | 1 |
| 30519 | 69277020 | Amiloride with timolol with hydrochlorothiazide tablets | Timolol Maleate/Amiloride Hydrochloride/Hydrochlorothiazide | 0 | 1 |
| 48745 | 37067020 | Timolol 10mg / Amiloride 2.5mg / Hydrochlorothiazide 25mg tablets | Timolol maleate/Amiloride hydrochloride/Hydrochlorothiazide | 0 | 1 |
| 12651 | 67081020 | Timolol 10mg / Bendroflumethiazide 2.5mg tablets | Timolol maleate/Bendroflumethiazide | 0 | 1 |
| 12517 | 67082020 | Timolol maleate with bendroflumethiazide 20mg + 5mg Tablet | Timolol Maleate/Bendroflumethiazide | 0 | 1 |
| 19142 | 67631020 | Bendroflumethiazide 2.5mg with Timolol maleate 10mg tablets | Timolol Maleate/Bendroflumethiazide | 0 | 1 |
| 25363 | 88272020 | Prestim tablets (Meda Pharmaceuticals Ltd) | Timolol maleate/Bendroflumethiazide | 0 | 1 |
| 8623 | 52834020 | Prestim Tablet (ICN Pharmaceuticals France S.A.) | Timolol maleate/Bendroflumethiazide | 0 | 1 |
| 21025 | 67090020 | Prestim forte Tablet (LEO Pharma) | Timolol Maleate/Bendroflumethiazide | 0 | 1 |
| 4605 | 54433020 | Moducren tablets (Merck Sharp & Dohme Ltd) | Timolol maleate/Hydrochlorothiazide/Amiloride hydrochloride | 0 | 1 |

**Supplementary Table 7. Code list for ACEi**

| **ACEi** |  |  |  |  |  |
| --- | --- | --- | --- | --- | --- |
| **Prod**  **code** | **Gemscriptc**  **ode** | **Product**  **name** | **Drug**  **Substance**  **name** | **To**  **include** | **combination therapy to exclude** |
| 60744 | 22980021 | Perindopril erbumine 8mg / Amlodipine 5mg tablets | Amlodipine besilate/Perindopril erbumine | 0 | 1 |
| 63149 | 22978021 | Perindopril erbumine 8mg / Amlodipine 10mg tablets | Amlodipine besilate/Perindopril erbumine | 0 | 1 |
| 56850 | 84385020 | Ecopace 12.5mg tablets (AMCo) | Captopril | 1 |  |
| 34544 | 56638020 | Captopril 12.5mg Tablet (IVAX Pharmaceuticals UK Ltd) | Captopril | 1 |  |
| 26995 | 84491020 | Kaplon 25mg tablets (Teva UK Ltd) | Captopril | 1 |  |
| 3310 | 48565020 | Capoten 12.5mg tablets (Bristol-Myers Squibb Pharmaceuticals Ltd) | Captopril | 1 |  |
| 21943 | 84490020 | Kaplon 12.5mg tablets (Teva UK Ltd) | Captopril | 1 |  |
| 36742 | 93652020 | Captopril 2mg/5ml oral suspension | Captopril | 1 |  |
| 54544 | 19241020 | Captopril 25mg/5ml oral suspension | Captopril | 1 |  |
| 60823 | 21648021 | Noyada 5mg/5ml oral solution (Martindale Pharmaceuticals Ltd) | Captopril | 1 |  |
| 1121 | 60377020 | Captopril 12.5mg tablets | Captopril | 1 |  |
| 34937 | 56640020 | Captopril 50mg Tablet (IVAX Pharmaceuticals UK Ltd) | Captopril | 1 |  |
| 3839 | 48567020 | Capoten 50mg tablets (Bristol-Myers Squibb Pharmaceuticals Ltd) | Captopril | 1 |  |
| 37655 | 59733020 | Captopril 25mg tablets (Teva UK Ltd) | Captopril | 1 |  |
| 41633 | 56801020 | Captopril 12.5mg tablets (Actavis UK Ltd) | Captopril | 1 |  |
| 32514 | 84386020 | Ecopace 25mg tablets (AMCo) | Captopril | 1 |  |
| 30039 | 84463020 | Tensopril 25mg tablets (Teva UK Ltd) | Captopril | 1 |  |
| 43507 | 56634020 | Captopril 25mg Tablet (Generics (UK) Ltd) | Captopril | 1 |  |
| 17624 | 88718020 | Captopril 5mg/5ml oral suspension | Captopril | 1 |  |
| 52293 | 20854020 | Captopril 2mg capsules | Captopril | 1 |  |
| 34936 | 56686020 | Captopril 25mg Tablet (Lagap) | Captopril | 1 |  |
| 58195 | 19216020 | Captopril 12.5mg/5ml oral solution | Captopril | 1 |  |
| 60349 | 21646021 | Noyada 25mg/5ml oral solution (Martindale Pharmaceuticals Ltd) | Captopril | 1 |  |
| 43649 | 56644020 | Captopril 25mg tablets (A A H Pharmaceuticals Ltd) | Captopril | 1 |  |
| 46951 | 56643020 | Captopril 12.5mg tablets (A A H Pharmaceuticals Ltd) | Captopril | 1 |  |
| 64739 | 19240020 | Captopril 25mg/5ml oral solution (Special Order) | Captopril | 1 |  |
| 24482 | 85703020 | Captomex 50mg tablets (Actavis UK Ltd) | Captopril | 1 |  |
| 69599 | 19279020 | Captopril 500micrograms/5ml oral suspension | Captopril | 1 |  |
| 69192 | 91310020 | Captopril oral solution | Captopril | 1 |  |
| 56509 | 1375020 | Capoten 12.5mg tablets (Dowelhurst Ltd) | Captopril | 1 |  |
| 41743 | 59737020 | Captopril 50mg tablets (Teva UK Ltd) | Captopril | 1 |  |
| 46957 | 59293020 | Captopril 12.5mg tablets (Tillomed Laboratories Ltd) | Captopril | 1 |  |
| 43432 | 92446020 | Captopril 6.25mg tablets | Captopril | 1 |  |
| 34719 | 56635020 | Captopril 50mg Tablet (Generics (UK) Ltd) | Captopril | 1 |  |
| 35302 | 91890020 | Captopril 12.5mg/5ml oral suspension | Captopril | 1 |  |
| 52499 | 19239020 | Captopril 25mg/5ml oral solution | Captopril | 1 |  |
| 20849 | 84462020 | Tensopril 12.5mg tablets (Teva UK Ltd) | Captopril | 1 |  |
| 66597 | 19210020 | Captopril 10mg/5ml oral suspension | Captopril | 1 |  |
| 59699 | 21647021 | Captopril 5mg/5ml oral solution sugar free | Captopril | 1 |  |
| 33646 | 56633020 | Captopril 12.5mg Tablet (Generics (UK) Ltd) | Captopril | 1 |  |
| 28486 | 89909020 | Captopril 6.25mg/5ml oral suspension | Captopril | 1 |  |
| 41617 | 56802020 | Captopril 25mg tablets (Actavis UK Ltd) | Captopril | 1 |  |
| 39512 | 96425020 | Captopril 25mg/5ml oral suspension | Captopril | 1 |  |
| 1143 | 60378020 | Captopril 25mg tablets | Captopril | 1 |  |
| 70994 | 66757020 | Captopril 12.5mg tablets (Sandoz Ltd) | Captopril | 1 |  |
| 33336 | 69419020 | Captopril 5mg/5ml Oral suspension (Eldon Laboratories) | Captopril | 1 |  |
| 25998 | 85701020 | Captomex 12.5mg tablets (Actavis UK Ltd) | Captopril | 1 |  |
| 32048 | 84492020 | Kaplon 50mg tablets (Teva UK Ltd) | Captopril | 1 |  |
| 17633 | 87231020 | Captopril 3mg/5ml oral solution | Captopril | 1 |  |
| 34562 | 56639020 | Captopril 25mg Tablet (IVAX Pharmaceuticals UK Ltd) | Captopril | 1 |  |
| 18269 | 52608020 | Acepril 12.5mg tablets (Bristol-Myers Squibb Pharmaceuticals Ltd) | Captopril | 1 |  |
| 28820 | 85702020 | Captomex 25mg tablets (Actavis UK Ltd) | Captopril | 1 |  |
| 23478 | 84464020 | Tensopril 50mg tablets (Teva UK Ltd) | Captopril | 1 |  |
| 18325 | 52610020 | Acepril 50mg tablets (Bristol-Myers Squibb Pharmaceuticals Ltd) | Captopril | 1 |  |
| 44527 | 98847020 | Captopril 5mg/ml oral solution sugar free | Captopril | 1 |  |
| 69600 | 19229020 | Captopril 1mg/5ml oral suspension | Captopril | 1 |  |
| 1144 | 48566020 | Capoten 25mg tablets (Bristol-Myers Squibb Pharmaceuticals Ltd) | Captopril | 1 |  |
| 45228 | 91312020 | Captopril capsules | Captopril | 1 |  |
| 3069 | 52609020 | Acepril 25mg tablets (Bristol-Myers Squibb Pharmaceuticals Ltd) | Captopril | 1 |  |
| 46851 | 417021 | Captopril 5mg/5ml oral solution | Captopril | 1 |  |
| 15958 | 82305020 | Captopril 2mg tablets | Captopril | 1 |  |
| 59915 | 21645021 | Captopril 25mg/5ml oral solution sugar free | Captopril | 1 |  |
| 1807 | 60379020 | Captopril 50mg tablets | Captopril | 1 |  |
| 15135 | 69269020 | Hydrochlorothiazide with captopril 12.5mg with 25mg Tablet | Captopril/Hydrochlorothiazide | 0 | 1 |
| 11133 | 69268020 | Hydrochlorothiazide with captopril 25mg with 50mg Tablet | Captopril/Hydrochlorothiazide | 0 | 1 |
| 10902 | 68129020 | Captopril 50mg with Hydrochlorothiazide 25mg tablets | Captopril/Hydrochlorothiazide | 0 | 1 |
| 11641 | 68130020 | Captopril 25mg with Hydrochlorothiazide 12.5mg tablets | Captopril/Hydrochlorothiazide | 0 | 1 |
| 15605 | 49385020 | Cilazapril 250micrograms tablets | Cilazapril | 1 |  |
| 23642 | 50587020 | Vascace 0.25mg Tablet (Roche Products Ltd) | Cilazapril | 1 |  |
| 16196 | 55291020 | Vascace 5mg tablets (Roche Products Ltd) | Cilazapril monohydrate | 1 |  |
| 12574 | 49387020 | Cilazapril 1mg tablets | Cilazapril monohydrate | 1 |  |
| 12412 | 54164020 | Cilazapril 2.5mg tablets | Cilazapril monohydrate | 1 |  |
| 21053 | 50588020 | Vascace 500microgram tablets (Roche Products Ltd) | Cilazapril monohydrate | 1 |  |
| 12411 | 49386020 | Cilazapril 500microgram tablets | Cilazapril monohydrate | 1 |  |
| 16212 | 50589020 | Vascace 1mg tablets (Roche Products Ltd) | Cilazapril monohydrate | 1 |  |
| 16197 | 55290020 | Vascace 2.5mg tablets (Roche Products Ltd) | Cilazapril monohydrate | 1 |  |
| 13026 | 54165020 | Cilazapril 5mg tablets | Cilazapril monohydrate | 1 |  |
| 42908 | 61247020 | Enalapril 5mg tablets (IVAX Pharmaceuticals UK Ltd) | Enalapril maleate | 1 |  |
| 34768 | 61253020 | Enalapril 20mg tablets (IVAX Pharmaceuticals UK Ltd) | Enalapril maleate | 1 |  |
| 20188 | 60798020 | Enalapril 2.5mg wafer | Enalapril Maleate | 1 |  |
| 34453 | 61301020 | Enalapril 20mg tablets (Mylan) | Enalapril maleate | 1 |  |
| 16708 | 60797020 | Enalapril titration pack | Enalapril Maleate | 1 |  |
| 8106 | 55119020 | Innovace 2.5mg tablets (Merck Sharp & Dohme Ltd) | Enalapril maleate | 1 |  |
| 41417 | 60324020 | Enalapril 2.5mg tablets (A A H Pharmaceuticals Ltd) | Enalapril maleate | 1 |  |
| 43563 | 61391020 | Enalapril 2.5mg tablets (Zentiva) | Enalapril maleate | 1 |  |
| 61133 | 1425020 | Enalapril 10mg tablets (Phoenix Healthcare Distribution Ltd) | Enalapril maleate | 1 |  |
| 23252 | 73042020 | Pralenal 10 tablets (Opus Pharmaceuticals Ltd) | Enalapril maleate | 1 |  |
| 27871 | 86081020 | Innovace melt 10mg Wafer (Merck Sharp & Dohme Ltd) | Enalapril Maleate | 1 |  |
| 8105 | 58451020 | Innovace 20mg tablets (Merck Sharp & Dohme Ltd) | Enalapril maleate | 1 |  |
| 45217 | 63368020 | Enalapril 5mg tablets (Kent Pharmaceuticals Ltd) | Enalapril maleate | 1 |  |
| 34400 | 55722020 | Enalapril 5mg Tablet (Dowelhurst Ltd) | Enalapril maleate | 1 |  |
| 62860 | 21324021 | Enalapril 5mg tablets (DE Pharmaceuticals) | Enalapril maleate | 1 |  |
| 22439 | 83449020 | Ednyt 20mg Tablet (Dominion Pharma) | Enalapril maleate | 1 |  |
| 34798 | 61362020 | Enalapril 20mg tablets (Sandoz Ltd) | Enalapril maleate | 1 |  |
| 52010 | 1418020 | Enalapril 10mg tablets (Alliance Healthcare (Distribution) Ltd) | Enalapril maleate | 1 |  |
| 53915 | 61780020 | Enalapril 5mg tablets (Dexcel-Pharma Ltd) | Enalapril maleate | 1 |  |
| 34953 | 61397020 | Enalapril 20mg tablets (Zentiva) | Enalapril maleate | 1 |  |
| 31587 | 86077020 | Innovace melt 20mg Wafer (Merck Sharp & Dohme Ltd) | Enalapril Maleate | 1 |  |
| 32241 | 60326020 | Enalapril 10mg tablets (A A H Pharmaceuticals Ltd) | Enalapril maleate | 1 |  |
| 46974 | 61297020 | Enalapril 5mg tablets (Mylan) | Enalapril maleate | 1 |  |
| 60143 | 38499020 | Enalapril 5mg tablets (Medreich Plc) | Enalapril maleate | 1 |  |
| 41694 | 61246020 | Enalapril 2.5mg tablets (IVAX Pharmaceuticals UK Ltd) | Enalapril maleate | 1 |  |
| 22708 | 77941020 | Enalapril 5mg wafer | Enalapril Maleate | 1 |  |
| 37087 | 94156020 | Enalapril 5mg/5ml oral suspension | Enalapril maleate | 1 |  |
| 196 | 60791020 | Enalapril 5mg tablets | Enalapril maleate | 1 |  |
| 34712 | 63374020 | Enalapril 20mg tablets (Kent Pharmaceuticals Ltd) | Enalapril maleate | 1 |  |
| 35794 | 60325020 | Enalapril 5mg tablets (A A H Pharmaceuticals Ltd) | Enalapril maleate | 1 |  |
| 50863 | 19819020 | Enalapril 5mg/5ml oral solution (Drug Tariff Special Order) | Enalapril maleate | 1 |  |
| 64877 | 61779020 | Enalapril 2.5mg tablets (Dexcel-Pharma Ltd) | Enalapril maleate | 1 |  |
| 11197 | 86080020 | Innovace melt 5mg Wafer (Merck Sharp & Dohme Ltd) | Enalapril Maleate | 1 |  |
| 68496 | 63371020 | Enalapril 10mg tablets (Kent Pharmaceuticals Ltd) | Enalapril maleate | 1 |  |
| 29530 | 86079020 | Innovace melt 2.5mg Wafer (Merck Sharp & Dohme Ltd) | Enalapril Maleate | 1 |  |
| 43411 | 61356020 | Enalapril 5mg tablets (Sandoz Ltd) | Enalapril maleate | 1 |  |
| 42894 | 61262020 | Enalapril 10mg tablets (Teva UK Ltd) | Enalapril maleate | 1 |  |
| 24041 | 77943020 | Enalapril 20mg wafer | Enalapril Maleate | 1 |  |
| 1904 | 60796020 | Enalapril 20mg tablets | Enalapril maleate | 1 |  |
| 8830 | 55121020 | Innovace 10mg tablets (Merck Sharp & Dohme Ltd) | Enalapril maleate | 1 |  |
| 33057 | 75030020 | Ednyt 5mg Tablet (Dominion Pharma) | Enalapril maleate | 1 |  |
| 448 | 60790020 | Enalapril 2.5mg tablets | Enalapril maleate | 1 |  |
| 28127 | 61260020 | Enalapril 2.5mg tablets (Teva UK Ltd) | Enalapril maleate | 1 |  |
| 31716 | 61347020 | Enalapril 20mg tablets (Actavis UK Ltd) | Enalapril maleate | 1 |  |
| 37080 | 94154020 | Enalapril 5mg/5ml oral solution | Enalapril maleate | 1 |  |
| 66895 | 19803020 | Enalapril 10mg/5ml oral suspension | Enalapril maleate | 1 |  |
| 42902 | 61266020 | Enalapril 20mg tablets (Teva UK Ltd) | Enalapril maleate | 1 |  |
| 52882 | 40203020 | Enalapril 5mg/5ml oral suspension sugar free | Enalapril maleate | 1 |  |
| 8800 | 55120020 | Innovace 5mg tablets (Merck Sharp & Dohme Ltd) | Enalapril maleate | 1 |  |
| 63322 | 71478020 | Enalapril 10mg tablets (Almus Pharmaceuticals Ltd) | Enalapril maleate | 1 |  |
| 44657 | 75029020 | Ednyt 2.5mg Tablet (Dominion Pharma) | Enalapril maleate | 1 |  |
| 57378 | 30788020 | Enalapril 2mg/5ml oral suspension | Enalapril maleate | 1 |  |
| 1299 | 60792020 | Enalapril 10mg tablets | Enalapril maleate | 1 |  |
| 64062 | 30780020 | Enalapril 1mg/5ml oral suspension | Enalapril maleate | 1 |  |
| 15085 | 58452020 | Innovace Titration pack (Merck Sharp & Dohme Ltd) | Enalapril Maleate | 1 |  |
| 19208 | 61344020 | Enalapril 10mg tablets (Actavis UK Ltd) | Enalapril maleate | 1 |  |
| 57882 | 19854020 | Enalapril 2.5mg/5ml oral suspension | Enalapril maleate | 1 |  |
| 34952 | 61298020 | Enalapril 10mg tablets (Mylan) | Enalapril maleate | 1 |  |
| 36753 | 75031020 | Ednyt 10mg Tablet (Dominion Pharma) | Enalapril maleate | 1 |  |
| 53719 | 1427020 | Enalapril 20mg tablets (Alliance Healthcare (Distribution) Ltd) | Enalapril maleate | 1 |  |
| 33078 | 60335020 | Enalapril 20mg tablets (A A H Pharmaceuticals Ltd) | Enalapril maleate | 1 |  |
| 50780 | 30786020 | Enalapril 2mg/5ml oral solution | Enalapril maleate | 1 |  |
| 42723 | 73041020 | Pralenal 5 tablets (Opus Pharmaceuticals Ltd) | Enalapril maleate | 1 |  |
| 42901 | 61261020 | Enalapril 5mg tablets (Teva UK Ltd) | Enalapril maleate | 1 |  |
| 50334 | 19817020 | Enalapril 4mg/5ml oral suspension | Enalapril maleate | 1 |  |
| 55903 | 61781020 | Enalapril 10mg tablets (Dexcel-Pharma Ltd) | Enalapril maleate | 1 |  |
| 41746 | 61357020 | Enalapril 10mg tablets (Sandoz Ltd) | Enalapril maleate | 1 |  |
| 59996 | 1435020 | Enalapril 20mg tablets (Milpharm Ltd) | Enalapril maleate | 1 |  |
| 58751 | 19799020 | Enalapril 1.25mg/5ml oral suspension | Enalapril maleate | 1 |  |
| 13755 | 77942020 | Enalapril 10mg wafer | Enalapril Maleate | 1 |  |
| 5189 | 51068020 | Enalapril 20mg / Hydrochlorothiazide 12.5mg tablets | Enalapril maleate/Hydrochlorothiazide | 0 | 1 |
| 1021 | 51047020 | Innozide 20mg/12.5mg tablets (Merck Sharp & Dohme Ltd) | Enalapril maleate/Hydrochlorothiazide | 0 | 1 |
| 11567 | 81446020 | Ramipril 5mg with felodipine 5mg modified-release tablet | Felodipine/Ramipril | 0 | 1 |
| 28438 | 85815020 | Triapin 2.5mg/2.5mg modified-release tablets (Sanofi) | Felodipine/Ramipril | 0 | 1 |
| 11965 | 81445020 | Ramipril 2.5mg with felodipine 2.5mg modified-release tablet | Felodipine/Ramipril | 0 | 1 |
| 17474 | 78452020 | Felodipine 5mg modified-release / Ramipril 5mg tablets | Felodipine/Ramipril | 0 | 1 |
| 17006 | 85824020 | Triapin 5mg/5mg modified-release tablets (Sanofi) | Felodipine/Ramipril | 0 | 1 |
| 21162 | 78451020 | Felodipine 2.5mg modified-release / Ramipril 2.5mg tablets | Felodipine/Ramipril | 0 | 1 |
| 13589 | 74175020 | Staril 20mg tablets (Bristol-Myers Squibb Pharmaceuticals Ltd) | Fosinopril sodium | 1 |  |
| 633 | 74178020 | Fosinopril 10mg tablets | Fosinopril sodium | 1 |  |
| 67307 | 1602020 | Staril 20mg tablets (Dowelhurst Ltd) | Fosinopril sodium | 1 |  |
| 4571 | 74174020 | Staril 10mg tablets (Bristol-Myers Squibb Pharmaceuticals Ltd) | Fosinopril sodium | 1 |  |
| 5861 | 74179020 | Fosinopril 20mg tablets | Fosinopril sodium | 1 |  |
| 3203 | 68134020 | Capozide LS Tablet (E R Squibb and Sons Ltd) | Hydrochlorothiazide/Captopril | 0 | 1 |
| 39227 | 95527020 | Capozide LS 12.5mg/25mg tablets (Bristol-Myers Squibb Pharmaceuticals Ltd) | Hydrochlorothiazide/Captopril | 0 | 1 |
| 18263 | 59646020 | Acezide 25mg/50mg tablets (Bristol-Myers Squibb Pharmaceuticals Ltd) | Hydrochlorothiazide/Captopril | 0 | 1 |
| 11351 | 84482020 | Co-zidocapt 25mg/50mg tablets | Hydrochlorothiazide/Captopril | 0 | 1 |
| 1520 | 68133020 | Capozide 25mg/50mg tablets (Bristol-Myers Squibb Pharmaceuticals Ltd) | Hydrochlorothiazide/Captopril | 0 | 1 |
| 11561 | 84483020 | Co-zidocapt 12.5mg/25mg tablets | Hydrochlorothiazide/Captopril | 0 | 1 |
| 32166 | 80831020 | Capto-co 25mg+50mg Tablet (IVAX Pharmaceuticals UK Ltd) | Hydrochlorothiazide/Captopril | 0 | 1 |
| 15031 | 74293020 | Accuretic 12.5mg/10mg tablets (Pfizer Ltd) | Hydrochlorothiazide/Quinapril hydrochloride | 0 | 1 |
| 15108 | 74296020 | Quinapril 10mg / Hydrochlorothiazide 12.5mg tablets | Hydrochlorothiazide/Quinapril hydrochloride | 0 | 1 |
| 6408 | 81266020 | Tanatril 5mg tablets (Mitsubishi Tanabe Pharma Europe Ltd) | Imidapril hydrochloride | 1 |  |
| 12815 | 81267020 | Tanatril 10mg tablets (Mitsubishi Tanabe Pharma Europe Ltd) | Imidapril hydrochloride | 1 |  |
| 16924 | 81181020 | Imidapril 5mg tablets | Imidapril hydrochloride | 1 |  |
| 18219 | 81183020 | Imidapril 20mg tablets | Imidapril hydrochloride | 1 |  |
| 32560 | 81268020 | Tanatril 20mg tablets (Mitsubishi Tanabe Pharma Europe Ltd) | Imidapril hydrochloride | 1 |  |
| 12858 | 81182020 | Imidapril 10mg tablets | Imidapril hydrochloride | 1 |  |
| 6794 | 83957020 | Perindopril erbumine 4mg / Indapamide 1.25mg tablets | Indapamide/Perindopril erbumine | 0 | 1 |
| 14228 | 84712020 | Coversyl Plus tablets (Servier Laboratories Ltd) | Indapamide/Perindopril erbumine | 0 | 1 |
| 56157 | 47939020 | Perindopril tosilate 5mg / Indapamide 1.25mg tablets | Indapamide/Perindopril tosilate | 0 | 1 |
| 33977 | 65098020 | Lisinopril 10mg tablets (Mylan) | Lisinopril | 1 |  |
| 45337 | 65231020 | Lisinopril 5mg tablets (Actavis UK Ltd) | Lisinopril | 1 |  |
| 56505 | 1458020 | Zestril 5mg tablets (Lexon (UK) Ltd) | Lisinopril | 1 |  |
| 58294 | 1461020 | Lisinopril 5mg tablets (Accord Healthcare Ltd) | Lisinopril | 1 |  |
| 46975 | 65137020 | Lisinopril 5mg tablets (Sandoz Ltd) | Lisinopril | 1 |  |
| 30921 | 64809020 | Lisinopril 2.5mg tablets (Teva UK Ltd) | Lisinopril | 1 |  |
| 56510 | 1481020 | Zestril 20mg tablets (Sigma Pharmaceuticals Plc) | Lisinopril | 1 |  |
| 68094 | 1456020 | Lisinopril 5mg tablets (Sigma Pharmaceuticals Plc) | Lisinopril | 1 |  |
| 69074 | 1479020 | Lisinopril 20mg tablets (Relonchem Ltd) | Lisinopril | 1 |  |
| 43413 | 65206020 | Lisinopril 20mg tablets (A A H Pharmaceuticals Ltd) | Lisinopril | 1 |  |
| 8268 | 68567020 | Zestril 20mg tablets (AstraZeneca UK Ltd) | Lisinopril | 1 |  |
| 3720 | 68562020 | Zestril 2.5mg tablets (AstraZeneca UK Ltd) | Lisinopril | 1 |  |
| 41573 | 65180020 | Lisopress 10mg tablets (Teva UK Ltd) | Lisinopril | 1 |  |
| 69269 | 14473021 | Lisinopril 20mg tablets (Waymade Healthcare Plc) | Lisinopril | 1 |  |
| 43416 | 65202020 | Lisinopril 10mg tablets (A A H Pharmaceuticals Ltd) | Lisinopril | 1 |  |
| 12313 | 68559020 | Carace 20mg tablets (Bristol-Myers Squibb Pharmaceuticals Ltd) | Lisinopril | 1 |  |
| 59111 | 1473020 | Lisinopril 20mg tablets (Alliance Healthcare (Distribution) Ltd) | Lisinopril | 1 |  |
| 55896 | 65227020 | Lisinopril 2.5mg tablets (Actavis UK Ltd) | Lisinopril | 1 |  |
| 45816 | 71542020 | Lisinopril 5mg tablets (Almus Pharmaceuticals Ltd) | Lisinopril | 1 |  |
| 65985 | 23676021 | Lisinopril 2.5mg tablets (DE Pharmaceuticals) | Lisinopril | 1 |  |
| 65416 | 29672021 | Lisinopril 5mg tablets (Lupin (Europe) Ltd) | Lisinopril | 1 |  |
| 41522 | 65183020 | Lisopress 20mg tablets (Teva UK Ltd) | Lisinopril | 1 |  |
| 43566 | 65134020 | Lisinopril 2.5mg tablets (Sandoz Ltd) | Lisinopril | 1 |  |
| 43418 | 65199020 | Lisinopril 5mg tablets (A A H Pharmaceuticals Ltd) | Lisinopril | 1 |  |
| 14387 | 68555020 | Carace 5mg tablets (Bristol-Myers Squibb Pharmaceuticals Ltd) | Lisinopril | 1 |  |
| 19198 | 64822020 | Lisinopril 20mg tablets (Teva UK Ltd) | Lisinopril | 1 |  |
| 20975 | 90115020 | Lisinopril 7.5mg/5ml oral suspension | Lisinopril | 1 |  |
| 65 | 68572020 | Lisinopril 10mg tablets | Lisinopril | 1 |  |
| 58461 | 1437020 | Lisinopril 2.5mg tablets (Kent Pharmaceuticals Ltd) | Lisinopril | 1 |  |
| 11987 | 88326020 | Lisinopril 5mg/5ml oral solution | Lisinopril | 1 |  |
| 54288 | 69238020 | Lisinopril 10mg tablets (Arrow Generics Ltd) | Lisinopril | 1 |  |
| 6807 | 68563020 | Zestril 5mg tablets (AstraZeneca UK Ltd) | Lisinopril | 1 |  |
| 60010 | 1462020 | Lisinopril 10mg tablets (Kent Pharmaceuticals Ltd) | Lisinopril | 1 |  |
| 45300 | 65236020 | Lisinopril 10mg tablets (Actavis UK Ltd) | Lisinopril | 1 |  |
| 58258 | 20373020 | Lisinopril 2.5mg/5ml oral suspension | Lisinopril | 1 |  |
| 65102 | 1469020 | Lisinopril 10mg tablets (Sigma Pharmaceuticals Plc) | Lisinopril | 1 |  |
| 67075 | 53285021 | Lisinopril 2.5mg tablets (Mawdsley-Brooks & Company Ltd) | Lisinopril | 1 |  |
| 67194 | 1445020 | Lisinopril 2.5mg tablets (Bristol Laboratories Ltd) | Lisinopril | 1 |  |
| 6806 | 68564020 | Zestril 10mg tablets (AstraZeneca UK Ltd) | Lisinopril | 1 |  |
| 55639 | 1472020 | Lisinopril 10mg tablets (Accord Healthcare Ltd) | Lisinopril | 1 |  |
| 51433 | 66292020 | Lisinopril 20mg tablets (Tillomed Laboratories Ltd) | Lisinopril | 1 |  |
| 47159 | 71545020 | Lisinopril 10mg tablets (Almus Pharmaceuticals Ltd) | Lisinopril | 1 |  |
| 65536 | 1436020 | Lisinopril 2.5mg tablets (Alliance Healthcare (Distribution) Ltd) | Lisinopril | 1 |  |
| 56279 | 20371020 | Lisinopril 2.5mg/5ml oral solution | Lisinopril | 1 |  |
| 34799 | 65220020 | Lisinopril 20mg tablets (Zentiva) | Lisinopril | 1 |  |
| 59109 | 66285020 | Lisinopril 5mg tablets (Tillomed Laboratories Ltd) | Lisinopril | 1 |  |
| 41538 | 65173020 | Lisopress 2.5mg tablets (Teva UK Ltd) | Lisinopril | 1 |  |
| 63559 | 1474020 | Lisinopril 20mg tablets (Kent Pharmaceuticals Ltd) | Lisinopril | 1 |  |
| 57048 | 65217020 | Lisinopril 10mg tablets (Zentiva) | Lisinopril | 1 |  |
| 41532 | 65176020 | Lisopress 5mg tablets (Teva UK Ltd) | Lisinopril | 1 |  |
| 58863 | 1471020 | Lisinopril 10mg tablets (Phoenix Healthcare Distribution Ltd) | Lisinopril | 1 |  |
| 58871 | 14472021 | Lisinopril 10mg tablets (Waymade Healthcare Plc) | Lisinopril | 1 |  |
| 62564 | 20367020 | Lisinopril 10mg/5ml oral solution | Lisinopril | 1 |  |
| 78 | 68571020 | Lisinopril 5mg tablets | Lisinopril | 1 |  |
| 54037 | 1467020 | Lisinopril 10mg tablets (Relonchem Ltd) | Lisinopril | 1 |  |
| 16701 | 68556020 | Carace 10mg tablets (Bristol-Myers Squibb Pharmaceuticals Ltd) | Lisinopril | 1 |  |
| 60232 | 65214020 | Lisinopril 5mg tablets (Zentiva) | Lisinopril | 1 |  |
| 54512 | 91738020 | Lisinopril Oral solution | Lisinopril | 1 |  |
| 45324 | 65239020 | Lisinopril 20mg tablets (Actavis UK Ltd) | Lisinopril | 1 |  |
| 53551 | 1484020 | Lisinopril 20mg tablets (Phoenix Healthcare Distribution Ltd) | Lisinopril | 1 |  |
| 34471 | 65081020 | Lisinopril 5mg tablets (Mylan) | Lisinopril | 1 |  |
| 58451 | 71538020 | Lisinopril 2.5mg tablets (Almus Pharmaceuticals Ltd) | Lisinopril | 1 |  |
| 63824 | 20369020 | Lisinopril 10mg/5ml oral suspension | Lisinopril | 1 |  |
| 63030 | 23678021 | Lisinopril 10mg tablets (DE Pharmaceuticals) | Lisinopril | 1 |  |
| 66772 | 14470021 | Lisinopril 2.5mg tablets (Waymade Healthcare Plc) | Lisinopril | 1 |  |
| 34696 | 65102020 | Lisinopril 20mg tablets (Mylan) | Lisinopril | 1 |  |
| 10882 | 68554020 | Carace 2.5mg tablets (Bristol-Myers Squibb Pharmaceuticals Ltd) | Lisinopril | 1 |  |
| 55456 | 1448020 | Lisinopril 5mg tablets (Alliance Healthcare (Distribution) Ltd) | Lisinopril | 1 |  |
| 55002 | 1486020 | Lisinopril 20mg tablets (Accord Healthcare Ltd) | Lisinopril | 1 |  |
| 66558 | 23677021 | Lisinopril 5mg tablets (DE Pharmaceuticals) | Lisinopril | 1 |  |
| 37778 | 94657020 | Lisinopril 5mg/5ml oral suspension | Lisinopril | 1 |  |
| 61262 | 1483020 | Lisinopril 20mg tablets (Bristol Laboratories Ltd) | Lisinopril | 1 |  |
| 60309 | 1454020 | Lisinopril 5mg tablets (Relonchem Ltd) | Lisinopril | 1 |  |
| 54928 | 1470020 | Lisinopril 10mg tablets (Bristol Laboratories Ltd) | Lisinopril | 1 |  |
| 19223 | 64818020 | Lisinopril 10mg tablets (Teva UK Ltd) | Lisinopril | 1 |  |
| 64902 | 53969021 | Lisinopril 5mg/5ml oral solution sugar free | Lisinopril | 1 |  |
| 277 | 68570020 | Lisinopril 2.5mg tablets | Lisinopril | 1 |  |
| 58682 | 66939020 | Lisinopril 2.5mg tablets (Mylan) | Lisinopril | 1 |  |
| 70667 | 53286021 | Lisinopril 5mg tablets (Mawdsley-Brooks & Company Ltd) | Lisinopril | 1 |  |
| 53820 | 69229020 | Lisinopril 5mg tablets (Arrow Generics Ltd) | Lisinopril | 1 |  |
| 69 | 68575020 | Lisinopril 20mg tablets | Lisinopril | 1 |  |
| 46979 | 65143020 | Lisinopril 20mg tablets (Sandoz Ltd) | Lisinopril | 1 |  |
| 67795 | 71548020 | Lisinopril 20mg tablets (Almus Pharmaceuticals Ltd) | Lisinopril | 1 |  |
| 65983 | 29671021 | Lisinopril 2.5mg tablets (Lupin (Europe) Ltd) | Lisinopril | 1 |  |
| 66622 | 23679021 | Lisinopril 20mg tablets (DE Pharmaceuticals) | Lisinopril | 1 |  |
| 32597 | 65140020 | Lisinopril 10mg tablets (Sandoz Ltd) | Lisinopril | 1 |  |
| 43412 | 65196020 | Lisinopril 2.5mg tablets (A A H Pharmaceuticals Ltd) | Lisinopril | 1 |  |
| 68247 | 1457020 | Lisinopril 5mg tablets (Bristol Laboratories Ltd) | Lisinopril | 1 |  |
| 55588 | 1482020 | Lisinopril 20mg tablets (Sigma Pharmaceuticals Plc) | Lisinopril | 1 |  |
| 57588 | 1446020 | Zestril 2.5mg tablets (Mawdsley-Brooks & Company Ltd) | Lisinopril | 1 |  |
| 19204 | 64814020 | Lisinopril 5mg tablets (Teva UK Ltd) | Lisinopril | 1 |  |
| 54283 | 20380020 | Lisinopril 5mg/5ml oral suspension (Special Order) | Lisinopril | 1 |  |
| 53271 | 1463020 | Lisinopril 10mg tablets (Alliance Healthcare (Distribution) Ltd) | Lisinopril | 1 |  |
| 52088 | 1459020 | Lisinopril 5mg tablets (Phoenix Healthcare Distribution Ltd) | Lisinopril | 1 |  |
| 60097 | 65209020 | Lisinopril 2.5mg tablets (Zentiva) | Lisinopril | 1 |  |
| 38995 | 95877020 | Zestoretic 20 tablets (AstraZeneca UK Ltd) | Lisinopril/Hydrochlorothiazide | 0 | 1 |
| 21231 | 87820020 | Caralpha 20mg/12.5mg tablets (Actavis UK Ltd) | Lisinopril/Hydrochlorothiazide | 0 | 1 |
| 6786 | 50823020 | Lisinopril 10mg / Hydrochlorothiazide 12.5mg tablets | Lisinopril/Hydrochlorothiazide | 0 | 1 |
| 55399 | 67847020 | Lisinopril 20mg / Hydrochlorothiazide 12.5mg tablets (A A H Pharmaceuticals Ltd) | Lisinopril/Hydrochlorothiazide | 0 | 1 |
| 33353 | 67697020 | Lisinopril 20mg / Hydrochlorothiazide 12.5mg tablets (Teva UK Ltd) | Lisinopril/Hydrochlorothiazide | 0 | 1 |
| 56244 | 1491020 | Lisinopril 20mg / Hydrochlorothiazide 12.5mg tablets (Tillomed Laboratories Ltd) | Lisinopril/Hydrochlorothiazide | 0 | 1 |
| 37710 | 67694020 | Lisinopril 10mg / Hydrochlorothiazide 12.5mg tablets (Teva UK Ltd) | Lisinopril/Hydrochlorothiazide | 0 | 1 |
| 39137 | 95875020 | Zestoretic 10 tablets (AstraZeneca UK Ltd) | Lisinopril/Hydrochlorothiazide | 0 | 1 |
| 9764 | 72035020 | Carace 20 Tablet (Bristol-Myers Squibb Pharmaceuticals Ltd) | Lisinopril/Hydrochlorothiazide | 0 | 1 |
| 71115 | 1500020 | Zestoretic 10 tablets (Waymade Healthcare Plc) | Lisinopril/Hydrochlorothiazide | 0 | 1 |
| 17655 | 72036020 | Carace 10 Tablet (Bristol-Myers Squibb Pharmaceuticals Ltd) | Lisinopril/Hydrochlorothiazide | 0 | 1 |
| 39147 | 95901020 | Carace 20 Plus tablets (Merck Sharp & Dohme Ltd) | Lisinopril/Hydrochlorothiazide | 0 | 1 |
| 6359 | 51463020 | Zestoretic 10- 10mg+12.5mg Tablet (AstraZeneca UK Ltd) | Lisinopril/Hydrochlorothiazide | 0 | 1 |
| 39242 | 95903020 | Carace 10 Plus tablets (Merck Sharp & Dohme Ltd) | Lisinopril/Hydrochlorothiazide | 0 | 1 |
| 67767 | 75921020 | Lisinopril 10mg / Hydrochlorothiazide 12.5mg tablets (Almus Pharmaceuticals Ltd) | Lisinopril/Hydrochlorothiazide | 0 | 1 |
| 2982 | 51462020 | Zestoretic 20- 20mg+12.5mg Tablet (AstraZeneca UK Ltd) | Lisinopril/Hydrochlorothiazide | 0 | 1 |
| 57539 | 1503020 | Zestoretic 10 tablets (Sigma Pharmaceuticals Plc) | Lisinopril/Hydrochlorothiazide | 0 | 1 |
| 6468 | 50822020 | Lisinopril 20mg / Hydrochlorothiazide 12.5mg tablets | Lisinopril/Hydrochlorothiazide | 0 | 1 |
| 54201 | 75924020 | Lisinopril 20mg / Hydrochlorothiazide 12.5mg tablets (Almus Pharmaceuticals Ltd) | Lisinopril/Hydrochlorothiazide | 0 | 1 |
| 15121 | 80674020 | Moexipril 7.5mg tablets | Moexipril hydrochloride | 1 |  |
| 17120 | 80675020 | Moexipril 15mg tablets | Moexipril hydrochloride | 1 |  |
| 28725 | 80672020 | Perdix 15mg tablets (UCB Pharma Ltd) | Moexipril hydrochloride | 1 |  |
| 28724 | 80671020 | Perdix 7.5mg tablets (UCB Pharma Ltd) | Moexipril hydrochloride | 1 |  |
| 51807 | 38770020 | Coversyl Arginine 5mg tablets (DE Pharmaceuticals) | Perindopril arginine | 1 |  |
| 37930 | 94699020 | Perindopril arginine 5mg tablets | Perindopril arginine | 1 |  |
| 37971 | 94701020 | Perindopril arginine 10mg tablets | Perindopril arginine | 1 |  |
| 38034 | 94703020 | Coversyl Arginine 2.5mg tablets (Servier Laboratories Ltd) | Perindopril arginine | 1 |  |
| 50347 | 34964020 | Coversyl Arginine 5mg tablets (Waymade Healthcare Plc) | Perindopril arginine | 1 |  |
| 37964 | 94697020 | Perindopril arginine 2.5mg tablets | Perindopril arginine | 1 |  |
| 38026 | 94707020 | Coversyl Arginine 10mg tablets (Servier Laboratories Ltd) | Perindopril arginine | 1 |  |
| 37965 | 94705020 | Coversyl Arginine 5mg tablets (Servier Laboratories Ltd) | Perindopril arginine | 1 |  |
| 51258 | 34961020 | Coversyl Arginine Plus 5mg/1.25mg tablets (DE Pharmaceuticals) | Perindopril arginine/Indapamide | 0 | 1 |
| 37978 | 94277020 | Perindopril arginine 5mg / Indapamide 1.25mg tablets | Perindopril arginine/Indapamide | 0 | 1 |
| 37908 | 94709020 | Coversyl Arginine Plus 5mg/1.25mg tablets (Servier Laboratories Ltd) | Perindopril arginine/Indapamide | 0 | 1 |
| 48214 | 76375020 | Perindopril erbumine 4mg tablets (Actavis UK Ltd) | Perindopril erbumine | 1 |  |
| 59972 | 1543020 | Perindopril erbumine 2mg tablets (Alliance Healthcare (Distribution) Ltd) | Perindopril erbumine | 1 |  |
| 11983 | 90557020 | Perindopril erbumine 4mg/5ml oral suspension | Perindopril erbumine | 1 |  |
| 5800 | 72569020 | Coversyl 4mg tablets (Servier Laboratories Ltd) | Perindopril erbumine | 1 |  |
| 61270 | 45151020 | Perindopril erbumine 4mg tablets (Accord Healthcare Ltd) | Perindopril erbumine | 1 |  |
| 14960 | 83699020 | Coversyl 8mg tablets (Servier Laboratories Ltd) | Perindopril erbumine | 1 |  |
| 65273 | 76557020 | Perindopril erbumine 4mg tablets (Mylan) | Perindopril erbumine | 1 |  |
| 54986 | 35397020 | Perindopril erbumine 8mg/5ml oral suspension | Perindopril erbumine | 1 |  |
| 59770 | 15619021 | Perindopril erbumine 4mg tablets (Aurobindo Pharma Ltd) | Perindopril erbumine | 1 |  |
| 43012 | 91957020 | Perindopril erbumine oral solution | Perindopril Erbumine | 1 |  |
| 67269 | 1540020 | Coversyl 2mg tablets (Waymade Healthcare Plc) | Perindopril erbumine | 1 |  |
| 48049 | 76554020 | Perindopril erbumine 2mg tablets (Mylan) | Perindopril erbumine | 1 |  |
| 58874 | 1550020 | Perindopril erbumine 2mg tablets (Somex Pharma) | Perindopril erbumine | 1 |  |
| 53058 | 40612020 | Perindopril erbumine 8mg tablets (Sandoz Ltd) | Perindopril erbumine | 1 |  |
| 58843 | 1542020 | Perindopril erbumine 2mg tablets (Kent Pharmaceuticals Ltd) | Perindopril erbumine | 1 |  |
| 56162 | 1556020 | Perindopril erbumine 4mg tablets (Consilient Health Ltd) | Perindopril erbumine | 1 |  |
| 71004 | 45210020 | Perindopril erbumine 8mg tablets (Accord Healthcare Ltd) | Perindopril erbumine | 1 |  |
| 45938 | 75040020 | Perindopril erbumine 8mg tablets (Teva UK Ltd) | Perindopril erbumine | 1 |  |
| 56473 | 1546020 | Perindopril erbumine 2mg tablets (Sigma Pharmaceuticals Plc) | Perindopril erbumine | 1 |  |
| 56472 | 1554020 | Perindopril erbumine 4mg tablets (Kent Pharmaceuticals Ltd) | Perindopril erbumine | 1 |  |
| 60065 | 1557020 | Perindopril erbumine 4mg tablets (Sigma Pharmaceuticals Plc) | Perindopril erbumine | 1 |  |
| 97 | 72573020 | Perindopril erbumine 4mg tablets | Perindopril erbumine | 1 |  |
| 38510 | 74776020 | Perindopril erbumine 4mg tablets (Apotex UK Ltd) | Perindopril erbumine | 1 |  |
| 57801 | 1558020 | Perindopril erbumine 4mg tablets (Glenmark Pharmaceuticals Europe Ltd) | Perindopril erbumine | 1 |  |
| 5612 | 72568020 | Coversyl 2mg tablets (Servier Laboratories Ltd) | Perindopril erbumine | 1 |  |
| 56516 | 75649020 | Perindopril erbumine 2mg tablets (Sandoz Ltd) | Perindopril erbumine | 1 |  |
| 6078 | 79311020 | Perindopril erbumine 8mg tablets | Perindopril erbumine | 1 |  |
| 49491 | 1545020 | Perindopril erbumine 2mg tablets (Consilient Health Ltd) | Perindopril erbumine | 1 |  |
| 57701 | 76377020 | Perindopril erbumine 8mg tablets (Actavis UK Ltd) | Perindopril erbumine | 1 |  |
| 68021 | 1555020 | Perindopril erbumine 4mg tablets (Alliance Healthcare (Distribution) Ltd) | Perindopril erbumine | 1 |  |
| 61693 | 15724021 | Perindopril erbumine 8mg tablets (Aurobindo Pharma Ltd) | Perindopril erbumine | 1 |  |
| 33095 | 73493020 | Perindopril erbumine 4mg tablets (A A H Pharmaceuticals Ltd) | Perindopril erbumine | 1 |  |
| 35731 | 73496020 | Perindopril erbumine 8mg tablets (A A H Pharmaceuticals Ltd) | Perindopril erbumine | 1 |  |
| 54733 | 9855020 | Perindopril erbumine 8mg tablets (Consilient Health Ltd) | Perindopril erbumine | 1 |  |
| 59790 | 39906020 | Perindopril erbumine 8mg tablets (Accord Healthcare Ltd) | Perindopril erbumine | 1 |  |
| 69016 | 53703021 | Perindopril erbumine 8mg tablets (DE Pharmaceuticals) | Perindopril erbumine | 1 |  |
| 54899 | 75033020 | Perindopril erbumine 2mg tablets (Teva UK Ltd) | Perindopril erbumine | 1 |  |
| 50402 | 74621020 | Perindopril 2mg Tablet (Servier Laboratories Ltd) | Perindopril erbumine | 1 |  |
| 48180 | 75652020 | Perindopril erbumine 4mg tablets (Sandoz Ltd) | Perindopril erbumine | 1 |  |
| 593 | 72572020 | Perindopril erbumine 2mg tablets | Perindopril erbumine | 1 |  |
| 54942 | 76559020 | Perindopril erbumine 8mg tablets (Mylan) | Perindopril erbumine | 1 |  |
| 64602 | 47141020 | Perindopril erbumine 2mg tablets (Waymade Healthcare Plc) | Perindopril erbumine | 1 |  |
| 43813 | 76373020 | Perindopril erbumine 2mg tablets (Actavis UK Ltd) | Perindopril erbumine | 1 |  |
| 67789 | 45150020 | Perindopril erbumine 2mg tablets (Accord Healthcare Ltd) | Perindopril erbumine | 1 |  |
| 70916 | 9854020 | Perindopril erbumine 8mg tablets (Glenmark Pharmaceuticals Europe Ltd) | Perindopril erbumine | 1 |  |
| 68381 | 57259021 | Perindopril erbumine 4mg tablets (Mawdsley-Brooks & Company Ltd) | Perindopril erbumine | 1 |  |
| 56508 | 1552020 | Coversyl 4mg tablets (Dowelhurst Ltd) | Perindopril erbumine | 1 |  |
| 56506 | 1541020 | Coversyl 2mg tablets (Dowelhurst Ltd) | Perindopril erbumine | 1 |  |
| 66060 | 53294021 | Perindopril erbumine 2mg tablets (DE Pharmaceuticals) | Perindopril erbumine | 1 |  |
| 45319 | 73490020 | Perindopril erbumine 2mg tablets (A A H Pharmaceuticals Ltd) | Perindopril erbumine | 1 |  |
| 61117 | 20667021 | Perindopril erbumine 4mg/5ml oral solution | Perindopril erbumine | 1 |  |
| 70917 | 1547020 | Perindopril erbumine 2mg tablets (Glenmark Pharmaceuticals Europe Ltd) | Perindopril erbumine | 1 |  |
| 38285 | 75036020 | Perindopril erbumine 4mg tablets (Teva UK Ltd) | Perindopril erbumine | 1 |  |
| 68759 | 15618021 | Perindopril erbumine 2mg tablets (Aurobindo Pharma Ltd) | Perindopril erbumine | 1 |  |
| 60067 | 22976021 | Perindopril erbumine 4mg / Amlodipine 5mg tablets | Perindopril erbumine/Amlodipine besilate | 0 | 1 |
| 60684 | 22974021 | Perindopril erbumine 4mg / Amlodipine 10mg tablets | Perindopril erbumine/Amlodipine besilate | 0 | 1 |
| 50607 | 92909020 | Perindopril arginine 2mg with Indapamide 625 micrograms tablet | Perindopril Erbumine/Indapamide | 0 | 1 |
| 48098 | 99501020 | Perindopril arginine 4mg with Indapamide 1.25mg tablet | Perindopril Erbumine/Indapamide | 0 | 1 |
| 57333 | 47942020 | Perindopril tosilate 5mg tablets | Perindopril tosilate | 1 |  |
| 56079 | 47933020 | Perindopril tosilate 10mg tablets | Perindopril tosilate | 1 |  |
| 57944 | 47936020 | Perindopril tosilate 2.5mg tablets | Perindopril tosilate | 1 |  |
| 15096 | 75604020 | Accupro 40mg tablets (Pfizer Ltd) | Quinapril hydrochloride | 1 |  |
| 14478 | 69845020 | Accupro 20mg tablets (Pfizer Ltd) | Quinapril hydrochloride | 1 |  |
| 6765 | 69848020 | Quinapril 5mg tablets | Quinapril hydrochloride | 1 |  |
| 7314 | 69843020 | Accupro 5mg tablets (Pfizer Ltd) | Quinapril hydrochloride | 1 |  |
| 61292 | 68165020 | Quinapril 40mg tablets (Mylan) | Quinapril hydrochloride | 1 |  |
| 14477 | 69844020 | Accupro 10mg tablets (Pfizer Ltd) | Quinapril hydrochloride | 1 |  |
| 3929 | 69849020 | Quinapril 10mg tablets | Quinapril hydrochloride | 1 |  |
| 46365 | 89048020 | Quinil 20mg tablets (Tillomed Laboratories Ltd) | Quinapril hydrochloride | 1 |  |
| 42285 | 89050020 | Quinil 40mg tablets (Tillomed Laboratories Ltd) | Quinapril hydrochloride | 1 |  |
| 38854 | 95370020 | Quinapril 20mg/5ml oral solution | Quinapril Hydrochloride | 1 |  |
| 5159 | 69850020 | Quinapril 20mg tablets | Quinapril hydrochloride | 1 |  |
| 40355 | 89044020 | Quinil 5mg tablets (Tillomed Laboratories Ltd) | Quinapril hydrochloride | 1 |  |
| 9731 | 75607020 | Quinapril 40mg tablets | Quinapril hydrochloride | 1 |  |
| 38899 | 89046020 | Quinil 10mg tablets (Tillomed Laboratories Ltd) | Quinapril hydrochloride | 1 |  |
| 28586 | 88107020 | Lopace 5mg capsules (Discovery Pharmaceuticals) | Ramipril | 1 |  |
| 52407 | 1585020 | Ramipril 10mg capsules (Kent Pharmaceuticals Ltd) | Ramipril | 1 |  |
| 709 | 72675020 | Ramipril 2.5mg capsules | Ramipril | 1 |  |
| 57658 | 67312020 | Ramipril 1.25mg tablets (A A H Pharmaceuticals Ltd) | Ramipril | 1 |  |
| 68372 | 73473020 | Ramipril 5mg tablets (Actavis UK Ltd) | Ramipril | 1 |  |
| 47998 | 68974020 | Ramipril 2.5mg capsules (Actavis UK Ltd) | Ramipril | 1 |  |
| 52399 | 1564020 | Ramipril 1.25mg capsules (Kent Pharmaceuticals Ltd) | Ramipril | 1 |  |
| 65936 | 53298021 | Ramipril 5mg capsules (DE Pharmaceuticals) | Ramipril | 1 |  |
| 38308 | 95107020 | Ramipril 2.5/5mg/10mg tablet | Ramipril | 1 |  |
| 34382 | 67206020 | Ramipril 5mg capsules (Zentiva) | Ramipril | 1 |  |
| 654 | 80174020 | Ramipril 2.5/5mg/10mg capsule | Ramipril | 1 |  |
| 45264 | 68971020 | Ramipril 1.25mg capsules (Actavis UK Ltd) | Ramipril | 1 |  |
| 34652 | 67455020 | Ramipril 5mg Capsule (Sovereign Medical Ltd) | Ramipril | 1 |  |
| 29627 | 88105020 | Lopace 2.5mg capsules (Discovery Pharmaceuticals) | Ramipril | 1 |  |
| 5275 | 72670020 | Tritace 2.5mg capsules (Sanofi) | Ramipril | 1 |  |
| 49164 | 1588020 | Ramipril 10mg capsules (Actavis UK Ltd) | Ramipril | 1 |  |
| 61694 | 67907020 | Ramipril 5mg tablets (Zentiva) | Ramipril | 1 |  |
| 11937 | 90539020 | Ramipril 2.5mg/5ml oral suspension | Ramipril | 1 |  |
| 56855 | 1590020 | Ramipril 10mg capsules (Sigma Pharmaceuticals Plc) | Ramipril | 1 |  |
| 61499 | 73468020 | Ramipril 2.5mg tablets (Actavis UK Ltd) | Ramipril | 1 |  |
| 9915 | 87063020 | Tritace 10mg tablets (Sanofi) | Ramipril | 1 |  |
| 34390 | 67186020 | Ramipril 5mg capsules (Genus Pharmaceuticals Ltd) | Ramipril | 1 |  |
| 34412 | 67140020 | Ramipril 5mg capsules (Teva UK Ltd) | Ramipril | 1 |  |
| 56129 | 1578020 | Ramipril 5mg capsules (Kent Pharmaceuticals Ltd) | Ramipril | 1 |  |
| 34657 | 67209020 | Ramipril 10mg capsules (Zentiva) | Ramipril | 1 |  |
| 42081 | 87446020 | Tritace 1.25mg Tablet (Sterwin Medicines) | Ramipril | 1 |  |
| 57073 | 47143020 | Ramipril 1.25mg capsules (Waymade Healthcare Plc) | Ramipril | 1 |  |
| 70709 | 10702020 | Ramipril 10mg tablets (Actavis UK Ltd) | Ramipril | 1 |  |
| 54620 | 1575020 | Ramipril 2.5mg capsules (Sigma Pharmaceuticals Plc) | Ramipril | 1 |  |
| 67741 | 73266020 | Ramipril 1.25mg capsules (Almus Pharmaceuticals Ltd) | Ramipril | 1 |  |
| 56148 | 10683020 | Ramipril 1.25mg tablets (Kent Pharmaceuticals Ltd) | Ramipril | 1 |  |
| 52197 | 1582020 | Ramipril 5mg capsules (Sigma Pharmaceuticals Plc) | Ramipril | 1 |  |
| 6364 | 87059020 | Tritace 2.5mg tablets (Sanofi) | Ramipril | 1 |  |
| 9646 | 72669020 | Tritace 1.25mg capsules (Aventis Pharma) | Ramipril | 1 |  |
| 55299 | 67297020 | Ramipril 1.25mg capsules (A A H Pharmaceuticals Ltd) | Ramipril | 1 |  |
| 82 | 70737020 | Ramipril 10mg capsules | Ramipril | 1 |  |
| 59603 | 1577020 | Ramipril 2.5mg capsules (Phoenix Healthcare Distribution Ltd) | Ramipril | 1 |  |
| 147 | 72674020 | Ramipril 1.25mg capsules | Ramipril | 1 |  |
| 66669 | 53299021 | Ramipril 10mg capsules (DE Pharmaceuticals) | Ramipril | 1 |  |
| 34732 | 67512020 | Ramipril 2.5mg Capsule (Dexcel-Pharma Ltd) | Ramipril | 1 |  |
| 34540 | 67305020 | Ramipril 5mg capsules (A A H Pharmaceuticals Ltd) | Ramipril | 1 |  |
| 68480 | 57263021 | Ramipril 10mg capsules (Mawdsley-Brooks & Company Ltd) | Ramipril | 1 |  |
| 50509 | 20723020 | Ramipril 10mg/5ml oral solution | Ramipril | 1 |  |
| 9693 | 55352020 | Tritace 10mg capsules (Sanofi) | Ramipril | 1 |  |
| 57235 | 74497020 | Ramipril 1.25mg tablets (Sandoz Ltd) | Ramipril | 1 |  |
| 70072 | 57260021 | Ramipril 1.25mg capsules (Mawdsley-Brooks & Company Ltd) | Ramipril | 1 |  |
| 80 | 72676020 | Ramipril 5mg capsules | Ramipril | 1 |  |
| 34431 | 67203020 | Ramipril 2.5mg capsules (Zentiva) | Ramipril | 1 |  |
| 55798 | 47145020 | Ramipril 5mg capsules (Waymade Healthcare Plc) | Ramipril | 1 |  |
| 45554 | 99318020 | Ramipril 5mg/5ml oral solution | Ramipril | 1 |  |
| 34429 | 69439020 | Ramipril 5mg capsules (Mylan) | Ramipril | 1 |  |
| 33811 | 69974020 | Ramipril 2.5mg capsules (Ranbaxy (UK) Ltd) | Ramipril | 1 |  |
| 64055 | 47865020 | Ramipril 2.5mg/5ml oral solution sugar free (Waymade Healthcare Plc) | Ramipril | 1 |  |
| 32857 | 67125020 | Ramipril 1.25mg capsules (Teva UK Ltd) | Ramipril | 1 |  |
| 66162 | 67147020 | Ramipril 10mg tablets (Teva UK Ltd) | Ramipril | 1 |  |
| 51701 | 1583020 | Ramipril 5mg capsules (Bristol Laboratories Ltd) | Ramipril | 1 |  |
| 54298 | 70068020 | Ramipril 2.5mg capsules (Arrow Generics Ltd) | Ramipril | 1 |  |
| 56763 | 1592020 | Ramipril 10mg capsules (Phoenix Healthcare Distribution Ltd) | Ramipril | 1 |  |
| 54941 | 1579020 | Ramipril 5mg capsules (Alliance Healthcare (Distribution) Ltd) | Ramipril | 1 |  |
| 34357 | 67189020 | Ramipril 10mg capsules (Genus Pharmaceuticals Ltd) | Ramipril | 1 |  |
| 45340 | 68982020 | Ramipril 10mg Capsule (Actavis UK Ltd) | Ramipril | 1 |  |
| 5735 | 72671020 | Tritace 5mg capsules (Sanofi) | Ramipril | 1 |  |
| 34893 | 68264020 | Ramipril 10mg Capsule (IVAX Pharmaceuticals UK Ltd) | Ramipril | 1 |  |
| 65443 | 67465020 | Ramipril 1.25mg Tablet (Sovereign Medical Ltd) | Ramipril | 1 |  |
| 51714 | 1572020 | Ramipril 2.5mg capsules (Alliance Healthcare (Distribution) Ltd) | Ramipril | 1 |  |
| 63442 | 67132020 | Ramipril 2.5mg tablets (Teva UK Ltd) | Ramipril | 1 |  |
| 71068 | 39004020 | Ramipril 2.5mg tablets (APC Pharmaceuticals & Chemicals (Europe) Ltd) | Ramipril | 1 |  |
| 40384 | 67322020 | Ramipril 10mg tablets (A A H Pharmaceuticals Ltd) | Ramipril | 1 |  |
| 53621 | 1576020 | Ramipril 2.5mg capsules (Bristol Laboratories Ltd) | Ramipril | 1 |  |
| 62039 | 67901020 | Ramipril 1.25mg tablets (Zentiva) | Ramipril | 1 |  |
| 56704 | 1565020 | Ramipril 1.25mg capsules (Alliance Healthcare (Distribution) Ltd) | Ramipril | 1 |  |
| 6288 | 87053020 | Ramipril 5mg tablets | Ramipril | 1 |  |
| 61339 | 73446020 | Ramipril 10mg capsules (Almus Pharmaceuticals Ltd) | Ramipril | 1 |  |
| 62918 | 39779020 | Ramipril 2.5mg/5ml oral solution | Ramipril | 1 |  |
| 34567 | 69436020 | Ramipril 2.5mg capsules (Mylan) | Ramipril | 1 |  |
| 34528 | 67301020 | Ramipril 2.5mg capsules (A A H Pharmaceuticals Ltd) | Ramipril | 1 |  |
| 34589 | 67515020 | Ramipril 5mg Capsule (Dexcel-Pharma Ltd) | Ramipril | 1 |  |
| 53612 | 10700020 | Ramipril 10mg tablets (Alliance Healthcare (Distribution) Ltd) | Ramipril | 1 |  |
| 47021 | 475021 | Ramipril 2.5mg/5ml oral solution sugar free | Ramipril | 1 |  |
| 71040 | 39748020 | Ramipril 5mg tablets (Pfizer Ltd) | Ramipril | 1 |  |
| 62036 | 47656020 | Ramipril 5mg tablets (Waymade Healthcare Plc) | Ramipril | 1 |  |
| 57864 | 10698020 | Ramipril 5mg tablets (Sigma Pharmaceuticals Plc) | Ramipril | 1 |  |
| 756 | 87055020 | Ramipril 10mg tablets | Ramipril | 1 |  |
| 34943 | 67309020 | Ramipril 10mg capsules (A A H Pharmaceuticals Ltd) | Ramipril | 1 |  |
| 61067 | 73443020 | Ramipril 5mg capsules (Almus Pharmaceuticals Ltd) | Ramipril | 1 |  |
| 56038 | 39749020 | Ramipril 10mg tablets (Pfizer Ltd) | Ramipril | 1 |  |
| 34432 | 67183020 | Ramipril 2.5mg capsules (Genus Pharmaceuticals Ltd) | Ramipril | 1 |  |
| 65599 | 67319020 | Ramipril 5mg tablets (A A H Pharmaceuticals Ltd) | Ramipril | 1 |  |
| 35007 | 91818020 | Ramipril 10mg/5ml oral suspension | Ramipril | 1 |  |
| 57346 | 47146020 | Ramipril 10mg capsules (Waymade Healthcare Plc) | Ramipril | 1 |  |
| 34877 | 67460020 | Ramipril 10mg Capsule (Sovereign Medical Ltd) | Ramipril | 1 |  |
| 69288 | 66003021 | Ramipril 10mg capsules (Brown & Burk UK Ltd) | Ramipril | 1 |  |
| 34539 | 67175020 | Ramipril 5mg capsules (Sandoz Ltd) | Ramipril | 1 |  |
| 34698 | 67770020 | Ramipril 1.25mg capsules (Zentiva) | Ramipril | 1 |  |
| 34583 | 67518020 | Ramipril 10mg Capsule (Dexcel-Pharma Ltd) | Ramipril | 1 |  |
| 34490 | 67129020 | Ramipril 2.5mg capsules (Teva UK Ltd) | Ramipril | 1 |  |
| 63010 | 10706020 | Ramipril 10mg tablets (Phoenix Healthcare Distribution Ltd) | Ramipril | 1 |  |
| 34505 | 67170020 | Ramipril 2.5mg capsules (Sandoz Ltd) | Ramipril | 1 |  |
| 46890 | 477021 | Ramipril 5mg/5ml oral suspension | Ramipril | 1 |  |
| 61985 | 67121020 | Ramipril 1.25mg tablets (Teva UK Ltd) | Ramipril | 1 |  |
| 68192 | 66002021 | Ramipril 5mg capsules (Brown & Burk UK Ltd) | Ramipril | 1 |  |
| 32934 | 88109020 | Lopace 10mg capsules (Discovery Pharmaceuticals) | Ramipril | 1 |  |
| 33894 | 67144020 | Ramipril 10mg capsules (Teva UK Ltd) | Ramipril | 1 |  |
| 761 | 87049020 | Ramipril 1.25mg tablets | Ramipril | 1 |  |
| 34651 | 69441020 | Ramipril 10mg capsules (Mylan) | Ramipril | 1 |  |
| 59557 | 1571020 | Ramipril 2.5mg capsules (Kent Pharmaceuticals Ltd) | Ramipril | 1 |  |
| 56013 | 47144020 | Ramipril 2.5mg capsules (Waymade Healthcare Plc) | Ramipril | 1 |  |
| 62958 | 67136020 | Ramipril 5mg tablets (Teva UK Ltd) | Ramipril | 1 |  |
| 48008 | 68978020 | Ramipril 5mg capsules (Actavis UK Ltd) | Ramipril | 1 |  |
| 56356 | 1586020 | Ramipril 10mg capsules (Alliance Healthcare (Distribution) Ltd) | Ramipril | 1 |  |
| 59788 | 1591020 | Ramipril 10mg capsules (Bristol Laboratories Ltd) | Ramipril | 1 |  |
| 71025 | 39003020 | Ramipril 1.25mg tablets (APC Pharmaceuticals & Chemicals (Europe) Ltd) | Ramipril | 1 |  |
| 48053 | 73269020 | Ramipril 2.5mg capsules (Almus Pharmaceuticals Ltd) | Ramipril | 1 |  |
| 67719 | 57262021 | Ramipril 5mg capsules (Mawdsley-Brooks & Company Ltd) | Ramipril | 1 |  |
| 60730 | 1584020 | Ramipril 5mg capsules (Phoenix Healthcare Distribution Ltd) | Ramipril | 1 |  |
| 39355 | 87452020 | Tritace 10mg Tablet (Sterwin Medicines) | Ramipril | 1 |  |
| 65749 | 44888021 | Ramipril 5mg capsules (Ennogen Pharma Ltd) | Ramipril | 1 |  |
| 6362 | 87061020 | Tritace 5mg tablets (Sanofi) | Ramipril | 1 |  |
| 6261 | 87057020 | Tritace 1.25mg tablets (Sanofi) | Ramipril | 1 |  |
| 6314 | 87051020 | Ramipril 2.5mg tablets | Ramipril | 1 |  |
| 34710 | 67179020 | Ramipril 10mg capsules (Sandoz Ltd) | Ramipril | 1 |  |
| 66329 | 91820020 | Ramipril oral solution | Ramipril | 1 |  |
| 56169 | 70076020 | Ramipril 10mg capsules (Arrow Generics Ltd) | Ramipril | 1 |  |
| 31810 | 68138020 | Odrik 1mg capsules (Aventis Pharma) | Trandolapril | 1 |  |
| 60757 | 75136020 | Trandolapril 500microgram capsules (Teva UK Ltd) | Trandolapril | 1 |  |
| 7419 | 69080020 | Trandolapril 500microgram capsules | Trandolapril | 1 |  |
| 16710 | 71438020 | Gopten 500microgram capsules (Abbott Laboratories Ltd) | Trandolapril | 1 |  |
| 29130 | 87608020 | Gopten 4mg capsules (Abbott Laboratories Ltd) | Trandolapril | 1 |  |
| 4103 | 69081020 | Trandolapril 1mg capsules | Trandolapril | 1 |  |
| 65389 | 1616020 | Gopten 500microgram capsules (Waymade Healthcare Plc) | Trandolapril | 1 |  |
| 9948 | 87606020 | Trandolapril 4mg capsules | Trandolapril | 1 |  |
| 54345 | 10867020 | Trandolapril 4mg capsules (Arrow Generics Ltd) | Trandolapril | 1 |  |
| 5047 | 69082020 | Trandolapril 2mg capsules | Trandolapril | 1 |  |
| 8025 | 71439020 | Gopten 1mg capsules (Abbott Laboratories Ltd) | Trandolapril | 1 |  |
| 28902 | 68139020 | Odrik 2mg capsules (Aventis Pharma) | Trandolapril | 1 |  |
| 65570 | 75295020 | Trandolapril 4mg capsules (Teva UK Ltd) | Trandolapril | 1 |  |
| 8026 | 71440020 | Gopten 2mg capsules (Abbott Laboratories Ltd) | Trandolapril | 1 |  |
| 66623 | 75170020 | Trandolapril 2mg capsules (A A H Pharmaceuticals Ltd) | Trandolapril | 1 |  |
| 31307 | 68137020 | Odrik 500microgram capsules (Aventis Pharma) | Trandolapril | 1 |  |
| 19690 | 85865020 | Verapamil 180mg modified-release / Trandolapril 2mg capsules | Verapamil hydrochloride/Trandolapril | 0 | 1 |
| 18223 | 85883020 | Trandolapril with verapamil 2mg + 180mg Modified-release capsule | Verapamil Hydrochloride/Trandolapril | 0 | 1 |
| 20579 | 85903020 | Tarka modified-release capsules (Abbott Laboratories Ltd) | Verapamil hydrochloride/Trandolapril | 0 | 1 |

ACEi, angiotensin-converting enzyme inhibitor

**Supplementary Table 8. Code list for ARB**

| **ARB** | | |  |  |  | | |  |  |
| --- | --- | --- | --- | --- | --- | --- | --- | --- | --- |
| **Prod**  **code** | | | **Gemscript code** | **Product**  **name** | **Drug**  **Substance**  **name** | | | **to_include** | **combination therapy to exclude** |
| 47616 | | | 99785020 | Sevikar HCT 40mg/10mg/12.5mg tablets (Daiichi Sankyo UK Ltd) | Amlodipine besilate/Hydrochlorothiazide/Olmesartan medoxomil | | | 0 | 1 |
| 47573 | | | 99783020 | Sevikar HCT 40mg/5mg/12.5mg tablets (Daiichi Sankyo UK Ltd) | Amlodipine besilate/Hydrochlorothiazide/Olmesartan medoxomil | | | 0 | 1 |
| 60007 | | | 38012020 | Generic Sevikar HCT 40mg/10mg/12.5mg tablets | Amlodipine besilate/Hydrochlorothiazide/Olmesartan medoxomil | | | 0 | 1 |
| 35304 | | | 92957020 | Valsartan 160mg with amlodipine 10mg tablets | Amlodipine Besilate/Valsartan | | | 0 | 1 |
| 35189 | | | 92767020 | Amlodipine 10mg / Valsartan 160mg tablets | Amlodipine besilate/Valsartan | | | 0 | 1 |
| 35096 | | | 92773020 | Exforge 10mg/160mg tablets (Novartis Pharmaceuticals UK Ltd) | Amlodipine besilate/Valsartan | | | 0 | 1 |
| 35329 | | | 92763020 | Amlodipine 5mg / Valsartan 80mg tablets | Amlodipine besilate/Valsartan | | | 0 | 1 |
| 35173 | | | 92953020 | Valsartan 160mg with amlodipine 5mg tablets | Amlodipine Besilate/Valsartan | | | 0 | 1 |
| 35317 | | | 92769020 | Exforge 5mg/80mg tablets (Novartis Pharmaceuticals UK Ltd) | Amlodipine besilate/Valsartan | | | 0 | 1 |
| 35174 | | | 92951020 | Valsartan 80mg with amlodipine 5mg tablets | Amlodipine Besilate/Valsartan | | | 0 | 1 |
| 55358 | | | 99777020 | Olmesartan medoxomil with amlodipine and hydrochlorothiazide 40mg + 10mg + 25mg Tablet | Amlodipine/Hydrochlorothiazide/Olmesartan Medoxomil | | | 0 | 1 |
| 46687 | | | 99769020 | Olmesartan medoxomil with amlodipine and hydrochlorothiazide 20mg + 5mg + 12.5mg Tablet | Amlodipine/Hydrochlorothiazide/Olmesartan Medoxomil | | | 0 | 1 |
| 46792 | | | 99771020 | Olmesartan medoxomil with amlodipine and hydrochlorothiazide 40mg + 5mg + 12.5mg Tablet | Amlodipine/Hydrochlorothiazide/Olmesartan Medoxomil | | | 0 | 1 |
| 46715 | | | 99773020 | Olmesartan medoxomil with amlodipine and hydrochlorothiazide 40mg + 10mg + 12.5mg Tablet | Amlodipine/Hydrochlorothiazide/Olmesartan Medoxomil | | | 0 | 1 |
| 47467 | | | 99775020 | Olmesartan medoxomil with amlodipine and hydrochlorothiazide 40mg + 5mg + 25mg Tablet | Amlodipine/Hydrochlorothiazide/Olmesartan Medoxomil | | | 0 | 1 |
| 56606 | | | 41454020 | Azilsartan medoxomil 40mg tablets | Azilsartan medoxomil | | | 1 |  |
| 51897 | | | 41680020 | Edarbi 20mg tablets (Takeda UK Ltd) | Azilsartan medoxomil | | | 1 |  |
| 51368 | | | 41456020 | Azilsartan medoxomil 80mg tablets | Azilsartan medoxomil | | | 1 |  |
| 531 | | | 81324020 | Candesartan 4mg tablets | Candesartan cilexetil | | | 1 |  |
| 54414 | | | 41324020 | Candesartan 16mg tablets (Consilient Health Ltd) | Candesartan cilexetil | | | 1 |  |
| 67929 | | | 66007021 | Candesartan 16mg tablets (Tillomed Laboratories Ltd) | Candesartan cilexetil | | | 1 |  |
| 57026 | | | 47162020 | Candesartan 8mg tablets (Waymade Healthcare Plc) | Candesartan cilexetil | | | 1 |  |
| 529 | | | 81323020 | Candesartan 2mg tablets | Candesartan cilexetil | | | 1 |  |
| 4685 | | | 79054020 | Amias 4mg tablets (Takeda UK Ltd) | Candesartan cilexetil | | | 1 |  |
| 31072 | | | 88790020 | Amias 32mg tablets (Takeda UK Ltd) | Candesartan cilexetil | | | 1 |  |
| 51117 | | | 1771020 | Candesartan 8mg tablets (DE Pharmaceuticals) | Candesartan cilexetil | | | 1 |  |
| 51647 | | | 1764020 | Candesartan 4mg tablets (Mawdsley-Brooks & Company Ltd) | Candesartan cilexetil | | | 1 |  |
| 5013 | | | 79055020 | Amias 8mg tablets (Takeda UK Ltd) | Candesartan cilexetil | | | 1 |  |
| 68718 | | | 41312020 | Candesartan 4mg tablets (A A H Pharmaceuticals Ltd) | Candesartan cilexetil | | | 1 |  |
| 66958 | | | 25451021 | Candesartan 8mg tablets (Sandoz Ltd) | Candesartan cilexetil | | | 1 |  |
| 5117 | | | 86113020 | Amias 16mg tablets (Takeda UK Ltd) | Candesartan cilexetil | | | 1 |  |
| 57977 | | | 41323020 | Candesartan 16mg tablets (Alliance Healthcare (Distribution) Ltd) | Candesartan cilexetil | | | 1 |  |
| 70805 | | | 73863021 | Candesartan 8mg tablets (Crescent Pharma Ltd) | Candesartan cilexetil | | | 1 |  |
| 69802 | | | 70618021 | Candesartan 4mg tablets (Mylan) | Candesartan cilexetil | | | 1 |  |
| 68647 | | | 69013021 | Candesartan 8mg tablets (Genesis Pharmaceuticals Ltd) | Candesartan cilexetil | | | 1 |  |
| 65228 | | | 1781020 | Candesartan 16mg tablets (Mawdsley-Brooks & Company Ltd) | Candesartan cilexetil | | | 1 |  |
| 64359 | | | 16671021 | Candesartan 4mg tablets (DE Pharmaceuticals) | Candesartan cilexetil | | | 1 |  |
| 57266 | | | 46326020 | Candesartan 2mg tablets (Actavis UK Ltd) | Candesartan cilexetil | | | 1 |  |
| 4155 | | | 79053020 | Amias 2mg tablets (Takeda UK Ltd) | Candesartan cilexetil | | | 1 |  |
| 4818 | | | 81325020 | Candesartan 8mg tablets | Candesartan cilexetil | | | 1 |  |
| 71080 | | | 42155020 | Candesartan 4mg tablets (Zentiva) | Candesartan cilexetil | | | 1 |  |
| 54326 | | | 41435020 | Candesartan 32mg tablets (Teva UK Ltd) | Candesartan cilexetil | | | 1 |  |
| 53680 | | | 41320020 | Candesartan 16mg tablets (Teva UK Ltd) | Candesartan cilexetil | | | 1 |  |
| 59690 | | | 41319020 | Candesartan 8mg tablets (Consilient Health Ltd) | Candesartan cilexetil | | | 1 |  |
| 65479 | | | 41308020 | Candesartan 2mg tablets (A A H Pharmaceuticals Ltd) | Candesartan cilexetil | | | 1 |  |
| 50185 | | | 41315020 | Candesartan 8mg tablets (Teva UK Ltd) | Candesartan cilexetil | | | 1 |  |
| 7043 | | | 88788020 | Candesartan 32mg tablets | Candesartan cilexetil | | | 1 |  |
| 53755 | | | 41310020 | Candesartan 4mg tablets (Teva UK Ltd) | Candesartan cilexetil | | | 1 |  |
| 59802 | | | 41901020 | Candesartan 2mg tablets (Teva UK Ltd) | Candesartan cilexetil | | | 1 |  |
| 68751 | | | 69014021 | Candesartan 16mg tablets (Genesis Pharmaceuticals Ltd) | Candesartan cilexetil | | | 1 |  |
| 62035 | | | 47163020 | Candesartan 16mg tablets (Waymade Healthcare Plc) | Candesartan cilexetil | | | 1 |  |
| 52208 | | | 41322020 | Candesartan 16mg tablets (A A H Pharmaceuticals Ltd) | Candesartan cilexetil | | | 1 |  |
| 57273 | | | 41316020 | Candesartan 8mg tablets (Actavis UK Ltd) | Candesartan cilexetil | | | 1 |  |
| 51519 | | | 41317020 | Candesartan 8mg tablets (A A H Pharmaceuticals Ltd) | Candesartan cilexetil | | | 1 |  |
| 4741 | | | 81321020 | Candesartan 16mg tablets | Candesartan cilexetil | | | 1 |  |
| 62140 | | | 25450021 | Candesartan 4mg tablets (Sandoz Ltd) | Candesartan cilexetil | | | 1 |  |
| 58646 | | | 41311020 | Candesartan 4mg tablets (Actavis UK Ltd) | Candesartan cilexetil | | | 1 |  |
| 52559 | | | 42156020 | Candesartan 8mg tablets (Zentiva) | Candesartan cilexetil | | | 1 |  |
| 66624 | | | 25452021 | Candesartan 16mg tablets (Sandoz Ltd) | Candesartan cilexetil | | | 1 |  |
| 70455 | | | 36604020 | Candesartan 4mg/5ml oral suspension | Candesartan cilexetil | | | 1 |  |
| 16371 | | | 84006020 | Teveten 600mg tablets (Mylan) | Eprosartan mesilate | | | 1 |  |
| 12836 | | | 84002020 | Eprosartan 600mg tablets | Eprosartan mesilate | | | 1 |  |
| 63337 | | | 44389020 | Eprosartan 600mg tablets (A A H Pharmaceuticals Ltd) | Eprosartan mesilate | | | 1 |  |
| 9745 | | | 84004020 | Teveten 300mg tablets (Mylan) | Eprosartan mesilate | | | 1 |  |
| 13123 | | | 84001020 | Eprosartan 400mg tablets | Eprosartan mesilate | | | 1 |  |
| 6939 | | | 84000020 | Eprosartan 300mg tablets | Eprosartan mesilate | | | 1 |  |
| 16285 | | | 84005020 | Teveten 400mg tablets (Abbott Healthcare Products Ltd) | Eprosartan mesilate | | | 1 |  |
| 47727 | | | 99789020 | Sevikar HCT 40mg/5mg/25mg tablets (Daiichi Sankyo UK Ltd) | Hydrochlorothiazide/Amlodipine besilate/Olmesartan medoxomil | | | 0 | 1 |
| 53220 | | | 99791020 | Sevikar HCT 40mg/10mg/25mg tablets (Daiichi Sankyo UK Ltd) | Hydrochlorothiazide/Amlodipine besilate/Olmesartan medoxomil | | | 0 | 1 |
| 35196 | | | 92367020 | CoAprovel 300mg/25mg tablets (Sanofi) | Hydrochlorothiazide/Irbesartan | | | 0 | 1 |
| 35481 | | | 92365020 | Irbesartan 300mg / Hydrochlorothiazide 25mg tablets | Hydrochlorothiazide/Irbesartan | | | 0 | 1 |
| 37650 | | | 94425020 | Losartan 100mg / Hydrochlorothiazide 12.5mg tablets | Hydrochlorothiazide/Losartan potassium | | | 0 | 1 |
| 48039 | | | 78259020 | Losartan 100mg / Hydrochlorothiazide 12.5mg tablets (Teva UK Ltd) | Hydrochlorothiazide/Losartan potassium | | | 0 | 1 |
| 37747 | | | 94429020 | Cozaar-Comp 100mg/12.5mg tablets (Merck Sharp & Dohme Ltd) | Hydrochlorothiazide/Losartan potassium | | | 0 | 1 |
| 70922 | | | 34060020 | Losartan 100mg / Hydrochlorothiazide 12.5mg tablets (Phoenix Healthcare Distribution Ltd) | Hydrochlorothiazide/Losartan potassium | | | 0 | 1 |
| 18903 | | | 90631020 | Olmesartan medoxomil 20mg / Hydrochlorothiazide 25mg tablets | Hydrochlorothiazide/Olmesartan medoxomil | | | 0 | 1 |
| 39021 | | | 90733020 | Hydrochlorothiazide with olmesartan medoxomil 25mg with 20mg tablet | Hydrochlorothiazide/Olmesartan Medoxomil | | | 0 | 1 |
| 35380 | | | 90735020 | Hydrochlorothiazide with olmesartan medoxomil 12.5mg with 20mg tablet | Hydrochlorothiazide/Olmesartan Medoxomil | | | 0 | 1 |
| 27520 | | | 90637020 | Olmetec Plus 20mg/25mg tablets (Daiichi Sankyo UK Ltd) | Hydrochlorothiazide/Olmesartan medoxomil | | | 0 | 1 |
| 43915 | | | 98339020 | Olmetec Plus 40mg/12.5mg tablets (Daiichi Sankyo UK Ltd) | Hydrochlorothiazide/Olmesartan medoxomil | | | 0 | 1 |
| 43322 | | | 98337020 | Olmesartan medoxomil 40mg / Hydrochlorothiazide 12.5mg tablets | Hydrochlorothiazide/Olmesartan medoxomil | | | 0 | 1 |
| 60780 | | | 38011020 | Generic Sevikar HCT 20mg/5mg/12.5mg tablets | Hydrochlorothiazide/Olmesartan medoxomil/Amlodipine besilate | | | 0 | 1 |
| 46355 | | | 99779020 | Sevikar HCT 20mg/5mg/12.5mg tablets (Daiichi Sankyo UK Ltd) | Hydrochlorothiazide/Olmesartan medoxomil/Amlodipine besilate | | | 0 | 1 |
| 38889 | | | 95336020 | MicardisPlus 80mg/25mg tablets (Boehringer Ingelheim Ltd) | Hydrochlorothiazide/Telmisartan | | | 0 | 1 |
| 38459 | | | 95334020 | Telmisartan 80mg / Hydrochlorothiazide 25mg tablets | Hydrochlorothiazide/Telmisartan | | | 0 | 1 |
| 16060 | | | 88420020 | Valsartan 80mg / Hydrochlorothiazide 12.5mg tablets | Hydrochlorothiazide/Valsartan | | | 0 | 1 |
| 52858 | | | 10088020 | Co-Diovan 80mg/12.5mg tablets (Sigma Pharmaceuticals Plc) | Hydrochlorothiazide/Valsartan | | | 0 | 1 |
| 764 | | | 88424020 | Co-Diovan 80mg/12.5mg tablets (Novartis Pharmaceuticals UK Ltd) | Hydrochlorothiazide/Valsartan | | | 0 | 1 |
| 70955 | | | 74725020 | Irbesartan 300mg/5ml Oral suspension (Martindale Pharmaceuticals Ltd) | Irbesartan | | | 1 |  |
| 7338 | | | 85709020 | Aprovel 75mg tablets (Sanofi) | Irbesartan | | | 1 |  |
| 63411 | | | 43824020 | Irbesartan 300mg tablets (Alliance Healthcare (Distribution) Ltd) | Irbesartan | | | 1 |  |
| 58201 | | | 44381020 | Irbesartan 150mg tablets (Actavis UK Ltd) | Irbesartan | | | 1 |  |
| 52972 | | | 1748020 | Irbesartan 300mg tablets (Sigma Pharmaceuticals Plc) | Irbesartan | | | 1 |  |
| 65065 | | | 44380020 | Irbesartan 75mg tablets (A A H Pharmaceuticals Ltd) | Irbesartan | | | 1 |  |
| 58108 | | | 44383020 | Irbesartan 150mg tablets (A A H Pharmaceuticals Ltd) | Irbesartan | | | 1 |  |
| 36939 | | | 94158020 | Irbesartan 300mg/5ml oral suspension | Irbesartan | | | 1 |  |
| 59393 | | | 47159020 | Irbesartan 300mg tablets (Sandoz Ltd) | Irbesartan | | | 1 |  |
| 1293 | | | 85706020 | Irbesartan 150mg tablets | Irbesartan | | | 1 |  |
| 71096 | | | 46324020 | Irbesartan 150mg tablets (Dr Reddy's Laboratories (UK) Ltd) | Irbesartan | | | 1 |  |
| 55017 | | | 45155020 | Irbesartan 300mg tablets (Accord Healthcare Ltd) | Irbesartan | | | 1 |  |
| 63385 | | | 45450020 | Sabervel 75mg tablets (Aspire Pharma Ltd) | Irbesartan | | | 1 |  |
| 60597 | | | 43819020 | Irbesartan 150mg tablets (Teva UK Ltd) | Irbesartan | | | 1 |  |
| 70431 | | | 31321021 | Irbesartan 150mg tablets (Lupin (Europe) Ltd) | Irbesartan | | | 1 |  |
| 61781 | | | 43823020 | Irbesartan 300mg tablets (Teva UK Ltd) | Irbesartan | | | 1 |  |
| 63717 | | | 23039021 | Irbesartan 300mg tablets (DE Pharmaceuticals) | Irbesartan | | | 1 |  |
| 62415 | | | 43822020 | Irbesartan 300mg tablets (A A H Pharmaceuticals Ltd) | Irbesartan | | | 1 |  |
| 2971 | | | 85707020 | Irbesartan 300mg tablets | Irbesartan | | | 1 |  |
| 71019 | | | 46323020 | Irbesartan 75mg tablets (Dr Reddy's Laboratories (UK) Ltd) | Irbesartan | | | 1 |  |
| 9196 | | | 85710020 | Aprovel 150mg tablets (Sanofi) | Irbesartan | | | 1 |  |
| 828 | | | 85705020 | Irbesartan 75mg tablets | Irbesartan | | | 1 |  |
| 11348 | | | 85711020 | Aprovel 300mg tablets (Sanofi) | Irbesartan | | | 1 |  |
| 11526 | | | 83982020 | CoAprovel 300mg/12.5mg tablets (Sanofi) | Irbesartan/Hydrochlorothiazide | | | 0 | 1 |
| 11469 | | | 80125020 | Irbesartan 300mg / Hydrochlorothiazide 12.5mg tablets | Irbesartan/Hydrochlorothiazide | | | 0 | 1 |
| 11448 | | | 80124020 | Irbesartan 150mg / Hydrochlorothiazide 12.5mg tablets | Irbesartan/Hydrochlorothiazide | | | 0 | 1 |
| 62337 | | | 20118021 | Irbesartan 300mg / Hydrochlorothiazide 12.5mg tablets (Actavis UK Ltd) | Irbesartan/Hydrochlorothiazide | | | 0 | 1 |
| 10316 | | | 83981020 | CoAprovel 150mg/12.5mg tablets (Sanofi) | Irbesartan/Hydrochlorothiazide | | | 0 | 1 |
| 41232 | | | 97245020 | Cozaar 2.5mg/ml oral suspension (Merck Sharp & Dohme Ltd) | Losartan potassium | | | 1 |  |
| 67902 | | | 40623020 | Losartan 100mg tablets (Sandoz Ltd) | Losartan potassium | | | 1 |  |
| 58274 | | | 1646020 | Losartan 25mg tablets (Accord Healthcare Ltd) | Losartan potassium | | | 1 |  |
| 58649 | | | 43813020 | Losartan 25mg tablets (Bristol Laboratories Ltd) | Losartan potassium | | | 1 |  |
| 54843 | | | 77349020 | Losartan 50mg tablets (Dexcel-Pharma Ltd) | Losartan potassium | | | 1 |  |
| 51601 | | | 77734020 | Losartan 50mg tablets (Actavis UK Ltd) | Losartan potassium | | | 1 |  |
| 61495 | | | 14777021 | Losartan 25mg tablets (Aptil Pharma Ltd) | Losartan potassium | | | 1 |  |
| 54057 | | | 77299020 | Losartan 50mg tablets (Teva UK Ltd) | Losartan potassium | | | 1 |  |
| 52658 | | | 36312020 | Losartan 100mg/5ml oral suspension | Losartan potassium | | | 1 |  |
| 65094 | | | 40516020 | Losartan 50mg tablets (Sandoz Ltd) | Losartan potassium | | | 1 |  |
| 52659 | | | 35425020 | Losartan 50mg/5ml oral solution | Losartan potassium | | | 1 |  |
| 68603 | | | 41900020 | Losartan 50mg tablets (Wockhardt UK Ltd) | Losartan potassium | | | 1 |  |
| 54404 | | | 77737020 | Losartan 100mg tablets (Actavis UK Ltd) | Losartan potassium | | | 1 |  |
| 69667 | | | 36310020 | Losartan 100mg/5ml oral solution | Losartan potassium | | | 1 |  |
| 59271 | | | 40515020 | Losartan 25mg tablets (Sandoz Ltd) | Losartan potassium | | | 1 |  |
| 40711 | | | 97243020 | Losartan 2.5mg/ml oral suspension sugar free | Losartan potassium | | | 1 |  |
| 55296 | | | 1660020 | Losartan 50mg tablets (Mylan) | Losartan potassium | | | 1 |  |
| 61288 | | | 10519020 | Losartan 100mg tablets (Accord Healthcare Ltd) | Losartan potassium | | | 1 |  |
| 59903 | | | 35427020 | Losartan 50mg/5ml oral suspension | Losartan potassium | | | 1 |  |
| 55718 | | | 1644020 | Losartan 25mg tablets (Phoenix Healthcare Distribution Ltd) | Losartan potassium | | | 1 |  |
| 54049 | | | 1669020 | Losartan 50mg tablets (Accord Healthcare Ltd) | Losartan potassium | | | 1 |  |
| 1780 | | | 76332020 | Losartan 50mg tablets | Losartan potassium | | | 1 |  |
| 14965 | | | 80730020 | Cozaar 100mg tablets (Merck Sharp & Dohme Ltd) | Losartan potassium | | | 1 |  |
| 56104 | | | 1658020 | Losartan 50mg tablets (A A H Pharmaceuticals Ltd) | Losartan potassium | | | 1 |  |
| 69858 | | | 1642020 | Losartan 25mg tablets (Alliance Healthcare (Distribution) Ltd) | Losartan potassium | | | 1 |  |
| 54735 | | | 1661020 | Losartan 50mg tablets (Alliance Healthcare (Distribution) Ltd) | Losartan potassium | | | 1 |  |
| 62388 | | | 24026021 | Losartan 12.5mg tablets (DE Pharmaceuticals) | Losartan potassium | | | 1 |  |
| 54740 | | | 77731020 | Losartan 25mg tablets (Actavis UK Ltd) | Losartan potassium | | | 1 |  |
| 58967 | | | 35999020 | Losartan 12.5mg tablets (Alliance Healthcare (Distribution) Ltd) | Losartan potassium | | | 1 |  |
| 50971 | | | 1640020 | Losartan 25mg tablets (A A H Pharmaceuticals Ltd) | Losartan potassium | | | 1 |  |
| 60506 | | | 77323020 | Losartan 100mg tablets (Dexcel-Pharma Ltd) | Losartan potassium | | | 1 |  |
| 55446 | | | 43882020 | Losartan 100mg tablets (Bristol Laboratories Ltd) | Losartan potassium | | | 1 |  |
| 68340 | | | 68779021 | Losartan 12.5mg tablets (Consilient Health Ltd) | Losartan potassium | | | 1 |  |
| 63222 | | | 1645020 | Losartan 25mg tablets (Pfizer Ltd) | Losartan potassium | | | 1 |  |
| 59340 | | | 77317020 | Losartan 12.5mg tablets (Dexcel-Pharma Ltd) | Losartan potassium | | | 1 |  |
| 64888 | | | 53033021 | Losartan 12.5mg tablets (Sigma Pharmaceuticals Plc) | Losartan potassium | | | 1 |  |
| 39944 | | | 96963020 | Losartan 12.5mg tablets | Losartan potassium | | | 1 |  |
| 66551 | | | 43814020 | Losartan 50mg tablets (Bristol Laboratories Ltd) | Losartan potassium | | | 1 |  |
| 624 | | | 79160020 | Losartan 100mg tablets | Losartan potassium | | | 1 |  |
| 4226 | | | 76329020 | Cozaar 25mg tablets (Merck Sharp & Dohme Ltd) | Losartan potassium | | | 1 |  |
| 48398 | | | 77320020 | Losartan 25mg tablets (Dexcel-Pharma Ltd) | Losartan potassium | | | 1 |  |
| 59086 | | | 41899020 | Losartan 25mg tablets (Wockhardt UK Ltd) | Losartan potassium | | | 1 |  |
| 51186 | | | 1643020 | Losartan 25mg tablets (Arrow Generics Ltd) | Losartan potassium | | | 1 |  |
| 66114 | | | 60052021 | Losartan 12.5mg tablets (Mawdsley-Brooks & Company Ltd) | Losartan potassium | | | 1 |  |
| 57028 | | | 10512020 | Losartan 100mg tablets (Mylan) | Losartan potassium | | | 1 |  |
| 520 | | | 76333020 | Losartan 25mg tablets | Losartan potassium | | | 1 |  |
| 59750 | | | 14778021 | Losartan 50mg tablets (Aptil Pharma Ltd) | Losartan potassium | | | 1 |  |
| 70765 | | | 68395021 | Losartan 100mg tablets (Almus Pharmaceuticals Ltd) | Losartan potassium | | | 1 |  |
| 52886 | | | 35998020 | Losartan 12.5mg tablets (A A H Pharmaceuticals Ltd) | Losartan potassium | | | 1 |  |
| 49492 | | | 1641020 | Losartan 25mg tablets (Mylan) | Losartan potassium | | | 1 |  |
| 63918 | | | 77296020 | Losartan 25mg tablets (Teva UK Ltd) | Losartan potassium | | | 1 |  |
| 61053 | | | 10513020 | Losartan 100mg tablets (Alliance Healthcare (Distribution) Ltd) | Losartan potassium | | | 1 |  |
| 47006 | | | 77302020 | Losartan 100mg tablets (Teva UK Ltd) | Losartan potassium | | | 1 |  |
| 61754 | | | 35460020 | Losartan 25mg/5ml oral suspension | Losartan potassium | | | 1 |  |
| 59351 | | | 1666020 | Losartan 50mg tablets (Pfizer Ltd) | Losartan potassium | | | 1 |  |
| 40571 | | | 96965020 | Cozaar 12.5mg tablets (Merck Sharp & Dohme Ltd) | Losartan potassium | | | 1 |  |
| 70325 | | | 68366021 | Losartan 50mg tablets (Almus Pharmaceuticals Ltd) | Losartan potassium | | | 1 |  |
| 5723 | | | 76328020 | Cozaar 50mg tablets (Merck Sharp & Dohme Ltd) | Losartan potassium | | | 1 |  |
| 49588 | | | 10510020 | Losartan 100mg tablets (A A H Pharmaceuticals Ltd) | Losartan potassium | | | 1 |  |
| 56970 | | | 10517020 | Losartan 100mg tablets (Pfizer Ltd) | Losartan potassium | | | 1 |  |
| 52427 | | | 10518020 | Cozaar 100mg tablets (Necessity Supplies Ltd) | Losartan potassium | | | 1 |  |
| 21423 | | | 89783020 | Cozaar-Comp 100mg/25mg tablets (Merck Sharp & Dohme Ltd) | Losartan potassium/Hydrochlorothiazide | | | 0 | 1 |
| 52189 | | | 10152020 | Losartan 100mg / Hydrochlorothiazide 25mg tablets (A A H Pharmaceuticals Ltd) | Losartan potassium/Hydrochlorothiazide | | | 0 | 1 |
| 24632 | | | 89781020 | Hydrochlorothiazide with losartan 25mg with 100mg Tablet | Losartan Potassium/Hydrochlorothiazide | | | 0 | 1 |
| 6437 | | | 79786020 | Losartan 50mg / Hydrochlorothiazide 12.5mg tablets | Losartan potassium/Hydrochlorothiazide | | | 0 | 1 |
| 56975 | | | 1677020 | Losartan 50mg / Hydrochlorothiazide 12.5mg tablets (A A H Pharmaceuticals Ltd) | Losartan potassium/Hydrochlorothiazide | | | 0 | 1 |
| 62911 | | | 77305020 | Losartan 50mg / Hydrochlorothiazide 12.5mg tablets (Teva UK Ltd) | Losartan potassium/Hydrochlorothiazide | | | 0 | 1 |
| 10323 | | | 89779020 | Losartan 100mg / Hydrochlorothiazide 25mg tablets | Losartan potassium/Hydrochlorothiazide | | | 0 | 1 |
| 38367 | | | 94427020 | Hydrochlorothiazide with losartan 12.5mg with 100mg Tablet | Losartan Potassium/Hydrochlorothiazide | | | 0 | 1 |
| 55160 | | | 1673020 | Cozaar-Comp 50mg/12.5mg tablets (Sigma Pharmaceuticals Plc) | Losartan potassium/Hydrochlorothiazide | | | 0 | 1 |
| 70754 | | | 1683020 | Losartan 50mg / Hydrochlorothiazide 12.5mg tablets (Ranbaxy (UK) Ltd) | Losartan potassium/Hydrochlorothiazide | | | 0 | 1 |
| 66598 | | | 31319021 | Losartan 50mg / Hydrochlorothiazide 12.5mg tablets (Lupin (Europe) Ltd) | Losartan potassium/Hydrochlorothiazide | | | 0 | 1 |
| 56204 | | | 77510020 | Losartan 50mg / Hydrochlorothiazide 12.5mg tablets (Actavis UK Ltd) | Losartan potassium/Hydrochlorothiazide | | | 0 | 1 |
| 4540 | | | 82246020 | Cozaar-Comp 50mg/12.5mg tablets (Merck Sharp & Dohme Ltd) | Losartan potassium/Hydrochlorothiazide | | | 0 | 1 |
| 14738 | | | 79035020 | Hydrochlorothiazide with losartan 12.5mg with 50mg Tablet | Losartan Potassium/Hydrochlorothiazide | | | 0 | 1 |
| 57796 | | | 1684020 | Cozaar-Comp 50mg/12.5mg tablets (DE Pharmaceuticals) | Losartan potassium/Hydrochlorothiazide | | | 0 | 1 |
| 6285 | | | 78212020 | Olmesartan medoxomil 20mg tablets | Olmesartan medoxomil | | | 1 |  |
| 6217 | | | 78179020 | Olmesartan medoxomil 10mg tablets | Olmesartan medoxomil | | | 1 |  |
| 6351 | | | 78176020 | Olmesartan medoxomil 40mg tablets | Olmesartan medoxomil | | | 1 |  |
| 14983 | | | 78194020 | Olmetec 10mg tablets (Daiichi Sankyo UK Ltd) | Olmesartan medoxomil | | | 1 |  |
| 20117 | | | 57407020 | Olmetec 40mg tablets (Daiichi Sankyo UK Ltd) | Olmesartan medoxomil | | | 1 |  |
| 39786 | | | 96351020 | Olmesartan medoxomil 10mg/5ml oral suspension | Olmesartan medoxomil | | | 1 |  |
| 18910 | | | 78191020 | Olmetec 20mg tablets (Daiichi Sankyo UK Ltd) | Olmesartan medoxomil | | | 1 |  |
| 40668 | | | 96927020 | Olmesartan medoxomil 40mg / Amlodipine 10mg tablets | Olmesartan medoxomil/Amlodipine besilate | | | 0 | 1 |
| 40639 | | | 96925020 | Olmesartan medoxomil 40mg / Amlodipine 5mg tablets | Olmesartan medoxomil/Amlodipine besilate | | | 0 | 1 |
| 40316 | | | 96923020 | Olmesartan medoxomil 20mg / Amlodipine 5mg tablets | Olmesartan medoxomil/Amlodipine besilate | | | 0 | 1 |
| 39984 | | | 96929020 | Sevikar 20mg/5mg tablets (Daiichi Sankyo UK Ltd) | Olmesartan medoxomil/Amlodipine besilate | | | 0 | 1 |
| 41203 | | | 96933020 | Sevikar 40mg/10mg tablets (Daiichi Sankyo UK Ltd) | Olmesartan medoxomil/Amlodipine besilate | | | 0 | 1 |
| 41205 | | | 96931020 | Sevikar 40mg/5mg tablets (Daiichi Sankyo UK Ltd) | Olmesartan medoxomil/Amlodipine besilate | | | 0 | 1 |
| 29634 | | | 90635020 | Olmetec Plus 20mg/12.5mg tablets (Daiichi Sankyo UK Ltd) | Olmesartan medoxomil/Hydrochlorothiazide | | | 0 | 1 |
| 18200 | | | 90629020 | Olmesartan medoxomil 20mg / Hydrochlorothiazide 12.5mg tablets | Olmesartan medoxomil/Hydrochlorothiazide | | | 0 | 1 |
| 66702 | | | 61233021 | Sacubitril 24mg / Valsartan 26mg tablets | Sacubitril/Valsartan | | | 0 | 1 |
| 66931 | | | 61237021 | Sacubitril 97mg / Valsartan 103mg tablets | Sacubitril/Valsartan | | | 0 | 1 |
| 66829 | | | 61238021 | Entresto 97mg/103mg tablets (Novartis Pharmaceuticals UK Ltd) | Sacubitril/Valsartan | | | 0 | 1 |
| 66261 | | | 61234021 | Entresto 24mg/26mg tablets (Novartis Pharmaceuticals UK Ltd) | Sacubitril/Valsartan | | | 0 | 1 |
| 17686 | | | 81476020 | Micardis 20mg tablets (Boehringer Ingelheim Ltd) | Telmisartan | | | 1 |  |
| 5988 | | | 74672020 | Telmisartan 40mg tablets | Telmisartan | | | 1 |  |
| 12874 | | | 74673020 | Telmisartan 80mg tablets | Telmisartan | | | 1 |  |
| 65274 | | | 21336021 | Telmisartan 40mg tablets (Actavis UK Ltd) | Telmisartan | | | 1 |  |
| 17545 | | | 81475020 | Micardis 80mg tablets (Boehringer Ingelheim Ltd) | Telmisartan | | | 1 |  |
| 6243 | | | 74674020 | Telmisartan 20mg tablets | Telmisartan | | | 1 |  |
| 13821 | | | 81474020 | Micardis 40mg tablets (Boehringer Ingelheim Ltd) | Telmisartan | | | 1 |  |
| 70251 | | | 21294021 | Telmisartan 20mg tablets (Teva UK Ltd) | Telmisartan | | | 1 |  |
| 61177 | | | 33020 | Telmisartan 20mg tablets (Sigma Pharmaceuticals Plc) | Telmisartan | | | 1 |  |
| 66997 | | | 52342021 | MicardisPlus 40mg/12.5mg tablets (Waymade Healthcare Plc) | Telmisartan/Hydrochlorothiazide | | | 0 | 1 |
| 16161 | | | 58831020 | Telmisartan 80mg / Hydrochlorothiazide 12.5mg tablets | Telmisartan/Hydrochlorothiazide | | | 0 | 1 |
| 62376 | | | 23794021 | Actelsar HCT 80mg/12.5mg tablets (Actavis UK Ltd) | Telmisartan/Hydrochlorothiazide | | | 0 | 1 |
| 14870 | | | 81237020 | Telmisartan 40mg / Hydrochlorothiazide 12.5mg tablets | Telmisartan/Hydrochlorothiazide | | | 0 | 1 |
| 17689 | | | 49280020 | MicardisPlus 80mg/12.5mg tablets (Boehringer Ingelheim Ltd) | Telmisartan/Hydrochlorothiazide | | | 0 | 1 |
| 18202 | | | 81233020 | MicardisPlus 40mg/12.5mg tablets (Boehringer Ingelheim Ltd) | Telmisartan/Hydrochlorothiazide | | | 0 | 1 |
| 63890 | | | 10554020 | MicardisPlus 80mg/12.5mg tablets (Waymade Healthcare Plc) | Telmisartan/Hydrochlorothiazide | | | 0 | 1 |
| 14943 | | | 88542020 | Valsartan 40mg tablets | Valsartan | | | 1 |  |
| 67663 | | | 1716020 | Valsartan 160mg capsules (Dexcel-Pharma Ltd) | Valsartan | | | 1 |  |
| 68948 | | | 1717020 | Valsartan 160mg capsules (Actavis UK Ltd) | Valsartan | | | 1 |  |
| 6518 | | | 83507020 | Diovan 160mg capsules (Novartis Pharmaceuticals UK Ltd) | Valsartan | | | 1 |  |
| 3222 | | | 83502020 | Valsartan 80mg capsules | Valsartan | | | 1 |  |
| 11251 | | | 83505020 | Diovan 40mg capsules (Novartis Pharmaceuticals UK Ltd) | Valsartan | | | 1 |  |
| 38395 | | | 91976020 | Valsartan 80mg tablets | Valsartan | | | 1 |  |
| 70628 | | | 99477020 | Diovan 3mg/1ml oral solution (Novartis Pharmaceuticals UK Ltd) | Valsartan | | | 1 |  |
| 39199 | | | 94445020 | Diovan 320mg tablets (Novartis Pharmaceuticals UK Ltd) | Valsartan | | | 1 |  |
| 71028 | | | 39797020 | Valsartan 80mg capsules (Arrow Generics Ltd) | Valsartan | | | 1 |  |
| 58669 | | | 1689020 | Valsartan 40mg capsules (Teva UK Ltd) | Valsartan | | | 1 |  |
| 24359 | | | 88544020 | Diovan 40mg tablets (Novartis Pharmaceuticals UK Ltd) | Valsartan | | | 1 |  |
| 58910 | | | 1699020 | Valsartan 80mg capsules (Sigma Pharmaceuticals Plc) | Valsartan | | | 1 |  |
| 54726 | | | 78678020 | Valsartan 40mg capsules (Teva UK Ltd) | Valsartan | | | 1 |  |
| 44778 | | | 91979020 | Valsartan 160mg tablets | Valsartan | | | 1 |  |
| 59448 | | | 38828020 | Valsartan 80mg capsules (A A H Pharmaceuticals Ltd) | Valsartan | | | 1 |  |
| 575 | | | 83501020 | Valsartan 40mg capsules | Valsartan | | | 1 |  |
| 53833 | | | 38960020 | Valsartan 160mg capsules (Mylan) | Valsartan | | | 1 |  |
| 37573 | | | 94443020 | Valsartan 320mg tablets | Valsartan | | | 1 |  |
| 59029 | | | 99475020 | Valsartan 3mg/1ml oral solution | Valsartan | | | 1 |  |
| 55187 | | | 39798020 | Valsartan 160mg capsules (Arrow Generics Ltd) | Valsartan | | | 1 |  |
| 61442 | | | 1715020 | Valsartan 160mg capsules (Teva UK Ltd) | Valsartan | | | 1 |  |
| 60076 | | | 47156020 | Valsartan 160mg capsules (Waymade Healthcare Plc) | Valsartan | | | 1 |  |
| 11252 | | | 83506020 | Diovan 80mg capsules (Novartis Pharmaceuticals UK Ltd) | Valsartan | | | 1 |  |
| 55821 | | | 78684020 | Valsartan 160mg capsules (Teva UK Ltd) | Valsartan | | | 1 |  |
| 45600 | | | 98993020 | Diovan 160mg Tablet (Novartis Pharmaceuticals UK Ltd) | Valsartan | | | 1 |  |
| 4645 | | | 83503020 | Valsartan 160mg capsules | Valsartan | | | 1 |  |
| 35343 | | | 92765020 | Amlodipine 5mg / Valsartan 160mg tablets | Valsartan/Amlodipine besilate | | | 0 | 1 |
| 35697 | | | 92771020 | Exforge 5mg/160mg tablets (Novartis Pharmaceuticals UK Ltd) | Valsartan/Amlodipine besilate | | | 0 | 1 |
| 67664 | | | 10168020 | Valsartan 160mg / Hydrochlorothiazide 12.5mg tablets (Teva UK Ltd) | Valsartan/Hydrochlorothiazide | | | 0 | 1 |
| 11864 | | | 87994020 | Valsartan 160mg / Hydrochlorothiazide 12.5mg tablets | Valsartan/Hydrochlorothiazide | | | 0 | 1 |
| 25382 | | | 88008020 | Co-Diovan 160mg/25mg tablets (Novartis Pharmaceuticals UK Ltd) | Valsartan/Hydrochlorothiazide | | | 0 | 1 |
| 24268 | | | 88422020 | Hydrochlorothiazide with valsartan 12.5mg with 80mg Tablet | Valsartan/Hydrochlorothiazide | | | 0 | 1 |
| 23456 | | | 88006020 | Hydrochlorothiazide with valsartan 25mg with 160mg Tablet | Valsartan/Hydrochlorothiazide | | | 0 | 1 |
| 24484 | | | 87996020 | Hydrochlorothiazide with valsartan 12.5mg with 160mg Tablet | Valsartan/Hydrochlorothiazide | | | 0 | 1 |
| 14283 | | | 88002020 | Valsartan 160mg / Hydrochlorothiazide 25mg tablets | Valsartan/Hydrochlorothiazide | | | 0 | 1 |
| 6877 | | | 87998020 | Co-Diovan 160mg/12.5mg tablets (Novartis Pharmaceuticals UK Ltd) | Valsartan/Hydrochlorothiazide | | | 0 | 1 |
| 66197 | | | 61235021 | Sacubitril 49mg / Valsartan 51mg tablets | Valsartan/Sacubitril | | | 0 | 1 |
| 66205 | 61236021 | Entresto 49mg/51mg tablets (Novartis Pharmaceuticals UK Ltd) | Valsartan/Sacubitril | | | 0 | 1 | | |

ARB, angiotensin II receptor blockers

**Supplementary Table 9. Code list for diuretics**

| **Diuretics** |  |  |  |  |  |
| --- | --- | --- | --- | --- | --- |
| **Prod**  **code** | **Gemscript**  **code** | **Product**  **name** | **Drugsubstance**  **name** | **To include** | **combination therapy to exclude** |
| 14126 | 59935020 | Acebutolol 200mg / Hydrochlorothiazide 12.5mg tablets | Acebutolol hydrochloride/Hydrochlorothiazide | 0 | 1 |
| 8189 | 51499020 | Secadrex 200mg/12.5mg tablets (Sanofi) | Acebutolol hydrochloride/Hydrochlorothiazide | 0 | 1 |
| 26217 | 68827020 | Berkamil 5mg Tablet (Berk Pharmaceuticals Ltd) | Amiloride hydrochloride | 1 |  |
| 43523 | 60182020 | Amiloride 5mg tablets (Mylan) | Amiloride hydrochloride | 1 |  |
| 33837 | 48461020 | Amiloride 5mg tablets (A A H Pharmaceuticals Ltd) | Amiloride hydrochloride | 1 |  |
| 13352 | 50402020 | Midamor 5mg Tablet (MSD Thomas Morson Pharmaceuticals) | Amiloride hydrochloride | 1 |  |
| 34750 | 56393020 | Amiloride 5mg tablets (Actavis UK Ltd) | Amiloride hydrochloride | 1 |  |
| 31375 | 82357020 | Amilamont 5mg/5ml oral solution sugar free (Rosemont Pharmaceuticals Ltd) | Amiloride hydrochloride | 1 |  |
| 41630 | 48466020 | Amiloride 5mg Tablet (IVAX Pharmaceuticals UK Ltd) | Amiloride hydrochloride | 1 |  |
| 24893 | 72514020 | Amilospare Tablet (Ashbourne Pharmaceuticals Ltd) | Amiloride hydrochloride | 1 |  |
| 60149 | 18921020 | Amiloride 5mg/5ml oral suspension | Amiloride hydrochloride | 1 |  |
| 46930 | 48455020 | Amiloride 5mg tablets (Wockhardt UK Ltd) | Amiloride hydrochloride | 1 |  |
| 9935 | 58944020 | Amiloride 5mg/5ml oral solution sugar free | Amiloride hydrochloride | 1 |  |
| 44254 | 58945020 | Amiloride 5.67mg tablets | Amiloride Hydrochloride | 1 |  |
| 34324 | 48451020 | Amiloride 5mg tablets (Teva UK Ltd) | Amiloride hydrochloride | 1 |  |
| 1060 | 58943020 | Amiloride 5mg tablets | Amiloride hydrochloride | 1 |  |
| 2255 | 73233020 | Navispare 2.5mg/250microgram tablets (AMCo) | Amiloride hydrochloride/Cyclopenthiazide | 0 | 1 |
| 5727 | 73241020 | Amiloride 2.5mg / Cyclopenthiazide 250microgram tablets | Amiloride hydrochloride/Cyclopenthiazide | 0 | 1 |
| 25965 | 55921020 | Co-amilofruse 2.5mg/20mg tablets (Wockhardt UK Ltd) | Amiloride hydrochloride/Furosemide | 0 | 1 |
| 43508 | 56458020 | Co-amilofruse 5mg/40mg tablets (Sandoz Ltd) | Amiloride hydrochloride/Furosemide | 0 | 1 |
| 31773 | 55922020 | Co-amilofruse 5mg/40mg tablets (Wockhardt UK Ltd) | Amiloride hydrochloride/Furosemide | 0 | 1 |
| 57908 | 62312020 | Co-amilofruse 5mg/40mg tablets (Kent Pharmaceuticals Ltd) | Amiloride hydrochloride/Furosemide | 0 | 1 |
| 60258 | 797020 | Co-amilofruse 2.5mg/20mg tablets (Aurobindo Pharma Ltd) | Amiloride hydrochloride/Furosemide | 0 | 1 |
| 9431 | 83268020 | Frusemek 40mg+5mg Tablet (Approved Prescription Services Ltd) | Amiloride hydrochloride/Furosemide | 0 | 1 |
| 1301 | 53108020 | Frumil ls 20mg+2.5mg Tablet (Helios Healthcare Ltd) | Amiloride hydrochloride/Furosemide | 0 | 1 |
| 39807 | 96714020 | Frumil 40mg/5mg tablets (Sanofi) | Amiloride hydrochloride/Furosemide | 0 | 1 |
| 34622 | 55923020 | Co-amilofruse 10mg/80mg tablets (Wockhardt UK Ltd) | Amiloride hydrochloride/Furosemide | 0 | 1 |
| 33527 | 60338020 | Co-amilofruse 5mg/40mg tablets (Mylan) | Amiloride hydrochloride/Furosemide | 0 | 1 |
| 3793 | 73911020 | Co-amilofruse 10mg/80mg tablets | Amiloride hydrochloride/Furosemide | 0 | 1 |
| 18332 | 75373020 | Aridil 20mg+2.5mg Tablet (C P Pharmaceuticals Ltd) | Amiloride hydrochloride/Furosemide | 0 | 1 |
| 56 | 73909020 | Co-amilofruse 5mg/40mg tablets | Amiloride hydrochloride/Furosemide | 0 | 1 |
| 21938 | 86513020 | Froop Co 5mg/40mg tablets (Ashbourne Pharmaceuticals Ltd) | Amiloride hydrochloride/Furosemide | 0 | 1 |
| 13435 | 53109020 | Frumil Forte 10mg/80mg tablets (Sanofi) | Amiloride hydrochloride/Furosemide | 0 | 1 |
| 193 | 73910020 | Co-amilofruse 2.5mg/20mg tablets | Amiloride hydrochloride/Furosemide | 0 | 1 |
| 211 | 53107020 | Frumil 40mg+5mg Tablet (Helios Healthcare Ltd) | Amiloride hydrochloride/Furosemide | 0 | 1 |
| 41719 | 56479020 | Co-amilofruse 5mg/40mg tablets (Actavis UK Ltd) | Amiloride hydrochloride/Furosemide | 0 | 1 |
| 4873 | 57115020 | Fru-Co 5mg/40mg tablets (Teva UK Ltd) | Amiloride hydrochloride/Furosemide | 0 | 1 |
| 38901 | 95531020 | Frumil LS 20mg/2.5mg tablets (Sanofi) | Amiloride hydrochloride/Furosemide | 0 | 1 |
| 2772 | 67963020 | Lasoride 5mg/40mg tablets (Sanofi) | Amiloride hydrochloride/Furosemide | 0 | 1 |
| 28129 | 62291020 | Co-amilofruse 5mg/40mg tablets (Teva UK Ltd) | Amiloride hydrochloride/Furosemide | 0 | 1 |
| 30773 | 54000020 | Co-amilofruse 5mg+40mg Tablet (Berk Pharmaceuticals Ltd) | Amiloride hydrochloride/Furosemide | 0 | 1 |
| 34280 | 56459020 | Co-amilofruse 2.5mg/20mg tablets (Sandoz Ltd) | Amiloride hydrochloride/Furosemide | 0 | 1 |
| 59412 | 47086020 | Co-amilofruse 5mg/40mg tablets (Waymade Healthcare Plc) | Amiloride hydrochloride/Furosemide | 0 | 1 |
| 33658 | 60274020 | Co-amilofruse 5mg/40mg tablets (A A H Pharmaceuticals Ltd) | Amiloride hydrochloride/Furosemide | 0 | 1 |
| 41533 | 63792020 | Co-amilofruse 2.5mg/20mg tablets (Teva UK Ltd) | Amiloride hydrochloride/Furosemide | 0 | 1 |
| 3293 | 50482020 | Moduretic Oral solution (Bristol-Myers Squibb Pharmaceuticals Ltd) | Amiloride Hydrochloride/Hydrochlorothiazide | 0 | 1 |
| 22923 | 63254020 | Hydrochlorothiazide with amiloride 50mg with 5mg Tablet | Amiloride Hydrochloride/Hydrochlorothiazide | 0 | 1 |
| 62249 | 10099020 | Co-amilozide 5mg/50mg tablets (Alliance Healthcare (Distribution) Ltd) | Amiloride hydrochloride/Hydrochlorothiazide | 0 | 1 |
| 26219 | 79843020 | Zida-co 5mg+50mg Tablet (Opus Pharmaceuticals Ltd) | Amiloride hydrochloride/Hydrochlorothiazide | 0 | 1 |
| 41556 | 53503020 | Co-amilozide 5mg/50mg tablets (Teva UK Ltd) | Amiloride hydrochloride/Hydrochlorothiazide | 0 | 1 |
| 31150 | 49311020 | Co-amilozide 5mg/50mg tablets (IVAX Pharmaceuticals UK Ltd) | Amiloride hydrochloride/Hydrochlorothiazide | 0 | 1 |
| 923 | 70711020 | Co-amilozide 5mg/50mg tablets | Amiloride hydrochloride/Hydrochlorothiazide | 0 | 1 |
| 46916 | 49316020 | Co-amilozide 5mg/50mg tablets (A A H Pharmaceuticals Ltd) | Amiloride hydrochloride/Hydrochlorothiazide | 0 | 1 |
| 348 | 50481020 | Moduretic Tablet (Bristol-Myers Squibb Pharmaceuticals Ltd) | Amiloride hydrochloride/Hydrochlorothiazide | 0 | 1 |
| 26220 | 68015020 | Delvas Tablet (Berk Pharmaceuticals Ltd) | Amiloride hydrochloride/Hydrochlorothiazide | 0 | 1 |
| 19890 | 63252020 | Hydrochlorothiazide with amiloride 25mgwith2.5mg Tablet | Amiloride Hydrochloride/Hydrochlorothiazide | 0 | 1 |
| 8058 | 63267020 | Normetic Tablet (Abbott Laboratories Ltd) | Amiloride hydrochloride/Hydrochlorothiazide | 0 | 1 |
| 4034 | 69293020 | Amiloride 5mg / hydrochlorothiazide 50mg/5ml solution | Amiloride Hydrochloride/Hydrochlorothiazide | 0 | 1 |
| 2002 | 69291020 | Amiloride 5mg / hydrochlorothiazide 50mg tablets | Amiloride Hydrochloride/Hydrochlorothiazide | 0 | 1 |
| 25500 | 68145020 | Hypertane 50 Tablet (Schwarz Pharma Ltd) | Amiloride hydrochloride/Hydrochlorothiazide | 0 | 1 |
| 42142 | 97965020 | Moduretic 5mg/50mg tablets (Merck Sharp & Dohme Ltd) | Amiloride hydrochloride/Hydrochlorothiazide | 0 | 1 |
| 3701 | 69292020 | Amiloride 2.5mg / hydrochlorothiazide 25mg tablets | Amiloride Hydrochloride/Hydrochlorothiazide | 0 | 1 |
| 62700 | 10107020 | Co-amilozide 5mg/50mg tablets (Phoenix Healthcare Distribution Ltd) | Amiloride hydrochloride/Hydrochlorothiazide | 0 | 1 |
| 18361 | 72435020 | Amilmaxco 5mg/50mg tablets (Ashbourne Pharmaceuticals Ltd) | Amiloride hydrochloride/Hydrochlorothiazide | 0 | 1 |
| 18733 | 70712020 | Co-amilozide 5mg with 50mg/ml oral solution | Amiloride Hydrochloride/Hydrochlorothiazide | 0 | 1 |
| 24008 | 72539020 | Vasetic Tablet (Shire Pharmaceuticals Ltd) | Amiloride hydrochloride/Hydrochlorothiazide | 0 | 1 |
| 20066 | 63264020 | Amil-Co 5mg/50mg tablets (IVAX Pharmaceuticals UK Ltd) | Amiloride hydrochloride/Hydrochlorothiazide | 0 | 1 |
| 47616 | 99785020 | Sevikar HCT 40mg/10mg/12.5mg tablets (Daiichi Sankyo UK Ltd) | Amlodipine besilate/Hydrochlorothiazide/Olmesartan medoxomil | 0 | 1 |
| 47573 | 99783020 | Sevikar HCT 40mg/5mg/12.5mg tablets (Daiichi Sankyo UK Ltd) | Amlodipine besilate/Hydrochlorothiazide/Olmesartan medoxomil | 0 | 1 |
| 60007 | 38012020 | Generic Sevikar HCT 40mg/10mg/12.5mg tablets | Amlodipine besilate/Hydrochlorothiazide/Olmesartan medoxomil | 0 | 1 |
| 55358 | 99777020 | Olmesartan medoxomil with amlodipine and hydrochlorothiazide 40mg + 10mg + 25mg Tablet | Amlodipine/Hydrochlorothiazide/Olmesartan Medoxomil | 0 | 1 |
| 46687 | 99769020 | Olmesartan medoxomil with amlodipine and hydrochlorothiazide 20mg + 5mg + 12.5mg Tablet | Amlodipine/Hydrochlorothiazide/Olmesartan Medoxomil | 0 | 1 |
| 46792 | 99771020 | Olmesartan medoxomil with amlodipine and hydrochlorothiazide 40mg + 5mg + 12.5mg Tablet | Amlodipine/Hydrochlorothiazide/Olmesartan Medoxomil | 0 | 1 |
| 46715 | 99773020 | Olmesartan medoxomil with amlodipine and hydrochlorothiazide 40mg + 10mg + 12.5mg Tablet | Amlodipine/Hydrochlorothiazide/Olmesartan Medoxomil | 0 | 1 |
| 47467 | 99775020 | Olmesartan medoxomil with amlodipine and hydrochlorothiazide 40mg + 5mg + 25mg Tablet | Amlodipine/Hydrochlorothiazide/Olmesartan Medoxomil | 0 | 1 |
| 7543 | 56162020 | Kalten capsules (M & A Pharmachem Ltd) | Atenolol/Amiloride hydrochloride/Hydrochlorothiazide | 0 | 1 |
| 4983 | 68844020 | Atenolol with amiloride and hydrochlorothiazide capsules | Atenolol/Amiloride Hydrochloride/Hydrochlorothiazide | 0 | 1 |
| 3526 | 69296020 | Amiloride with atenolol with hydrochlorothiazide capsules | Atenolol/Amiloride Hydrochloride/Hydrochlorothiazide | 0 | 1 |
| 28177 | 68841020 | Hydrochlorothiazide with atenolol and amiloride Capsule | Atenolol/Amiloride Hydrochloride/Hydrochlorothiazide | 0 | 1 |
| 9178 | 83986020 | Atenolol 25mg / Bendroflumethiazide 1.25mg capsules | Atenolol/Bendroflumethiazide | 0 | 1 |
| 18743 | 83989020 | Tenben 25mg/1.25mg capsules (Galen Ltd) | Atenolol/Bendroflumethiazide | 0 | 1 |
| 1288 | 51857020 | Tenoret 50mg/12.5mg tablets (AstraZeneca UK Ltd) | Atenolol/Chlortalidone | 0 | 1 |
| 5721 | 73883020 | Co-tenidone 100mg/25mg tablets | Atenolol/Chlortalidone | 0 | 1 |
| 34449 | 60352020 | Co-tenidone 50mg/12.5mg tablets (Mylan) | Atenolol/Chlortalidone | 0 | 1 |
| 46952 | 56911020 | Co-tenidone 100mg/25mg tablets (Actavis UK Ltd) | Atenolol/Chlortalidone | 0 | 1 |
| 34034 | 55247020 | Co-tenidone 50mg/12.5mg tablets (IVAX Pharmaceuticals UK Ltd) | Atenolol/Chlortalidone | 0 | 1 |
| 62537 | 21452021 | Co-tenidone 100mg/25mg tablets (DE Pharmaceuticals) | Atenolol/Chlortalidone | 0 | 1 |
| 24280 | 80150020 | Totaretic 100mg+25mg Tablet (C P Pharmaceuticals Ltd) | Atenolol/Chlortalidone | 0 | 1 |
| 31708 | 56910020 | Co-tenidone 50mg/12.5mg tablets (Actavis UK Ltd) | Atenolol/Chlortalidone | 0 | 1 |
| 31470 | 57164020 | Tenchlor 50mg/12.5mg tablets (Teva UK Ltd) | Atenolol/Chlortalidone | 0 | 1 |
| 32094 | 53521020 | Co-tenidone 50mg/12.5mg tablets (A A H Pharmaceuticals Ltd) | Atenolol/Chlortalidone | 0 | 1 |
| 26741 | 80149020 | Totaretic 50mg+12.5mg Tablet (C P Pharmaceuticals Ltd) | Atenolol/Chlortalidone | 0 | 1 |
| 1124 | 54815020 | Tenoretic 100mg/25mg tablets (AstraZeneca UK Ltd) | Atenolol/Chlortalidone | 0 | 1 |
| 26248 | 57165020 | Tenchlor 100mg/25mg tablets (Teva UK Ltd) | Atenolol/Chlortalidone | 0 | 1 |
| 34825 | 54653020 | Co-tenidone 50mg/12.5mg tablets (Teva UK Ltd) | Atenolol/Chlortalidone | 0 | 1 |
| 41572 | 54654020 | Co-tenidone 100mg/25mg tablets (Teva UK Ltd) | Atenolol/Chlortalidone | 0 | 1 |
| 34899 | 53522020 | Co-tenidone 100mg/25mg tablets (A A H Pharmaceuticals Ltd) | Atenolol/Chlortalidone | 0 | 1 |
| 13526 | 63763020 | Atenix Co 100 tablets (Ashbourne Pharmaceuticals Ltd) | Atenolol/Chlortalidone | 0 | 1 |
| 37725 | 60353020 | Co-tenidone 100mg/25mg tablets (Mylan) | Atenolol/Chlortalidone | 0 | 1 |
| 34012 | 55246020 | Co-tenidone 100mg/25mg tablets (IVAX Pharmaceuticals UK Ltd) | Atenolol/Chlortalidone | 0 | 1 |
| 9783 | 73882020 | Co-tenidone 50mg/12.5mg tablets | Atenolol/Chlortalidone | 0 | 1 |
| 21873 | 63762020 | Atenix Co 50 tablets (Ashbourne Pharmaceuticals Ltd) | Atenolol/Chlortalidone | 0 | 1 |
| 66517 | 19158020 | Bendroflumethiazide 1.25mg/5ml oral suspension | Bendroflumethiazide | 1 |  |
| 64907 | 70975020 | Bendroflumethiazide 5mg tablets (Almus Pharmaceuticals Ltd) | Bendroflumethiazide | 1 |  |
| 24190 | 75590020 | Neo-bendromax 5mg Tablet (Ashbourne Pharmaceuticals Ltd) | Bendroflumethiazide | 1 |  |
| 31820 | 48839020 | Bendroflumethiazide 5mg tablets (Wockhardt UK Ltd) | Bendroflumethiazide | 1 |  |
| 58 | 58977020 | Bendroflumethiazide 5mg tablets | Bendroflumethiazide | 1 |  |
| 67780 | 16655021 | Bendroflumethiazide 5mg tablets (DE Pharmaceuticals) | Bendroflumethiazide | 1 |  |
| 7351 | 90559020 | Bendroflumethiazide 2.5mg/5ml oral suspension | Bendroflumethiazide | 1 |  |
| 47844 | 66033020 | Bendroflumethiazide 2.5mg tablets (Kent Pharmaceuticals Ltd) | Bendroflumethiazide | 1 |  |
| 24189 | 75589020 | Neo-bendromax 2.5mg Tablet (Ashbourne Pharmaceuticals Ltd) | Bendroflumethiazide | 1 |  |
| 29991 | 52359020 | Centyl 5mg Tablet (Edwin Burgess Ltd) | Bendroflumethiazide | 1 |  |
| 27689 | 57793020 | Bendroflumethiazide 2.5mg tablets (IVAX Pharmaceuticals UK Ltd) | Bendroflumethiazide | 1 |  |
| 21867 | 48387020 | Berkozide 5mg Tablet (Berk Pharmaceuticals Ltd) | Bendroflumethiazide | 1 |  |
| 46302 | 99566020 | Neo-Naclex 2.5mg tablets (AMCo) | Bendroflumethiazide | 1 |  |
| 53812 | 92033020 | Bendroflumethiazide oral solution | Bendroflumethiazide | 1 |  |
| 40149 | 57796020 | Bendroflumethiazide 5mg tablets (IVAX Pharmaceuticals UK Ltd) | Bendroflumethiazide | 1 |  |
| 33415 | 63225020 | Bendroflumethiazide 2.5mg tablets (Mylan) | Bendroflumethiazide | 1 |  |
| 1209 | 50651020 | Neo-Naclex 5mg tablets (Mercury Pharma Group Ltd) | Bendroflumethiazide | 1 |  |
| 7698 | 48202020 | Aprinox 5mg tablets (Amdipharm Plc) | Bendroflumethiazide | 1 |  |
| 40886 | 68065020 | Bendroflumethiazide 2.5mg tablets (Almus Pharmaceuticals Ltd) | Bendroflumethiazide | 1 |  |
| 67737 | 71762020 | Bendroflumethiazide 2.5mg tablets (Dr Reddy's Laboratories (UK) Ltd) | Bendroflumethiazide | 1 |  |
| 67738 | 71766020 | Bendroflumethiazide 5mg tablets (Dr Reddy's Laboratories (UK) Ltd) | Bendroflumethiazide | 1 |  |
| 31670 | 54691020 | Bendroflumethiazide 2.5mg tablets (Teva UK Ltd) | Bendroflumethiazide | 1 |  |
| 34803 | 62827020 | Bendroflumethiazide 2.5mg Tablet (Regent Laboratories Ltd) | Bendroflumethiazide | 1 |  |
| 21803 | 48386020 | Berkozide 2.5mg Tablet (Berk Pharmaceuticals Ltd) | Bendroflumethiazide | 1 |  |
| 34059 | 55391020 | Bendroflumethiazide 2.5mg tablets (Actavis UK Ltd) | Bendroflumethiazide | 1 |  |
| 34124 | 55390020 | Bendroflumethiazide 5mg tablets (Actavis UK Ltd) | Bendroflumethiazide | 1 |  |
| 18973 | 52358020 | Centyl 2.5mg Tablet (Edwin Burgess Ltd) | Bendroflumethiazide | 1 |  |
| 41517 | 54690020 | Bendroflumethiazide 5mg tablets (Teva UK Ltd) | Bendroflumethiazide | 1 |  |
| 2 | 58976020 | Bendroflumethiazide 2.5mg tablets | Bendroflumethiazide | 1 |  |
| 27256 | 48838020 | Bendroflumethiazide 2.5mg tablets (Wockhardt UK Ltd) | Bendroflumethiazide | 1 |  |
| 33651 | 48846020 | Bendroflumethiazide 2.5mg tablets (A A H Pharmaceuticals Ltd) | Bendroflumethiazide | 1 |  |
| 70989 | 40498020 | Bendroflumethiazide 2.5mg tablets (Genesis Pharmaceuticals Ltd) | Bendroflumethiazide | 1 |  |
| 34602 | 66500020 | Bendroflumethiazide 2.5mg tablets (Sovereign Medical Ltd) | Bendroflumethiazide | 1 |  |
| 23427 | 48847020 | Bendroflumethiazide 5mg tablets (A A H Pharmaceuticals Ltd) | Bendroflumethiazide | 1 |  |
| 8526 | 48201020 | Aprinox 2.5mg tablets (AMCo) | Bendroflumethiazide | 1 |  |
| 1211 | 67326020 | Bendroflumethiazide 2.5mg / Potassium chloride 630mg (potassium 8.4mmol) modified-release tablets | Bendroflumethiazide/Potassium chloride | 1 |  |
| 2979 | 48615020 | Centyl k Tablet (Edwin Burgess Ltd) | Bendroflumethiazide/Potassium chloride | 1 |  |
| 20426 | 48616020 | Centyl k 2.5mg+7.7mmol Tablet (Edwin Burgess Ltd) | Bendroflumethiazide/Potassium chloride | 1 |  |
| 1213 | 50654020 | Neo-Naclex-K modified-release tablets (Mercury Pharma Group Ltd) | Bendroflumethiazide/Potassium chloride | 1 |  |
| 17561 | 67327020 | Bendroflumethiazide 2.5mg / Potassium chloride 573mg (potassium 7.7mmol) modified-release tablets | Bendroflumethiazide/Potassium chloride | 1 |  |
| 20431 | 78362020 | Centyl K modified-release tablets (LEO Pharma) | Bendroflumethiazide/Potassium chloride | 1 |  |
| 4406 | 54101020 | Decaserpyl plus Tablet (Roussel Laboratories Ltd) | Benzthiazide/methoserpidine | 0 | 1 |
| 29696 | 64499020 | Methoserpidine with benzthiazide Tablet | Benzthiazide/methoserpidine | 0 | 1 |
| 30272 | 64126020 | Benthiazide with Triamterene capsules | Benzthiazide/Triamterene | 0 | 1 |
| 17462 | 75023020 | Bisoprolol 10mg / Hydrochlorothiazide 6.25mg tablets | Bisoprolol fumarate/Hydrochlorothiazide | 0 | 1 |
| 17149 | 75020020 | Monozide 10 tablets (Wyeth Pharmaceuticals) | Bisoprolol fumarate/Hydrochlorothiazide | 0 | 1 |
| 7806 | 64265020 | Bumetanide 5mg tablets | Bumetanide | 1 |  |
| 2788 | 48499020 | Burinex 1mg tablets (LEO Pharma) | Bumetanide | 1 |  |
| 31932 | 56655020 | Bumetanide 1mg tablets (C P Pharmaceuticals Ltd) | Bumetanide | 1 |  |
| 12294 | 52763020 | Burinex 5mg tablets (LEO Pharma) | Bumetanide | 1 |  |
| 55548 | 727020 | Bumetanide 1mg tablets (Alliance Healthcare (Distribution) Ltd) | Bumetanide | 1 |  |
| 5218 | 64266020 | Bumetanide 1mg/5ml oral solution sugar free | Bumetanide | 1 |  |
| 12226 | 52766020 | Burinex 1mg/5ml Oral solution (LEO Pharma) | Bumetanide | 1 |  |
| 45305 | 59626020 | Bumetanide 1mg tablets (Teva UK Ltd) | Bumetanide | 1 |  |
| 62024 | 56649020 | Bumetanide 5mg tablets (A A H Pharmaceuticals Ltd) | Bumetanide | 1 |  |
| 34934 | 62808020 | Bumetanide 1mg tablets (Mylan) | Bumetanide | 1 |  |
| 36767 | 59167020 | Bumetanide 1mg tablets (IVAX Pharmaceuticals UK Ltd) | Bumetanide | 1 |  |
| 34613 | 59627020 | Bumetanide 5mg tablets (Teva UK Ltd) | Bumetanide | 1 |  |
| 32091 | 56648020 | Bumetanide 1mg tablets (A A H Pharmaceuticals Ltd) | Bumetanide | 1 |  |
| 39602 | 59590020 | Bumetanide 1mg tablets (Actavis UK Ltd) | Bumetanide | 1 |  |
| 814 | 64264020 | Bumetanide 1mg tablets | Bumetanide | 1 |  |
| 30913 | 85983020 | Betinex 1mg Tablet (Berk Pharmaceuticals Ltd) | Bumetanide | 1 |  |
| 66195 | 70978020 | Bumetanide 1mg tablets (Almus Pharmaceuticals Ltd) | Bumetanide | 1 |  |
| 63555 | 730020 | Bumetanide 1mg tablets (Phoenix Healthcare Distribution Ltd) | Bumetanide | 1 |  |
| 14587 | 73977020 | Amiloride 5mg / Bumetanide 1mg tablets | Bumetanide/Amiloride hydrochloride | 0 | 1 |
| 2495 | 73971020 | Bumetanide with Amiloride tablets | Bumetanide/Amiloride Hydrochloride | 0 | 1 |
| 2493 | 73968020 | Burinex A 5mg/1mg tablets (LEO Pharma) | Bumetanide/Amiloride hydrochloride | 0 | 1 |
| 1776 | 52368020 | Burinex K modified-release tablets (LEO Pharma) | Bumetanide/Potassium chloride | 1 |  |
| 6160 | 64272020 | Bumetanide 500microgram / Potassium chloride 573mg (potassium 7.7mmol) modified-release tablets | Bumetanide/Potassium chloride | 1 |  |
| 15135 | 69269020 | Hydrochlorothiazide with captopril 12.5mg with 25mg Tablet | Captopril/Hydrochlorothiazide | 0 | 1 |
| 11133 | 69268020 | Hydrochlorothiazide with captopril 25mg with 50mg Tablet | Captopril/Hydrochlorothiazide | 0 | 1 |
| 10902 | 68129020 | Captopril 50mg with Hydrochlorothiazide 25mg tablets | Captopril/Hydrochlorothiazide | 0 | 1 |
| 11641 | 68130020 | Captopril 25mg with Hydrochlorothiazide 12.5mg tablets | Captopril/Hydrochlorothiazide | 0 | 1 |
| 13246 | 90731020 | Chlorothiazide 150mg/5ml oral suspension | Chlorothiazide | 1 |  |
| 8836 | 61214020 | Chlorothiazide 500mg tablets | Chlorothiazide | 1 |  |
| 33724 | 86952020 | Diuril 250mg/5ml oral suspension (Imported (United States)) | Chlorothiazide | 1 |  |
| 64798 | 31984020 | Chlorothiazide 120mg/5ml oral solution | Chlorothiazide | 1 |  |
| 54679 | 98525020 | Chlorothiazide 250mg tablets | Chlorothiazide | 1 |  |
| 54341 | 30321020 | Chlorothiazide 5mg/5ml oral suspension | Chlorothiazide | 1 |  |
| 59834 | 32028020 | Chlorothiazide 250mg/5ml oral solution | Chlorothiazide | 1 |  |
| 17720 | 51466020 | Saluric 500mg Tablet (Merck Sharp & Dohme Ltd) | Chlorothiazide | 1 |  |
| 6816 | 86950020 | Chlorothiazide 250mg/5ml oral suspension | Chlorothiazide | 1 |  |
| 60603 | 19370020 | Chlorothiazide 50mg/5ml oral suspension | Chlorothiazide | 1 |  |
| 63227 | 30329020 | Chlorothiazide 70mg/5ml oral solution | Chlorothiazide | 1 |  |
| 56804 | 19362020 | Chlorothiazide 25mg/5ml oral suspension | Chlorothiazide | 1 |  |
| 55889 | 91770020 | Chlorothiazide oral solution | Chlorothiazide | 1 |  |
| 3997 | 49872020 | Hygroton 50mg tablets (Alliance Pharmaceuticals Ltd) | Chlortalidone | 1 |  |
| 605 | 61267020 | Chlortalidone 50mg tablets | Chlortalidone | 1 |  |
| 3054 | 49873020 | Hygroton 100mg Tablet (Alliance Pharmaceuticals Ltd) | Chlortalidone | 1 |  |
| 3548 | 61268020 | Chlortalidone 100mg tablets | Chlortalidone | 1 |  |
| 581 | 67597020 | Atenolol 50mg with Chlortalidone 12.5mg tablets | Chlortalidone/Atenolol | 0 | 1 |
| 16786 | 61276020 | Chlortalidone 25mg with Atenolol 100mg tablets | Chlortalidone/Atenolol | 0 | 1 |
| 19055 | 61275020 | Chlortalidone 12.5mg with Atenolol 50mg tablets | Chlortalidone/Atenolol | 0 | 1 |
| 1788 | 67598020 | Atenolol 100mg with Chlortalidone 25mg tablets | Chlortalidone/Atenolol | 0 | 1 |
| 12546 | 53154020 | Kalspare Tablet (Dominion Pharma) | Chlortalidone/Triamterene | 0 | 1 |
| 16498 | 69032020 | Kalspare tablets (DHP Healthcare Ltd) | Chlortalidone/Triamterene | 0 | 1 |
| 12547 | 67219020 | Triamterene 50mg / Chlortalidone 50mg tablets | Chlortalidone/Triamterene | 0 | 1 |
| 14057 | 65806020 | Pindolol 10mg / Clopamide 5mg tablets | Clopamide/Pindolol | 0 | 1 |
| 9143 | 52285020 | Viskaldix tablets (AMCo) | Clopamide/Pindolol | 0 | 1 |
| 2046 | 50633020 | Navidrex 500microgram tablets (AMCo) | Cyclopenthiazide | 1 |  |
| 1170 | 61684020 | Cyclopenthiazide 500microgram tablets | Cyclopenthiazide | 1 |  |
| 23505 | 81470020 | Adizem xl plus 150mg+12.5mg Modified-release capsule (Napp Pharmaceuticals Ltd) | Diltiazem Hydrochloride/Hydrochlorothiazide | 0 | 1 |
| 18606 | 81472020 | Diltiazem and hydrochlorothiazide 150mg+12.5mg modified-release capsules | Diltiazem Hydrochloride/Hydrochlorothiazide | 0 | 1 |
| 5189 | 51068020 | Enalapril 20mg / Hydrochlorothiazide 12.5mg tablets | Enalapril maleate/Hydrochlorothiazide | 0 | 1 |
| 1021 | 51047020 | Innozide 20mg/12.5mg tablets (Merck Sharp & Dohme Ltd) | Enalapril maleate/Hydrochlorothiazide | 0 | 1 |
| 14144 | 88414020 | Inspra 25mg tablets (Pfizer Ltd) | Eplerenone | 1 |  |
| 29694 | 88416020 | Inspra 50mg tablets (Pfizer Ltd) | Eplerenone | 1 |  |
| 16531 | 88412020 | Eplerenone 50mg tablets | Eplerenone | 1 |  |
| 70370 | 48886021 | Eplerenone 25mg tablets (Actavis UK Ltd) | Eplerenone | 1 |  |
| 70918 | 29757021 | Eplerenone 25mg tablets (Alliance Healthcare (Distribution) Ltd) | Eplerenone | 1 |  |
| 10251 | 88410020 | Eplerenone 25mg tablets | Eplerenone | 1 |  |
| 66011 | 31397021 | Eplerenone 50mg tablets (A A H Pharmaceuticals Ltd) | Eplerenone | 1 |  |
| 70543 | 48887021 | Eplerenone 50mg tablets (Actavis UK Ltd) | Eplerenone | 1 |  |
| 18650 | 49199020 | Edecrin 50mg Tablet (Merck Sharp & Dohme Ltd) | Etacrynic Acid | 1 |  |
| 12354 | 60884020 | Etacrynic 50mg tablets | Etacrynic Acid | 1 |  |
| 27447 | 49707020 | Furosemide 40mg tablets (Wockhardt UK Ltd) | Furosemide | 1 |  |
| 59030 | 30633020 | Furosemide 20mg/5ml oral solution | Furosemide | 1 |  |
| 21849 | 49105020 | Dryptal 40mg Tablet (Berk Pharmaceuticals Ltd) | Furosemide | 1 |  |
| 5728 | 75244020 | Furosemide 40mg/5ml oral solution sugar free | Furosemide | 1 |  |
| 7799 | 50115020 | Lasix 20mg tablets (Borg Medicare) | Furosemide | 1 |  |
| 6 | 59420020 | Furosemide 40mg tablets | Furosemide | 1 |  |
| 59884 | 698020 | Furosemide 20mg tablets (Phoenix Healthcare Distribution Ltd) | Furosemide | 1 |  |
| 55 | 59419020 | Furosemide 20mg tablets | Furosemide | 1 |  |
| 41828 | 53873020 | Furosemide 500mg tablets (Actavis UK Ltd) | Furosemide | 1 |  |
| 9680 | 83765020 | Frusol 40mg/5ml oral solution (Rosemont Pharmaceuticals Ltd) | Furosemide | 1 |  |
| 66149 | 30814020 | Furosemide 4.5mg/5ml oral solution | Furosemide | 1 |  |
| 34006 | 53872020 | Furosemide 40mg tablets (Actavis UK Ltd) | Furosemide | 1 |  |
| 61365 | 30820020 | Furosemide 40mg/5ml oral suspension | Furosemide | 1 |  |
| 42488 | 72847020 | Furosemide 40mg/5ml oral solution sugar free (A A H Pharmaceuticals Ltd) | Furosemide | 1 |  |
| 31548 | 53871020 | Furosemide 20mg tablets (Actavis UK Ltd) | Furosemide | 1 |  |
| 60465 | 35231020 | Furosemide 5mg/5ml oral solution | Furosemide | 1 |  |
| 66017 | 71522020 | Furosemide 20mg tablets (Almus Pharmaceuticals Ltd) | Furosemide | 1 |  |
| 56375 | 703020 | Furosemide 40mg tablets (Accord Healthcare Ltd) | Furosemide | 1 |  |
| 30625 | 49717020 | Furosemide 20mg tablets (A A H Pharmaceuticals Ltd) | Furosemide | 1 |  |
| 69445 | 714020 | Furosemide 40mg/5ml oral solution sugar free (Sigma Pharmaceuticals Plc) | Furosemide | 1 |  |
| 27926 | 59809020 | Furosemide 20mg tablets (Mylan) | Furosemide | 1 |  |
| 19192 | 63601020 | Furosemide 40mg Tablet (M & A Pharmachem Ltd) | Furosemide | 1 |  |
| 34374 | 53338020 | Furosemide 40mg tablets (Teva UK Ltd) | Furosemide | 1 |  |
| 58224 | 30609020 | Furosemide 10mg/5ml oral solution | Furosemide | 1 |  |
| 51983 | 35233020 | Furosemide 5mg/5ml oral suspension | Furosemide | 1 |  |
| 19056 | 55562020 | Furosemide 50mg/5ml sugar free Oral solution (Rosemont Pharmaceuticals Ltd) | Furosemide | 1 |  |
| 34557 | 53877020 | Furosemide 40mg tablets (IVAX Pharmaceuticals UK Ltd) | Furosemide | 1 |  |
| 32918 | 62708020 | Furosemide 20mg tablets (Sandoz Ltd) | Furosemide | 1 |  |
| 64677 | 21688021 | Furosemide 40mg tablets (DE Pharmaceuticals) | Furosemide | 1 |  |
| 14837 | 83766020 | Frusol 50mg/5ml oral solution (Rosemont Pharmaceuticals Ltd) | Furosemide | 1 |  |
| 4182 | 50110020 | Lasix 5mg/5ml oral solution (Borg Medicare) | Furosemide | 1 |  |
| 41405 | 53339020 | Furosemide 500mg tablets (Teva UK Ltd) | Furosemide | 1 |  |
| 67910 | 30818020 | Furosemide 40mg/5ml oral solution | Furosemide | 1 |  |
| 27690 | 49715020 | Furosemide 40mg tablets (A A H Pharmaceuticals Ltd) | Furosemide | 1 |  |
| 64255 | 30645020 | Furosemide 2mg/5ml oral solution | Furosemide | 1 |  |
| 42388 | 73018020 | Furosemide 40mg/5ml oral solution sugar free (Focus Pharmaceuticals Ltd) | Furosemide | 1 |  |
| 59290 | 692020 | Furosemide 20mg tablets (Alliance Healthcare (Distribution) Ltd) | Furosemide | 1 |  |
| 5868 | 83764020 | Frusol 20mg/5ml oral solution (Rosemont Pharmaceuticals Ltd) | Furosemide | 1 |  |
| 32896 | 60151020 | Furosemide 40mg tablets (Ranbaxy (UK) Ltd) | Furosemide | 1 |  |
| 63237 | 695020 | Furosemide 20mg tablets (Boston Healthcare Ltd) | Furosemide | 1 |  |
| 3287 | 76008020 | Furosemide 1mg/ml Oral solution | Furosemide | 1 |  |
| 36190 | 93839020 | Furosemide 5mg/5ml oral solution sugar free | Furosemide | 1 |  |
| 16206 | 74377020 | Froop 40mg tablets (Ashbourne Pharmaceuticals Ltd) | Furosemide | 1 |  |
| 49268 | 30828020 | Furosemide 50mg/5ml oral suspension | Furosemide | 1 |  |
| 56051 | 693020 | Furosemide 20mg tablets (Kent Pharmaceuticals Ltd) | Furosemide | 1 |  |
| 46948 | 67666020 | Furosemide 40mg tablets (Arrow Generics Ltd) | Furosemide | 1 |  |
| 68068 | 19963020 | Furosemide 4mg/5ml oral solution | Furosemide | 1 |  |
| 46699 | 71525020 | Furosemide 40mg tablets (Almus Pharmaceuticals Ltd) | Furosemide | 1 |  |
| 64745 | 30844020 | Furosemide 8mg/5ml oral suspension | Furosemide | 1 |  |
| 54825 | 696020 | Furosemide 20mg tablets (Sigma Pharmaceuticals Plc) | Furosemide | 1 |  |
| 7606 | 50116020 | Lasix 40mg tablets (Sanofi) | Furosemide | 1 |  |
| 69338 | 55561020 | Furosemide 40mg/5ml sugar free Oral solution (Rosemont Pharmaceuticals Ltd) | Furosemide | 1 |  |
| 5249 | 75245020 | Furosemide 50mg/5ml oral solution sugar free | Furosemide | 1 |  |
| 58078 | 30842020 | Furosemide 8mg/5ml oral solution | Furosemide | 1 |  |
| 59911 | 699020 | Furosemide 40mg tablets (Alliance Healthcare (Distribution) Ltd) | Furosemide | 1 |  |
| 6118 | 59421020 | Furosemide 20mg/5ml oral solution sugar free | Furosemide | 1 |  |
| 65583 | 30810020 | Furosemide 3mg/5ml oral solution | Furosemide | 1 |  |
| 29780 | 49706020 | Furosemide 20mg Tablet (C P Pharmaceuticals Ltd) | Furosemide | 1 |  |
| 59939 | 30635020 | Furosemide 20mg/5ml oral suspension | Furosemide | 1 |  |
| 25334 | 49716020 | Furosemide 500mg tablets (A A H Pharmaceuticals Ltd) | Furosemide | 1 |  |
| 47815 | 49711020 | Furosemide 20mg Tablet (Celltech Pharma Europe Ltd) | Furosemide | 1 |  |
| 26292 | 58432020 | Diuresal 40mg Tablet (Lagap) | Furosemide | 1 |  |
| 3248 | 59424020 | Furosemide 500mg tablets | Furosemide | 1 |  |
| 27696 | 65493020 | Furosemide 40mg tablets (Kent Pharmaceuticals Ltd) | Furosemide | 1 |  |
| 61475 | 21687021 | Furosemide 20mg tablets (DE Pharmaceuticals) | Furosemide | 1 |  |
| 14761 | 59523020 | Frusid 40mg tablets (Dr Reddy's Laboratories (UK) Ltd) | Furosemide | 1 |  |
| 57610 | 73015020 | Furosemide 20mg/5ml oral solution sugar free (Focus Pharmaceuticals Ltd) | Furosemide | 1 |  |
| 25717 | 59808020 | Furosemide 40mg tablets (Mylan) | Furosemide | 1 |  |
| 10392 | 62154020 | Lasix 500mg tablets (Sanofi) | Furosemide | 1 |  |
| 60291 | 704020 | Furosemide 40mg tablets (AMCo) | Furosemide | 1 |  |
| 53967 | 697020 | Furosemide 20mg tablets (Bristol Laboratories Ltd) | Furosemide | 1 |  |
| 19194 | 53340020 | Furosemide 20mg tablets (Teva UK Ltd) | Furosemide | 1 |  |
| 41292 | 76949020 | Furosemide 20mg tablets (Wockhardt UK Ltd) | Furosemide | 1 |  |
| 20538 | 48216020 | Frumax 40mg Tablet (Ashbourne Pharmaceuticals Ltd) | Furosemide | 1 |  |
| 47647 | 92509020 | Co-amilofruse oral liquid | Furosemide/Amiloride Hydrochloride | 0 | 1 |
| 9456 | 69287020 | Amiloride 5mg / furosemide 40mg tablets | Furosemide/Amiloride Hydrochloride | 0 | 1 |
| 1369 | 62142020 | Furosemide with amiloride 40mg+5mg Tablet | Furosemide/Amiloride Hydrochloride | 0 | 1 |
| 4211 | 62143020 | Furosemide with amiloride 20mg+2.5mg Tablet | Furosemide/Amiloride Hydrochloride | 0 | 1 |
| 18497 | 69288020 | Amiloride 10mg / furosemide 80mg tablets | Furosemide/Amiloride Hydrochloride | 0 | 1 |
| 5220 | 62144020 | Furosemide with amiloride 80mg+10mg Tablet | Furosemide/Amiloride Hydrochloride | 0 | 1 |
| 15874 | 69286020 | Amiloride 2.5mg / furosemide 20mg tablets | Furosemide/Amiloride Hydrochloride | 0 | 1 |
| 16632 | 63261020 | Hydrosaluric 50mg tablets (Merck Sharp & Dohme Ltd) | Hydrochlorothiazide | 1 |  |
| 542 | 63248020 | Hydrochlorothiazide 25mg tablets | Hydrochlorothiazide | 1 |  |
| 48132 | 94685020 | Hydrochlorothiazide Capsule | Hydrochlorothiazide | 1 |  |
| 3517 | 63249020 | Hydrochlorothiazide 50mg tablets | Hydrochlorothiazide | 1 |  |
| 17252 | 49321020 | Esidrex 25mg Tablet (Novartis Pharmaceuticals UK Ltd) | Hydrochlorothiazide | 1 |  |
| 13363 | 49322020 | Esidrex 50mg Tablet (Novartis Pharmaceuticals UK Ltd) | Hydrochlorothiazide | 1 |  |
| 57488 | 92809020 | Hydrochlorothiazide Oral solution | Hydrochlorothiazide | 1 |  |
| 12440 | 63260020 | Hydrosaluric 25mg tablets (Merck Sharp & Dohme Ltd) | Hydrochlorothiazide | 1 |  |
| 62516 | 26913021 | Hydrochlorothiazide 12.5mg tablets | Hydrochlorothiazide | 1 |  |
| 924 | 70710020 | Co-amilozide 2.5mg/25mg tablets | Hydrochlorothiazide/Amiloride hydrochloride | 0 | 1 |
| 60354 | 787020 | Co-amilozide 2.5mg/25mg tablets (Kent Pharmaceuticals Ltd) | Hydrochlorothiazide/Amiloride hydrochloride | 0 | 1 |
| 1251 | 58026020 | Moduret 25 tablets (Merck Sharp & Dohme Ltd) | Hydrochlorothiazide/Amiloride hydrochloride | 0 | 1 |
| 34367 | 55013020 | Co-amilozide 2.5mg/25mg tablets (Wockhardt UK Ltd) | Hydrochlorothiazide/Amiloride hydrochloride | 0 | 1 |
| 47727 | 99789020 | Sevikar HCT 40mg/5mg/25mg tablets (Daiichi Sankyo UK Ltd) | Hydrochlorothiazide/Amlodipine besilate/Olmesartan medoxomil | 0 | 1 |
| 53220 | 99791020 | Sevikar HCT 40mg/10mg/25mg tablets (Daiichi Sankyo UK Ltd) | Hydrochlorothiazide/Amlodipine besilate/Olmesartan medoxomil | 0 | 1 |
| 3203 | 68134020 | Capozide LS Tablet (E R Squibb and Sons Ltd) | Hydrochlorothiazide/Captopril | 0 | 1 |
| 39227 | 95527020 | Capozide LS 12.5mg/25mg tablets (Bristol-Myers Squibb Pharmaceuticals Ltd) | Hydrochlorothiazide/Captopril | 0 | 1 |
| 18263 | 59646020 | Acezide 25mg/50mg tablets (Bristol-Myers Squibb Pharmaceuticals Ltd) | Hydrochlorothiazide/Captopril | 0 | 1 |
| 11351 | 84482020 | Co-zidocapt 25mg/50mg tablets | Hydrochlorothiazide/Captopril | 0 | 1 |
| 1520 | 68133020 | Capozide 25mg/50mg tablets (Bristol-Myers Squibb Pharmaceuticals Ltd) | Hydrochlorothiazide/Captopril | 0 | 1 |
| 11561 | 84483020 | Co-zidocapt 12.5mg/25mg tablets | Hydrochlorothiazide/Captopril | 0 | 1 |
| 32166 | 80831020 | Capto-co 25mg+50mg Tablet (IVAX Pharmaceuticals UK Ltd) | Hydrochlorothiazide/Captopril | 0 | 1 |
| 35196 | 92367020 | CoAprovel 300mg/25mg tablets (Sanofi) | Hydrochlorothiazide/Irbesartan | 0 | 1 |
| 35481 | 92365020 | Irbesartan 300mg / Hydrochlorothiazide 25mg tablets | Hydrochlorothiazide/Irbesartan | 0 | 1 |
| 37650 | 94425020 | Losartan 100mg / Hydrochlorothiazide 12.5mg tablets | Hydrochlorothiazide/Losartan potassium | 0 | 1 |
| 48039 | 78259020 | Losartan 100mg / Hydrochlorothiazide 12.5mg tablets (Teva UK Ltd) | Hydrochlorothiazide/Losartan potassium | 0 | 1 |
| 37747 | 94429020 | Cozaar-Comp 100mg/12.5mg tablets (Merck Sharp & Dohme Ltd) | Hydrochlorothiazide/Losartan potassium | 0 | 1 |
| 70922 | 34060020 | Losartan 100mg / Hydrochlorothiazide 12.5mg tablets (Phoenix Healthcare Distribution Ltd) | Hydrochlorothiazide/Losartan potassium | 0 | 1 |
| 18903 | 90631020 | Olmesartan medoxomil 20mg / Hydrochlorothiazide 25mg tablets | Hydrochlorothiazide/Olmesartan medoxomil | 0 | 1 |
| 39021 | 90733020 | Hydrochlorothiazide with olmesartan medoxomil 25mg with 20mg tablet | Hydrochlorothiazide/Olmesartan Medoxomil | 0 | 1 |
| 35380 | 90735020 | Hydrochlorothiazide with olmesartan medoxomil 12.5mg with 20mg tablet | Hydrochlorothiazide/Olmesartan Medoxomil | 0 | 1 |
| 27520 | 90637020 | Olmetec Plus 20mg/25mg tablets (Daiichi Sankyo UK Ltd) | Hydrochlorothiazide/Olmesartan medoxomil | 0 | 1 |
| 43915 | 98339020 | Olmetec Plus 40mg/12.5mg tablets (Daiichi Sankyo UK Ltd) | Hydrochlorothiazide/Olmesartan medoxomil | 0 | 1 |
| 43322 | 98337020 | Olmesartan medoxomil 40mg / Hydrochlorothiazide 12.5mg tablets | Hydrochlorothiazide/Olmesartan medoxomil | 0 | 1 |
| 60780 | 38011020 | Generic Sevikar HCT 20mg/5mg/12.5mg tablets | Hydrochlorothiazide/Olmesartan medoxomil/Amlodipine besilate | 0 | 1 |
| 46355 | 99779020 | Sevikar HCT 20mg/5mg/12.5mg tablets (Daiichi Sankyo UK Ltd) | Hydrochlorothiazide/Olmesartan medoxomil/Amlodipine besilate | 0 | 1 |
| 15031 | 74293020 | Accuretic 12.5mg/10mg tablets (Pfizer Ltd) | Hydrochlorothiazide/Quinapril hydrochloride | 0 | 1 |
| 15108 | 74296020 | Quinapril 10mg / Hydrochlorothiazide 12.5mg tablets | Hydrochlorothiazide/Quinapril hydrochloride | 0 | 1 |
| 38889 | 95336020 | MicardisPlus 80mg/25mg tablets (Boehringer Ingelheim Ltd) | Hydrochlorothiazide/Telmisartan | 0 | 1 |
| 38459 | 95334020 | Telmisartan 80mg / Hydrochlorothiazide 25mg tablets | Hydrochlorothiazide/Telmisartan | 0 | 1 |
| 47804 | 74717020 | Co-triamterzide 50mg/25mg tablets (A A H Pharmaceuticals Ltd) | Hydrochlorothiazide/Triamterene | 0 | 1 |
| 18726 | 63766020 | Triamaxco 50mg/25mg tablets (Ashbourne Pharmaceuticals Ltd) | Hydrochlorothiazide/Triamterene | 0 | 1 |
| 1721 | 49166020 | Dyazide 50mg/25mg tablets (AMCo) | Hydrochlorothiazide/Triamterene | 0 | 1 |
| 67801 | 10850020 | Dyazide 50mg/25mg tablets (Lexon (UK) Ltd) | Hydrochlorothiazide/Triamterene | 0 | 1 |
| 8897 | 63273020 | Triam-Co 50mg/25mg tablets (IVAX Pharmaceuticals UK Ltd) | Hydrochlorothiazide/Triamterene | 0 | 1 |
| 5416 | 57075020 | Co-triamterzide 50mg/25mg tablets | Hydrochlorothiazide/Triamterene | 0 | 1 |
| 16060 | 88420020 | Valsartan 80mg / Hydrochlorothiazide 12.5mg tablets | Hydrochlorothiazide/Valsartan | 0 | 1 |
| 52858 | 10088020 | Co-Diovan 80mg/12.5mg tablets (Sigma Pharmaceuticals Plc) | Hydrochlorothiazide/Valsartan | 0 | 1 |
| 764 | 88424020 | Co-Diovan 80mg/12.5mg tablets (Novartis Pharmaceuticals UK Ltd) | Hydrochlorothiazide/Valsartan | 0 | 1 |
| 13525 | 58231020 | Hydrenox 50mg Tablet (Knoll Ltd) | Hydroflumethiazide | 1 |  |
| 12110 | 63279020 | Hydroflumethiazide 50mg Tablet | Hydroflumethiazide | 1 |  |
| 60020 | 14463021 | Indapamide 1.5mg modified-release tablets (Waymade Healthcare Plc) | Indapamide | 1 |  |
| 64066 | 46287020 | Indapamide 2.5mg/5ml oral suspension | Indapamide | 1 |  |
| 39447 | 95950020 | Varbim XL 1.5mg tablets (Teva UK Ltd) | Indapamide | 1 |  |
| 3056 | 84103020 | Natrilix SR 1.5mg tablets (Servier Laboratories Ltd) | Indapamide | 1 |  |
| 46675 | 75757020 | Indapamide 1.5mg modified-release tablets (A A H Pharmaceuticals Ltd) | Indapamide | 1 |  |
| 44168 | 97012020 | Indipam XL 1.5mg tablets (Actavis UK Ltd) | Indapamide | 1 |  |
| 62771 | 23030021 | Indapamide 1.5mg modified-release tablets (DE Pharmaceuticals) | Indapamide | 1 |  |
| 62066 | 23031021 | Cardide SR 1.5mg tablets (Teva UK Ltd) | Indapamide | 1 |  |
| 41885 | 95348020 | Ethibide XL 1.5mg tablets (Genus Pharmaceuticals Ltd) | Indapamide | 1 |  |
| 41861 | 95960020 | Tensaid XL 1.5mg tablets (Mylan) | Indapamide | 1 |  |
| 59616 | 678020 | Rawel XL 1.5mg tablets (Consilient Health Ltd) | Indapamide | 1 |  |
| 43184 | 97115020 | Mapemid XL 1.5mg tablets (Teva UK Ltd) | Indapamide | 1 |  |
| 5112 | 63541020 | Indapamide 1.5mg modified-release tablets | Indapamide | 1 |  |
| 43516 | 57007020 | Indapamide 2.5mg tablets (Actavis UK Ltd) | Indapamide hemihydrate | 1 |  |
| 2612 | 63540020 | Indapamide 2.5mg tablets | Indapamide hemihydrate | 1 |  |
| 55259 | 682020 | Indapamide 2.5mg tablets (Kent Pharmaceuticals Ltd) | Indapamide hemihydrate | 1 |  |
| 48099 | 60381020 | Indapamide 2.5mg tablets (A A H Pharmaceuticals Ltd) | Indapamide hemihydrate | 1 |  |
| 56296 | 685020 | Indapamide 2.5mg tablets (Boston Healthcare Ltd) | Indapamide hemihydrate | 1 |  |
| 34551 | 60492020 | Indapamide 2.5mg tablets (Mylan) | Indapamide hemihydrate | 1 |  |
| 26256 | 78942020 | Opumide 2.5mg Tablet (Opus Pharmaceuticals Ltd) | Indapamide hemihydrate | 1 |  |
| 42906 | 63983020 | Indapamide 2.5mg tablets (Niche Generics Ltd) | Indapamide hemihydrate | 1 |  |
| 56760 | 690020 | Indapamide 2.5mg tablets (Strides Shasun (UK) Ltd) | Indapamide hemihydrate | 1 |  |
| 26275 | 75814020 | Nindaxa 2.5 tablets (Ashbourne Pharmaceuticals Ltd) | Indapamide hemihydrate | 1 |  |
| 54316 | 683020 | Indapamide 2.5mg tablets (Alliance Healthcare (Distribution) Ltd) | Indapamide hemihydrate | 1 |  |
| 49529 | 689020 | Indapamide 2.5mg tablets (Phoenix Healthcare Distribution Ltd) | Indapamide hemihydrate | 1 |  |
| 70509 | 684020 | Indapamide 2.5mg tablets (DE Pharmaceuticals) | Indapamide hemihydrate | 1 |  |
| 40907 | 68581020 | Indapamide 2.5mg tablets (Genus Pharmaceuticals Ltd) | Indapamide hemihydrate | 1 |  |
| 7641 | 58678020 | Natrilix 2.5mg tablets (Servier Laboratories Ltd) | Indapamide hemihydrate | 1 |  |
| 48079 | 59993020 | Indapamide 2.5mg tablets (Zentiva) | Indapamide hemihydrate | 1 |  |
| 33083 | 56755020 | Indapamide 2.5mg tablets (Teva UK Ltd) | Indapamide hemihydrate | 1 |  |
| 27957 | 78343020 | Natramid 2.5mg Tablet (Trinity Pharmaceuticals Ltd) | Indapamide hemihydrate | 1 |  |
| 6794 | 83957020 | Perindopril erbumine 4mg / Indapamide 1.25mg tablets | Indapamide/Perindopril erbumine | 0 | 1 |
| 14228 | 84712020 | Coversyl Plus tablets (Servier Laboratories Ltd) | Indapamide/Perindopril erbumine | 0 | 1 |
| 56157 | 47939020 | Perindopril tosilate 5mg / Indapamide 1.25mg tablets | Indapamide/Perindopril tosilate | 0 | 1 |
| 11526 | 83982020 | CoAprovel 300mg/12.5mg tablets (Sanofi) | Irbesartan/Hydrochlorothiazide | 0 | 1 |
| 11469 | 80125020 | Irbesartan 300mg / Hydrochlorothiazide 12.5mg tablets | Irbesartan/Hydrochlorothiazide | 0 | 1 |
| 11448 | 80124020 | Irbesartan 150mg / Hydrochlorothiazide 12.5mg tablets | Irbesartan/Hydrochlorothiazide | 0 | 1 |
| 62337 | 20118021 | Irbesartan 300mg / Hydrochlorothiazide 12.5mg tablets (Actavis UK Ltd) | Irbesartan/Hydrochlorothiazide | 0 | 1 |
| 10316 | 83981020 | CoAprovel 150mg/12.5mg tablets (Sanofi) | Irbesartan/Hydrochlorothiazide | 0 | 1 |
| 38995 | 95877020 | Zestoretic 20 tablets (AstraZeneca UK Ltd) | Lisinopril/Hydrochlorothiazide | 0 | 1 |
| 21231 | 87820020 | Caralpha 20mg/12.5mg tablets (Actavis UK Ltd) | Lisinopril/Hydrochlorothiazide | 0 | 1 |
| 6786 | 50823020 | Lisinopril 10mg / Hydrochlorothiazide 12.5mg tablets | Lisinopril/Hydrochlorothiazide | 0 | 1 |
| 55399 | 67847020 | Lisinopril 20mg / Hydrochlorothiazide 12.5mg tablets (A A H Pharmaceuticals Ltd) | Lisinopril/Hydrochlorothiazide | 0 | 1 |
| 33353 | 67697020 | Lisinopril 20mg / Hydrochlorothiazide 12.5mg tablets (Teva UK Ltd) | Lisinopril/Hydrochlorothiazide | 0 | 1 |
| 56244 | 1491020 | Lisinopril 20mg / Hydrochlorothiazide 12.5mg tablets (Tillomed Laboratories Ltd) | Lisinopril/Hydrochlorothiazide | 0 | 1 |
| 37710 | 67694020 | Lisinopril 10mg / Hydrochlorothiazide 12.5mg tablets (Teva UK Ltd) | Lisinopril/Hydrochlorothiazide | 0 | 1 |
| 39137 | 95875020 | Zestoretic 10 tablets (AstraZeneca UK Ltd) | Lisinopril/Hydrochlorothiazide | 0 | 1 |
| 9764 | 72035020 | Carace 20 Tablet (Bristol-Myers Squibb Pharmaceuticals Ltd) | Lisinopril/Hydrochlorothiazide | 0 | 1 |
| 71115 | 1500020 | Zestoretic 10 tablets (Waymade Healthcare Plc) | Lisinopril/Hydrochlorothiazide | 0 | 1 |
| 17655 | 72036020 | Carace 10 Tablet (Bristol-Myers Squibb Pharmaceuticals Ltd) | Lisinopril/Hydrochlorothiazide | 0 | 1 |
| 39147 | 95901020 | Carace 20 Plus tablets (Merck Sharp & Dohme Ltd) | Lisinopril/Hydrochlorothiazide | 0 | 1 |
| 6359 | 51463020 | Zestoretic 10- 10mg+12.5mg Tablet (AstraZeneca UK Ltd) | Lisinopril/Hydrochlorothiazide | 0 | 1 |
| 39242 | 95903020 | Carace 10 Plus tablets (Merck Sharp & Dohme Ltd) | Lisinopril/Hydrochlorothiazide | 0 | 1 |
| 67767 | 75921020 | Lisinopril 10mg / Hydrochlorothiazide 12.5mg tablets (Almus Pharmaceuticals Ltd) | Lisinopril/Hydrochlorothiazide | 0 | 1 |
| 2982 | 51462020 | Zestoretic 20- 20mg+12.5mg Tablet (AstraZeneca UK Ltd) | Lisinopril/Hydrochlorothiazide | 0 | 1 |
| 57539 | 1503020 | Zestoretic 10 tablets (Sigma Pharmaceuticals Plc) | Lisinopril/Hydrochlorothiazide | 0 | 1 |
| 6468 | 50822020 | Lisinopril 20mg / Hydrochlorothiazide 12.5mg tablets | Lisinopril/Hydrochlorothiazide | 0 | 1 |
| 54201 | 75924020 | Lisinopril 20mg / Hydrochlorothiazide 12.5mg tablets (Almus Pharmaceuticals Ltd) | Lisinopril/Hydrochlorothiazide | 0 | 1 |
| 21423 | 89783020 | Cozaar-Comp 100mg/25mg tablets (Merck Sharp & Dohme Ltd) | Losartan potassium/Hydrochlorothiazide | 0 | 1 |
| 52189 | 10152020 | Losartan 100mg / Hydrochlorothiazide 25mg tablets (A A H Pharmaceuticals Ltd) | Losartan potassium/Hydrochlorothiazide | 0 | 1 |
| 24632 | 89781020 | Hydrochlorothiazide with losartan 25mg with 100mg Tablet | Losartan Potassium/Hydrochlorothiazide | 0 | 1 |
| 6437 | 79786020 | Losartan 50mg / Hydrochlorothiazide 12.5mg tablets | Losartan potassium/Hydrochlorothiazide | 0 | 1 |
| 56975 | 1677020 | Losartan 50mg / Hydrochlorothiazide 12.5mg tablets (A A H Pharmaceuticals Ltd) | Losartan potassium/Hydrochlorothiazide | 0 | 1 |
| 62911 | 77305020 | Losartan 50mg / Hydrochlorothiazide 12.5mg tablets (Teva UK Ltd) | Losartan potassium/Hydrochlorothiazide | 0 | 1 |
| 10323 | 89779020 | Losartan 100mg / Hydrochlorothiazide 25mg tablets | Losartan potassium/Hydrochlorothiazide | 0 | 1 |
| 38367 | 94427020 | Hydrochlorothiazide with losartan 12.5mg with 100mg Tablet | Losartan Potassium/Hydrochlorothiazide | 0 | 1 |
| 55160 | 1673020 | Cozaar-Comp 50mg/12.5mg tablets (Sigma Pharmaceuticals Plc) | Losartan potassium/Hydrochlorothiazide | 0 | 1 |
| 70754 | 1683020 | Losartan 50mg / Hydrochlorothiazide 12.5mg tablets (Ranbaxy (UK) Ltd) | Losartan potassium/Hydrochlorothiazide | 0 | 1 |
| 66598 | 31319021 | Losartan 50mg / Hydrochlorothiazide 12.5mg tablets (Lupin (Europe) Ltd) | Losartan potassium/Hydrochlorothiazide | 0 | 1 |
| 56204 | 77510020 | Losartan 50mg / Hydrochlorothiazide 12.5mg tablets (Actavis UK Ltd) | Losartan potassium/Hydrochlorothiazide | 0 | 1 |
| 4540 | 82246020 | Cozaar-Comp 50mg/12.5mg tablets (Merck Sharp & Dohme Ltd) | Losartan potassium/Hydrochlorothiazide | 0 | 1 |
| 14738 | 79035020 | Hydrochlorothiazide with losartan 12.5mg with 50mg Tablet | Losartan Potassium/Hydrochlorothiazide | 0 | 1 |
| 57796 | 1684020 | Cozaar-Comp 50mg/12.5mg tablets (DE Pharmaceuticals) | Losartan potassium/Hydrochlorothiazide | 0 | 1 |
| 15457 | 48303020 | Baycaron 25mg Tablet (Bayer Plc) | Mefruside | 1 |  |
| 17143 | 64368020 | Mefruside 25mg Tablet | Mefruside | 1 |  |
| 8303 | 51854020 | Tenavoid Tablet (Edwin Burgess Ltd) | Meprobamate/Bendroflumethiazide | 1 |  |
| 8464 | 64409020 | Meprobamate with bendroflumethiazide Tablet | Meprobamate/Bendroflumethiazide | 1 | 0 |
| 20057 | 64530020 | Methyclothiazide 5mg Tablet | Methyclothiazide | 1 |  |
| 18267 | 64533020 | Enduron 5mg Tablet (Abbott Laboratories Ltd) | Methyclothiazide | 1 |  |
| 28738 | 64556020 | Methyldopa with hydrochlorothiazide Tablet | Methyldopa Anhydrous/Hydrochlorothiazide | 0 | 1 |
| 21346 | 64564020 | Hydromet Tablet (MSD Thomas Morson Pharmaceuticals) | Methyldopa Anhydrous/Hydrochlorothiazide | 0 | 1 |
| 19352 | 71715020 | Xuret 0.5mg Tablet (Galen Ltd) | Metolazone | 1 |  |
| 61846 | 45437020 | Zaroxolyn 2.5mg tablets (IDIS) | Metolazone | 1 |  |
| 8602 | 50380020 | Metenix 5mg tablets (Sanofi) | Metolazone | 1 |  |
| 49752 | 33486020 | Metolazone 2.5mg/5ml oral solution | Metolazone | 1 |  |
| 4334 | 63211020 | Metolazone 500microgram low dose Tablet | Metolazone | 1 |  |
| 54643 | 94928020 | Metolazone Oral solution | Metolazone | 1 |  |
| 53674 | 45436020 | Metolazone 2.5mg tablets | Metolazone | 1 |  |
| 54329 | 33496020 | Metolazone 5mg/5ml oral suspension | Metolazone | 1 |  |
| 68432 | 33488020 | Metolazone 2.5mg/5ml oral suspension | Metolazone | 1 |  |
| 4332 | 64663020 | Metolazone 5mg tablets | Metolazone | 1 |  |
| 55777 | 33494020 | Metolazone 5mg/5ml oral solution | Metolazone | 1 |  |
| 69009 | 45448020 | Zaroxolyn 5mg tablets (Imported (Canada)) | Metolazone | 1 |  |
| 8147 | 50252020 | Lopresoretic Tablet (Novartis Pharmaceuticals UK Ltd) | Metoprolol Tartrate/Chlortalidone | 0 | 1 |
| 15488 | 68972020 | Metoprolol tartrate with chlortalidone Tablet | Metoprolol Tartrate/Chlortalidone | 0 | 1 |
| 7066 | 68976020 | Metoprolol 100mg / Hydrochlorothiazide 12.5mg tablets | Metoprolol tartrate/Hydrochlorothiazide | 0 | 1 |
| 33659 | 68979020 | Hydrochlorothiazide with metoprolol tartrate 25mg with 200mg Modified-release tablet | Metoprolol Tartrate/Hydrochlorothiazide | 0 | 1 |
| 29427 | 68980020 | Hydrochlorothiazide with metoprolol tartrate 12.5mg with 100mg tablet | Metoprolol Tartrate/Hydrochlorothiazide | 0 | 1 |
| 20093 | 68975020 | Metoprolol 200mg modified-release / Hydrochlorothiazide 25mg tablets | Metoprolol tartrate/Hydrochlorothiazide | 0 | 1 |
| 18287 | 53671020 | Co-Betaloc SA tablets (Pfizer Ltd) | Metoprolol tartrate/Hydrochlorothiazide | 0 | 1 |
| 10627 | 48722020 | Co-Betaloc tablets (Pfizer Ltd) | Metoprolol tartrate/Hydrochlorothiazide | 0 | 1 |
| 11338 | 67627020 | Bendroflumethiazide 5mg with Nadolol 40mg tablets | Nadolol/Bendroflumethiazide | 0 | 1 |
| 23134 | 64899020 | Nadolol 40mg / Bendroflumethiazide 5mg tablets | Nadolol/Bendroflumethiazide | 0 | 1 |
| 14438 | 54543020 | Corgaretic 80mg tablets (Sanofi-Synthelabo Ltd) | Nadolol/Bendroflumethiazide | 0 | 1 |
| 27946 | 64900020 | Nadolol 80mg / Bendroflumethiazide 5mg tablets | Nadolol/Bendroflumethiazide | 0 | 1 |
| 69334 | 67628020 | Bendroflumethiazide 5mg with Nadolol 80mg tablets | Nadolol/Bendroflumethiazide | 0 | 1 |
| 5330 | 48767020 | Corgaretic 40mg tablets (Sanofi-Synthelabo Ltd) | Nadolol/Bendroflumethiazide | 0 | 1 |
| 29634 | 90635020 | Olmetec Plus 20mg/12.5mg tablets (Daiichi Sankyo UK Ltd) | Olmesartan medoxomil/Hydrochlorothiazide | 0 | 1 |
| 18200 | 90629020 | Olmesartan medoxomil 20mg / Hydrochlorothiazide 12.5mg tablets | Olmesartan medoxomil/Hydrochlorothiazide | 0 | 1 |
| 8673 | 73918020 | Oxprenolol with cyclopenthiazide 160mg+0.25mg Modified-release tablet | Oxprenolol Hydrochloride/Cyclopenthiazide | 0 | 1 |
| 13871 | 73921020 | Co-prenozide 160mg/0.25mg modified-release tablets | Oxprenolol hydrochloride/Cyclopenthiazide | 0 | 1 |
| 52145 | 61691020 | Cyclopenthiazide 0.25mg with oxprenolol 160mg modified-release tablets | Oxprenolol Hydrochloride/Cyclopenthiazide | 0 | 1 |
| 4429 | 51994020 | Trasidrex modified-release tablets (Mercury Pharma Group Ltd) | Oxprenolol hydrochloride/Cyclopenthiazide | 0 | 1 |
| 24832 | 52999020 | Lasipressin Tablet (Hoechst UK Ltd) | Penbutolol/Furosemide | 0 | 1 |
| 26529 | 62151020 | Furosemide with penbutolol Tablet | Penbutolol/Furosemide | 0 | 1 |
| 51258 | 34961020 | Coversyl Arginine Plus 5mg/1.25mg tablets (DE Pharmaceuticals) | Perindopril arginine/Indapamide | 0 | 1 |
| 37978 | 94277020 | Perindopril arginine 5mg / Indapamide 1.25mg tablets | Perindopril arginine/Indapamide | 0 | 1 |
| 37908 | 94709020 | Coversyl Arginine Plus 5mg/1.25mg tablets (Servier Laboratories Ltd) | Perindopril arginine/Indapamide | 0 | 1 |
| 50607 | 92909020 | Perindopril arginine 2mg with Indapamide 625 micrograms tablet | Perindopril Erbumine/Indapamide | 0 | 1 |
| 48098 | 99501020 | Perindopril arginine 4mg with Indapamide 1.25mg tablet | Perindopril Erbumine/Indapamide | 0 | 1 |
| 25462 | 61503020 | Clopamide 5mg with Pindolol 10mg tablets | Pindolol/Clopamide | 0 | 1 |
| 12367 | 65855020 | Piretanide 6mg capsule | Piretanide | 1 |  |
| 7709 | 53005020 | Arelix 6mg Capsule (Hoechst Marion Roussel) | Piretanide | 1 |  |
| 12926 | 65972020 | Polythiazide 1mg tablets | Polythiazide | 1 |  |
| 12360 | 50657020 | Nephril 1mg Tablet (Pfizer Ltd) | Polythiazide | 1 |  |
| 8891 | 49876020 | Hygroton -k Tablet (Novartis Pharmaceuticals UK Ltd) | Potassium Chloride/Chlortalidone | 1 |  |
| 22839 | 79106020 | Clopamide with Potassium effervescent tablets | Potassium Chloride/Clopamide | 1 |  |
| 18983 | 48459020 | Brinaldix k Effervescent tablet (Berk Pharmaceuticals Ltd) | Potassium Chloride/Clopamide | 1 |  |
| 1125 | 50636020 | Navidrex -k Tablet (Novartis Pharmaceuticals UK Ltd) | Potassium Chloride/Cyclopenthiazide | 1 |  |
| 2833 | 61687020 | CYCLOPENTHIAZIDE -K tablets | Potassium Chloride/Cyclopenthiazide | 1 |  |
| 17960 | 62139020 | Furosemide 20mg / Potassium chloride 750mg (potassium 10mmol) modified-release tablets | Potassium chloride/Furosemide | 1 |  |
| 7734 | 49059020 | Diumide-K Continus tablets (Teofarma) | Potassium chloride/Furosemide | 1 |  |
| 8102 | 62138020 | Furosemide 40mg / Potassium chloride 600mg (potassium 8mmol) modified-release tablets | Potassium chloride/Furosemide | 1 |  |
| 7582 | 50100020 | Lasikal modified-release tablets (Borg Medicare) | Potassium chloride/Furosemide | 1 |  |
| 10781 | 52640020 | Lasix with k Tablet (Hoechst Marion Roussel) | Potassium chloride/Furosemide | 1 |  |
| 36519 | 49326020 | Esidrex -k Tablet (Novartis Pharmaceuticals UK Ltd) | Potassium Chloride/Hydrochlorothiazide | 1 |  |
| 12054 | 69745020 | Propranolol 80mg / Bendroflumethiazide 2.5mg capsules | Propranolol hydrochloride/Bendroflumethiazide | 0 | 1 |
| 4796 | 54256020 | Inderetic 80mg/2.5mg capsules (AstraZeneca UK Ltd) | Propranolol hydrochloride/Bendroflumethiazide | 0 | 1 |
| 23131 | 67621020 | Bendroflumethiazide 5mg with Propanolol 160mg modified-release capsules | Propranolol Hydrochloride/Bendroflumethiazide | 0 | 1 |
| 22912 | 67620020 | Bendroflumethiazide 2.5mg with Propanolol 80mg capsules | Propranolol Hydrochloride/Bendroflumethiazide | 0 | 1 |
| 8369 | 54259020 | Inderex 160mg/5mg modified-release capsules (AstraZeneca UK Ltd) | Propranolol hydrochloride/Bendroflumethiazide | 0 | 1 |
| 8987 | 69746020 | Propranolol 160mg modified-release / Bendroflumethiazide 5mg capsules | Propranolol hydrochloride/Bendroflumethiazide | 0 | 1 |
| 17783 | 57624020 | Spiroprop Tablet (Pharmacia Ltd) | Propranolol Hydrochloride/Spironolactone | 0 | 1 |
| 30129 | 47989020 | Abicol Tablet (Knoll Ltd) | Reserpine/Bendroflumethiazide | 0 | 1 |
| 19892 | 57809020 | Serpasil -esidrex Tablet (Novartis Pharmaceuticals UK Ltd) | Reserpine/Hydrochlorothiazide | 0 | 1 |
| 15493 | 66489020 | Reserpine with hydrochlorothiazide tablet | Reserpine/Hydrochlorothiazide | 0 | 1 |
| 3691 | 66694020 | Sotalol 160mg with hydrochlorothiazide 25mg tablet | Sotalol Hydrochloride/Hydrochlorothiazide | 0 | 1 |
| 8061 | 66693020 | Sotalol 80mg with hydrochlorothiazide 12.5mg tablet | Sotalol Hydrochloride/Hydrochlorothiazide | 0 | 1 |
| 15042 | 66704020 | Tolerzide Tablet (Bristol-Myers Squibb Pharmaceuticals Ltd) | Sotalol Hydrochloride/Hydrochlorothiazide | 0 | 1 |
| 12456 | 51646020 | Sotazide Tablet (Bristol-Myers Squibb Pharmaceuticals Ltd) | Sotalol Hydrochloride/Hydrochlorothiazide | 0 | 1 |
| 47018 | 517021 | Spironolactone 25mg/5ml oral suspension | Spironolactone | 1 |  |
| 34908 | 55251020 | Spironolactone 25mg tablets (IVAX Pharmaceuticals UK Ltd) | Spironolactone | 1 |  |
| 787 | 66713020 | Spironolactone 100mg capsule | Spironolactone | 1 |  |
| 17902 | 53136020 | Spirolone 100mg Tablet (Berk Pharmaceuticals Ltd) | Spironolactone | 1 |  |
| 57556 | 34605020 | Spironolactone 12mg/5ml oral solution | Spironolactone | 1 |  |
| 23091 | 68514020 | Spirospare 100 tablets (Ashbourne Pharmaceuticals Ltd) | Spironolactone | 1 |  |
| 54120 | 34806020 | Spironolactone 4mg/5ml oral suspension | Spironolactone | 1 |  |
| 41592 | 55623020 | Spironolactone 100mg tablets (Actavis UK Ltd) | Spironolactone | 1 |  |
| 11156 | 53134020 | Spirolone 25mg Tablet (Berk Pharmaceuticals Ltd) | Spironolactone | 1 |  |
| 12946 | 75249020 | Spironolactone 10mg/5ml oral suspension sugar free | Spironolactone | 1 |  |
| 43514 | 50618020 | Spironolactone 50mg tablets (A A H Pharmaceuticals Ltd) | Spironolactone | 1 |  |
| 32837 | 53610020 | Spironolactone 50mg tablets (Teva UK Ltd) | Spironolactone | 1 |  |
| 29397 | 59102020 | Spiretic 100mg Tablet (DDSA Pharmaceuticals Ltd) | Spironolactone | 1 |  |
| 11519 | 75250020 | Spironolactone 25mg/5ml oral suspension sugar free | Spironolactone | 1 |  |
| 51933 | 35256020 | Spironolactone 50mg/5ml oral solution | Spironolactone | 1 |  |
| 41660 | 53611020 | Spironolactone 100mg tablets (Teva UK Ltd) | Spironolactone | 1 |  |
| 35789 | 50611020 | Spironolactone 25mg Tablet (Celltech Pharma Europe Ltd) | Spironolactone | 1 |  |
| 46990 | 519021 | Spironolactone 50mg/5ml oral suspension | Spironolactone | 1 |  |
| 4161 | 58739020 | Spiroctan 25mg Tablet (Roche Products Ltd) | Spironolactone | 1 |  |
| 2142 | 59045020 | Spironolactone 100mg tablets | Spironolactone | 1 |  |
| 49388 | 20841020 | Spironolactone 100mg/5ml oral suspension | Spironolactone | 1 |  |
| 56274 | 34670020 | Spironolactone 4.5mg/5ml oral suspension | Spironolactone | 1 |  |
| 47687 | 59101020 | Spiretic 25mg Tablet (DDSA Pharmaceuticals Ltd) | Spironolactone | 1 |  |
| 61025 | 34621020 | Spironolactone 20mg/5ml oral solution | Spironolactone | 1 |  |
| 34296 | 50617020 | Spironolactone 25mg tablets (A A H Pharmaceuticals Ltd) | Spironolactone | 1 |  |
| 65582 | 34664020 | Spironolactone 3mg/5ml oral solution | Spironolactone | 1 |  |
| 51652 | 755020 | Spironolactone 25mg tablets (DE Pharmaceuticals) | Spironolactone | 1 |  |
| 4960 | 57448020 | Aldactone 50mg tablets (Pfizer Ltd) | Spironolactone | 1 |  |
| 31529 | 53609020 | Spironolactone 25mg tablets (Teva UK Ltd) | Spironolactone | 1 |  |
| 25494 | 57612020 | Diatensec 50mg Tablet (Pharmacia Ltd) | Spironolactone | 1 |  |
| 69473 | 34654020 | Spironolactone 3.5mg/5ml oral suspension | Spironolactone | 1 |  |
| 46674 | 55570020 | Spironolactone 50mg/5ml Oral suspension sugar free (Rosemont Pharmaceuticals Ltd) | Spironolactone | 1 |  |
| 692 | 59043020 | Spironolactone 25mg tablets | Spironolactone | 1 |  |
| 13264 | 90717020 | Spironolactone 15mg/5ml oral suspension | Spironolactone | 1 |  |
| 708 | 59044020 | Spironolactone 50mg tablets | Spironolactone | 1 |  |
| 19195 | 55626020 | Spironolactone 50mg Tablet (Wyeth Pharmaceuticals) | Spironolactone | 1 |  |
| 71010 | 40499020 | Spironolactone 25mg tablets (Genesis Pharmaceuticals Ltd) | Spironolactone | 1 |  |
| 58077 | 34866020 | Spironolactone 8mg/5ml oral suspension | Spironolactone | 1 |  |
| 58757 | 34623020 | Spironolactone 20mg/5ml oral suspension | Spironolactone | 1 |  |
| 58225 | 34615020 | Spironolactone 2.5mg/5ml oral suspension | Spironolactone | 1 |  |
| 63309 | 34846020 | Spironolactone 6mg/5ml oral suspension | Spironolactone | 1 |  |
| 60660 | 34666020 | Spironolactone 3mg/5ml oral suspension | Spironolactone | 1 |  |
| 50079 | 20835020 | Spironolactone 10mg/5ml oral suspension | Spironolactone | 1 |  |
| 52970 | 35252020 | Spironolactone 10mg/5ml oral solution | Spironolactone | 1 |  |
| 10214 | 75248020 | Spironolactone 5mg/5ml oral suspension sugar free | Spironolactone | 1 |  |
| 52366 | 35258020 | Spironolactone 5mg/5ml oral solution | Spironolactone | 1 |  |
| 21911 | 68513020 | Spirospare 25mg Tablet (Ashbourne Pharmaceuticals Ltd) | Spironolactone | 1 |  |
| 7991 | 58741020 | Spiroctan 100mg Capsule (Roche Products Ltd) | Spironolactone | 1 |  |
| 60343 | 752020 | Spironolactone 25mg tablets (Kent Pharmaceuticals Ltd) | Spironolactone | 1 |  |
| 2389 | 57447020 | Aldactone 25mg tablets (Pfizer Ltd) | Spironolactone | 1 |  |
| 56536 | 35250020 | Spironolactone 100mg/5ml oral solution | Spironolactone | 1 |  |
| 31219 | 50619020 | Spironolactone 100mg tablets (A A H Pharmaceuticals Ltd) | Spironolactone | 1 |  |
| 45078 | 55567020 | Spironolactone 25mg/5ml Oral solution sugar free (Rosemont Pharmaceuticals Ltd) | Spironolactone | 1 |  |
| 14109 | 75281020 | Spironolactone 100mg/5ml oral solution sugar free | Spironolactone | 1 |  |
| 41074 | 69121020 | Spironolactone 25mg tablets (Almus Pharmaceuticals Ltd) | Spironolactone | 1 |  |
| 57104 | 34619020 | Spironolactone 200mg/5ml oral suspension | Spironolactone | 1 |  |
| 6815 | 75280020 | Spironolactone 50mg/5ml oral suspension sugar free | Spironolactone | 1 |  |
| 53253 | 20821020 | Spironolactone 50mg/5ml oral suspension (Drug Tariff Special Order) | Spironolactone | 1 |  |
| 41706 | 55250020 | Spironolactone 50mg tablets (IVAX Pharmaceuticals UK Ltd) | Spironolactone | 1 |  |
| 50370 | 20819020 | Spironolactone 5mg/5ml oral suspension | Spironolactone | 1 |  |
| 57933 | 34802020 | Spironolactone 40mg/5ml oral suspension | Spironolactone | 1 |  |
| 17950 | 53135020 | Spirolone 50mg Tablet (Berk Pharmaceuticals Ltd) | Spironolactone | 1 |  |
| 67913 | 758020 | Spironolactone 50mg tablets (Kent Pharmaceuticals Ltd) | Spironolactone | 1 |  |
| 7952 | 57449020 | Aldactone 100mg tablets (Pfizer Ltd) | Spironolactone | 1 |  |
| 65822 | 34599020 | Spironolactone 12.5mg/5ml oral suspension | Spironolactone | 1 |  |
| 56067 | 34804020 | Spironolactone 4mg/5ml oral solution | Spironolactone | 1 |  |
| 51720 | 35254020 | Spironolactone 25mg/5ml oral solution | Spironolactone | 1 |  |
| 15052 | 58740020 | Spiroctan 50mg Tablet (Roche Products Ltd) | Spironolactone | 1 |  |
| 34347 | 55621020 | Spironolactone 25mg tablets (Actavis UK Ltd) | Spironolactone | 1 |  |
| 53508 | 31962020 | Spironolactone 5mg/5ml / Chlorothiazide 50mg/5ml oral suspension | Spironolactone/Chlorothiazide | 0 | 1 |
| 4661 | 66726020 | Spironolactone 50mg / Furosemide 20mg capsules | Spironolactone/Furosemide | 0 | 1 |
| 7441 | 50105020 | Lasilactone 20mg/50mg capsules (Sanofi) | Spironolactone/Furosemide | 0 | 1 |
| 45916 | 63283020 | Hydroflumethiazide with spironolactone 50mg+50mg Tablet | Spironolactone/Hydroflumethiazide | 0 | 1 |
| 29529 | 63282020 | Hydroflumethiazide with spironolactone 25mg+25mg Tablet | Spironolactone/Hydroflumethiazide | 0 | 1 |
| 25505 | 75350020 | Spiro-co 50mg+50mg Tablet (IVAX Pharmaceuticals UK Ltd) | Spironolactone/Hydroflumethiazide | 0 | 1 |
| 8521 | 66716020 | Spironolactone 25mg with hydroflumethiazide 25mg tablet | Spironolactone/Hydroflumethiazide | 0 | 1 |
| 31131 | 75349020 | Spiro-co 25mg+25mg Tablet (IVAX Pharmaceuticals UK Ltd) | Spironolactone/Hydroflumethiazide | 0 | 1 |
| 7961 | 66717020 | Spironolactone 50mg with hydroflumethiazide 50mg tablet | Spironolactone/Hydroflumethiazide | 0 | 1 |
| 1297 | 57454020 | Aldactide 50 tablets (Pfizer Ltd) | Spironolactone/Hydroflumethiazide | 0 | 1 |
| 2001 | 57453020 | Aldactide 25 tablets (Pfizer Ltd) | Spironolactone/Hydroflumethiazide | 0 | 1 |
| 11384 | 73915020 | Co-flumactone 50mg/50mg tablets | Spironolactone/Hydroflumethiazide | 0 | 1 |
| 15811 | 73914020 | Co-flumactone 25mg/25mg tablets | Spironolactone/Hydroflumethiazide | 0 | 1 |
| 66997 | 52342021 | MicardisPlus 40mg/12.5mg tablets (Waymade Healthcare Plc) | Telmisartan/Hydrochlorothiazide | 0 | 1 |
| 16161 | 58831020 | Telmisartan 80mg / Hydrochlorothiazide 12.5mg tablets | Telmisartan/Hydrochlorothiazide | 0 | 1 |
| 62376 | 23794021 | Actelsar HCT 80mg/12.5mg tablets (Actavis UK Ltd) | Telmisartan/Hydrochlorothiazide | 0 | 1 |
| 14870 | 81237020 | Telmisartan 40mg / Hydrochlorothiazide 12.5mg tablets | Telmisartan/Hydrochlorothiazide | 0 | 1 |
| 17689 | 49280020 | MicardisPlus 80mg/12.5mg tablets (Boehringer Ingelheim Ltd) | Telmisartan/Hydrochlorothiazide | 0 | 1 |
| 18202 | 81233020 | MicardisPlus 40mg/12.5mg tablets (Boehringer Ingelheim Ltd) | Telmisartan/Hydrochlorothiazide | 0 | 1 |
| 63890 | 10554020 | MicardisPlus 80mg/12.5mg tablets (Waymade Healthcare Plc) | Telmisartan/Hydrochlorothiazide | 0 | 1 |
| 21182 | 69280020 | Hydrochlorothiazide with timolol and amiloride 25mg with 10mg with 2.5mg Tablet | Timolol Maleate/Amiloride Hydrochloride/Hydrochlorothiazide | 0 | 1 |
| 25730 | 69274020 | Timolol maleate with amiloride and hydrochlorothiazide Tablet | Timolol Maleate/Amiloride Hydrochloride/Hydrochlorothiazide | 0 | 1 |
| 30519 | 69277020 | Amiloride with timolol with hydrochlorothiazide tablets | Timolol Maleate/Amiloride Hydrochloride/Hydrochlorothiazide | 0 | 1 |
| 48745 | 37067020 | Timolol 10mg / Amiloride 2.5mg / Hydrochlorothiazide 25mg tablets | Timolol maleate/Amiloride hydrochloride/Hydrochlorothiazide | 0 | 1 |
| 12651 | 67081020 | Timolol 10mg / Bendroflumethiazide 2.5mg tablets | Timolol maleate/Bendroflumethiazide | 0 | 1 |
| 12517 | 67082020 | Timolol maleate with bendroflumethiazide 20mg + 5mg Tablet | Timolol Maleate/Bendroflumethiazide | 0 | 1 |
| 21025 | 67090020 | Prestim forte Tablet (LEO Pharma) | Timolol Maleate/Bendroflumethiazide | 0 | 1 |
| 19142 | 67631020 | Bendroflumethiazide 2.5mg with Timolol maleate 10mg tablets | Timolol Maleate/Bendroflumethiazide | 0 | 1 |
| 25363 | 88272020 | Prestim tablets (Meda Pharmaceuticals Ltd) | Timolol maleate/Bendroflumethiazide | 0 | 1 |
| 8623 | 52834020 | Prestim Tablet (ICN Pharmaceuticals France S.A.) | Timolol maleate/Bendroflumethiazide | 0 | 1 |
| 4605 | 54433020 | Moducren tablets (Merck Sharp & Dohme Ltd) | Timolol maleate/Hydrochlorothiazide/Amiloride hydrochloride | 0 | 1 |
| 18096 | 75561020 | Torasemide 10mg tablets | Torasemide | 1 |  |
| 40898 | 67325020 | Torasemide 5mg tablets (A A H Pharmaceuticals Ltd) | Torasemide | 1 |  |
| 8052 | 75560020 | Torasemide 5mg tablets | Torasemide | 1 |  |
| 11487 | 75559020 | Torasemide 2.5mg tablets | Torasemide | 1 |  |
| 11268 | 75554020 | Torem 2.5mg tablets (Meda Pharmaceuticals Ltd) | Torasemide | 1 |  |
| 10066 | 75555020 | Torem 5mg tablets (Meda Pharmaceuticals Ltd) | Torasemide | 1 |  |
| 46525 | 67150020 | Torasemide 5mg tablets (Teva UK Ltd) | Torasemide | 1 |  |
| 22658 | 75556020 | Torem 10mg tablets (Meda Pharmaceuticals Ltd) | Torasemide | 1 |  |
| 4068 | 49169020 | Dytac 50mg capsules (AMCo) | Triamterene | 1 |  |
| 2179 | 67201020 | Triamterene 50mg capsules | Triamterene | 1 |  |
| 7136 | 49174020 | Dytide capsules (Mercury Pharma Group Ltd) | Triamterene/Benzthiazide | 0 | 1 |
| 7740 | 67210020 | Triamterene 50mg / Benzthiazide 25mg capsules | Triamterene/Benzthiazide | 0 | 1 |
| 37294 | 67220020 | Triamterene with chlortalidone 50mg + 25mg Tablet | Triamterene/Chlortalidone | 0 | 1 |
| 28157 | 53155020 | Kalspare ls Tablet (Dominion Pharma) | Triamterene/Chlortalidone | 0 | 1 |
| 3050 | 62148020 | Furosemide with triamterene 40mgwith50mg Tablet | Triamterene/Furosemide | 0 | 1 |
| 11265 | 67213020 | Triamterene 50mg / Furosemide 40mg tablets | Triamterene/Furosemide | 0 | 1 |
| 2961 | 49564020 | Frusene 50mg/40mg tablets (Orion Pharma (UK) Ltd) | Triamterene/Furosemide | 0 | 1 |
| 9223 | 67204020 | Triamterene with hydrochlorothiazide 50mg + 25mg Tablet | Triamterene/Hydrochlorothiazide | 0 | 1 |
| 15127 | 63257020 | Hydrochlorothiazide with triamterene 25mgwith50mg Tablet | Triamterene/Hydrochlorothiazide | 0 | 1 |
| 67664 | 10168020 | Valsartan 160mg / Hydrochlorothiazide 12.5mg tablets (Teva UK Ltd) | Valsartan/Hydrochlorothiazide | 0 | 1 |
| 11864 | 87994020 | Valsartan 160mg / Hydrochlorothiazide 12.5mg tablets | Valsartan/Hydrochlorothiazide | 0 | 1 |
| 25382 | 88008020 | Co-Diovan 160mg/25mg tablets (Novartis Pharmaceuticals UK Ltd) | Valsartan/Hydrochlorothiazide | 0 | 1 |
| 24268 | 88422020 | Hydrochlorothiazide with valsartan 12.5mg with 80mg Tablet | Valsartan/Hydrochlorothiazide | 0 | 1 |
| 23456 | 88006020 | Hydrochlorothiazide with valsartan 25mg with 160mg Tablet | Valsartan/Hydrochlorothiazide | 0 | 1 |
| 24484 | 87996020 | Hydrochlorothiazide with valsartan 12.5mg with 160mg Tablet | Valsartan/Hydrochlorothiazide | 0 | 1 |
| 14283 | 88002020 | Valsartan 160mg / Hydrochlorothiazide 25mg tablets | Valsartan/Hydrochlorothiazide | 0 | 1 |
| 6877 | 87998020 | Co-Diovan 160mg/12.5mg tablets (Novartis Pharmaceuticals UK Ltd) | Valsartan/Hydrochlorothiazide | 0 | 1 |
| 4044 | 49062020 | Diurexan 20mg tablets (Meda Pharmaceuticals Ltd) | Xipamide | 1 |  |
| 7618 | 65442020 | Xipamide 20mg tablets | Xipamide | 1 |  |

**Supplementary Table 10. Code list for CCB**

| **CCB** | | | |  |  |  | | |  |  |
| --- | --- | --- | --- | --- | --- | --- | --- | --- | --- | --- |
| **Prod**  **code** | | | | **Gemscript**  **code** | **Product**  **name** | **Drug**  **substance**  **name** | | | **To include** | **combination therapy to exclude** |
| 46233 | | | | 91269020 | Amlodipine Oral solution | Amlodipine | | | 1 |  |
| 31761 | | | | 88254020 | Amlostin 10mg tablets (Discovery Pharmaceuticals) | Amlodipine | | | 1 |  |
| 67662 | | | | 2012020 | Istin 10mg tablets (DE Pharmaceuticals) | Amlodipine | | | 1 |  |
| 17640 | | | | 88252020 | Amlostin 5mg tablets (Discovery Pharmaceuticals) | Amlodipine | | | 1 |  |
| 61422 | | | | 2002020 | Amlodipine 5mg tablets (Accord Healthcare Ltd) | Amlodipine | | | 1 |  |
| 56147 | | | | 2023020 | Amlodipine 10mg tablets (Accord Healthcare Ltd) | Amlodipine | | | 1 |  |
| 29 | | | | 72826020 | Amlodipine besilate 5mg tablets | Amlodipine | | | 1 |  |
| 59001 | | | | 68889020 | Amlodipine 10mg tablets (Mylan) | Amlodipine | | | 1 |  |
| 54696 | | | | 68498020 | Amlodipine 10mg tablets (Sandoz Ltd) | Amlodipine | | | 1 |  |
| 54654 | | | | 75178020 | Amlodipine 10mg tablets (Almus Pharmaceuticals Ltd) | Amlodipine | | | 1 |  |
| 63515 | | | | 70042020 | Amlodipine 10mg tablets (Dr Reddy's Laboratories (UK) Ltd) | Amlodipine | | | 1 |  |
| 39914 | | | | 67735020 | Amlodipine 5mg tablets (Teva UK Ltd) | Amlodipine | | | 1 |  |
| 749 | | | | 88863020 | Amlodipine 5mg tablets | Amlodipine | | | 1 |  |
| 70999 | | | | 38961020 | Amlodipine 5mg tablets (APC Pharmaceuticals & Chemicals (Europe) Ltd) | Amlodipine | | | 1 |  |
| 64623 | | | | 52580021 | Amlodipine 10mg/5ml oral solution sugar free | Amlodipine | | | 1 |  |
| 64606 | | | | 70110020 | Amlodipine 5mg tablets (Focus Pharmaceuticals Ltd) | Amlodipine | | | 1 |  |
| 43880 | | | | 75222020 | Amlodipine 5mg tablets (Almus Pharmaceuticals Ltd) | Amlodipine | | | 1 |  |
| 729 | | | | 87704020 | Amlodipine maleate 5mg tablets | Amlodipine | | | 1 |  |
| 6856 | | | | 88865020 | Amlodipine 10mg tablets | Amlodipine | | | 1 |  |
| 61374 | | | | 29717020 | Amlodipine 4mg/5ml oral suspension | Amlodipine | | | 1 |  |
| 68311 | | | | 53056021 | Amlodipine 5mg/5ml oral solution sugar free (A A H Pharmaceuticals Ltd) | Amlodipine | | | 1 |  |
| 64441 | | | | 1986020 | Amlodipine 5mg tablets (Alliance Healthcare (Distribution) Ltd) | Amlodipine | | | 1 |  |
| 52440 | | | | 41782020 | Amlodipine 10mg/5ml oral solution | Amlodipine | | | 1 |  |
| 66574 | | | | 2000020 | Amlodipine 5mg tablets (Phoenix Healthcare Distribution Ltd) | Amlodipine | | | 1 |  |
| 32917 | | | | 67688020 | Amlodipine 5mg tablets (IVAX Pharmaceuticals UK Ltd) | Amlodipine | | | 1 |  |
| 6477 | | | | 87706020 | Amlodipine maleate 10mg tablets | Amlodipine | | | 1 |  |
| 42210 | | | | 68111020 | Amlodipine 10mg tablets (Zentiva) | Amlodipine | | | 1 |  |
| 59762 | | | | 67739020 | Amlodipine 10mg tablets (Teva UK Ltd) | Amlodipine | | | 1 |  |
| 39804 | | | | 70039020 | Amlodipine 5mg tablets (Dr Reddy's Laboratories (UK) Ltd) | Amlodipine | | | 1 |  |
| 58580 | | | | 38962020 | Amlodipine 10mg tablets (APC Pharmaceuticals & Chemicals (Europe) Ltd) | Amlodipine | | | 1 |  |
| 68221 | | | | 2007020 | Amlodipine 10mg tablets (Kent Pharmaceuticals Ltd) | Amlodipine | | | 1 |  |
| 49636 | | | | 38506020 | Amlodipine 10mg tablets (DE Pharmaceuticals) | Amlodipine | | | 1 |  |
| 3917 | | | | 72822020 | Istin 5mg tablets (Pfizer Ltd) | Amlodipine | | | 1 |  |
| 66430 | | | | 1985020 | Amlodipine 5mg tablets (Kent Pharmaceuticals Ltd) | Amlodipine | | | 1 |  |
| 45279 | | | | 68495020 | Amlodipine 5mg tablets (Sandoz Ltd) | Amlodipine | | | 1 |  |
| 45070 | | | | 99159020 | Amlodipine 10mg/5ml oral suspension | Amlodipine | | | 1 |  |
| 71 | | | | 72827020 | Amlodipine besilate 10mg tablets | Amlodipine | | | 1 |  |
| 32595 | | | | 67634020 | Amlodipine 5mg tablets (A A H Pharmaceuticals Ltd) | Amlodipine | | | 1 |  |
| 64418 | | | | 14479021 | Amlodipine 5mg tablets (Waymade Healthcare Plc) | Amlodipine | | | 1 |  |
| 16162 | | | | 91263020 | Amlodipine 5mg/5ml oral suspension | Amlodipine | | | 1 |  |
| 47002 | | | | 413021 | Amlodipine 10mg/5ml sugar free Oral suspension | Amlodipine | | | 1 |  |
| 54983 | | | | 18978020 | Amlodipine 2.5mg/5ml oral suspension | Amlodipine | | | 1 |  |
| 65745 | | | | 38505020 | Amlodipine 5mg tablets (DE Pharmaceuticals) | Amlodipine | | | 1 |  |
| 34093 | | | | 67637020 | Amlodipine 10mg tablets (A A H Pharmaceuticals Ltd) | Amlodipine | | | 1 |  |
| 46724 | | | | 415021 | Amlodipine 5mg/5ml oral solution | Amlodipine | | | 1 |  |
| 64327 | | | | 68885020 | Amlodipine 5mg tablets (Mylan) | Amlodipine | | | 1 |  |
| 60244 | | | | 2021020 | Amlodipine 10mg tablets (Phoenix Healthcare Distribution Ltd) | Amlodipine | | | 1 |  |
| 43470 | | | | 75838020 | Amlodipine 5mg tablets (Wockhardt UK Ltd) | Amlodipine | | | 1 |  |
| 56334 | | | | 2018020 | Amlodipine 10mg tablets (Bristol Laboratories Ltd) | Amlodipine | | | 1 |  |
| 36202 | | | | 68410020 | Amlodipine 10mg tablets (Actavis UK Ltd) | Amlodipine | | | 1 |  |
| 64447 | | | | 2003020 | Amlodipine 5mg tablets (Somex Pharma) | Amlodipine | | | 1 |  |
| 53868 | | | | 68406020 | Amlodipine 5mg tablets (Actavis UK Ltd) | Amlodipine | | | 1 |  |
| 66817 | | | | 75843020 | Amlodipine 10mg tablets (Wockhardt UK Ltd) | Amlodipine | | | 1 |  |
| 54515 | | | | 2008020 | Amlodipine 10mg tablets (Alliance Healthcare (Distribution) Ltd) | Amlodipine | | | 1 |  |
| 54633 | | | | 1996020 | Amlodipine 5mg tablets (Bristol Laboratories Ltd) | Amlodipine | | | 1 |  |
| 5914 | | | | 72823020 | Istin 10mg tablets (Pfizer Ltd) | Amlodipine | | | 1 |  |
| 64166 | | | | 52583021 | Amlodipine 5mg/5ml oral solution sugar free | Amlodipine | | | 1 |  |
| 47616 | | | | 99785020 | Sevikar HCT 40mg/10mg/12.5mg tablets (Daiichi Sankyo UK Ltd) | Amlodipine besilate/Hydrochlorothiazide/Olmesartan medoxomil | | | 0 | 1 |
| 47573 | | | | 99783020 | Sevikar HCT 40mg/5mg/12.5mg tablets (Daiichi Sankyo UK Ltd) | Amlodipine besilate/Hydrochlorothiazide/Olmesartan medoxomil | | | 0 | 1 |
| 60007 | | | | 38012020 | Generic Sevikar HCT 40mg/10mg/12.5mg tablets | Amlodipine besilate/Hydrochlorothiazide/Olmesartan medoxomil | | | 0 | 1 |
| 60744 | | | | 22980021 | Perindopril erbumine 8mg / Amlodipine 5mg tablets | Amlodipine besilate/Perindopril erbumine | | | 0 | 1 |
| 63149 | | | | 22978021 | Perindopril erbumine 8mg / Amlodipine 10mg tablets | Amlodipine besilate/Perindopril erbumine | | | 0 | 1 |
| 35304 | | | | 92957020 | Valsartan 160mg with amlodipine 10mg tablets | Amlodipine Besilate/Valsartan | | | 0 | 1 |
| 35189 | | | | 92767020 | Amlodipine 10mg / Valsartan 160mg tablets | Amlodipine besilate/Valsartan | | | 0 | 1 |
| 35096 | | | | 92773020 | Exforge 10mg/160mg tablets (Novartis Pharmaceuticals UK Ltd) | Amlodipine besilate/Valsartan | | | 0 | 1 |
| 35329 | | | | 92763020 | Amlodipine 5mg / Valsartan 80mg tablets | Amlodipine besilate/Valsartan | | | 0 | 1 |
| 35173 | | | | 92953020 | Valsartan 160mg with amlodipine 5mg tablets | Amlodipine Besilate/Valsartan | | | 0 | 1 |
| 35317 | | | | 92769020 | Exforge 5mg/80mg tablets (Novartis Pharmaceuticals UK Ltd) | Amlodipine besilate/Valsartan | | | 0 | 1 |
| 35174 | | | | 92951020 | Valsartan 80mg with amlodipine 5mg tablets | Amlodipine Besilate/Valsartan | | | 0 | 1 |
| 55358 | | | | 99777020 | Olmesartan medoxomil with amlodipine and hydrochlorothiazide 40mg + 10mg + 25mg Tablet | Amlodipine/Hydrochlorothiazide/Olmesartan Medoxomil | | | 0 | 1 |
| 46687 | | | | 99769020 | Olmesartan medoxomil with amlodipine and hydrochlorothiazide 20mg + 5mg + 12.5mg Tablet | Amlodipine/Hydrochlorothiazide/Olmesartan Medoxomil | | | 0 | 1 |
| 46792 | | | | 99771020 | Olmesartan medoxomil with amlodipine and hydrochlorothiazide 40mg + 5mg + 12.5mg Tablet | Amlodipine/Hydrochlorothiazide/Olmesartan Medoxomil | | | 0 | 1 |
| 46715 | | | | 99773020 | Olmesartan medoxomil with amlodipine and hydrochlorothiazide 40mg + 10mg + 12.5mg Tablet | Amlodipine/Hydrochlorothiazide/Olmesartan Medoxomil | | | 0 | 1 |
| 47467 | | | | 99775020 | Olmesartan medoxomil with amlodipine and hydrochlorothiazide 40mg + 5mg + 25mg Tablet | Amlodipine/Hydrochlorothiazide/Olmesartan Medoxomil | | | 0 | 1 |
| 4542 | | | | 68847020 | Atenolol 50mg / Nifedipine 20mg modified-release capsules | Atenolol/Nifedipine | | | 0 | 1 |
| 52728 | | | | 1077020 | Beta-Adalat modified-release capsules (Lexon (UK) Ltd) | Atenolol/Nifedipine | | | 0 | 1 |
| 61719 | | | | 1072020 | Beta-Adalat modified-release capsules (Waymade Healthcare Plc) | Atenolol/Nifedipine | | | 0 | 1 |
| 8642 | | | | 68652020 | Tenif 50mg/20mg modified-release capsules (AstraZeneca UK Ltd) | Atenolol/Nifedipine | | | 0 | 1 |
| 68020 | | | | 1075020 | Beta-Adalat modified-release capsules (Sigma Pharmaceuticals Plc) | Atenolol/Nifedipine | | | 0 | 1 |
| 1684 | | | | 68629020 | Beta-Adalat modified-release capsules (Bayer Plc) | Atenolol/Nifedipine | | | 0 | 1 |
| 28949 | | | | 84959020 | Bi-carzem sr 120mg Modified-release capsule (Tillomed Laboratories Ltd) | Diltiazem Hydrochloride | | | 1 |  |
| 70961 | | | | 1872020 | Tildiem Retard 90mg tablets (DE Pharmaceuticals) | Diltiazem hydrochloride | | | 1 |  |
| 20642 | | | | 84957020 | Bi-carzem sr 60mg Modified-release capsule (Tillomed Laboratories Ltd) | Diltiazem hydrochloride | | | 1 |  |
| 56467 | | | | 1909020 | Tildiem 60mg modified-release tablets (DE Pharmaceuticals) | Diltiazem hydrochloride | | | 1 |  |
| 67890 | | | | 47165020 | Uard 300XL capsules (Ennogen Healthcare Ltd) | Diltiazem hydrochloride | | | 1 |  |
| 5513 | | | | 74596020 | Dilzem sr 60mg Capsule (Elan Pharma) | Diltiazem hydrochloride | | | 1 |  |
| 38964 | | | | 95553020 | Adizem-SR 120mg tablets (Napp Pharmaceuticals Ltd) | Diltiazem hydrochloride | | | 1 |  |
| 66834 | | | | 47170020 | Uard 240XL capsules (Ennogen Healthcare Ltd) | Diltiazem hydrochloride | | | 1 |  |
| 3676 | | | | 75265020 | Dilzem xl mr 180mg Modified-release capsule (Elan Pharma) | Diltiazem Hydrochloride | | | 1 |  |
| 27135 | | | | 68574020 | Diltiazem sr 90mg Capsule (Hillcross Pharmaceuticals Ltd) | Diltiazem hydrochloride | | | 1 |  |
| 66850 | | | | 27252021 | Diltiazem 240mg modified-release capsules (Icarus Pharmaceuticals Ltd) | Diltiazem hydrochloride | | | 1 |  |
| 2811 | | | | 57067020 | Adizem sr 180mg Modified-release capsule (Napp Pharmaceuticals Ltd) | Diltiazem Hydrochloride | | | 1 |  |
| 47415 | | | | 77105020 | Diltiazem sr 60mg Capsule (Hillcross Pharmaceuticals Ltd) | Diltiazem hydrochloride | | | 1 |  |
| 11922 | | | | 90541020 | Diltiazem 60mg/5ml oral suspension | Diltiazem hydrochloride | | | 1 |  |
| 34377 | | | | 60316020 | Diltiazem HCl 90mg Modified-release capsule (Hillcross Pharmaceuticals Ltd) | Diltiazem hydrochloride | | | 1 |  |
| 5296 | | | | 62587020 | Tildiem la 300mg Modified-release capsule (Sanofi) | Diltiazem hydrochloride | | | 1 |  |
| 26270 | | | | 77217020 | Optil xl 300mg Modified-release capsule (Opus Pharmaceuticals Ltd) | Diltiazem hydrochloride | | | 1 |  |
| 60620 | | | | 21730021 | Adizem-XL 240mg capsules (Waymade Healthcare Plc) | Diltiazem hydrochloride | | | 1 |  |
| 34581 | | | | 68132020 | Diltiazem HCl 60mg Modified-release tablet (Kent Pharmaceuticals Ltd) | Diltiazem hydrochloride | | | 1 |  |
| 1130 | | | | 84427020 | Viazem XL 300mg capsules (Thornton & Ross Ltd) | Diltiazem hydrochloride | | | 1 |  |
| 30197 | | | | 90599020 | Diltiazem 120mg modified-release capsules | Diltiazem hydrochloride | | | 1 |  |
| 7398 | | | | 84428020 | Viazem XL 360mg capsules (Thornton & Ross Ltd) | Diltiazem hydrochloride | | | 1 |  |
| 3061 | | | | 57178020 | Diltiazem 12hr 180mg modified-release capsules | Diltiazem Hydrochloride | | | 1 |  |
| 47732 | | | | 97275020 | Zemret 180 XL capsules (Tillomed Laboratories Ltd) | Diltiazem hydrochloride | | | 1 |  |
| 22619 | | | | 68606020 | Britiazim 60mg Modified-release tablet (Thames Laboratories Ltd) | Diltiazem hydrochloride | | | 1 |  |
| 8558 | | | | 75162020 | Adizem xl 120mg Capsule (Napp Pharmaceuticals Ltd) | Diltiazem Hydrochloride | | | 1 |  |
| 26309 | | | | 77216020 | Optil xl 240mg Modified-release capsule (Opus Pharmaceuticals Ltd) | Diltiazem hydrochloride | | | 1 |  |
| 15221 | | | | 75963020 | Dilcardia xl 180mg Modified-release capsule (Generics (UK) Ltd) | Diltiazem Hydrochloride | | | 1 |  |
| 4732 | | | | 72564020 | Diltiazem 90mg modified-release tablets | Diltiazem hydrochloride | | | 1 |  |
| 66172 | | | | 47169020 | Uard 180XL capsules (Ennogen Healthcare Ltd) | Diltiazem hydrochloride | | | 1 |  |
| 17666 | | | | 84422020 | Viazem XL 180mg capsules (Thornton & Ross Ltd) | Diltiazem hydrochloride | | | 1 |  |
| 45759 | | | | 65091020 | Diltiazem HCl 240mg Capsule (PLIVA Pharma Ltd) | Diltiazem hydrochloride | | | 1 |  |
| 21918 | | | | 85943020 | Optil 60mg modified-release tablets (Opus Pharmaceuticals Ltd) | Diltiazem hydrochloride | | | 1 |  |
| 23733 | | | | 69392020 | Optil sr 90mg Modified-release capsule (Opus Pharmaceuticals Ltd) | Diltiazem hydrochloride | | | 1 |  |
| 21763 | | | | 49566020 | Diltiazem 60mg modified-release tablets (A A H Pharmaceuticals Ltd) | Diltiazem hydrochloride | | | 1 |  |
| 18403 | | | | 60315020 | Diltiazem HCl 180mg Modified-release capsule (Hillcross Pharmaceuticals Ltd) | Diltiazem Hydrochloride | | | 1 |  |
| 69277 | | | | 52686021 | Adizem-XL 240mg capsules (Lexon (UK) Ltd) | Diltiazem hydrochloride | | | 1 |  |
| 793 | | | | 75164020 | Adizem xl 240mg Capsule (Napp Pharmaceuticals Ltd) | Diltiazem hydrochloride | | | 1 |  |
| 20890 | | | | 86888020 | Zemtard 240 XL capsules (Galen Ltd) | Diltiazem hydrochloride | | | 1 |  |
| 34475 | | | | 53802020 | Diltiazem HCl 90mg Modified-release tablet (IVAX Pharmaceuticals UK Ltd) | Diltiazem hydrochloride | | | 1 |  |
| 9374 | | | | 72559020 | Adizem 60mg Modified-release tablet (Napp Pharmaceuticals Ltd) | Diltiazem hydrochloride | | | 1 |  |
| 536 | | | | 62588020 | Tildiem la 200mg Modified-release capsule (Sanofi) | Diltiazem hydrochloride | | | 1 |  |
| 35696 | | | | 88983020 | Kenzem SR 120mg capsules (Kent Pharmaceuticals Ltd) | Diltiazem hydrochloride | | | 1 |  |
| 32262 | | | | 49558020 | Diltiazem HCl 60mg Tablet (C P Pharmaceuticals Ltd) | Diltiazem hydrochloride | | | 1 |  |
| 59863 | | | | 1895020 | Dilzem XL 240 capsules (Lexon (UK) Ltd) | Diltiazem hydrochloride | | | 1 |  |
| 66635 | | | | 1892020 | Dilzem XL 180 capsules (DE Pharmaceuticals) | Diltiazem hydrochloride | | | 1 |  |
| 11973 | | | | 79934020 | Calcicard CR 120mg tablets (Teva UK Ltd) | Diltiazem hydrochloride | | | 1 |  |
| 44192 | | | | 97277020 | Zemret 240 XL capsules (Tillomed Laboratories Ltd) | Diltiazem hydrochloride | | | 1 |  |
| 26759 | | | | 80908020 | Zildil SR 60mg capsules (Chanelle Medical UK Ltd) | Diltiazem hydrochloride | | | 1 |  |
| 25777 | | | | 86145020 | Dilcardia SR 120mg capsules (Mylan) | Diltiazem hydrochloride | | | 1 |  |
| 67344 | | | | 23042021 | Diltiazem 300mg modified-release capsules (Ennogen Pharma Ltd) | Diltiazem hydrochloride | | | 1 |  |
| 2592 | | | | 84421020 | Viazem XL 120mg capsules (Thornton & Ross Ltd) | Diltiazem hydrochloride | | | 1 |  |
| 9410 | | | | 79520020 | Angitil SR 120 capsules (Ethypharm UK Ltd) | Diltiazem hydrochloride | | | 1 |  |
| 18830 | | | | 82004020 | Disogram SR 90mg capsules (Ranbaxy (UK) Ltd) | Diltiazem hydrochloride | | | 1 |  |
| 57859 | | | | 47164020 | Diltiazem 90mg modified-release tablets (Cubic Pharmaceuticals Ltd) | Diltiazem hydrochloride | | | 1 |  |
| 5326 | | | | 75621020 | Diltiazem 24hr 300mg modified-release capsules | Diltiazem Hydrochloride | | | 1 |  |
| 4308 | | | | 74597020 | Dilzem sr 90mg Capsule (Elan Pharma) | Diltiazem hydrochloride | | | 1 |  |
| 13075 | | | | 87834020 | Dilzem XL 180 capsules (Teva UK Ltd) | Diltiazem hydrochloride | | | 1 |  |
| 3370 | | | | 75264020 | Dilzem xl mr 120mg Modified-release capsule (Elan Pharma) | Diltiazem Hydrochloride | | | 1 |  |
| 49001 | | | | 11003020 | Diltiazem 120mg modified-release tablets (Alliance Healthcare (Distribution) Ltd) | Diltiazem hydrochloride | | | 1 |  |
| 27136 | | | | 60317020 | Diltiazem 90mg modified-release tablets (A A H Pharmaceuticals Ltd) | Diltiazem hydrochloride | | | 1 |  |
| 13033 | | | | 86668020 | Angitil XL 240 capsules (Ethypharm UK Ltd) | Diltiazem hydrochloride | | | 1 |  |
| 60415 | | | | 1891020 | Dilzem XL 180 capsules (Sigma Pharmaceuticals Plc) | Diltiazem hydrochloride | | | 1 |  |
| 48282 | | | | 1882020 | Diltiazem 90mg modified-release capsules (A A H Pharmaceuticals Ltd) | Diltiazem hydrochloride | | | 1 |  |
| 38831 | | | | 95539020 | Adizem-SR 180mg capsules (Napp Pharmaceuticals Ltd) | Diltiazem hydrochloride | | | 1 |  |
| 1995 | | | | 57177020 | Diltiazem 12hr 120mg modified-release capsules | Diltiazem Hydrochloride | | | 1 |  |
| 18852 | | | | 86924020 | Disogram SR 120mg capsules (Ranbaxy (UK) Ltd) | Diltiazem hydrochloride | | | 1 |  |
| 27401 | | | | 88981020 | Kenzem SR 90mg capsules (Kent Pharmaceuticals Ltd) | Diltiazem hydrochloride | | | 1 |  |
| 62207 | | | | 21725021 | Adizem-SR 120mg capsules (Waymade Healthcare Plc) | Diltiazem hydrochloride | | | 1 |  |
| 11770 | | | | 87826020 | Dilzem SR 60 capsules (Teva UK Ltd) | Diltiazem hydrochloride | | | 1 |  |
| 26267 | | | | 69393020 | Optil sr 120mg Modified-release capsule (Opus Pharmaceuticals Ltd) | Diltiazem Hydrochloride | | | 1 |  |
| 34824 | | | | 53803020 | Diltiazem HCl 120mg Modified-release tablet (IVAX Pharmaceuticals UK Ltd) | Diltiazem hydrochloride | | | 1 |  |
| 29676 | | | | 80745020 | Calazem 60mg Modified-release tablet (Berk Pharmaceuticals Ltd) | Diltiazem hydrochloride | | | 1 |  |
| 13302 | | | | 87828020 | Dilzem SR 90 capsules (Teva UK Ltd) | Diltiazem hydrochloride | | | 1 |  |
| 13926 | | | | 75622020 | Diltiazem 360mg modified-release capsules | Diltiazem hydrochloride | | | 1 |  |
| 23233 | | | | 84958020 | Bi-carzem sr 90mg Modified-release capsule (Tillomed Laboratories Ltd) | Diltiazem hydrochloride | | | 1 |  |
| 5194 | | | | 74598020 | Dilzem sr 120mg Capsule (Elan Pharma) | Diltiazem Hydrochloride | | | 1 |  |
| 47724 | | | | 95577020 | Bi-Carzem XL 240mg capsules (Tillomed Laboratories Ltd) | Diltiazem hydrochloride | | | 1 |  |
| 66048 | | | | 11004020 | Tildiem Retard 120mg tablets (DE Pharmaceuticals) | Diltiazem hydrochloride | | | 1 |  |
| 38865 | | | | 95559020 | Adizem-XL 120mg capsules (Napp Pharmaceuticals Ltd) | Diltiazem hydrochloride | | | 1 |  |
| 27685 | | | | 65095020 | Diltiazem HCl 300mg Capsule (PLIVA Pharma Ltd) | Diltiazem hydrochloride | | | 1 |  |
| 21795 | | | | 50256020 | Retalzem 60 modified-release tablets (Kent Pharmaceuticals Ltd) | Diltiazem hydrochloride | | | 1 |  |
| 39171 | | | | 95543020 | Bi-Carzem SR 60mg capsules (Tillomed Laboratories Ltd) | Diltiazem hydrochloride | | | 1 |  |
| 19426 | | | | 79164020 | Disogram SR 240mg capsules (Ranbaxy (UK) Ltd) | Diltiazem hydrochloride | | | 1 |  |
| 37774 | | | | 88979020 | Kenzem SR 60mg capsules (Kent Pharmaceuticals Ltd) | Diltiazem hydrochloride | | | 1 |  |
| 26460 | | | | 75964020 | Dilcardia xl 240mg Modified-release capsule (Generics (UK) Ltd) | Diltiazem hydrochloride | | | 1 |  |
| 13410 | | | | 70830020 | Angiozem 60mg modified-release tablets (Ashbourne Pharmaceuticals Ltd) | Diltiazem hydrochloride | | | 1 |  |
| 69108 | | | | 52680021 | Adizem-XL 300mg capsules (Lexon (UK) Ltd) | Diltiazem hydrochloride | | | 1 |  |
| 52276 | | | | 39293020 | Adizem-XL 180mg capsules (DE Pharmaceuticals) | Diltiazem hydrochloride | | | 1 |  |
| 61532 | | | | 26725021 | Diltiazem 120mg modified-release capsules (Sigma Pharmaceuticals Plc) | Diltiazem hydrochloride | | | 1 |  |
| 6309 | | | | 57068020 | Adizem xl 300mg Capsule (Napp Pharmaceuticals Ltd) | Diltiazem hydrochloride | | | 1 |  |
| 26269 | | | | 69394020 | Optil sr 180mg Modified-release capsule (Opus Pharmaceuticals Ltd) | Diltiazem Hydrochloride | | | 1 |  |
| 49289 | | | | 1884020 | Diltiazem 120mg modified-release capsules (Alliance Healthcare (Distribution) Ltd) | Diltiazem hydrochloride | | | 1 |  |
| 41635 | | | | 53801020 | Diltiazem 60mg modified-release tablets (IVAX Pharmaceuticals UK Ltd) | Diltiazem hydrochloride | | | 1 |  |
| 17425 | | | | 84733020 | Zemtard 120 XL capsules (Galen Ltd) | Diltiazem hydrochloride | | | 1 |  |
| 17406 | | | | 84734020 | Zemtard 180 XL capsules (Galen Ltd) | Diltiazem hydrochloride | | | 1 |  |
| 4635 | | | | 72177020 | Diltiazem 200mg modified-release capsules | Diltiazem hydrochloride | | | 1 |  |
| 69028 | | | | 21729021 | Diltiazem 60mg modified-release capsules (DE Pharmaceuticals) | Diltiazem hydrochloride | | | 1 |  |
| 10267 | | | | 81943020 | Adizem-XL 200mg capsules (Napp Pharmaceuticals Ltd) | Diltiazem hydrochloride | | | 1 |  |
| 62065 | | | | 23040021 | Diltiazem 90mg modified-release tablets (Colorama Pharmaceuticals Ltd) | Diltiazem hydrochloride | | | 1 |  |
| 2663 | | | | 72565020 | Diltiazem 240mg modified-release capsules | Diltiazem Hydrochloride | | | 1 |  |
| 21778 | | | | 54633020 | Diltiazem 60mg modified-release tablets (Teva UK Ltd) | Diltiazem hydrochloride | | | 1 |  |
| 42731 | | | | 68577020 | Diltiazem sr 120mg Capsule (Hillcross Pharmaceuticals Ltd) | Diltiazem Hydrochloride | | | 1 |  |
| 1836 | | | | 71948020 | Diltiazem 60mg modified-release tablets | Diltiazem hydrochloride | | | 1 |  |
| 38882 | | | | 95563020 | Adizem-XL 240mg capsules (Napp Pharmaceuticals Ltd) | Diltiazem hydrochloride | | | 1 |  |
| 18975 | | | | 68163020 | Calcicard 60mg Tablet (3M Health Care Ltd) | Diltiazem hydrochloride | | | 1 |  |
| 636 | | | | 86603020 | Diltiazem 60mg modified-release capsules | Diltiazem hydrochloride | | | 1 |  |
| 54799 | | | | 1878020 | Tildiem LA 300 capsules (Mawdsley-Brooks & Company Ltd) | Diltiazem hydrochloride | | | 1 |  |
| 65636 | | | | 52683021 | Adizem-XL 120mg capsules (Lexon (UK) Ltd) | Diltiazem hydrochloride | | | 1 |  |
| 48457 | | | | 1881020 | Diltiazem 90mg modified-release capsules (Alliance Healthcare (Distribution) Ltd) | Diltiazem hydrochloride | | | 1 |  |
| 9708 | | | | 75520020 | Diltiazem 24hr 120mg modified-release capsules | Diltiazem Hydrochloride | | | 1 |  |
| 48288 | | | | 1886020 | Diltiazem 120mg modified-release capsules (A A H Pharmaceuticals Ltd) | Diltiazem hydrochloride | | | 1 |  |
| 65602 | | | | 21726021 | Adizem-XL 120mg capsules (Waymade Healthcare Plc) | Diltiazem hydrochloride | | | 1 |  |
| 52701 | | | | 1902020 | Tildiem LA 200 capsules (Mawdsley-Brooks & Company Ltd) | Diltiazem hydrochloride | | | 1 |  |
| 44887 | | | | 86212020 | Bi-carzem xl 300mg Capsule (Tillomed Laboratories Ltd) | Diltiazem hydrochloride | | | 1 |  |
| 4808 | | | | 75522020 | Diltiazem 240mg modified-release capsules | Diltiazem hydrochloride | | | 1 |  |
| 16038 | | | | 87830020 | Dilzem SR 120 capsules (Teva UK Ltd) | Diltiazem hydrochloride | | | 1 |  |
| 4408 | | | | 76295020 | Slozem 240mg capsules (Merck Serono Ltd) | Diltiazem hydrochloride | | | 1 |  |
| 2686 | | | | 75266020 | Dilzem xl mr 240mg Modified-release capsule (Elan Pharma) | Diltiazem hydrochloride | | | 1 |  |
| 26463 | | | | 81081020 | Zemret xl 240mg Capsule (Neo Laboratories Ltd) | Diltiazem hydrochloride | | | 1 |  |
| 68429 | | | | 26726021 | Diltiazem 120mg modified-release capsules (Ethigen Ltd) | Diltiazem hydrochloride | | | 1 |  |
| 17586 | | | | 80135020 | Slozem 300mg capsules (Merck Serono Ltd) | Diltiazem hydrochloride | | | 1 |  |
| 18834 | | | | 82002020 | Disogram SR 60mg capsules (Ranbaxy (UK) Ltd) | Diltiazem hydrochloride | | | 1 |  |
| 65504 | | | | 52684021 | Adizem-SR 180mg capsules (Lexon (UK) Ltd) | Diltiazem hydrochloride | | | 1 |  |
| 30242 | | | | 90601020 | Diltiazem 180mg modified-release capsules | Diltiazem hydrochloride | | | 1 |  |
| 59585 | | | | 47168020 | Uard 120XL capsules (Ennogen Healthcare Ltd) | Diltiazem hydrochloride | | | 1 |  |
| 46937 | | | | 56930020 | Diltiazem 60mg modified-release tablets (Actavis UK Ltd) | Diltiazem hydrochloride | | | 1 |  |
| 4923 | | | | 75521020 | Diltiazem 24hr 180mg modified-release capsules | Diltiazem Hydrochloride | | | 1 |  |
| 9723 | | | | 79935020 | Calcicard CR 90mg tablets (Teva UK Ltd) | Diltiazem hydrochloride | | | 1 |  |
| 41489 | | | | 95547020 | Bi-Carzem SR 120mg capsules (Tillomed Laboratories Ltd) | Diltiazem hydrochloride | | | 1 |  |
| 16850 | | | | 83067020 | Angiozem CR 120mg tablets (Ashbourne Pharmaceuticals Ltd) | Diltiazem hydrochloride | | | 1 |  |
| 13127 | | | | 87836020 | Dilzem XL 240 capsules (Teva UK Ltd) | Diltiazem hydrochloride | | | 1 |  |
| 70306 | | | | 25462021 | Diltiazem 180mg modified-release capsules (Mawdsley-Brooks & Company Ltd) | Diltiazem hydrochloride | | | 1 |  |
| 49390 | | | | 1871020 | Diltiazem 90mg modified-release tablets (Alliance Healthcare (Distribution) Ltd) | Diltiazem hydrochloride | | | 1 |  |
| 36664 | | | | 81082020 | Zemret xl 300mg Capsule (Neo Laboratories Ltd) | Diltiazem hydrochloride | | | 1 |  |
| 57594 | | | | 1906020 | Tildiem 60mg modified-release tablets (Waymade Healthcare Plc) | Diltiazem hydrochloride | | | 1 |  |
| 18379 | | | | 86144020 | Dilcardia SR 90mg capsules (Mylan) | Diltiazem hydrochloride | | | 1 |  |
| 31489 | | | | 86211020 | Bi-carzem xl 240mg Capsule (Tillomed Laboratories Ltd) | Diltiazem hydrochloride | | | 1 |  |
| 57208 | | | | 47167020 | Diltiazem 120mg modified-release capsules (Cubic Pharmaceuticals Ltd) | Diltiazem hydrochloride | | | 1 |  |
| 17492 | | | | 84732020 | Zemtard 300 XL capsules (Galen Ltd) | Diltiazem hydrochloride | | | 1 |  |
| 21773 | | | | 60454020 | Diltiazem HCl 60mg Tablet (Generics (UK) Ltd) | Diltiazem hydrochloride | | | 1 |  |
| 38855 | | | | 95561020 | Adizem-XL 180mg capsules (Napp Pharmaceuticals Ltd) | Diltiazem hydrochloride | | | 1 |  |
| 38634 | | | | 95575020 | Adizem-XL 300mg capsules (Napp Pharmaceuticals Ltd) | Diltiazem hydrochloride | | | 1 |  |
| 48272 | | | | 1893020 | Diltiazem 60mg modified-release capsules (Alliance Healthcare (Distribution) Ltd) | Diltiazem hydrochloride | | | 1 |  |
| 939 | | | | 60296020 | Tildiem Retard 90mg tablets (Sanofi) | Diltiazem hydrochloride | | | 1 |  |
| 32089 | | | | 49567020 | Diltiazem HCl 120mg Modified-release capsule (Hillcross Pharmaceuticals Ltd) | Diltiazem Hydrochloride | | | 1 |  |
| 18874 | | | | 68687020 | Disogram SR 180mg capsules (Ranbaxy (UK) Ltd) | Diltiazem hydrochloride | | | 1 |  |
| 69116 | | | | 53313021 | Diltiazem 60mg modified-release tablets (DE Pharmaceuticals) | Diltiazem hydrochloride | | | 1 |  |
| 48870 | | | | 39292020 | Adizem-SR 90mg capsules (DE Pharmaceuticals) | Diltiazem hydrochloride | | | 1 |  |
| 47608 | | | | 97279020 | Zemret 300 XL capsules (Tillomed Laboratories Ltd) | Diltiazem hydrochloride | | | 1 |  |
| 32870 | | | | 60682020 | Diltiazem 60mg modified-release tablets (Sterwin Medicines) | Diltiazem hydrochloride | | | 1 |  |
| 61010 | | | | 20298021 | Diltiazem 120mg modified-release tablets (Cubic Pharmaceuticals Ltd) | Diltiazem hydrochloride | | | 1 |  |
| 219 | | | | 72563020 | Diltiazem 120mg modified-release tablets | Diltiazem hydrochloride | | | 1 |  |
| 61245 | | | | 26728021 | Diltiazem 60mg modified-release capsules (Sigma Pharmaceuticals Plc) | Diltiazem hydrochloride | | | 1 |  |
| 38632 | | | | 95541020 | Adizem-SR 90mg capsules (Napp Pharmaceuticals Ltd) | Diltiazem hydrochloride | | | 1 |  |
| 43430 | | | | 61274020 | Diltiazem 120mg modified-release tablets (A A H Pharmaceuticals Ltd) | Diltiazem hydrochloride | | | 1 |  |
| 11223 | | | | 79519020 | Angitil SR 90 capsules (Ethypharm UK Ltd) | Diltiazem hydrochloride | | | 1 |  |
| 67317 | | | | 1885020 | Dilzem XL 120 capsules (Mawdsley-Brooks & Company Ltd) | Diltiazem hydrochloride | | | 1 |  |
| 68054 | | | | 1904020 | Diltiazem 60mg modified-release tablets (Alliance Healthcare (Distribution) Ltd) | Diltiazem hydrochloride | | | 1 |  |
| 62064 | | | | 25559021 | Diltiazem 120mg modified-release tablets (Mawdsley-Brooks & Company Ltd) | Diltiazem hydrochloride | | | 1 |  |
| 36583 | | | | 81080020 | Zemret xl 180mg Capsule (Neo Laboratories Ltd) | Diltiazem Hydrochloride | | | 1 |  |
| 38545 | | | | 95521020 | Tildiem LA 200 capsules (Sanofi) | Diltiazem hydrochloride | | | 1 |  |
| 517 | | | | 57066020 | Adizem sr 120mg Modified-release capsule (Napp Pharmaceuticals Ltd) | Diltiazem Hydrochloride | | | 1 |  |
| 15288 | | | | 86669020 | Angitil XL 300 capsules (Ethypharm UK Ltd) | Diltiazem hydrochloride | | | 1 |  |
| 5348 | | | | 72176020 | Diltiazem 300mg modified-release capsules | Diltiazem hydrochloride | | | 1 |  |
| 2888 | | | | 62586020 | Tildiem 60mg modified-release tablets (Sanofi) | Diltiazem hydrochloride | | | 1 |  |
| 12639 | | | | 56931020 | Diltiazem HCl 90mg Modified-release tablet (Actavis UK Ltd) | Diltiazem hydrochloride | | | 1 |  |
| 39298 | | | | 95545020 | Bi-Carzem SR 90mg capsules (Tillomed Laboratories Ltd) | Diltiazem hydrochloride | | | 1 |  |
| 1538 | | | | 68602020 | Diltiazem 60mg tablets | Diltiazem Hydrochloride | | | 1 |  |
| 12705 | | | | 83066020 | Angiozem CR 90mg tablets (Ashbourne Pharmaceuticals Ltd) | Diltiazem hydrochloride | | | 1 |  |
| 42804 | | | | 65086020 | Diltiazem HCl 180mg Capsule (PLIVA Pharma Ltd) | Diltiazem Hydrochloride | | | 1 |  |
| 42819 | | | | 69923020 | Diltiazem xl 240mg Capsule (Hillcross Pharmaceuticals Ltd) | Diltiazem hydrochloride | | | 1 |  |
| 38066 | | | | 59378020 | Diltiazem HCl 60mg Modified-release tablet (Lagap) | Diltiazem hydrochloride | | | 1 |  |
| 51261 | | | | 11007020 | Tildiem Retard 120mg tablets (Mawdsley-Brooks & Company Ltd) | Diltiazem hydrochloride | | | 1 |  |
| 3118 | | | | 72560020 | Adizem sr 90mg Modified-release capsule (Napp Pharmaceuticals Ltd) | Diltiazem hydrochloride | | | 1 |  |
| 31676 | | | | 56932020 | Diltiazem HCl 120mg Modified-release tablet (Actavis UK Ltd) | Diltiazem hydrochloride | | | 1 |  |
| 55257 | | | | 19777020 | Diltiazem 60mg/5ml oral solution | Diltiazem hydrochloride | | | 1 |  |
| 1686 | | | | 57176020 | Diltiazem 90mg modified-release capsules | Diltiazem hydrochloride | | | 1 |  |
| 38876 | | | | 95519020 | Tildiem LA 300 capsules (Sanofi) | Diltiazem hydrochloride | | | 1 |  |
| 1289 | | | | 60297020 | Tildiem Retard 120mg tablets (Sanofi) | Diltiazem hydrochloride | | | 1 |  |
| 32658 | | | | 75962020 | Dilcardia xl 120mg Modified-release capsule (Generics (UK) Ltd) | Diltiazem Hydrochloride | | | 1 |  |
| 59098 | | | | 1889020 | Dilzem XL 180 capsules (Lexon (UK) Ltd) | Diltiazem hydrochloride | | | 1 |  |
| 62912 | | | | 44230021 | Diltiazem 120mg modified-release capsules (AM Distributions (Yorkshire) Ltd) | Diltiazem hydrochloride | | | 1 |  |
| 13027 | | | | 84423020 | Viazem XL 240mg capsules (Thornton & Ross Ltd) | Diltiazem hydrochloride | | | 1 |  |
| 13240 | | | | 87832020 | Dilzem XL 120 capsules (Teva UK Ltd) | Diltiazem hydrochloride | | | 1 |  |
| 18404 | | | | 49568020 | Diltiazem 60mg modified-release capsules (A A H Pharmaceuticals Ltd) | Diltiazem hydrochloride | | | 1 |  |
| 31737 | | | | 80910020 | Zildil SR 120mg capsules (Chanelle Medical UK Ltd) | Diltiazem hydrochloride | | | 1 |  |
| 5054 | | | | 79521020 | Angitil SR 180 capsules (Ethypharm UK Ltd) | Diltiazem hydrochloride | | | 1 |  |
| 2453 | | | | 68603020 | Diltiazem 60mg modified-release capsules | Diltiazem Hydrochloride | | | 1 |  |
| 38818 | | | | 95537020 | Adizem-SR 120mg capsules (Napp Pharmaceuticals Ltd) | Diltiazem hydrochloride | | | 1 |  |
| 56758 | | | | 47166020 | Diltiazem 90mg modified-release capsules (Cubic Pharmaceuticals Ltd) | Diltiazem hydrochloride | | | 1 |  |
| 47530 | | | | 76823020 | Horizem SR 60mg capsules (Horizon lifecare) | Diltiazem hydrochloride | | | 1 |  |
| 19440 | | | | 77223020 | Disogram SR 300mg capsules (Ranbaxy (UK) Ltd) | Diltiazem hydrochloride | | | 1 |  |
| 5234 | | | | 76294020 | Slozem 180mg capsules (Merck Serono Ltd) | Diltiazem hydrochloride | | | 1 |  |
| 66701 | | | | 23046021 | Diltiazem 240mg modified-release capsules (DE Pharmaceuticals) | Diltiazem hydrochloride | | | 1 |  |
| 4852 | | | | 72558020 | Adizem sr 120mg Modified-release tablet (Napp Pharmaceuticals Ltd) | Diltiazem hydrochloride | | | 1 |  |
| 9240 | | | | 75163020 | Adizem xl 180mg Capsule (Napp Pharmaceuticals Ltd) | Diltiazem Hydrochloride | | | 1 |  |
| 21145 | | | | 86146020 | Dilcardia SR 60mg capsules (Mylan) | Diltiazem hydrochloride | | | 1 |  |
| 2528 | | | | 76293020 | Slozem 120mg capsules (Merck Serono Ltd) | Diltiazem hydrochloride | | | 1 |  |
| 23505 | | | | 81470020 | Adizem xl plus 150mg+12.5mg Modified-release capsule (Napp Pharmaceuticals Ltd) | Diltiazem Hydrochloride/Hydrochlorothiazide | | | 0 | 1 |
| 18606 | | | | 81472020 | Diltiazem and hydrochlorothiazide 150mg+12.5mg modified-release capsules | Diltiazem Hydrochloride/Hydrochlorothiazide | | | 0 | 1 |
| 26337 | | | | 76787020 | Cabren 10mg modified-release tablets (Teva UK Ltd) | Felodipine | | | 1 |  |
| 29044 | | | | 88260020 | Neofel XL 10mg tablets (Kent Pharmaceuticals Ltd) | Felodipine | | | 1 |  |
| 17566 | | | | 87067020 | Felotens XL 10mg tablets (Thornton & Ross Ltd) | Felodipine | | | 1 |  |
| 68499 | | | | 66909020 | Felodipine sr 10mg Tablet (Approved Prescription Services Ltd) | Felodipine | | | 1 |  |
| 33091 | | | | 66476020 | Felodipine 10mg modified-release tablets (A A H Pharmaceuticals Ltd) | Felodipine | | | 1 |  |
| 20459 | | | | 87085020 | Felendil xl 10mg Modified-release tablet (Ratiopharm UK Ltd) | Felodipine | | | 1 |  |
| 66095 | | | | 53318021 | Felodipine 5mg modified-release tablets (Mawdsley-Brooks & Company Ltd) | Felodipine | | | 1 |  |
| 7280 | | | | 65383020 | Plendil 10mg modified-release tablets (AstraZeneca UK Ltd) | Felodipine | | | 1 |  |
| 29145 | | | | 91113020 | Felendil xl 2.5mg Modified-release tablet (Ratiopharm UK Ltd) | Felodipine | | | 1 |  |
| 9334 | | | | 65384020 | Plendil 2.5mg modified-release tablets (AstraZeneca UK Ltd) | Felodipine | | | 1 |  |
| 58339 | | | | 2044020 | Neofel XL 2.5mg tablets (Almus Pharmaceuticals Ltd) | Felodipine | | | 1 |  |
| 48009 | | | | 68173020 | Felodipine 5mg Modified-release tablet (Sandoz Ltd) | Felodipine | | | 1 |  |
| 64504 | | | | 2030020 | Plendil 5mg modified-release tablets (Necessity Supplies Ltd) | Felodipine | | | 1 |  |
| 36620 | | | | 88802020 | Parmid XL 10mg tablets (Sandoz Ltd) | Felodipine | | | 1 |  |
| 33932 | | | | 88800020 | Parmid XL 5mg tablets (Sandoz Ltd) | Felodipine | | | 1 |  |
| 55306 | | | | 99312020 | Folpik XL 5mg tablets (Teva UK Ltd) | Felodipine | | | 1 |  |
| 60884 | | | | 21738021 | Felodipine 2.5mg modified-release tablets (Phoenix Healthcare Distribution Ltd) | Felodipine | | | 1 |  |
| 38434 | | | | 93025020 | Keloc SR 10mg tablets (Teva UK Ltd) | Felodipine | | | 1 |  |
| 68181 | | | | 52188021 | Felodipine 5mg modified-release tablets (Sigma Pharmaceuticals Plc) | Felodipine | | | 1 |  |
| 491 | | | | 52181020 | Felodipine 2.5mg modified-release tablets | Felodipine | | | 1 |  |
| 9437 | | | | 65382020 | Plendil 5mg modified-release tablets (AstraZeneca UK Ltd) | Felodipine | | | 1 |  |
| 25572 | | | | 86937020 | Felogen XL 5mg tablets (Mylan) | Felodipine | | | 1 |  |
| 43790 | | | | 92017020 | Vascalpha 10mg modified-release tablets (Almus Pharmaceuticals Ltd) | Felodipine | | | 1 |  |
| 14305 | | | | 87544020 | Vascalpha 10mg modified-release tablets (Actavis UK Ltd) | Felodipine | | | 1 |  |
| 68828 | | | | 21735021 | Felodipine 5mg modified-release tablets (DE Pharmaceuticals) | Felodipine | | | 1 |  |
| 39357 | | | | 93501020 | Neofel XL 2.5mg tablets (Kent Pharmaceuticals Ltd) | Felodipine | | | 1 |  |
| 35592 | | | | 92751020 | Cardioplen XL 2.5mg tablets (Chiesi Ltd) | Felodipine | | | 1 |  |
| 64474 | | | | 73487020 | Felodipine 2.5mg modified-release tablets (A A H Pharmaceuticals Ltd) | Felodipine | | | 1 |  |
| 63331 | | | | 99300020 | Folpik XL 2.5mg tablets (Teva UK Ltd) | Felodipine | | | 1 |  |
| 65349 | | | | 99314020 | Folpik XL 10mg tablets (Teva UK Ltd) | Felodipine | | | 1 |  |
| 501 | | | | 52179020 | Felodipine 5mg modified-release tablets | Felodipine | | | 1 |  |
| 44859 | | | | 66904020 | Felodipine sr 5mg Tablet (Approved Prescription Services Ltd) | Felodipine | | | 1 |  |
| 69206 | | | | 37681020 | Felodipine 2.5mg/5ml oral solution | Felodipine | | | 1 |  |
| 55740 | | | | 2041020 | Neofel XL 2.5mg tablets (Actavis UK Ltd) | Felodipine | | | 1 |  |
| 43394 | | | | 89315020 | Pinefeld XL 10mg tablets (Tillomed Laboratories Ltd) | Felodipine | | | 1 |  |
| 64719 | | | | 52190021 | Felodipine 2.5mg modified-release tablets (Sigma Pharmaceuticals Plc) | Felodipine | | | 1 |  |
| 40633 | | | | 92015020 | Vascalpha 5mg modified-release tablets (Almus Pharmaceuticals Ltd) | Felodipine | | | 1 |  |
| 64760 | | | | 21736021 | Felodipine 10mg modified-release tablets (Phoenix Healthcare Distribution Ltd) | Felodipine | | | 1 |  |
| 37897 | | | | 94403020 | Felotens XL 2.5mg tablets (Thornton & Ross Ltd) | Felodipine | | | 1 |  |
| 568 | | | | 52180020 | Felodipine 10mg modified-release tablets | Felodipine | | | 1 |  |
| 30557 | | | | 87103020 | Felogen XL 10mg tablets (Mylan) | Felodipine | | | 1 |  |
| 32922 | | | | 68176020 | Felodipine 10mg Modified-release tablet (Sandoz Ltd) | Felodipine | | | 1 |  |
| 60652 | | | | 23052021 | Parmid XL 2.5mg tablets (Sandoz Ltd) | Felodipine | | | 1 |  |
| 24365 | | | | 87956020 | Cardioplen XL 5mg tablets (Chiesi Ltd) | Felodipine | | | 1 |  |
| 30991 | | | | 76784020 | Cabren 5mg modified-release tablets (Teva UK Ltd) | Felodipine | | | 1 |  |
| 30915 | | | | 78440020 | Cabren 2.5mg modified-release tablets (Teva UK Ltd) | Felodipine | | | 1 |  |
| 10153 | | | | 87083020 | Felendil xl 5mg Modified-release tablet (Ratiopharm UK Ltd) | Felodipine | | | 1 |  |
| 17557 | | | | 87065020 | Felotens XL 5mg tablets (Thornton & Ross Ltd) | Felodipine | | | 1 |  |
| 64917 | | | | 20171021 | Felodipine 10mg modified-release tablets (Waymade Healthcare Plc) | Felodipine | | | 1 |  |
| 24366 | | | | 87958020 | Cardioplen XL 10mg tablets (Chiesi Ltd) | Felodipine | | | 1 |  |
| 66910 | | | | 57274021 | Felodipine 2.5mg modified-release tablets (DE Pharmaceuticals) | Felodipine | | | 1 |  |
| 43512 | | | | 66473020 | Felodipine 5mg modified-release tablets (A A H Pharmaceuticals Ltd) | Felodipine | | | 1 |  |
| 60569 | | | | 20172021 | Felodipine 2.5mg modified-release tablets (Waymade Healthcare Plc) | Felodipine | | | 1 |  |
| 28721 | | | | 88258020 | Neofel XL 5mg tablets (Kent Pharmaceuticals Ltd) | Felodipine | | | 1 |  |
| 35084 | | | | 87542020 | Vascalpha 5mg modified-release tablets (Actavis UK Ltd) | Felodipine | | | 1 |  |
| 11567 | | | | 81446020 | Ramipril 5mg with felodipine 5mg modified-release tablet | Felodipine/Ramipril | | | 0 | 1 |
| 28438 | | | | 85815020 | Triapin 2.5mg/2.5mg modified-release tablets (Sanofi) | Felodipine/Ramipril | | | 0 | 1 |
| 11965 | | | | 81445020 | Ramipril 2.5mg with felodipine 2.5mg modified-release tablet | Felodipine/Ramipril | | | 0 | 1 |
| 17474 | | | | 78452020 | Felodipine 5mg modified-release / Ramipril 5mg tablets | Felodipine/Ramipril | | | 0 | 1 |
| 17006 | | | | 85824020 | Triapin 5mg/5mg modified-release tablets (Sanofi) | Felodipine/Ramipril | | | 0 | 1 |
| 21162 | | | | 78451020 | Felodipine 2.5mg modified-release / Ramipril 2.5mg tablets | Felodipine/Ramipril | | | 0 | 1 |
| 47727 | | | | 99789020 | Sevikar HCT 40mg/5mg/25mg tablets (Daiichi Sankyo UK Ltd) | Hydrochlorothiazide/Amlodipine besilate/Olmesartan medoxomil | | | 0 | 1 |
| 53220 | | | | 99791020 | Sevikar HCT 40mg/10mg/25mg tablets (Daiichi Sankyo UK Ltd) | Hydrochlorothiazide/Amlodipine besilate/Olmesartan medoxomil | | | 0 | 1 |
| 60780 | | | | 38011020 | Generic Sevikar HCT 20mg/5mg/12.5mg tablets | Hydrochlorothiazide/Olmesartan medoxomil/Amlodipine besilate | | | 0 | 1 |
| 46355 | | | | 99779020 | Sevikar HCT 20mg/5mg/12.5mg tablets (Daiichi Sankyo UK Ltd) | Hydrochlorothiazide/Olmesartan medoxomil/Amlodipine besilate | | | 0 | 1 |
| 8310 | | | | 69563020 | Isradipine 2.5mg tablets | Isradipine | | | 1 |  |
| 8257 | | | | 69560020 | Prescal 2.5mg tablets (Novartis Pharmaceuticals UK Ltd) | Isradipine | | | 1 |  |
| 11966 | | | | 74740020 | Motens 2mg tablets (GlaxoSmithKline UK Ltd) | Lacidipine | | | 1 |  |
| 5158 | | | | 74744020 | Lacidipine 2mg tablets | Lacidipine | | | 1 |  |
| 3221 | | | | 74745020 | Lacidipine 4mg tablets | Lacidipine | | | 1 |  |
| 56994 | | | | 77966020 | Lacidipine 4mg tablets (Teva UK Ltd) | Lacidipine | | | 1 |  |
| 57680 | | | | 78002020 | Lacidipine 4mg tablets (A A H Pharmaceuticals Ltd) | Lacidipine | | | 1 |  |
| 9670 | | | | 74741020 | Motens 4mg tablets (GlaxoSmithKline UK Ltd) | Lacidipine | | | 1 |  |
| 60699 | | | | 2051020 | Lacidipine 2mg tablets (Sigma Pharmaceuticals Plc) | Lacidipine | | | 1 |  |
| 70990 | | | | 78005020 | Lacidipine 2mg tablets (A A H Pharmaceuticals Ltd) | Lacidipine | | | 1 |  |
| 70732 | | | | 28124020 | Lercanidipine 20mg tablets (Alliance Healthcare (Distribution) Ltd) | Lercanidipine hydrochloride | | | 1 |  |
| 61611 | | | | 23689021 | Lercanidipine 10mg tablets (DE Pharmaceuticals) | Lercanidipine hydrochloride | | | 1 |  |
| 70827 | | | | 77159020 | Lercanidipine 10mg tablets (Teva UK Ltd) | Lercanidipine hydrochloride | | | 1 |  |
| 5570 | | | | 85619020 | Zanidip 10mg tablets (Recordati Pharmaceuticals Ltd) | Lercanidipine hydrochloride | | | 1 |  |
| 56767 | | | | 77347020 | Lercanidipine 20mg tablets (Mylan) | Lercanidipine hydrochloride | | | 1 |  |
| 71018 | | | | 42160020 | Lercanidipine 10mg tablets (Arrow Generics Ltd) | Lercanidipine hydrochloride | | | 1 |  |
| 14300 | | | | 90707020 | Zanidip 20mg tablets (Recordati Pharmaceuticals Ltd) | Lercanidipine hydrochloride | | | 1 |  |
| 5593 | | | | 79553020 | Lercanidipine 10mg tablets | Lercanidipine hydrochloride | | | 1 |  |
| 65659 | | | | 77164020 | Lercanidipine 20mg tablets (Teva UK Ltd) | Lercanidipine hydrochloride | | | 1 |  |
| 69239 | | | | 2096020 | Lercanidipine 10mg tablets (A A H Pharmaceuticals Ltd) | Lercanidipine hydrochloride | | | 1 |  |
| 71030 | | | | 43674020 | Lercanidipine 20mg tablets (Arrow Generics Ltd) | Lercanidipine hydrochloride | | | 1 |  |
| 57444 | | | | 14783021 | Lercanidipine 10mg tablets (Aptil Pharma Ltd) | Lercanidipine hydrochloride | | | 1 |  |
| 64424 | | | | 28126020 | Lercanidipine 20mg tablets (Zentiva) | Lercanidipine hydrochloride | | | 1 |  |
| 64227 | | | | 77284020 | Lercanidipine 10mg tablets (Actavis UK Ltd) | Lercanidipine hydrochloride | | | 1 |  |
| 59233 | | | | 77287020 | Lercanidipine 20mg tablets (Actavis UK Ltd) | Lercanidipine hydrochloride | | | 1 |  |
| 63917 | | | | 28127020 | Lercanidipine 20mg tablets (A A H Pharmaceuticals Ltd) | Lercanidipine hydrochloride | | | 1 |  |
| 47331 | | | | 77345020 | Lercanidipine 10mg tablets (Mylan) | Lercanidipine hydrochloride | | | 1 |  |
| 13243 | | | | 90705020 | Lercanidipine 20mg tablets | Lercanidipine hydrochloride | | | 1 |  |
| 3931 | | | | 84204020 | Posicor 100mg Tablet (Roche Products Ltd) | Mibefradil | | | 1 |  |
| 22241 | | | | 84495020 | Mibefradil 100mg Tablet | Mibefradil | | | 1 |  |
| 1529 | | | | 84203020 | Posicor 50mg Tablet (Roche Products Ltd) | Mibefradil | | | 1 |  |
| 15652 | | | | 84494020 | Mibefradil 50mg Tablet | Mibefradil | | | 1 |  |
| 11943 | | | | 58763020 | Cardene 20mg capsules (Astellas Pharma Ltd) | Nicardipine hydrochloride | | | 1 |  |
| 7562 | | | | 58764020 | Cardene 30mg capsules (Astellas Pharma Ltd) | Nicardipine hydrochloride | | | 1 |  |
| 8201 | | | | 65032020 | Nicardipine 30mg capsules | Nicardipine hydrochloride | | | 1 |  |
| 2926 | | | | 65031020 | Nicardipine 20mg capsules | Nicardipine hydrochloride | | | 1 |  |
| 3302 | | | | 54862020 | Cardene SR 30mg capsules (Astellas Pharma Ltd) | Nicardipine hydrochloride | | | 1 |  |
| 9386 | | | | 52064020 | Nicardipine 45mg modified-release capsules | Nicardipine hydrochloride | | | 1 |  |
| 5477 | | | | 52063020 | Nicardipine 30mg modified-release capsules | Nicardipine hydrochloride | | | 1 |  |
| 45292 | | | | 64571020 | Nicardipine 30mg capsules (A A H Pharmaceuticals Ltd) | Nicardipine hydrochloride | | | 1 |  |
| 12875 | | | | 54863020 | Cardene SR 45mg capsules (Astellas Pharma Ltd) | Nicardipine hydrochloride | | | 1 |  |
| 24228 | | | | 83201020 | Nimodrel 20mg modified-release tablet (Opus Pharmaceuticals Ltd) | Nifedipine | | | 1 |  |
| 52017 | | | | 1952020 | Adalat LA 30 tablets (Mawdsley-Brooks & Company Ltd) | Nifedipine | | | 1 |  |
| 5181 | | | | 71013020 | Angiopine MR 20mg tablets (Ashbourne Pharmaceuticals Ltd) | Nifedipine | | | 1 |  |
| 19170 | | | | 82932020 | Tensipine MR 10 tablets (Thornton & Ross Ltd) | Nifedipine | | | 1 |  |
| 67074 | | | | 1937020 | Adalat 10mg capsules (DE Pharmaceuticals) | Nifedipine | | | 1 |  |
| 47027 | | | | 63403020 | Nifedipine 10mg Modified-release tablet (Kent Pharmaceuticals Ltd) | Nifedipine | | | 1 |  |
| 45685 | | | | 99249020 | Adanif XL 30mg tablets (Focus Pharmaceuticals Ltd) | Nifedipine | | | 1 |  |
| 21872 | | | | 70717020 | Angiopine 5mg Capsule (Ashbourne Pharmaceuticals Ltd) | Nifedipine | | | 1 |  |
| 26774 | | | | 91013020 | Nifedipine 10mg/5ml Oral suspension | Nifedipine | | | 1 |  |
| 13699 | | | | 83276020 | Angiopine la 40mg Tablet (Ashbourne Pharmaceuticals Ltd) | Nifedipine | | | 1 |  |
| 53629 | | | | 1972020 | Adalat retard 20mg tablets (Lexon (UK) Ltd) | Nifedipine | | | 1 |  |
| 33025 | | | | 90565020 | Nimodrel XL 30mg tablets (Zurich Pharmaceuticals) | Nifedipine | | | 1 |  |
| 22217 | | | | 83200020 | Nimodrel 10mg modified-release tablet (Opus Pharmaceuticals Ltd) | Nifedipine | | | 1 |  |
| 20257 | | | | 75490020 | Cardilate MR 20mg tablets (IVAX Pharmaceuticals UK Ltd) | Nifedipine | | | 1 |  |
| 1262 | | | | 65057020 | Nifedipine 12 20mg Modified-release tablet | Nifedipine | | | 1 |  |
| 34522 | | | | 49116020 | Nifedipine 5mg capsules (A A H Pharmaceuticals Ltd) | Nifedipine | | | 1 |  |
| 49762 | | | | 1947020 | Nifedipine 10mg modified-release tablets (Alliance Healthcare (Distribution) Ltd) | Nifedipine | | | 1 |  |
| 60856 | | | | 23048021 | Nifedipine 10mg modified-release tablets (Sigma Pharmaceuticals Plc) | Nifedipine | | | 1 |  |
| 66191 | | | | 1969020 | Adalat retard 20mg tablets (DE Pharmaceuticals) | Nifedipine | | | 1 |  |
| 11512 | | | | 84876020 | Nifedipress MR 10 tablets (Dexcel-Pharma Ltd) | Nifedipine | | | 1 |  |
| 34146 | | | | 61467020 | Nifedipine mr 10mg Modified-release tablet (IVAX Pharmaceuticals UK Ltd) | Nifedipine | | | 1 |  |
| 662 | | | | 52564020 | Adalat 5mg capsules (Bayer Plc) | Nifedipine | | | 1 |  |
| 53500 | | | | 1950020 | Adalat LA 30 tablets (DE Pharmaceuticals) | Nifedipine | | | 1 |  |
| 20311 | | | | 57957020 | Nifedipress mr 20mg Modified-release tablet (Generics (UK) Ltd) | Nifedipine | | | 1 |  |
| 737 | | | | 73827020 | Nifedipine 20mg modified-release capsules | Nifedipine | | | 1 |  |
| 452 | | | | 65052020 | Nifedipine 10mg capsules | Nifedipine | | | 1 |  |
| 47285 | | | | 73272020 | Nifedipine xl 60mg Tablet (Hillcross Pharmaceuticals Ltd) | Nifedipine | | | 1 |  |
| 69202 | | | | 33046020 | Nifedipine 2mg/5ml oral suspension | Nifedipine | | | 1 |  |
| 25132 | | | | 90751020 | Nifopress MR 20mg tablets (Teva UK Ltd) | Nifedipine | | | 1 |  |
| 34975 | | | | 54545020 | Nifedipine 5mg capsules (Teva UK Ltd) | Nifedipine | | | 1 |  |
| 4856 | | | | 73758020 | Coracten SR 20mg capsules (UCB Pharma Ltd) | Nifedipine | | | 1 |  |
| 2605 | | | | 73828020 | Nifedipine 10mg modified-release capsules | Nifedipine | | | 1 |  |
| 53357 | | | | 20553020 | Nifedipine 10mg/5ml oral suspension | Nifedipine | | | 1 |  |
| 59163 | | | | 47174020 | Nifedipine 20mg modified-release tablets (Cubic Pharmaceuticals Ltd) | Nifedipine | | | 1 |  |
| 53278 | | | | 1953020 | Adalat LA 30 tablets (Necessity Supplies Ltd) | Nifedipine | | | 1 |  |
| 9485 | | | | 50475020 | Hypolar Retard 20 tablets (Sandoz Ltd) | Nifedipine | | | 1 |  |
| 1300 | | | | 54944020 | Nifensar xl 20mg Modified-release tablet (Rhone-Poulenc Rorer Ltd) | Nifedipine | | | 1 |  |
| 11769 | | | | 77698020 | Calchan MR 20 tablets (Ranbaxy (UK) Ltd) | Nifedipine | | | 1 |  |
| 17448 | | | | 84211020 | Nifedipress mr 10mg Modified-release tablet (Sterwin Medicines) | Nifedipine | | | 1 |  |
| 8213 | | | | 75515020 | Nifedipine 24 20mg Modified-release tablet | Nifedipine | | | 1 |  |
| 21886 | | | | 85847020 | Nifedipress MR 20 tablets (Actavis UK Ltd) | Nifedipine | | | 1 |  |
| 58557 | | | | 1975020 | Adalat LA 20 tablets (Necessity Supplies Ltd) | Nifedipine | | | 1 |  |
| 41979 | | | | 85923020 | Adipine la 30mg Modified-release tablet (Chiesi Ltd) | Nifedipine | | | 1 |  |
| 42912 | | | | 54546020 | Nifedipine 10mg capsules (Teva UK Ltd) | Nifedipine | | | 1 |  |
| 14861 | | | | 77697020 | Calchan MR 10 tablets (Ranbaxy (UK) Ltd) | Nifedipine | | | 1 |  |
| 43818 | | | | 98791020 | Adalat LA 60mg tablets (Bayer Plc) | Nifedipine | | | 1 |  |
| 34607 | | | | 54160020 | Nifedipine 5mg capsules (IVAX Pharmaceuticals UK Ltd) | Nifedipine | | | 1 |  |
| 47529 | | | | 98903020 | Nifedipine 20mg/ml oral drops | Nifedipine | | | 1 |  |
| 43753 | | | | 98789020 | Adalat LA 30mg tablets (Bayer Plc) | Nifedipine | | | 1 |  |
| 35646 | | | | 91231020 | Neozipine XL 60mg tablets (Kent Pharmaceuticals Ltd) | Nifedipine | | | 1 |  |
| 9573 | | | | 54677020 | Slofedipine XL 30mg tablets (Zentiva) | Nifedipine | | | 1 |  |
| 22142 | | | | 70808020 | Calcilat 10mg Capsule (Eastern Pharmaceuticals Ltd) | Nifedipine | | | 1 |  |
| 43511 | | | | 49117020 | Nifedipine 10mg capsules (A A H Pharmaceuticals Ltd) | Nifedipine | | | 1 |  |
| 34101 | | | | 61468020 | Nifedipine mr 20mg Modified-release tablet (IVAX Pharmaceuticals UK Ltd) | Nifedipine | | | 1 |  |
| 69668 | | | | 33048020 | Nifedipine 30mg/5ml oral suspension | Nifedipine | | | 1 |  |
| 269 | | | | 65053020 | Nifedipine 5mg capsules | Nifedipine | | | 1 |  |
| 10136 | | | | 84877020 | Nifedipress MR 20 tablets (Dexcel-Pharma Ltd) | Nifedipine | | | 1 |  |
| 40074 | | | | 65054020 | Nifedipine 20mg Capsule | Nifedipine | | | 1 |  |
| 13139 | | | | 88895020 | Adipine XL 30mg tablets (Chiesi Ltd) | Nifedipine | | | 1 |  |
| 55455 | | | | 1943020 | Nifedipine 10mg capsules (Strides Shasun (UK) Ltd) | Nifedipine | | | 1 |  |
| 43410 | | | | 75517020 | Nifedipine extra 60mg Modified-release tablet | Nifedipine | | | 1 |  |
| 1854 | | | | 64630020 | Adalat la 30mg Tablet (Bayer Plc) | Nifedipine | | | 1 |  |
| 5162 | | | | 79993020 | Nifedipine 30mg modified-release capsules | Nifedipine | | | 1 |  |
| 21216 | | | | 87081020 | Hypolar Retard 10mg tablets (Sandoz Ltd) | Nifedipine | | | 1 |  |
| 12613 | | | | 81213020 | Unipine xl 30mg Modified-release tablet (Genus Pharmaceuticals Ltd) | Nifedipine | | | 1 |  |
| 34187 | | | | 59923020 | Nifedipine 10mg Modified-release tablet (Generics (UK) Ltd) | Nifedipine | | | 1 |  |
| 28688 | | | | 60425020 | Nifedipine 10mg modified-release tablets (A A H Pharmaceuticals Ltd) | Nifedipine | | | 1 |  |
| 541 | | | | 84301020 | Adalat LA 20mg tablets (Bayer Plc) | Nifedipine | | | 1 |  |
| 17325 | | | | 75491020 | Cardilate MR 10mg tablets (Teva UK Ltd) | Nifedipine | | | 1 |  |
| 3712 | | | | 79074020 | Coracten XL 30mg capsules (UCB Pharma Ltd) | Nifedipine | | | 1 |  |
| 16073 | | | | 86867020 | Nifedipress MR 10 tablets (Teva UK Ltd) | Nifedipine | | | 1 |  |
| 51917 | | | | 1957020 | Adalat LA 60 tablets (Sigma Pharmaceuticals Plc) | Nifedipine | | | 1 |  |
| 5806 | | | | 82933020 | Tensipine MR 20 tablets (Thornton & Ross Ltd) | Nifedipine | | | 1 |  |
| 20878 | | | | 70715020 | Angiopine 10 capsules (Ashbourne Pharmaceuticals Ltd) | Nifedipine | | | 1 |  |
| 3930 | | | | 80874020 | Nifedipine 60mg modified-release tablets | Nifedipine | | | 1 |  |
| 12606 | | | | 76104020 | Nifelease 20mg Modified-release tablet (Eastern Pharmaceuticals Ltd) | Nifedipine | | | 1 |  |
| 57653 | | | | 1970020 | Adalat LA 20 tablets (Sigma Pharmaceuticals Plc) | Nifedipine | | | 1 |  |
| 34247 | | | | 49104020 | Nifedipine 10mg Capsule (Berk Pharmaceuticals Ltd) | Nifedipine | | | 1 |  |
| 4239 | | | | 80337020 | Adipine MR 10 tablets (Chiesi Ltd) | Nifedipine | | | 1 |  |
| 30199 | | | | 68509020 | Nifedipine 30mg modified-release tablets | Nifedipine | | | 1 |  |
| 25646 | | | | 86340020 | Nivaten retard 20mg Modified-release tablet (Actavis UK Ltd) | Nifedipine | | | 1 |  |
| 37530 | | | | 91229020 | Neozipine XL 30mg tablets (Kent Pharmaceuticals Ltd) | Nifedipine | | | 1 |  |
| 47217 | | | | 85924020 | Adipine la 60mg Modified-release tablet (Chiesi Ltd) | Nifedipine | | | 1 |  |
| 26265 | | | | 79357020 | Calanif 5mg Capsule (Berk Pharmaceuticals Ltd) | Nifedipine | | | 1 |  |
| 47614 | | | | 60424020 | Nifedipine 30mg modified-release tablets (A A H Pharmaceuticals Ltd) | Nifedipine | | | 1 |  |
| 5277 | | | | 83624020 | Fortipine LA 40 tablets (AMCo) | Nifedipine | | | 1 |  |
| 10246 | | | | 88897020 | Adipine XL 60mg tablets (Chiesi Ltd) | Nifedipine | | | 1 |  |
| 410 | | | | 68508020 | Nifedipine 10mg modified-release tablets | Nifedipine | | | 1 |  |
| 38107 | | | | 73256020 | Nifedipine sr 30mg Tablet (Hillcross Pharmaceuticals Ltd) | Nifedipine | | | 1 |  |
| 43515 | | | | 57071020 | Nifedipine 10mg capsules (Actavis UK Ltd) | Nifedipine | | | 1 |  |
| 25919 | | | | 49118020 | Nifedipine 20mg modified-release tablets (A A H Pharmaceuticals Ltd) | Nifedipine | | | 1 |  |
| 34115 | | | | 68510020 | Nifedipine 60mg Modified-release tablet | Nifedipine | | | 1 |  |
| 3711 | | | | 79719020 | Adipine MR 20 tablets (Chiesi Ltd) | Nifedipine | | | 1 |  |
| 13672 | | | | 71014020 | Angiopine MR 10mg tablets (Ashbourne Pharmaceuticals Ltd) | Nifedipine | | | 1 |  |
| 10135 | | | | 86770020 | Nifedipress mr 10mg Modified-release tablet (Sandoz Ltd) | Nifedipine | | | 1 |  |
| 9269 | | | | 80875020 | Nifedipine 40mg modified-release tablets | Nifedipine | | | 1 |  |
| 47887 | | | | 90567020 | Nimodrel XL 60mg tablets (Zurich Pharmaceuticals) | Nifedipine | | | 1 |  |
| 30473 | | | | 86711020 | Coroday MR 20mg tablets (Mylan) | Nifedipine | | | 1 |  |
| 37726 | | | | 92999020 | Nifedipine 100mg/5ml oral suspension | Nifedipine | | | 1 |  |
| 4227 | | | | 64631020 | Adalat la 60mg Tablet (Bayer Plc) | Nifedipine | | | 1 |  |
| 20591 | | | | 86868020 | Nifedipress MR 20 tablets (Teva UK Ltd) | Nifedipine | | | 1 |  |
| 7541 | | | | 57458020 | Nifopress Retard 20mg tablets (AMCo) | Nifedipine | | | 1 |  |
| 23736 | | | | 86128020 | Hypolar XL 30 tablets (Sandoz Ltd) | Nifedipine | | | 1 |  |
| 4939 | | | | 79075020 | Coracten XL 60mg capsules (UCB Pharma Ltd) | Nifedipine | | | 1 |  |
| 22019 | | | | 79356020 | Calanif 10mg Capsule (Berk Pharmaceuticals Ltd) | Nifedipine | | | 1 |  |
| 15715 | | | | 85589020 | Genalat retard 20mg Modified-release tablet (Wyeth Pharmaceuticals) | Nifedipine | | | 1 |  |
| 53990 | | | | 20557020 | Nifedipine 5mg/5ml oral suspension | Nifedipine | | | 1 |  |
| 58990 | | | | 47173020 | Nifedipine 10mg modified-release tablets (Cubic Pharmaceuticals Ltd) | Nifedipine | | | 1 |  |
| 1449 | | | | 80873020 | Nifedipine 24 30mg Modified-release tablet | Nifedipine | | | 1 |  |
| 39800 | | | | 94186020 | Valni XL 60mg tablets (Zentiva) | Nifedipine | | | 1 |  |
| 56469 | | | | 1958020 | Adalat LA 60 tablets (Necessity Supplies Ltd) | Nifedipine | | | 1 |  |
| 2521 | | | | 52565020 | Adalat 10mg capsules (Bayer Plc) | Nifedipine | | | 1 |  |
| 37025 | | | | 90709020 | Nifedipine 20mg modified-release tablets | Nifedipine | | | 1 |  |
| 21245 | | | | 85846020 | Nifedipress mr 10mg Modified-release tablet (Actavis UK Ltd) | Nifedipine | | | 1 |  |
| 9750 | | | | 79994020 | Nifedipine 60mg modified-release capsules | Nifedipine | | | 1 |  |
| 22696 | | | | 84383020 | Slofedipine 20mg tablets (Sterwin Medicines) | Nifedipine | | | 1 |  |
| 9553 | | | | 54678020 | Slofedipine XL 60 tablets (Zentiva) | Nifedipine | | | 1 |  |
| 57531 | | | | 1954020 | Adalat LA 60 tablets (Waymade Healthcare Plc) | Nifedipine | | | 1 |  |
| 2280 | | | | 52574020 | Adalat retard 10mg tablets (Bayer Plc) | Nifedipine | | | 1 |  |
| 49338 | | | | 1966020 | Nifedipine 20mg modified-release tablets (Alliance Healthcare (Distribution) Ltd) | Nifedipine | | | 1 |  |
| 63041 | | | | 59921020 | Nifedipine 10mg capsules (Mylan) | Nifedipine | | | 1 |  |
| 2746 | | | | 73759020 | Coracten SR 10mg capsules (UCB Pharma Ltd) | Nifedipine | | | 1 |  |
| 17338 | | | | 84579020 | Nifedotard 20 mr 20mg Modified-release tablet (Galen Ltd) | Nifedipine | | | 1 |  |
| 46887 | | | | 99251020 | Adanif XL 60mg tablets (Focus Pharmaceuticals Ltd) | Nifedipine | | | 1 |  |
| 17342 | | | | 86339020 | Nivaten retard 10mg Modified-release tablet (Actavis UK Ltd) | Nifedipine | | | 1 |  |
| 43222 | | | | 97381020 | Valni 20 Retard tablets (Tillomed Laboratories Ltd) | Nifedipine | | | 1 |  |
| 2343 | | | | 52575020 | Adalat retard 20mg tablets (Bayer Plc) | Nifedipine | | | 1 |  |
| 46445 | | | | 54159020 | Nifedipine 10mg capsules (IVAX Pharmaceuticals UK Ltd) | Nifedipine | | | 1 |  |
| 37184 | | | | 94184020 | Valni XL 30mg tablets (Zentiva) | Nifedipine | | | 1 |  |
| 47707 | | | | 93331020 | Nifedipine Oral solution | Nifedipine | | | 1 |  |
| 63246 | | | | 44233021 | Nifedipine 10mg modified-release tablets (AM Distributions (Yorkshire) Ltd) | Nifedipine | | | 1 |  |
| 55824 | | | | 59219020 | Nifedipine 20mg Modified-release tablet (Berk Pharmaceuticals Ltd) | Nifedipine | | | 1 |  |
| 66236 | | | | 63407020 | Nifedipine 20mg Modified-release tablet (Kent Pharmaceuticals Ltd) | Nifedipine | | | 1 |  |
| 15117 | | | | 68850020 | Nifedipine with atenolol 20mg + 50mg Capsule | Nifedipine/Atenolol | | | 0 | 1 |
| 10595 | | | | 68599020 | Nimotop 30mg tablets (Bayer Plc) | Nimodipine | | | 1 |  |
| 11547 | | | | 74190020 | Nimodipine 30mg tablets | Nimodipine | | | 1 |  |
| 31337 | | | | 83488020 | Syscor MR 20 tablets (Forest Laboratories UK Ltd) | Nisoldipine | | | 1 |  |
| 19129 | | | | 83487020 | Syscor MR 10 tablets (Forest Laboratories UK Ltd) | Nisoldipine | | | 1 |  |
| 31336 | | | | 83489020 | Syscor MR 30 tablets (Forest Laboratories UK Ltd) | Nisoldipine | | | 1 |  |
| 18038 | | | | 83479020 | Nisoldipine 20mg modified-release tablets | Nisoldipine | | | 1 |  |
| 23823 | | | | 83480020 | Nisoldipine 30mg modified-release tablets | Nisoldipine | | | 1 |  |
| 23805 | | | | 83478020 | Nisoldipine 10mg modified-release tablets | Nisoldipine | | | 1 |  |
| 40668 | | | | 96927020 | Olmesartan medoxomil 40mg / Amlodipine 10mg tablets | Olmesartan medoxomil/Amlodipine besilate | | | 0 | 1 |
| 40639 | | | | 96925020 | Olmesartan medoxomil 40mg / Amlodipine 5mg tablets | Olmesartan medoxomil/Amlodipine besilate | | | 0 | 1 |
| 40316 | | | | 96923020 | Olmesartan medoxomil 20mg / Amlodipine 5mg tablets | Olmesartan medoxomil/Amlodipine besilate | | | 0 | 1 |
| 39984 | | | | 96929020 | Sevikar 20mg/5mg tablets (Daiichi Sankyo UK Ltd) | Olmesartan medoxomil/Amlodipine besilate | | | 0 | 1 |
| 41203 | | | | 96933020 | Sevikar 40mg/10mg tablets (Daiichi Sankyo UK Ltd) | Olmesartan medoxomil/Amlodipine besilate | | | 0 | 1 |
| 41205 | | | | 96931020 | Sevikar 40mg/5mg tablets (Daiichi Sankyo UK Ltd) | Olmesartan medoxomil/Amlodipine besilate | | | 0 | 1 |
| 60067 | | | | 22976021 | Perindopril erbumine 4mg / Amlodipine 5mg tablets | Perindopril erbumine/Amlodipine besilate | | | 0 | 1 |
| 60684 | | | | 22974021 | Perindopril erbumine 4mg / Amlodipine 10mg tablets | Perindopril erbumine/Amlodipine besilate | | | 0 | 1 |
| 35343 | | | | 92765020 | Amlodipine 5mg / Valsartan 160mg tablets | Valsartan/Amlodipine besilate | | | 0 | 1 |
| 35697 | | | | 92771020 | Exforge 5mg/160mg tablets (Novartis Pharmaceuticals UK Ltd) | Valsartan/Amlodipine besilate | | | 0 | 1 |
| 26252 | | | | 53578020 | Berkatens 160mg Tablet (Berk Pharmaceuticals Ltd) | Verapamil hydrochloride | | | 1 |  |
| 8975 | | | | 68473020 | Verapamil 180mg modified-release capsules | Verapamil hydrochloride | | | 1 |  |
| 45308 | | | | 71935020 | Verapamil 240mg modified-release tablets (Mylan) | Verapamil hydrochloride | | | 1 |  |
| 17599 | | | | 88811020 | Verapress MR 240mg tablets (Sandoz Ltd) | Verapamil hydrochloride | | | 1 |  |
| 12392 | | | | 68478020 | Univer 180mg modified-release capsules (Teva UK Ltd) | Verapamil hydrochloride | | | 1 |  |
| 13856 | | | | 84570020 | Verapress MR 240mg tablets (Actavis UK Ltd) | Verapamil hydrochloride | | | 1 |  |
| 32590 | | | | 60639020 | Verapamil 40mg tablets (Mylan) | Verapamil hydrochloride | | | 1 |  |
| 46009 | | | | 63423020 | Verapamil 120mg tablets (Kent Pharmaceuticals Ltd) | Verapamil hydrochloride | | | 1 |  |
| 19457 | | | | 84468020 | Ranvera MR 240mg tablets (Ranbaxy (UK) Ltd) | Verapamil hydrochloride | | | 1 |  |
| 35729 | | | | 54551020 | Verapamil 80mg tablets (Teva UK Ltd) | Verapamil hydrochloride | | | 1 |  |
| 13251 | | | | 78368020 | Vera-Til SR 240mg tablets (Tillomed Laboratories Ltd) | Verapamil hydrochloride | | | 1 |  |
| 47222 | | | | 75703020 | Verapamil 120mg modified-release tablets (A A H Pharmaceuticals Ltd) | Verapamil hydrochloride | | | 1 |  |
| 700 | | | | 84862020 | Vera-Til SR 120mg tablets (Tillomed Laboratories Ltd) | Verapamil hydrochloride | | | 1 |  |
| 28843 | | | | 50788020 | Verapamil hc 80mg Tablet (Celltech Pharma Europe Ltd) | Verapamil hydrochloride | | | 1 |  |
| 33471 | | | | 56374020 | Verapamil 40mg tablets (Actavis UK Ltd) | Verapamil hydrochloride | | | 1 |  |
| 68531 | | | | 33673020 | Verapamil 40mg/5ml oral suspension | Verapamil hydrochloride | | | 1 |  |
| 16328 | | | | 78409020 | Verapress MR 240mg tablets (Dexcel-Pharma Ltd) | Verapamil hydrochloride | | | 1 |  |
| 8759 | | | | 56680020 | Verapamil hcl 120mg modified release tablets | Verapamil Hydrochloride | | | 1 |  |
| 8945 | | | | 68479020 | Univer 240mg modified-release capsules (Teva UK Ltd) | Verapamil hydrochloride | | | 1 |  |
| 6510 | | | | 68477020 | Univer 120mg modified-release capsules (Teva UK Ltd) | Verapamil hydrochloride | | | 1 |  |
| 59264 | | | | 872020 | Securon SR 240mg tablets (DE Pharmaceuticals) | Verapamil hydrochloride | | | 1 |  |
| 8524 | | | | 58375020 | Securon 40mg Tablet (Abbott Laboratories Ltd) | Verapamil hydrochloride | | | 1 |  |
| 12104 | | | | 53065020 | Cordilox 160mg tablets (IVAX Pharmaceuticals UK Ltd) | Verapamil hydrochloride | | | 1 |  |
| 3057 | | | | 58377020 | Securon 120mg tablets (Abbott Laboratories Ltd) | Verapamil hydrochloride | | | 1 |  |
| 41693 | | | | 60641020 | Verapamil 120mg tablets (Mylan) | Verapamil hydrochloride | | | 1 |  |
| 1118 | | | | 65395020 | Verapamil 40mg tablets | Verapamil hydrochloride | | | 1 |  |
| 11972 | | | | 86724020 | Vertab SR 240 tablets (Chiesi Ltd) | Verapamil hydrochloride | | | 1 |  |
| 28844 | | | | 53573020 | Berkatens 120mg Tablet (Berk Pharmaceuticals Ltd) | Verapamil hydrochloride | | | 1 |  |
| 9569 | | | | 65404020 | Verapamil 120mg modified-release tablets | Verapamil hydrochloride | | | 1 |  |
| 1298 | | | | 65403020 | Verapamil 240mg modified-release tablets | Verapamil hydrochloride | | | 1 |  |
| 34959 | | | | 50799020 | Verapamil 40mg tablets (A A H Pharmaceuticals Ltd) | Verapamil hydrochloride | | | 1 |  |
| 19459 | | | | 51963020 | Verapamil 240mg modified-release tablets (A A H Pharmaceuticals Ltd) | Verapamil hydrochloride | | | 1 |  |
| 47230 | | | | 59413020 | Verapamil 240mg modified-release tablets (Teva UK Ltd) | Verapamil hydrochloride | | | 1 |  |
| 46884 | | | | 65991020 | Verapamil hc 240mg Modified-release tablet (Sandoz Ltd) | Verapamil hydrochloride | | | 1 |  |
| 71009 | | | | 65538020 | Verapamil 40mg tablets (Kent Pharmaceuticals Ltd) | Verapamil hydrochloride | | | 1 |  |
| 13965 | | | | 71435020 | Cordilox MR 240mg tablets (Teva UK Ltd) | Verapamil hydrochloride | | | 1 |  |
| 3343 | | | | 56683020 | Half Securon SR 120mg tablets (Mylan) | Verapamil hydrochloride | | | 1 |  |
| 30462 | | | | 85740020 | Ethimil MR 240mg tablets (Genus Pharmaceuticals Ltd) | Verapamil hydrochloride | | | 1 |  |
| 39009 | | | | 54550020 | Verapamil 40mg tablets (Teva UK Ltd) | Verapamil hydrochloride | | | 1 |  |
| 10688 | | | | 69592020 | Verapamil 160mg tablets | Verapamil hydrochloride | | | 1 |  |
| 1120 | | | | 65396020 | Verapamil 80mg tablets | Verapamil hydrochloride | | | 1 |  |
| 1748 | | | | 53062020 | Cordilox 120mg tablets (IVAX Pharmaceuticals UK Ltd) | Verapamil hydrochloride | | | 1 |  |
| 41679 | | | | 56209020 | Verapamil 80mg tablets (IVAX Pharmaceuticals UK Ltd) | Verapamil hydrochloride | | | 1 |  |
| 16677 | | | | 48759020 | Cordilox 80mg tablets (IVAX Pharmaceuticals UK Ltd) | Verapamil hydrochloride | | | 1 |  |
| 8884 | | | | 48758020 | Cordilox 40mg tablets (IVAX Pharmaceuticals UK Ltd) | Verapamil hydrochloride | | | 1 |  |
| 1574 | | | | 68472020 | Verapamil 120mg modified-release capsules | Verapamil hydrochloride | | | 1 |  |
| 1747 | | | | 65397020 | Verapamil 120mg tablets | Verapamil hydrochloride | | | 1 |  |
| 40405 | | | | 54552020 | Verapamil 120mg tablets (Teva UK Ltd) | Verapamil hydrochloride | | | 1 |  |
| 3342 | | | | 58870020 | Securon SR 240mg tablets (Mylan) | Verapamil hydrochloride | | | 1 |  |
| 41586 | | | | 56375020 | Verapamil 80mg tablets (Actavis UK Ltd) | Verapamil hydrochloride | | | 1 |  |
| 62552 | | | | 861020 | Verapamil 80mg tablets (Alliance Healthcare (Distribution) Ltd) | Verapamil hydrochloride | | | 1 |  |
| 43879 | | | | 97884020 | Vera-Til SR 240mg tablets (Actavis UK Ltd) | Verapamil hydrochloride | | | 1 |  |
| 46955 | | | | 60640020 | Verapamil 80mg tablets (Mylan) | Verapamil hydrochloride | | | 1 |  |
| 22826 | | | | 56814020 | Securon 160mg Tablet (Abbott Laboratories Ltd) | Verapamil hydrochloride | | | 1 |  |
| 19175 | | | | 56208020 | Verapamil 40mg tablets (IVAX Pharmaceuticals UK Ltd) | Verapamil hydrochloride | | | 1 |  |
| 23872 | | | | 53571020 | Berkatens 40mg Tablet (Berk Pharmaceuticals Ltd) | Verapamil hydrochloride | | | 1 |  |
| 25059 | | | | 53572020 | Berkatens 80mg Tablet (Berk Pharmaceuticals Ltd) | Verapamil hydrochloride | | | 1 |  |
| 31711 | | | | 50800020 | Verapamil 80mg tablets (A A H Pharmaceuticals Ltd) | Verapamil hydrochloride | | | 1 |  |
| 3943 | | | | 68474020 | Verapamil 240mg modified-release capsules | Verapamil hydrochloride | | | 1 |  |
| 42625 | | | | 97882020 | Vera-Til SR 120mg tablets (Actavis UK Ltd) | Verapamil hydrochloride | | | 1 |  |
| 45051 | | | | 56381020 | Verapamil hc 240mg Modified-release tablet (Actavis UK Ltd) | Verapamil hydrochloride | | | 1 |  |
| 31490 | | | | 81761020 | Zolvera 40mg/5ml oral solution (Rosemont Pharmaceuticals Ltd) | Verapamil hydrochloride | | | 1 |  |
| 67293 | | | | 876020 | Half Securon SR 120mg tablets (Mawdsley-Brooks & Company Ltd) | Verapamil hydrochloride | | | 1 |  |
| 11777 | | | | 75258020 | Verapamil 40mg/5ml oral solution sugar free | Verapamil hydrochloride | | | 1 |  |
| 51461 | | | | 865020 | Securon SR 240mg tablets (Waymade Healthcare Plc) | Verapamil hydrochloride | | | 1 |  |
| 10832 | | | | 58376020 | Securon 80mg Tablet (Abbott Laboratories Ltd) | Verapamil hydrochloride | | | 1 |  |
| 29637 | | | | 89660020 | Verapress MR 240mg tablets (Teva UK Ltd) | Verapamil hydrochloride | | | 1 |  |
| 19690 | | | | 85865020 | Verapamil 180mg modified-release / Trandolapril 2mg capsules | Verapamil hydrochloride/Trandolapril | | | 0 | 1 |
| 18223 | | | | 85883020 | Trandolapril with verapamil 2mg + 180mg Modified-release capsule | Verapamil Hydrochloride/Trandolapril | | | 0 | 1 |
| 20579 | 85903020 | Tarka modified-release capsules (Abbott Laboratories Ltd) | Verapamil hydrochloride/Trandolapril | | | | 0 | 1 | | |

**Supplementary Table 11. Code list for myocardial infarction**

CCB, calcium channel blockers

| **Myocardial infarction** | |  |
| --- | --- | --- |
| **medcode** | **readcode** | **readterm** |
| 7783 | 323..00 | ECG: myocardial infarction |
| 26975 | 3233.00 | ECG: antero-septal infarct. |
| 26972 | 3234.00 | ECG:posterior/inferior infarct |
| 55401 | 3235.00 | ECG: subendocardial infarct |
| 52705 | 3236.00 | ECG: lateral infarction |
| 59032 | 323Z.00 | ECG: myocardial infarct NOS |
| 61670 | 889A.00 | Diab mellit insulin-glucose infus acute myocardial infarct |
| 241 | G30..00 | Acute myocardial infarction |
| 13566 | G30..11 | Attack - heart |
| 2491 | G30..12 | Coronary thrombosis |
| 30421 | G30..13 | Cardiac rupture following myocardial infarction (MI) |
| 1204 | G30..14 | Heart attack |
| 1677 | G30..15 | MI - acute myocardial infarction |
| 13571 | G30..16 | Thrombosis - coronary |
| 17689 | G30..17 | Silent myocardial infarction |
| 12139 | G300.00 | Acute anterolateral infarction |
| 5387 | G301.00 | Other specified anterior myocardial infarction |
| 40429 | G301000 | Acute anteroapical infarction |
| 17872 | G301100 | Acute anteroseptal infarction |
| 14897 | G301z00 | Anterior myocardial infarction NOS |
| 8935 | G302.00 | Acute inferolateral infarction |
| 29643 | G303.00 | Acute inferoposterior infarction |
| 23892 | G304.00 | Posterior myocardial infarction NOS |
| 14898 | G305.00 | Lateral myocardial infarction NOS |
| 63467 | G306.00 | True posterior myocardial infarction |
| 3704 | G307.00 | Acute subendocardial infarction |
| 9507 | G307000 | Acute non-Q wave infarction |
| 10562 | G307100 | Acute non-ST segment elevation myocardial infarction |
| 1678 | G308.00 | Inferior myocardial infarction NOS |
| 30330 | G309.00 | Acute Q-wave infarct |
| 17133 | G30A.00 | Mural thrombosis |
| 32854 | G30B.00 | Acute posterolateral myocardial infarction |
| 29758 | G30X.00 | Acute transmural myocardial infarction of unspecif site |
| 12229 | G30X000 | Acute ST segment elevation myocardial infarction |
| 34803 | G30y.00 | Other acute myocardial infarction |
| 28736 | G30y000 | Acute atrial infarction |
| 62626 | G30y100 | Acute papillary muscle infarction |
| 41221 | G30y200 | Acute septal infarction |
| 46017 | G30yz00 | Other acute myocardial infarction NOS |
| 14658 | G30z.00 | Acute myocardial infarction NOS |
| 15661 | G310.11 | Dressler's syndrome |
| 68357 | G31y100 | Microinfarction of heart |
| 18842 | G35..00 | Subsequent myocardial infarction |
| 45809 | G350.00 | Subsequent myocardial infarction of anterior wall |
| 38609 | G351.00 | Subsequent myocardial infarction of inferior wall |
| 72562 | G353.00 | Subsequent myocardial infarction of other sites |
| 46166 | G35X.00 | Subsequent myocardial infarction of unspecified site |
| 36423 | G36..00 | Certain current complication follow acute myocardial infarct |
| 24126 | G360.00 | Haemopericardium/current comp folow acut myocard infarct |
| 23708 | G361.00 | Atrial septal defect/curr comp folow acut myocardal infarct |
| 37657 | G362.00 | Ventric septal defect/curr comp fol acut myocardal infarctn |
| 59189 | G363.00 | Ruptur cardiac wall w'out haemopericard/cur comp fol ac MI |
| 59940 | G364.00 | Ruptur chordae tendinae/curr comp fol acute myocard infarct |
| 69474 | G365.00 | Rupture papillary muscle/curr comp fol acute myocard infarct |
| 29553 | G366.00 | Thrombosis atrium,auric append&vent/curr comp foll acute MI |
| 32272 | G38..00 | Postoperative myocardial infarction |
| 46112 | G380.00 | Postoperative transmural myocardial infarction anterior wall |
| 46276 | G381.00 | Postoperative transmural myocardial infarction inferior wall |
| 106812 | G383.00 | Postoperative transmural myocardial infarction unspec site |
| 41835 | G384.00 | Postoperative subendocardial myocardial infarction |
| 68748 | G38z.00 | Postoperative myocardial infarction, unspecified |
| 35119 | G501.00 | Post infarction pericarditis |
| 96838 | Gyu3400 | [X]Acute transmural myocardial infarction of unspecif site |

**Supplementary Table 12. Code list for stroke**

| **Stroke** | |  |  |  |
| --- | --- | --- | --- | --- |
| **Med**  **code** | **Read**  **code** | **readterm** | **Haemorrhagic**  **stroke** | **Ischaemic**  **stroke** |
| 1786 | G60..00 | Subarachnoid haemorrhage | 1 |  |
| 29939 | G600.00 | Ruptured berry aneurysm | 1 |  |
| 56007 | G601.00 | Subarachnoid haemorrhage from carotid siphon and bifurcation | 1 |  |
| 19412 | G602.00 | Subarachnoid haemorrhage from middle cerebral artery | 1 |  |
| 42331 | G603.00 | Subarachnoid haemorrhage from anterior communicating artery | 1 |  |
| 9696 | G604.00 | Subarachnoid haemorrhage from posterior communicating artery | 1 |  |
| 41910 | G605.00 | Subarachnoid haemorrhage from basilar artery | 1 |  |
| 60692 | G606.00 | Subarachnoid haemorrhage from vertebral artery | 1 |  |
| 17326 | G60X.00 | Subarachnoid haemorrh from intracranial artery, unspecif | 1 |  |
| 23580 | G60z.00 | Subarachnoid haemorrhage NOS | 1 |  |
| 5051 | G61..00 | Intracerebral haemorrhage | 1 |  |
| 6960 | G61..11 | CVA - cerebrovascular accid due to intracerebral haemorrhage | 1 |  |
| 18604 | G61..12 | Stroke due to intracerebral haemorrhage | 1 |  |
| 31595 | G610.00 | Cortical haemorrhage | 1 |  |
| 40338 | G611.00 | Internal capsule haemorrhage | 1 |  |
| 46316 | G612.00 | Basal nucleus haemorrhage | 1 |  |
| 13564 | G613.00 | Cerebellar haemorrhage | 1 |  |
| 7912 | G614.00 | Pontine haemorrhage | 1 |  |
| 62342 | G615.00 | Bulbar haemorrhage | 1 |  |
| 30045 | G616.00 | External capsule haemorrhage | 1 |  |
| 30202 | G617.00 | Intracerebral haemorrhage, intraventricular | 1 |  |
| 57315 | G618.00 | Intracerebral haemorrhage, multiple localized | 1 |  |
| 107440 | G619.00 | Lobar cerebral haemorrhage | 1 |  |
| 31060 | G61X.00 | Intracerebral haemorrhage in hemisphere, unspecified | 1 |  |
| 28314 | G61X000 | Left sided intracerebral haemorrhage, unspecified | 1 |  |
| 19201 | G61X100 | Right sided intracerebral haemorrhage, unspecified | 1 |  |
| 3535 | G61z.00 | Intracerebral haemorrhage NOS | 1 |  |
| 31805 | G62..00 | Other and unspecified intracranial haemorrhage | 1 |  |
| 36178 | G620.00 | Extradural haemorrhage - nontraumatic | 1 |  |
| 4273 | G621.00 | Subdural haemorrhage - nontraumatic | 1 |  |
| 17734 | G622.00 | Subdural haematoma - nontraumatic | 1 |  |
| 18912 | G623.00 | Subdural haemorrhage NOS | 1 |  |
| 20284 | G62z.00 | Intracranial haemorrhage NOS | 1 |  |
| 23671 | G63y000 | Cerebral infarct due to thrombosis of precerebral arteries |  | 1 |
| 24446 | G63y100 | Cerebral infarction due to embolism of precerebral arteries |  | 1 |
| 8837 | G64..00 | Cerebral arterial occlusion |  | 1 |
| 5363 | G64..11 | CVA - cerebral artery occlusion |  | 1 |
| 569 | G64..12 | Infarction - cerebral |  | 1 |
| 6155 | G64..13 | Stroke due to cerebral arterial occlusion |  | 1 |
| 16517 | G640.00 | Cerebral thrombosis |  | 1 |
| 36717 | G640000 | Cerebral infarction due to thrombosis of cerebral arteries |  | 1 |
| 15019 | G641.00 | Cerebral embolism |  | 1 |
| 34758 | G641.11 | Cerebral embolus |  | 1 |
| 27975 | G641000 | Cerebral infarction due to embolism of cerebral arteries |  | 1 |
| 3149 | G64z.00 | Cerebral infarction NOS |  | 1 |
| 15252 | G64z.11 | Brainstem infarction NOS |  | 1 |
| 5602 | G64z.12 | Cerebellar infarction |  | 1 |
| 25615 | G64z000 | Brainstem infarction |  | 1 |
| 9985 | G64z200 | Left sided cerebral infarction |  | 1 |
| 10504 | G64z300 | Right sided cerebral infarction |  | 1 |
| 26424 | G64z400 | Infarction of basal ganglia |  | 1 |
| 1469 | G66..00 | Stroke and cerebrovascular accident unspecified |  |  |
| 1298 | G66..11 | CVA unspecified |  |  |
| 6253 | G66..12 | Stroke unspecified |  |  |
| 6116 | G66..13 | CVA - Cerebrovascular accident unspecified |  |  |
| 8443 | G663.00 | Brain stem stroke syndrome |  |  |
| 17322 | G664.00 | Cerebellar stroke syndrome |  |  |
| 7780 | G667.00 | Left sided CVA |  |  |
| 12833 | G668.00 | Right sided CVA |  | 1 |
| 40053 | G671.00 | Generalised ischaemic cerebrovascular disease NOS |  | 1 |
| 70536 | G671000 | Acute cerebrovascular insufficiency NOS |  | 1 |
| 12555 | G671z00 | Generalised ischaemic cerebrovascular disease NOS |  | 1 |
| 39344 | G676000 | Cereb infarct due cerebral venous thrombosis, nonpyogenic |  | 1 |
| 51759 | G677000 | Occlusion and stenosis of middle cerebral artery |  | 1 |
| 57527 | G677100 | Occlusion and stenosis of anterior cerebral artery |  | 1 |
| 65770 | G677200 | Occlusion and stenosis of posterior cerebral artery |  | 1 |
| 55602 | G677300 | Occlusion and stenosis of cerebellar arteries |  | 1 |
| 71274 | G677400 | Occlusion+stenosis of multiple and bilat cerebral arteries |  | 1 |
| 101733 | G67A.00 | Cerebral vein thrombosis |  | 1 |

**Supplementary Table 13. Code list for platelet aggregation inhibitors**

| **Platelet aggregation inhibitors** | | |  |
| --- | --- | --- | --- |
| **prodcode** | **gemscriptcode** | **productname** | **drugsubstancename** |
| 3 | 52994020 | Aspirin 75mg dispersible tablets | Aspirin |
| 16 | 72648020 | Aspirin 75mg tablets | Aspirin |
| 34 | 52995020 | Aspirin 75mg gastro-resistant tablets | Aspirin |
| 254 | 54359020 | Aspirin 300mg tablets | Aspirin |
| 377 | 54360020 | Aspirin 300mg dispersible tablets | Aspirin |
| 393 | 73356020 | Disprin 300mg dispersible tablets (Reckitt Benckiser Healthcare (UK) Ltd) | Aspirin |
| 434 | 70336020 | Aspirin 300mg gastro-resistant tablets | Aspirin |
| 645 | 75037020 | Aspirin 300mg suppositories | Aspirin |
| 657 | 86989020 | Aspirin 500mg granules sachets sugar free | Aspirin |
| 1049 | 50796020 | Nu-seals aspirin 600mg Tablet (Eli Lilly and Company Ltd) | Aspirin |
| 1137 | 50795020 | Nu-seals aspirin ec 300mg Gastro-resistant tablet (Eli Lilly and Company Ltd) | Aspirin |
| 1902 | 70337020 | Aspirn 600mg gastro-resistant tablets | Aspirin |
| 2105 | 58313020 | Solprin 300mg Tablet (Reckitt Benckiser Healthcare (UK) Ltd) | Aspirin |
| 2607 | 70474020 | Paynocil Tablet (Beecham Research Laboratories) | Aspirin |
| 2628 | 50797020 | Nu-seals aspirin ec 75mg Gastro-resistant tablet (Eli Lilly and Company Ltd) | Aspirin |
| 6006 | 84142020 | Nu-Seals 75 gastro-resistant tablets (Alliance Pharmaceuticals Ltd) | Aspirin |
| 6007 | 84144020 | Nu-Seals 300 gastro-resistant tablets (Alliance Pharmaceuticals Ltd) | Aspirin |
| 6696 | 77773020 | Micropirin 75mg gastro-resistant tablets (Dexcel-Pharma Ltd) | Aspirin |
| 7516 | 68709020 | Aspirin 300mg effervescent tablets sugar free | Aspirin |
| 8185 | 74303020 | Disprin CV 300mg modified-release tablets (Reckitt Benckiser Healthcare (UK) Ltd) | Aspirin |
| 8186 | 70462020 | Aspirin 300mg modified-release tablets | Aspirin |
| 8645 | 69426020 | Aspirin 300mg effervescent tablets | Aspirin |
| 9144 | 80899020 | Caprin 75mg gastro-resistant tablets (Wockhardt UK Ltd) | Aspirin |
| 9301 | 52996020 | Aspirin 100mg modified-release tablets | Aspirin |
| 9939 | 68708020 | Aspirin 500mg effervescent tablets sugar free | Aspirin |
| 10305 | 68710020 | Aspirin 162.5mg capsules | Aspirin |
| 10310 | 52556020 | Aspirin powder | Aspirin |
| 11977 | 81582020 | Aspro clear maximum strength tablets | Aspirin |
| 15364 | 75038020 | Aspirin 150mg suppositories | Aspirin |
| 17704 | 69134020 | Platet 100mg Effervescent tablet (Roche Products Ltd) | Aspirin |
| 17920 | 74302020 | Disprin cv 100mg Modified-release tablet (Reckitt Benckiser Healthcare (UK) Ltd) | Aspirin |
| 18217 | 78272020 | Aspirin 300mg orodispersible tablets sugar free | Aspirin |
| 18329 | 84994020 | Enprin 75mg gastro-resistant tablets (Galpharm International Ltd) | Aspirin |
| 19189 | 82090020 | Micropirin 75mg Gastro-resistant tablet (Ratiopharm UK Ltd) | Aspirin |
| 21921 | 75686020 | Postmi ec 300mg Gastro-resistant tablet (Ashbourne Pharmaceuticals Ltd) | Aspirin |
| 22138 | 70461020 | Aspirin 324mg modified-release tablets | Aspirin |
| 22232 | 75436020 | Disprin Direct 300mg orodispersible tablets (Reckitt Benckiser Healthcare (UK) Ltd) | Aspirin |
| 22618 | 59214020 | Solprin 75mg Tablet (Reckitt Benckiser Healthcare (UK) Ltd) | Aspirin |
| 23488 | 48698020 | Claradin 300mg Tablet (Nicholas Laboratories Ltd) | Aspirin |
| 23593 | 79130020 | PostMI 75 dispersible tablets (Ashbourne Pharmaceuticals Ltd) | Aspirin |
| 23878 | 83262020 | Nu-seals cardio ec 75mg Gastro-resistant tablet (Genus Pharmaceuticals Ltd) | Aspirin |
| 23932 | 81581020 | Aspro Clear 300mg effervescent tablets (Bayer Plc) | Aspirin |
| 24025 | 70471020 | Caprin 300mg gastro-resistant tablets (Pinewood Healthcare) | Aspirin |
| 24960 | 56223020 | Aspirin 300mg tablets (Vantage) | Aspirin |
| 25335 | 79131020 | PostMI 75 EC tablets (Ashbourne Pharmaceuticals Ltd) | Aspirin |
| 25718 | 72645020 | Angettes 75 tablets (Bristol-Myers Squibb Pharmaceuticals Ltd) | Aspirin |
| 28810 | 70466020 | Aspirin 300mg with Glycine 133mg soluble tablets | Aspirin |
| 29759 | 81579020 | Aspro Tablet (Roche Consumer Health) | Aspirin |
| 29848 | 70467020 | Aspirin 300mg with Glycine 150mg chewable tablets | Aspirin |
| 30920 | 63543020 | Aspirin 300mg Dispersible tablet (M & A Pharmachem Ltd) | Aspirin |
| 31210 | 54011020 | Aspirin 300mg Tablet (Co-operative) | Aspirin |
| 31211 | 56282020 | Aspirin 75mg Dispersible tablet (A A H Pharmaceuticals Ltd) | Aspirin |
| 31858 | 86558020 | Caspac xl 162.5mg Capsule (Pharmacia Ltd) | Aspirin |
| 31870 | 54361020 | Aspirin 320mg tablets | Aspirin |
| 31938 | 57868020 | Aspirin 75mg gastro-resistant tablets (Sandoz Ltd) | Aspirin |
| 31953 | 58872020 | Aspirin 75mg dispersible tablets (IVAX Pharmaceuticals UK Ltd) | Aspirin |
| 31954 | 53701020 | Aspirin 75mg dispersible tablets (Teva UK Ltd) | Aspirin |
| 31956 | 62304020 | Aspirin 75mg gastro-resistant tablets (Kent Pharmaceuticals Ltd) | Aspirin |
| 32036 | 48769020 | Aspirin 75mg dispersible tablets (Actavis UK Ltd) | Aspirin |
| 32210 | 48770020 | Aspirin 300mg dispersible tablets (Actavis UK Ltd) | Aspirin |
| 32992 | 62496020 | Aspirin 75mg gastro-resistant tablets (Mylan) | Aspirin |
| 33293 | 62271020 | Aspirin 75mg gastro-resistant tablets (Sterwin Medicines) | Aspirin |
| 33320 | 66537020 | Aspirin 75mg Dispersible tablet (Sovereign Medical Ltd) | Aspirin |
| 33656 | 48774020 | Aspirin 75mg dispersible tablets (A A H Pharmaceuticals Ltd) | Aspirin |
| 33662 | 56283020 | Aspirin 300mg Dispersible tablet (A A H Pharmaceuticals Ltd) | Aspirin |
| 33668 | 62403020 | Aspirin 300mg Dispersible tablet (Rusco Ltd) | Aspirin |
| 33676 | 63290020 | Aspirin 75mg dispersible tablets (Kent Pharmaceuticals Ltd) | Aspirin |
| 34309 | 48775020 | Aspirin 300mg dispersible tablets (A A H Pharmaceuticals Ltd) | Aspirin |
| 34385 | 54012020 | Aspirin 75mg Soluble tablet (Co-operative) | Aspirin |
| 34386 | 48771020 | Aspirin 300mg tablets (Actavis UK Ltd) | Aspirin |
| 34434 | 57295020 | Aspirin 75mg dispersible tablets (Thornton & Ross Ltd) | Aspirin |
| 34485 | 58873020 | Aspirin 75mg gastro-resistant tablets (IVAX Pharmaceuticals UK Ltd) | Aspirin |
| 34611 | 51009020 | Aspirin 75mg gastro-resistant tablets (C P Pharmaceuticals Ltd) | Aspirin |
| 34666 | 56222020 | Aspirin ec 300mg Gastro-resistant tablet (A A H Pharmaceuticals Ltd) | Aspirin |
| 34762 | 59131020 | Aspirin 300mg Gastro-resistant tablet (Galen Ltd) | Aspirin |
| 34796 | 59128020 | Aspirin 75mg Gastro-resistant tablet (Galen Ltd) | Aspirin |
| 34797 | 59503020 | Aspirin 75mg gastro-resistant tablets (Actavis UK Ltd) | Aspirin |
| 34942 | 66393020 | Aspirin 75mg Dispersible tablet (Nucare Plc) | Aspirin |
| 36521 | 75811020 | Aspirin 500mg modified-release tablets | Aspirin |
| 36543 | 69425020 | Aspirin 100mg effervescent tablets | Aspirin |
| 37541 | 70379020 | Aspirin 227mg medicated chewing-gum | Aspirin |
| 39738 | 96734020 | Aspirin 162.5mg modified-release capsules | Aspirin |
| 40144 | 51004020 | Aspirin 300mg Dispersible tablet (Thornton & Ross Ltd) | Aspirin |
| 40381 | 51008020 | Aspirin 75mg Soluble tablet (C P Pharmaceuticals Ltd) | Aspirin |
| 41512 | 59216020 | Aspirin 75mg gastro-resistant tablets (Teva UK Ltd) | Aspirin |
| 41569 | 48776020 | Aspirin 300mg tablets (A A H Pharmaceuticals Ltd) | Aspirin |
| 41594 | 53703020 | Aspirin 300mg Dispersible tablet (Teva UK Ltd) | Aspirin |
| 41766 | 95905020 | Maximum Strength Aspro Clear 500mg effervescent tablets (Bayer Plc) | Aspirin |
| 43060 | 48762020 | Aspirin 300mg Soluble tablet (Celltech Pharma Europe Ltd) | Aspirin |
| 43434 | 59945020 | Aspirin 300mg gastro-resistant tablets (A A H Pharmaceuticals Ltd) | Aspirin |
| 43679 | 96736020 | Flamasacard 162.5mg Modified-release capsule (Abbey Pharmaceuticals Ltd) | Aspirin |
| 43709 | 69677020 | Aspirin 75mg gastro-resistant tablets (Almus Pharmaceuticals Ltd) | Aspirin |
| 43806 | 57869020 | Aspirin 300mg gastro-resistant tablets (Sandoz Ltd) | Aspirin |
| 44639 | 66396020 | Aspirin 300mg Dispersible tablet (Nucare Plc) | Aspirin |
| 45643 | 48763020 | Aspirin 75mg Soluble tablet (Celltech Pharma Europe Ltd) | Aspirin |
| 45840 | 56069020 | Aspirin 300mg Dispersible tablet (Numark Management Ltd) | Aspirin |
| 45851 | 55399020 | Aspirin 300mg Soluble tablet (Ranbaxy (UK) Ltd) | Aspirin |
| 47937 | 78627020 | Aspirin 75mg dispersible tablets (Wockhardt UK Ltd) | Aspirin |
| 47992 | 59946020 | Aspirin 75mg gastro-resistant tablets (A A H Pharmaceuticals Ltd) | Aspirin |
| 48000 | 62226020 | Aspirin 300mg tablets (Sigma Pharmaceuticals Plc) | Aspirin |
| 48021 | 77564020 | Aspirin 75mg Tablet (Hillcross Pharmaceuticals Ltd) | Aspirin |
| 48165 | 61768020 | Aspirin 300mg tablets (Aspar Pharmaceuticals Ltd) | Aspirin |
| 48974 | 2354020 | Aspirin 75mg tablets (Phoenix Healthcare Distribution Ltd) | Aspirin |
| 49060 | 2342020 | Aspirin 75mg dispersible tablets (Alliance Healthcare (Distribution) Ltd) | Aspirin |
| 49220 | 8029020 | Aspirin 300mg tablets (Kent Pharmaceuticals Ltd) | Aspirin |
| 49685 | 2346020 | Aspirin 75mg dispersible tablets (Sigma Pharmaceuticals Plc) | Aspirin |
| 49799 | 4200020 | Aspirin 150mg suppositories (A A H Pharmaceuticals Ltd) | Aspirin |
| 50555 | 8038020 | Aspirin 300mg dispersible tablets (DE Pharmaceuticals) | Aspirin |
| 50926 | 2341020 | Aspirin 75mg dispersible tablets (The Boots Company Plc) | Aspirin |
| 50949 | 2353020 | Aspirin 75mg tablets (A A H Pharmaceuticals Ltd) | Aspirin |
| 51474 | 59817020 | Aspirin 150mg suppositories (Martindale Pharmaceuticals Ltd) | Aspirin |
| 51561 | 2360020 | Aspirin 75mg gastro-resistant tablets (Zanza Laboratories Ltd) | Aspirin |
| 52044 | 8034020 | Aspirin 300mg caplets (The Boots Company Plc) | Aspirin |
| 52280 | 78796020 | Aspirin 300mg Tablet (Wockhardt UK Ltd) | Aspirin |
| 52618 | 2347020 | Aspirin 75mg dispersible tablets (Bristol Laboratories Ltd) | Aspirin |
| 52905 | 8031020 | Aspirin 300mg tablets (Lloyds Pharmacy Ltd) | Aspirin |
| 53178 | 76832020 | Aspirin 75mg gastro-resistant tablets (Wockhardt UK Ltd) | Aspirin |
| 53622 | 63567020 | Aspirin 300mg Tablet (M & A Pharmachem Ltd) | Aspirin |
| 53711 | 66390020 | Aspirin 300mg Tablet (Nucare Plc) | Aspirin |
| 53791 | 4199020 | Aspirin 150mg suppositories (Alliance Healthcare (Distribution) Ltd) | Aspirin |
| 53804 | 4190020 | Aspirin 300mg gastro-resistant tablets (Alliance Healthcare (Distribution) Ltd) | Aspirin |
| 53816 | 8036020 | Aspirin 300mg dispersible tablets (Alliance Healthcare (Distribution) Ltd) | Aspirin |
| 54284 | 69594020 | Aspirin 75mg dispersible tablets (Almus Pharmaceuticals Ltd) | Aspirin |
| 54430 | 2352020 | Aspirin 75mg tablets (Alliance Healthcare (Distribution) Ltd) | Aspirin |
| 54526 | 8030020 | Aspirin 300mg tablets (Alliance Healthcare (Distribution) Ltd) | Aspirin |
| 54565 | 2343020 | Aspirin 75mg dispersible tablets (Lloyds Pharmacy Ltd) | Aspirin |
| 54734 | 74953020 | Aspirin 300mg tablets (Wockhardt UK Ltd) | Aspirin |
| 54997 | 2344020 | Aspirin 75mg dispersible tablets (Dowelhurst Ltd) | Aspirin |
| 55230 | 63295020 | Aspirin 300mg dispersible tablets (Kent Pharmaceuticals Ltd) | Aspirin |
| 55579 | 78846020 | Aspirin 300mg tablets (Almus Pharmaceuticals Ltd) | Aspirin |
| 56007 | 8040020 | Aspirin 300mg dispersible tablets (Sigma Pharmaceuticals Plc) | Aspirin |
| 56736 | 47552020 | Aspirin 300mg tablets (Waymade Healthcare Plc) | Aspirin |
| 56883 | 47189020 | Aspirin 75mg tablets (Waymade Healthcare Plc) | Aspirin |
| 56995 | 2349020 | Aspirin 75mg dispersible tablets (Phoenix Healthcare Distribution Ltd) | Aspirin |
| 56996 | 47188020 | Aspirin 75mg dispersible tablets (Waymade Healthcare Plc) | Aspirin |
| 57057 | 2350020 | Aspirin 75mg dispersible tablets (Wockhardt UK Ltd) | Aspirin |
| 58331 | 75319020 | Aspirin 300mg gastro-resistant tablets (Mylan) | Aspirin |
| 59021 | 2361020 | Aspirin 75mg gastro-resistant tablets (Bristol Laboratories Ltd) | Aspirin |
| 59244 | 44166020 | Aspirin 100mg capsules | Aspirin |
| 59253 | 47190020 | Aspirin 75mg gastro-resistant tablets (Waymade Healthcare Plc) | Aspirin |
| 59728 | 2351020 | Aspirin 75mg tablets (Alissa Healthcare Research Ltd) | Aspirin |
| 59791 | 68788020 | Aspirin 75mg dispersible tablets (Aspar Pharmaceuticals Ltd) | Aspirin |
| 60127 | 16678021 | Aspirin 75mg tablets (DE Pharmaceuticals) | Aspirin |
| 60278 | 16783021 | Aspirin 300mg tablets (DE Pharmaceuticals) | Aspirin |
| 60693 | 30275020 | Aspirin 15mg/5ml oral solution | Aspirin |
| 60694 | 30279020 | Aspirin 25mg/5ml oral solution | Aspirin |
| 60777 | 16679021 | Aspirin 75mg gastro-resistant tablets (DE Pharmaceuticals) | Aspirin |
| 62334 | 29731021 | Aspirin 300mg caplets (Wockhardt UK Ltd) | Aspirin |
| 62430 | 4196020 | Aspirin 300mg suppositories (A A H Pharmaceuticals Ltd) | Aspirin |
| 63603 | 50070020 | Laboprin Tablet (Laboratories For Applied Biology Ltd) | Aspirin |
| 64071 | 67291020 | Aspirin powder (J M Loveridge Ltd) | Aspirin |
| 66345 | 16677021 | Aspirin 75mg dispersible tablets (DE Pharmaceuticals) | Aspirin |
| 66546 | 2345020 | Aspirin 75mg dispersible tablets (Numark Ltd) | Aspirin |
| 66563 | 2362020 | Aspirin 75mg gastro-resistant tablets (Phoenix Healthcare Distribution Ltd) | Aspirin |
| 66861 | 21876020 | Aspirin 75mg effervescent tablets | Aspirin |
| 67160 | 8037020 | Aspirin 300mg dispersible tablets (Lloyds Pharmacy Ltd) | Aspirin |
| 67362 | 4198020 | Aspirin 300mg suppositories (Alliance Healthcare (Distribution) Ltd) | Aspirin |
| 67521 | 30277020 | Aspirin 15mg/5ml oral suspension | Aspirin |
| 67754 | 69591020 | Aspirin 300mg dispersible tablets (Almus Pharmaceuticals Ltd) | Aspirin |
| 67858 | 60151021 | Aspirin 25mg capsules | Aspirin |
| 68051 | 17420021 | Aspirin 150mg suppositories (Colorama Pharmaceuticals Ltd) | Aspirin |
| 68752 | 52194021 | Aspirin 75mg tablets (Sigma Pharmaceuticals Plc) | Aspirin |
| 70549 | 69973021 | Danamep 75mg dispersible tablets (Ecogen Europe Ltd) | Aspirin |
| 70841 | 52904020 | Aspirin 300mg Dispersible tablet (Family Health) | Aspirin |
| 71078 | 8041020 | Aspirin 300mg dispersible tablets (Mawdsley-Brooks & Company Ltd) | Aspirin |
| 71192 | 62254021 | Aspirin 75mg tablets (Kent Pharmaceuticals Ltd) | Aspirin |
| 65027 | 44190021 | Bisoprolol 5mg / Aspirin 100mg capsules | Aspirin/Bisoprolol fumarate |
| 13882 | 80846020 | Imazin XL tablets (Napp Pharmaceuticals Ltd) | Aspirin/Isosorbide mononitrate |
| 18030 | 80848020 | Imazin XL forte tablets (Napp Pharmaceuticals Ltd) | Aspirin/Isosorbide mononitrate |
| 21380 | 80852020 | Aspirin 75mg / Isosorbide mononitrate 60mg modified-release tablets | Aspirin/Isosorbide mononitrate |
| 21382 | 80853020 | Aspirin 150mg / Isosorbide mononitrate 60mg modified-release tablets | Aspirin/Isosorbide mononitrate |
| 67124 | 44188021 | Bisoprolol 10mg / Aspirin 75mg capsules | Bisoprolol fumarate/Aspirin |
| 489 | 83784020 | Clopidogrel 75mg tablets | Clopidogrel |
| 836 | 83768020 | Plavix 75mg tablets (Sanofi) | Clopidogrel |
| 40913 | 97373020 | Grepid 75mg tablets (Kent Pharmaceuticals Ltd) | Clopidogrel |
| 42750 | 77086020 | Clopidogrel 75mg tablets (Actavis UK Ltd) | Clopidogrel |
| 52761 | 2384020 | Clopidogrel 75mg tablets (Dr Reddy's Laboratories (UK) Ltd) | Clopidogrel |
| 53751 | 2380020 | Clopidogrel 75mg tablets (Phoenix Healthcare Distribution Ltd) | Clopidogrel |
| 54700 | 76842020 | Clopidogrel 75mg tablets (A A H Pharmaceuticals Ltd) | Clopidogrel |
| 55161 | 77882020 | Clopidogrel 75mg tablets (Wockhardt UK Ltd) | Clopidogrel |
| 56807 | 76752020 | Clopidogrel 75mg tablets (Teva UK Ltd) | Clopidogrel |
| 57036 | 77043020 | Clopidogrel 75mg tablets (Mylan) | Clopidogrel |
| 58347 | 2370020 | Clopidogrel 75mg tablets (DE Pharmaceuticals) | Clopidogrel |
| 58448 | 2381020 | Clopidogrel 75mg tablets (Aspire Pharma Ltd) | Clopidogrel |
| 62855 | 2375020 | Clopidogrel 75mg tablets (Alliance Healthcare (Distribution) Ltd) | Clopidogrel |
| 62978 | 23054021 | Clopidogrel 75mg tablets (Sandoz Ltd) | Clopidogrel |
| 63450 | 46327020 | Clopidogrel 75mg tablets (Almus Pharmaceuticals Ltd) | Clopidogrel |
| 65909 | 21742021 | Clopidogrel 75mg tablets (Milpharm Ltd) | Clopidogrel |
| 67037 | 17199021 | Clopidogrel 75mg tablets (Zentiva) | Clopidogrel |
| 38349 | 94934020 | Clopidogrel 300mg tablets | Clopidogrel hydrogen sulfate |
| 38998 | 94936020 | Plavix 300mg tablets (Sanofi) | Clopidogrel hydrogen sulfate |
| 45905 | 99683020 | Clopidogrel 1mg/ml oral suspension | Clopidogrel hydrogen sulfate |
| 46891 | 396021 | Clopidogrel 75mg/5ml oral suspension | Clopidogrel hydrogen sulfate |
| 59904 | 16245021 | Clopidogrel 75mg/5ml oral solution | Clopidogrel hydrogen sulfate |
| 689 | 51077020 | Persantin 25mg tablets (Boehringer Ingelheim Ltd) | Dipyridamole |
| 714 | 62696020 | Dipyridamole 200mg modified-release capsules | Dipyridamole |
| 771 | 62694020 | Dipyridamole 25mg tablets | Dipyridamole |
| 1814 | 62695020 | Dipyridamole 100mg tablets | Dipyridamole |
| 2106 | 51078020 | Persantin 100mg tablets (Boehringer Ingelheim Ltd) | Dipyridamole |
| 3832 | 84256020 | Persantin Retard 200mg capsules (Boehringer Ingelheim Ltd) | Dipyridamole |
| 5882 | 84976020 | Dipyridamole 50mg/5ml oral suspension sugar free | Dipyridamole |
| 21989 | 63357020 | Cerebrovase 100mg Tablet (Ashbourne Pharmaceuticals Ltd) | Dipyridamole |
| 25232 | 70665020 | Vasyrol cold sore 100mg Tablet (Shire Pharmaceuticals Ltd) | Dipyridamole |
| 25284 | 63356020 | Cerebrovase 25mg Tablet (Ashbourne Pharmaceuticals Ltd) | Dipyridamole |
| 30975 | 74843020 | Modaplate 25mg Tablet (Berk Pharmaceuticals Ltd) | Dipyridamole |
| 30976 | 74842020 | Modaplate 100mg Tablet (Berk Pharmaceuticals Ltd) | Dipyridamole |
| 31192 | 70664020 | Vasyrol cold sore 25mg Tablet (Shire Pharmaceuticals Ltd) | Dipyridamole |
| 34709 | 51223020 | Dipyridamole 100mg tablets (A A H Pharmaceuticals Ltd) | Dipyridamole |
| 35108 | 91810020 | Dipyridamole 100mg/5ml oral suspension | Dipyridamole |
| 43530 | 56938020 | Dipyridamole 100mg tablets (Actavis UK Ltd) | Dipyridamole |
| 46976 | 65447020 | Dipyridamole 100mg tablets (Kent Pharmaceuticals Ltd) | Dipyridamole |
| 51358 | 60705020 | Dipyridamole 100mg tablets (Mylan) | Dipyridamole |
| 59470 | 20734021 | Persantin Retard 200mg capsules (Consilient Health Ltd) | Dipyridamole |
| 60321 | 2330020 | Dipyridamole 25mg tablets (Phoenix Healthcare Distribution Ltd) | Dipyridamole |
| 60605 | 51224020 | Dipyridamole 25mg tablets (A A H Pharmaceuticals Ltd) | Dipyridamole |
| 62707 | 2329020 | Dipyridamole 25mg tablets (Alliance Healthcare (Distribution) Ltd) | Dipyridamole |
| 64222 | 19755020 | Dipyridamole 50mg/5ml oral solution | Dipyridamole |
| 64837 | 29679021 | Ofcram PR 200mg capsules (Focus Pharmaceuticals Ltd) | Dipyridamole |
| 64929 | 19765020 | Dipyridamole 200mg/5ml oral suspension | Dipyridamole |
| 66784 | 23691021 | Attia 200mg modified-release capsules (Dr Reddy's Laboratories (UK) Ltd) | Dipyridamole |
| 66840 | 62201021 | Dipyridamole 200mg/5ml oral suspension sugar free | Dipyridamole |
| 67403 | 71796020 | Dipyridamole 100mg tablets (Dr Reddy's Laboratories (UK) Ltd) | Dipyridamole |
| 67567 | 19770020 | Dipyridamole 100mg/5ml oral solution | Dipyridamole |
| 4679 | 85728020 | Asasantin Retard capsules (Boehringer Ingelheim Ltd) | Dipyridamole/Aspirin |
| 6666 | 85724020 | Dipyridamole 200mg modified-release / Aspirin 25mg capsules | Dipyridamole/Aspirin |
| 10031 | 85731020 | Aspirin 25mg with Dipyridamole 200mg modified-release capsules | Dipyridamole/Aspirin |
| 56503 | 2365020 | Asasantin Retard capsules (Mawdsley-Brooks & Company Ltd) | Dipyridamole/Aspirin |
| 57585 | 2364020 | Asasantin Retard capsules (Dowelhurst Ltd) | Dipyridamole/Aspirin |
| 68764 | 17408021 | Molita 200mg/25mg modified-release capsules (Dr Reddy's Laboratories (UK) Ltd) | Dipyridamole/Aspirin |
| 69216 | 31328021 | Dipyridamole 200mg modified-release / Aspirin 25mg capsules (A A H Pharmaceuticals Ltd) | Dipyridamole/Aspirin |
| 39932 | 96708020 | Prasugrel 10mg tablets | Prasugrel hydrochloride |
| 40114 | 96706020 | Prasugrel 5mg tablets | Prasugrel hydrochloride |
| 40591 | 96710020 | Efient 5mg tablets (Eli Lilly and Company Ltd) | Prasugrel hydrochloride |
| 41229 | 96712020 | Efient 10mg tablets (Eli Lilly and Company Ltd) | Prasugrel hydrochloride |
| 45576 | 99283020 | Ticagrelor 90mg tablets | Ticagrelor |
| 47895 | 99285020 | Brilique 90mg tablets (AstraZeneca UK Ltd) | Ticagrelor |
| 66973 | 67744021 | Ticagrelor 60mg tablets | Ticagrelor |
| 68710 | 67745021 | Brilique 60mg tablets (AstraZeneca UK Ltd) | Ticagrelor |
| 70606 | 78181021 | Ticagrelor 90mg orodispersible tablets sugar free | Ticagrelor |
| 572 | 84320020 | Ticlopidine 250mg tablets | Ticlopidine hydrochloride |
| 17449 | 56792020 | Ticlid 250mg tablets (Sanofi-Synthelabo Ltd) | Ticlopidine hydrochloride |

**Supplementary Table 14. Code list for anticoagulants**

| **Anticoagulants** | |  |  |
| --- | --- | --- | --- |
| **prodcode** | **gemscriptcode** | **productname** | **drugsubstancename** |
| 4446 | 60995020 | Acenocoumarol 1mg tablets | Acenocoumarol |
| 15376 | 60996020 | Acenocoumarol 4mg tablets | Acenocoumarol |
| 55577 | 2314020 | Sinthrome 1mg tablets (Lexon (UK) Ltd) | Acenocoumarol |
| 5305 | 51558020 | Sinthrome 1mg tablets (Merus Labs Luxco S.a R.L.) | Acenocoumarol |
| 15006 | 51559020 | Sinthrome 4mg Tablet (Alliance Pharmaceuticals Ltd) | Acenocoumarol |
| 58594 | 46753020 | Eliquis 5mg tablets (Bristol-Myers Squibb Pharmaceuticals Ltd) | Apixaban |
| 47566 | 345021 | Apixaban 2.5mg tablets | Apixaban |
| 53740 | 347021 | Eliquis 2.5mg tablets (Bristol-Myers Squibb Pharmaceuticals Ltd) | Apixaban |
| 54066 | 46752020 | Apixaban 5mg tablets | Apixaban |
| 42474 | 94835020 | Pradaxa 75mg capsules (Boehringer Ingelheim Ltd) | Dabigatran etexilate mesilate |
| 46678 | 253021 | Pradaxa 150mg capsules (Boehringer Ingelheim Ltd) | Dabigatran etexilate mesilate |
| 39444 | 94831020 | Dabigatran etexilate 110mg capsules | Dabigatran etexilate mesilate |
| 39755 | 94837020 | Pradaxa 110mg capsules (Boehringer Ingelheim Ltd) | Dabigatran etexilate mesilate |
| 46632 | 251021 | Dabigatran etexilate 150mg capsules | Dabigatran etexilate mesilate |
| 39503 | 94833020 | Dabigatran etexilate 75mg capsules | Dabigatran etexilate mesilate |
| 16530 | 88316020 | Fragmin 12,500units/0.5ml solution for injection pre-filled syringes (Pfizer Ltd) | Dalteparin sodium |
| 19486 | 88302020 | Dalteparin sodium 7,500units/0.3ml solution for injection pre-filled syringes | Dalteparin sodium |
| 36911 | 93934020 | Fragmin 10,000units/1ml solution for injection ampoules (Pfizer Ltd) | Dalteparin sodium |
| 9140 | 54874020 | Dalteparin sodium 10,000units/4ml solution for injection ampoules | Dalteparin sodium |
| 10194 | 88306020 | Dalteparin sodium 12,500units/0.5ml solution for injection pre-filled syringes | Dalteparin sodium |
| 56398 | 9750020 | Fragmin 5,000units/0.2ml solution for injection pre-filled syringes (Mawdsley-Brooks & Company Ltd) | Dalteparin sodium |
| 10072 | 88314020 | Fragmin 10,000units/0.4ml solution for injection pre-filled syringes (Pfizer Ltd) | Dalteparin sodium |
| 49578 | 2272020 | Dalteparin sodium 10,000units/1ml solution for injection pre-filled syringes | Dalteparin sodium |
| 22428 | 75575020 | Dalteparin sodium 100,000units/4ml solution for injection vials | Dalteparin sodium |
| 16476 | 88320020 | Fragmin 18,000units/0.72ml solution for injection pre-filled syringes (Pfizer Ltd) | Dalteparin sodium |
| 55096 | 9749020 | Fragmin 5,000units/0.2ml solution for injection pre-filled syringes (Waymade Healthcare Plc) | Dalteparin sodium |
| 52004 | 2263020 | Fragmin 12,500units/0.5ml solution for injection pre-filled syringes (Waymade Healthcare Plc) | Dalteparin sodium |
| 26146 | 73245020 | Heparin low molecular weight 10,000 iu/ml Sterile solution | Dalteparin Sodium |
| 6860 | 88318020 | Fragmin 15,000units/0.6ml solution for injection pre-filled syringes (Pfizer Ltd) | Dalteparin sodium |
| 10170 | 88308020 | Dalteparin sodium 15,000units/0.6ml solution for injection pre-filled syringes | Dalteparin sodium |
| 9593 | 75576020 | Dalteparin 25000iu/ml injection solution | Dalteparin Sodium |
| 5998 | 73237020 | Fragmin 10,000 iu/ml Solution for injection (Pfizer Ltd) | Dalteparin Sodium |
| 6695 | 54876020 | Dalteparin sodium 2,500units/0.2ml solution for injection pre-filled syringes | Dalteparin sodium |
| 9605 | 75574020 | Dalteparin sodium 5,000units/0.2ml solution for injection pre-filled syringes | Dalteparin sodium |
| 10044 | 88304020 | Dalteparin sodium 10,000units/0.4ml solution for injection pre-filled syringes | Dalteparin sodium |
| 36989 | 93936020 | Fragmin 10,000units/1ml solution for injection pre-filled syringes (Pfizer Ltd) | Dalteparin sodium |
| 24896 | 73246020 | Heparin low molecular weight 2500 iu/0.2ml Sterile solution | Dalteparin Sodium |
| 5747 | 71650020 | Fragmin 25,000iu/ml Solution for injection (Pfizer Ltd) | Dalteparin Sodium |
| 50391 | 2264020 | Fragmin 18,000units/0.72ml solution for injection pre-filled syringes (Waymade Healthcare Plc) | Dalteparin sodium |
| 48673 | 2266020 | Dalteparin sodium 10,000units/1ml solution for injection ampoules | Dalteparin sodium |
| 5526 | 73238020 | Fragmin 2,500units/0.2ml solution for injection pre-filled syringes (Pfizer Ltd) | Dalteparin sodium |
| 29318 | 73244020 | Heparin low molecular weight 2500 iu/ml Sterile solution | Dalteparin Sodium |
| 2675 | 73236020 | Fragmin 10,000units/4ml solution for injection ampoules (Pfizer Ltd) | Dalteparin sodium |
| 18209 | 88312020 | Fragmin 7,500units/0.3ml solution for injection pre-filled syringes (Pfizer Ltd) | Dalteparin sodium |
| 2676 | 71648020 | Fragmin 5,000units/0.2ml solution for injection pre-filled syringes (Pfizer Ltd) | Dalteparin sodium |
| 10002 | 54875020 | Dalteparin 10000iu/1ml injection solution | Dalteparin Sodium |
| 14891 | 88310020 | Dalteparin sodium 18,000units/0.72ml solution for injection pre-filled syringes | Dalteparin sodium |
| 25155 | 71649020 | Fragmin 100,000units/4ml solution for injection vials (Pfizer Ltd) | Dalteparin sodium |
| 51350 | 2265020 | Fragmin 15,000units/0.6ml solution for injection pre-filled syringes (Waymade Healthcare Plc) | Dalteparin sodium |
| 55604 | 74751020 | Orgaran 750units/0.6ml solution for injection ampoules (Aspen Pharma Trading Ltd) | Danaparoid sodium |
| 60041 | 74748020 | Danaparoid sodium 750units/0.6ml solution for injection ampoules | Danaparoid sodium |
| 64678 | 53104021 | Edoxaban 60mg tablets | Edoxaban tosilate |
| 65247 | 53102021 | Edoxaban 30mg tablets | Edoxaban tosilate |
| 65876 | 53100021 | Edoxaban 15mg tablets | Edoxaban tosilate |
| 65850 | 53105021 | Lixiana 60mg tablets (Daiichi Sankyo UK Ltd) | Edoxaban tosilate |
| 66529 | 53103021 | Lixiana 30mg tablets (Daiichi Sankyo UK Ltd) | Edoxaban tosilate |
| 70866 | 77912021 | Inhixa 40mg/0.4ml solution for injection pre-filled syringes (Techdow Pharma England Ltd) | Enoxaparin sodium |
| 2677 | 74023020 | Clexane 100mg/ml Injection (Aventis Pharma) | Enoxaparin Sodium |
| 7307 | 87676020 | Clexane 40mg/0.4ml solution for injection pre-filled syringes (Sanofi) | Enoxaparin sodium |
| 14341 | 91199020 | Clexane Forte 150mg/1ml solution for injection pre-filled syringes (Sanofi) | Enoxaparin sodium |
| 4995 | 74027020 | Enoxaparin 100mg/ml injection | Enoxaparin Sodium |
| 14099 | 70688020 | Clexane forte 150mg/ml Injection (Aventis Pharma) | Enoxaparin Sodium |
| 13210 | 87670020 | Enoxaparin sodium 80mg/0.8ml solution for injection pre-filled syringes | Enoxaparin sodium |
| 51642 | 39295020 | Clexane 100mg/1ml solution for injection pre-filled syringes (Lexon (UK) Ltd) | Enoxaparin sodium |
| 14138 | 87668020 | Enoxaparin sodium 60mg/0.6ml solution for injection pre-filled syringes | Enoxaparin sodium |
| 71132 | 2275020 | Clexane 20mg/0.2ml solution for injection pre-filled syringes (DE Pharmaceuticals) | Enoxaparin sodium |
| 13270 | 87672020 | Enoxaparin sodium 120mg/0.8ml solution for injection pre-filled syringes | Enoxaparin sodium |
| 7199 | 87666020 | Enoxaparin sodium 40mg/0.4ml solution for injection pre-filled syringes | Enoxaparin sodium |
| 36172 | 93583020 | Clexane 300mg/3ml solution for injection multidose vials (Sanofi) | Enoxaparin sodium |
| 55565 | 2293020 | Clexane 100mg/1ml solution for injection pre-filled syringes (DE Pharmaceuticals) | Enoxaparin sodium |
| 7154 | 87682020 | Clexane Forte 120mg/0.8ml solution for injection pre-filled syringes (Sanofi) | Enoxaparin sodium |
| 13058 | 74028020 | Enoxaparin 150mg/ml injection | Enoxaparin Sodium |
| 20153 | 91195020 | Enoxaparin sodium 150mg/1ml solution for injection pre-filled syringes | Enoxaparin sodium |
| 37086 | 93581020 | Enoxaparin sodium 300mg/3ml solution for injection vials | Enoxaparin sodium |
| 13097 | 87674020 | Clexane 20mg/0.2ml solution for injection pre-filled syringes (Sanofi) | Enoxaparin sodium |
| 51006 | 2291020 | Clexane 80mg/0.8ml solution for injection pre-filled syringes (DE Pharmaceuticals) | Enoxaparin sodium |
| 10004 | 87680020 | Clexane 80mg/0.8ml solution for injection pre-filled syringes (Sanofi) | Enoxaparin sodium |
| 17664 | 87678020 | Clexane 60mg/0.6ml solution for injection pre-filled syringes (Sanofi) | Enoxaparin sodium |
| 12974 | 74024020 | Clexane 150mg/ml Injection (Aventis Pharma) | Enoxaparin Sodium |
| 64969 | 2279020 | Clexane 20mg/0.2ml solution for injection pre-filled syringes (Sigma Pharmaceuticals Plc) | Enoxaparin sodium |
| 6478 | 87662020 | Enoxaparin sodium 20mg/0.2ml solution for injection pre-filled syringes | Enoxaparin sodium |
| 7371 | 91197020 | Clexane 100mg/1ml solution for injection pre-filled syringes (Sanofi) | Enoxaparin sodium |
| 20154 | 91193020 | Enoxaparin sodium 100mg/1ml solution for injection pre-filled syringes | Enoxaparin sodium |
| 38536 | 94008020 | Fondaparinux sodium 1.5mg/0.3ml solution for injection pre-filled syringes | Fondaparinux sodium |
| 29043 | 84673020 | Arixtra 2.5mg/0.5ml solution for injection pre-filled syringes (Aspen Pharma Trading Ltd) | Fondaparinux sodium |
| 23573 | 89132020 | Fondaparinux sodium 5mg/0.4ml solution for injection pre-filled syringes | Fondaparinux sodium |
| 23570 | 89134020 | Fondaparinux sodium 7.5mg/0.6ml solution for injection pre-filled syringes | Fondaparinux sodium |
| 45911 | 94010020 | Arixtra 1.5mg/0.3ml solution for injection pre-filled syringes (Aspen Pharma Trading Ltd) | Fondaparinux sodium |
| 38327 | 89140020 | Arixtra 7.5mg/0.6ml solution for injection pre-filled syringes (Aspen Pharma Trading Ltd) | Fondaparinux sodium |
| 61949 | 89136020 | Fondaparinux sodium 10mg/0.8ml solution for injection pre-filled syringes | Fondaparinux sodium |
| 38839 | 89138020 | Arixtra 5mg/0.4ml solution for injection pre-filled syringes (Aspen Pharma Trading Ltd) | Fondaparinux sodium |
| 23579 | 77866020 | Fondaparinux sodium 2.5mg/0.5ml solution for injection pre-filled syringes | Fondaparinux sodium |
| 69194 | 71630020 | Heparin low molecular weight 5000iu/0.2ml Sterile solution | Heparin |
| 42853 | 53910020 | Heparin calcium 25,000iu/ml Injection | Heparin Calcium |
| 33558 | 71426020 | Monoparin calcium 5,000units/0.2ml solution for injection ampoules (Wockhardt UK Ltd) | Heparin calcium |
| 30108 | 91616020 | Heparin calcium 5000iu/0.2ml Injection | Heparin Calcium |
| 10533 | 58274020 | Calciparine 25,000iu/ml Injection (Sanofi-Synthelabo Ltd) | Heparin Calcium |
| 20010 | 67842020 | Uniparin calcium 25,000iu/ml Subcutaneous injection (C P Pharmaceuticals Ltd) | Heparin Calcium |
| 52841 | 2267020 | Heparin calcium 5,000units/0.2ml solution for injection ampoules | Heparin calcium |
| 62959 | 2268020 | Heparin calcium 5,000units/0.2ml solution for injection ampoules (A A H Pharmaceuticals Ltd) | Heparin calcium |
| 10532 | 52793020 | Minihep calcium 5000iu/0.2ml Injection (LEO Pharma) | Heparin Calcium |
| 12681 | 81560020 | Heparin calcium 25,000iu/ml Injection | Heparin Calcium |
| 31541 | 91618020 | Heparin calcium 12,500units/0.5ml solution for injection pre-filled syringes | Heparin calcium |
| 35955 | 91624020 | Calciparine 5,000units/0.2ml solution for injection pre-filled syringes (Sanofi-Synthelabo Ltd) | Heparin calcium |
| 57204 | 2269020 | Heparin calcium 5,000units/0.2ml solution for injection pre-filled syringes | Heparin calcium |
| 36142 | 91650020 | Heparin sodium 25,000units/1ml solution for injection ampoules | Heparin sodium |
| 60188 | 16334021 | Heparin sodium 5,000units/1litre infusion bags | Heparin sodium |
| 27035 | 63165020 | Pump-hep 1000unit/ml Infusion (LEO Pharma) | Heparin Sodium |
| 36196 | 91636020 | Heparin sodium 1,000units/1ml solution for injection ampoules | Heparin sodium |
| 42106 | 54957020 | Unihep leo 25,000iu/ml Injection (LEO Pharma) | Heparin Sodium |
| 44491 | 91634020 | Heparin sodium 125,000units/5ml solution for injection vials | Heparin sodium |
| 3895 | 73282020 | Heparin sodium 1000iu/ml Injection | Heparin Sodium |
| 25195 | 87654020 | Heparin sodium 25,000iu/ml pre-filled Injection | Heparin Sodium |
| 33307 | 91644020 | Heparin sodium 5,000units/1ml solution for injection ampoules | Heparin sodium |
| 25287 | 54952020 | Unihep leo 1000unit/ml Injection (LEO Pharma) | Heparin Sodium |
| 59761 | 73368020 | Heparin sodium 1,000units/1ml solution for injection ampoules (Wockhardt UK Ltd) | Heparin sodium |
| 54234 | 37216020 | Heparin sodium 1,000units/500ml infusion Viaflex bags (Baxter Healthcare Ltd) | Heparin sodium |
| 37616 | 73289020 | Heparin sodium 10 unit/ml Solution | Heparin Sodium |
| 35941 | 91638020 | Heparin sodium 5,000units/5ml solution for injection ampoules | Heparin sodium |
| 56166 | 37981020 | Heparin sodium 100units/1ml solution for injection ampoules | Heparin sodium |
| 21365 | 57897020 | Uniparin 5000iu/0.2ml Injection (C P Pharmaceuticals Ltd) | Heparin Sodium |
| 50994 | 37213020 | Heparin sodium 500units/500ml infusion bags | Heparin sodium |
| 40715 | 63677020 | Heparin 100iu/ml Oral solution (LEO Pharma) | Heparin Sodium |
| 30396 | 54953020 | Unihep leo 5000iu/ml Injection (LEO Pharma) | Heparin Sodium |
| 28593 | 87650020 | Heparin sodium 1000iu/ml pre-filled Injection | Heparin Sodium |
| 37678 | 91648020 | Heparin sodium 5,000units/0.2ml solution for injection ampoules | Heparin sodium |
| 37131 | 91632020 | Heparin sodium 25,000units/5ml solution for injection vials | Heparin sodium |
| 53350 | 37215020 | Heparin sodium 1,000units/500ml infusion bags | Heparin sodium |
| 13568 | 73288020 | Heparin sodium 25,000iu/ml Subcutaneous injection | Heparin Sodium |
| 37613 | 91640020 | Heparin sodium 10,000units/10ml solution for injection ampoules | Heparin sodium |
| 8664 | 73283020 | Heparin sodium 5000iu/ml Injection | Heparin Sodium |
| 19337 | 57889020 | Multiparin 125,000units/5ml solution for injection vials (Wockhardt UK Ltd) | Heparin sodium |
| 35033 | 91630020 | Heparin sodium 5,000units/5ml solution for injection vials | Heparin sodium |
| 15293 | 87652020 | Heparin sodium 5000iu/ml pre-filled Injection | Heparin Sodium |
| 20028 | 57887020 | Multiparin 5,000units/5ml solution for injection vials (Wockhardt UK Ltd) | Heparin sodium |
| 37704 | 50417020 | Minihep 25,000iu/ml Subcutaneous preparation (LEO Pharma) | Heparin Sodium |
| 14794 | 57892020 | Monoparin 1000iu/ml Injection (C P Pharmaceuticals Ltd) | Heparin Sodium |
| 20024 | 68229020 | Uniparin forte 10,000 iu/0.4ml Subcutaneous injection (C P Pharmaceuticals Ltd) | Heparin Sodium |
| 54927 | 37217020 | Heparin sodium 2,000units/1litre infusion bags | Heparin sodium |
| 64559 | 2234020 | Heparin sodium 1,000units/1ml solution for injection ampoules (A A H Pharmaceuticals Ltd) | Heparin sodium |
| 63297 | 67623020 | Heparin sodium 5,000units/5ml solution for injection vials (LEO Pharma) | Heparin sodium |
| 20029 | 57888020 | Multiparin 25,000units/5ml solution for injection vials (Wockhardt UK Ltd) | Heparin sodium |
| 21490 | 57893020 | Monoparin 5000iu/ml Injection (C P Pharmaceuticals Ltd) | Heparin Sodium |
| 47397 | 91646020 | Heparin sodium 25,000units/5ml solution for injection ampoules | Heparin sodium |
| 63146 | 91642020 | Heparin sodium 20,000units/20ml solution for injection ampoules | Heparin sodium |
| 21518 | 57894020 | Monoparin 25,000iu/ml Injection (C P Pharmaceuticals Ltd) | Heparin Sodium |
| 55490 | 73284020 | Heparin sodium 10,000 iu/ml Injection | Heparin Sodium |
| 32645 | 53913020 | Heparin sodium 25,000iu/ml Injection | Heparin Sodium |
| 13716 | 73287020 | Heparin sodium 25,000iu/ml Injection | Heparin Sodium |
| 64133 | 2271020 | Heparin sodium 5,000units/0.2ml solution for injection ampoules (A A H Pharmaceuticals Ltd) | Heparin sodium |
| 70831 | 62258020 | Phenindione 50mg tablets (AMCo) | Phenindione |
| 13644 | 54848020 | Dindevan 25mg Tablet (Goldshield Pharmaceuticals Ltd) | Phenindione |
| 13505 | 65648020 | Phenindione 10mg tablets | Phenindione |
| 46924 | 62256020 | Phenindione 10mg tablets (AMCo) | Phenindione |
| 13501 | 54849020 | Dindevan 50mg Tablet (Goldshield Pharmaceuticals Ltd) | Phenindione |
| 13502 | 54847020 | Dindevan 10mg Tablet (Goldshield Pharmaceuticals Ltd) | Phenindione |
| 13504 | 65649020 | Phenindione 25mg tablets | Phenindione |
| 13503 | 65650020 | Phenindione 50mg tablets | Phenindione |
| 56640 | 39230020 | Xarelto 15mg tablets (Bayer Plc) | Rivaroxaban |
| 56289 | 39232020 | Xarelto 20mg tablets (Bayer Plc) | Rivaroxaban |
| 54451 | 39231020 | Rivaroxaban 20mg tablets | Rivaroxaban |
| 48966 | 39229020 | Rivaroxaban 15mg tablets | Rivaroxaban |
| 39639 | 95937020 | Xarelto 10mg tablets (Bayer Plc) | Rivaroxaban |
| 39119 | 95923020 | Rivaroxaban 10mg tablets | Rivaroxaban |
| 64500 | 40546021 | Xarelto 2.5mg tablets (Bayer Plc) | Rivaroxaban |
| 62150 | 40545021 | Rivaroxaban 2.5mg tablets | Rivaroxaban |
| 47353 | 756021 | Rivaroxaban 15mg tablets | Rivaroxaban |
| 48134 | 758021 | Xarelto 15mg tablets (Bayer Plc) | Rivaroxaban |
| 47925 | 762021 | Xarelto 20mg tablets (Bayer Plc) | Rivaroxaban |
| 47207 | 760021 | Rivaroxaban 20mg tablets | Rivaroxaban |
| 17592 | 87578020 | Innohep 4,500units/0.45ml solution for injection pre-filled syringes (LEO Pharma) | Tinzaparin sodium |
| 63169 | 44859021 | Innohep 12,000units/0.6ml solution for injection pre-filled syringes (LEO Pharma) | Tinzaparin sodium |
| 14851 | 87568020 | Tinzaparin sodium 4,500units/0.45ml solution for injection pre-filled syringes | Tinzaparin sodium |
| 17049 | 87596020 | Innohep 18,000units/0.9ml solution for injection pre-filled syringes (LEO Pharma) | Tinzaparin sodium |
| 32511 | 81243020 | Tinzaparin 5000 i.u./5ml Sterile solution | Tinzaparin Sodium |
| 14308 | 87588020 | Tinzaparin sodium 18,000units/0.9ml solution for injection pre-filled syringes | Tinzaparin sodium |
| 64581 | 44863021 | Innohep 8,000units/0.4ml solution for injection pre-filled syringes (LEO Pharma) | Tinzaparin sodium |
| 17004 | 87564020 | Tinzaparin sodium 20,000units/2ml solution for injection vials | Tinzaparin sodium |
| 62856 | 44858021 | Tinzaparin sodium 12,000units/0.6ml solution for injection pre-filled syringes | Tinzaparin sodium |
| 29207 | 57360020 | Innohep 5000iu/0.5ml Sterile solution (LEO Pharma) | Tinzaparin Sodium |
| 14110 | 87584020 | Tinzaparin sodium 10,000units/0.5ml solution for injection pre-filled syringes | Tinzaparin sodium |
| 28506 | 57494020 | Heparin low molecular weight 3500 iu/0.3ml Sterile solution | Tinzaparin Sodium |
| 19989 | 87582020 | Tinzaparin sodium 40,000units/2ml solution for injection vials | Tinzaparin sodium |
| 13663 | 75893020 | Innohep 20000 iu/ml Injection (LEO Pharma) | Tinzaparin Sodium |
| 63571 | 44861021 | Innohep 16,000units/0.8ml solution for injection pre-filled syringes (LEO Pharma) | Tinzaparin sodium |
| 21233 | 87572020 | Innohep 20,000units/2ml solution for injection vials (LEO Pharma) | Tinzaparin sodium |
| 27325 | 87580020 | Innohep 2,500units/0.25ml solution for injection pre-filled syringes (LEO Pharma) | Tinzaparin sodium |
| 21316 | 87590020 | Innohep 40,000units/2ml solution for injection vials (LEO Pharma) | Tinzaparin sodium |
| 9640 | 52033020 | Tinzaparin 10000 IU/ml Injection | Tinzaparin Sodium |
| 17484 | 57361020 | Innohep 10,000 IU/ml Injection (LEO Pharma) | Tinzaparin Sodium |
| 10240 | 87586020 | Tinzaparin sodium 14,000units/0.7ml solution for injection pre-filled syringes | Tinzaparin sodium |
| 14788 | 87592020 | Innohep 10,000units/0.5ml solution for injection pre-filled syringes (LEO Pharma) | Tinzaparin sodium |
| 17791 | 81245020 | Innohep 5000iu/5ml Sterile solution (LEO Pharma) | Tinzaparin Sodium |
| 17007 | 87570020 | Tinzaparin sodium 2,500units/0.25ml solution for injection pre-filled syringes | Tinzaparin sodium |
| 15709 | 57467020 | Tinzaparin 3500 IU/0.3ml Sterile solution | Tinzaparin Sodium |
| 29317 | 57468020 | Tinzaparin 5000 i.u./0.5ml Sterile solution | Tinzaparin Sodium |
| 19280 | 87594020 | Innohep 14,000units/0.7ml solution for injection pre-filled syringes (LEO Pharma) | Tinzaparin sodium |
| 16061 | 87576020 | Innohep 3,500units/0.35ml solution for injection pre-filled syringes (LEO Pharma) | Tinzaparin sodium |
| 63101 | 44862021 | Tinzaparin sodium 8,000units/0.4ml solution for injection pre-filled syringes | Tinzaparin sodium |
| 9610 | 79398020 | Tinzaparin 20000 iu/ml Injection | Tinzaparin Sodium |
| 62902 | 44860021 | Tinzaparin sodium 16,000units/0.8ml solution for injection pre-filled syringes | Tinzaparin sodium |
| 18732 | 57359020 | Innohep 3500 iu/0.3ml Sterile solution (LEO Pharma) | Tinzaparin Sodium |
| 14212 | 87566020 | Tinzaparin sodium 3,500units/0.35ml solution for injection pre-filled syringes | Tinzaparin sodium |
| 62310 | 41332020 | Warfarin 500microgram tablets (AMCo) | Warfarin sodium |
| 31937 | 55826020 | Warfarin 5mg tablets (Teva UK Ltd) | Warfarin sodium |
| 55316 | 20945020 | Warfarin 3mg/5ml oral suspension | Warfarin sodium |
| 66570 | 47181020 | Warfarin 1mg tablets (Waymade Healthcare Plc) | Warfarin sodium |
| 44866 | 99253020 | Warfarin sodium 1mg/ml oral supension SF | Warfarin Sodium |
| 34417 | 55825020 | Warfarin 3mg tablets (Teva UK Ltd) | Warfarin sodium |
| 38041 | 94827020 | Warfarin sodium 5mg/ml oral suspension | Warfarin Sodium |
| 34418 | 62805020 | Warfarin 5mg tablets (Mylan) | Warfarin sodium |
| 68795 | 69461021 | Warfarin 1mg capsules | Warfarin sodium |
| 1781 | 65439020 | Warfarin 5mg tablets | Warfarin sodium |
| 43655 | 91752020 | Warfarin sodium oral solution | Warfarin Sodium |
| 34864 | 55897020 | Warfarin 5mg tablets (IVAX Pharmaceuticals UK Ltd) | Warfarin sodium |
| 8466 | 54866020 | Marevan 1mg tablets (AMCo) | Warfarin sodium |
| 36099 | 93666020 | Warfarin 1mg/5ml oral suspension | Warfarin sodium |
| 13348 | 54868020 | Marevan 5mg tablets (AMCo) | Warfarin sodium |
| 30202 | 52500020 | Warfarin wbp 1mg Tablet (Boehringer Ingelheim Ltd) | Warfarin sodium |
| 58519 | 17195021 | Warfarin 1mg tablets (DE Pharmaceuticals) | Warfarin sodium |
| 48070 | 96752020 | Warfarin sodium tablets | Warfarin Sodium |
| 59400 | 2311020 | Warfarin 500microgram tablets (Sigma Pharmaceuticals Plc) | Warfarin sodium |
| 69128 | 33709020 | Warfarin 500micrograms/5ml oral solution | Warfarin sodium |
| 54946 | 57162020 | Warfarin 3mg tablets (Actavis UK Ltd) | Warfarin sodium |
| 34299 | 55824020 | Warfarin 1mg tablets (Teva UK Ltd) | Warfarin sodium |
| 38044 | 94825020 | Warfarin 5mg/5ml oral solution | Warfarin sodium |
| 56314 | 63429020 | Warfarin 3mg tablets (Kent Pharmaceuticals Ltd) | Warfarin sodium |
| 47944 | 57161020 | Warfarin 1mg tablets (Actavis UK Ltd) | Warfarin sodium |
| 50000 | 41649020 | Warfarin 1mg/ml oral suspension sugar free (A A H Pharmaceuticals Ltd) | Warfarin sodium |
| 45 | 65437020 | Warfarin 1mg tablets | Warfarin sodium |
| 34019 | 55895020 | Warfarin 1mg tablets (IVAX Pharmaceuticals UK Ltd) | Warfarin sodium |
| 68667 | 69465021 | Warfarin 5mg capsules | Warfarin sodium |
| 48869 | 37790020 | Warfarin 1mg/ml oral suspension sugar free | Warfarin sodium |
| 23078 | 51984020 | Warfarin 1mg Tablet (WB Pharmaceuticals Ltd) | Warfarin sodium |
| 8467 | 54867020 | Marevan 3mg tablets (AMCo) | Warfarin sodium |
| 34576 | 59540020 | Warfarin 1mg Tablet (Lagap) | Warfarin sodium |
| 54892 | 37791020 | Warfarin 1mg/ml oral suspension sugar free (Alliance Healthcare (Distribution) Ltd) | Warfarin sodium |
| 61 | 65438020 | Warfarin 3mg tablets | Warfarin sodium |
| 65285 | 47991021 | Warfarin 1mg tablets (Crescent Pharma Ltd) | Warfarin sodium |
| 6262 | 84736020 | Warfarin 500microgram tablets | Warfarin sodium |
| 60949 | 20948020 | Warfarin 5mg/5ml oral suspension | Warfarin sodium |
| 63071 | 9950020 | Warfarin 4mg tablets | Warfarin sodium |
| 62309 | 2309020 | Warfarin 500microgram tablets (Kent Pharmaceuticals Ltd) | Warfarin sodium |
| 51484 | 2298020 | Warfarin 1mg tablets (Bristol Laboratories Ltd) | Warfarin sodium |
| 31511 | 51985020 | Warfarin 3mg Tablet (WB Pharmaceuticals Ltd) | Warfarin sodium |
| 34517 | 62803020 | Warfarin 1mg tablets (Mylan) | Warfarin sodium |
| 43409 | 56202020 | Warfarin 5mg tablets (A A H Pharmaceuticals Ltd) | Warfarin sodium |
| 51509 | 38963020 | Warfarin 1mg tablets (APC Pharmaceuticals & Chemicals (Europe) Ltd) | Warfarin sodium |
| 34416 | 63426020 | Warfarin 1mg tablets (Kent Pharmaceuticals Ltd) | Warfarin sodium |
| 33711 | 51986020 | Warfarin 5mg Tablet (WB Pharmaceuticals Ltd) | Warfarin sodium |
| 34086 | 50811020 | Warfarin 3mg Tablet (Celltech Pharma Europe Ltd) | Warfarin sodium |
| 60589 | 72795020 | Warfarin 500microgram tablets (Actavis UK Ltd) | Warfarin sodium |
| 40143 | 65390020 | Warfarin 500microgram tablets (A A H Pharmaceuticals Ltd) | Warfarin sodium |
| 65746 | 17198021 | Warfarin 500microgram tablets (DE Pharmaceuticals) | Warfarin sodium |
| 58962 | 17196021 | Warfarin 3mg tablets (DE Pharmaceuticals) | Warfarin sodium |
| 34088 | 50812020 | Warfarin 5mg Tablet (Celltech Pharma Europe Ltd) | Warfarin sodium |
| 17965 | 77096020 | Marevan 500microgram tablets (AMCo) | Warfarin sodium |
| 43408 | 56200020 | Warfarin 1mg tablets (A A H Pharmaceuticals Ltd) | Warfarin sodium |
| 833 | 89966020 | Warfarin 3mg/5ml oral solution | Warfarin sodium |
| 68591 | 2308020 | Warfarin 500microgram tablets (Alliance Healthcare (Distribution) Ltd) | Warfarin sodium |
| 34087 | 50810020 | Warfarin 1mg Tablet (Celltech Pharma Europe Ltd) | Warfarin sodium |
| 59578 | 2303020 | Warfarin 3mg tablets (Phoenix Healthcare Distribution Ltd) | Warfarin sodium |
| 51496 | 2299020 | Warfarin 1mg tablets (Phoenix Healthcare Distribution Ltd) | Warfarin sodium |
| 57032 | 77754020 | Warfarin 1mg/ml oral suspension sugar free (Rosemont Pharmaceuticals Ltd) | Warfarin sodium |
| 65496 | 2312020 | Warfarin 500microgram tablets (Phoenix Healthcare Distribution Ltd) | Warfarin sodium |
| 34691 | 62856020 | Warfarin 5mg Tablet (Regent Laboratories Ltd) | Warfarin sodium |
| 53752 | 2297020 | Warfarin 1mg tablets (Alliance Healthcare (Distribution) Ltd) | Warfarin sodium |
| 34095 | 52502020 | Warfarin wbp 5mg Tablet (Boehringer Ingelheim Ltd) | Warfarin sodium |
| 58787 | 2305020 | Warfarin 5mg tablets (Alliance Healthcare (Distribution) Ltd) | Warfarin sodium |
| 66286 | 33701020 | Warfarin 2.5mg/5ml oral solution | Warfarin sodium |
| 34526 | 62804020 | Warfarin 3mg tablets (Mylan) | Warfarin sodium |
| 39866 | 68098020 | Warfarin 1mg tablets (Almus Pharmaceuticals Ltd) | Warfarin sodium |
| 34758 | 55896020 | Warfarin 3mg tablets (IVAX Pharmaceuticals UK Ltd) | Warfarin sodium |
| 34918 | 57163020 | Warfarin 5mg tablets (Actavis UK Ltd) | Warfarin sodium |
| 71196 | 33697020 | Warfarin 1.5mg/5ml oral solution | Warfarin sodium |
| 30203 | 52501020 | Warfarin wbp 3mg Tablet (Boehringer Ingelheim Ltd) | Warfarin sodium |
| 43407 | 56201020 | Warfarin 3mg tablets (A A H Pharmaceuticals Ltd) | Warfarin sodium |
| 53745 | 2302020 | Warfarin 3mg tablets (Bristol Laboratories Ltd) | Warfarin sodium |

**Supplementary Table 15. Code list for anti-inflammatory non-steroids (AINS)**

| **AINS** |  |  |  |
| --- | --- | --- | --- |
| **prod**  **code** | **Gemscript**  **code** | **Product name** | **Drug**  **Substance name** |
| 7458 | 2704007 | DICLOFENAC SODIUM (3ML) 25 MG/ML INJ |  |
| 7774 | 3449007 | DICLOFENAC 75 MG INJ |  |
| 345 | 3721007 | IBUPROFEN S/R 300 MG CAP |  |
| 10519 | 4147007 | CODEINE PHOS/IBUPROFEN SR (20MG/300MG) TAB |  |
| 2391 | 4699007 | NAPROXEN 250 MG CAP |  |
| 215 | 4856007 | IBUPROFEN 200 MG CAP |  |
| 22158 | 4985007 | FENOPROFEN disp 300 MG TAB |  |
| 22410 | 5629007 | TOLMETIN 200 MG TAB |  |
| 316 | 5712007 | PHENYLBUTAZONE 250 MG SUP |  |
| 63612 | 53040020 | Suprol 200mg Capsule (Lorex Synthelabo Ltd) |  |
| 20653 |  | DICLOFENAC SODIUM S/R |  |
| 29523 |  | DICLOFENAC SODIUM (3ML) |  |
| 24687 |  | FENBUFEN |  |
| 20709 |  | MEFENAMIC ACID DISPERSIBLE |  |
| 20704 |  | NAPROXEN SODIUM |  |
| 25092 |  | NAPROXEN |  |
| 28313 |  | NAPROXEN |  |
| 20699 |  | PIROXICAM DISPERSIBLE |  |
| 20663 |  | PIROXICAM |  |
| 20742 |  | PIROXICAM DISPERSIBLE |  |
| 30090 |  | ROFECOXIB |  |
| 36669 |  | ROFECOXIB |  |
| 32362 |  | ROFECOXIB |  |
| 9474 | 81066020 | Preservex 100mg tablets (Almirall Ltd) | Aceclofenac |
| 526 | 81068020 | Aceclofenac 100mg tablets | Aceclofenac |
| 2258 | 53285020 | Emflex 60mg capsules (Merck Serono Ltd) | Acemetacin |
| 344 | 73771020 | Acemetacin 60mg capsules | Acemetacin |
| 395 | 52557020 | Aspirin mixture | Aspirin |
| 20840 | 55429020 | Acetylsalicylic acid mix | Aspirin |
| 66171 | 59825020 | Aspirin 150mg Suppository (Distriphar (UK)) | Aspirin |
| 55579 | 78846020 | Aspirin 300mg tablets (Almus Pharmaceuticals Ltd) | Aspirin |
| 32728 | 73591020 | Askit oral powder sachets (Bayer Plc) | Aspirin/Aloxiprin/Caffeine citrate |
| 24622 | 61334020 | Aspirin 325mg / Caffeine 22mg tablets | Aspirin/Caffeine |
| 12964 | 73332020 | Aspirin 600mg / Caffeine 50mg oral powder sachets sugar free | Aspirin/Caffeine |
| 3309 | 73333020 | Aspirin 325mg / Caffeine 15mg tablets | Aspirin/Caffeine |
| 381 | 73336020 | Anadin Tablet (Wyeth Consumer Healthcare) | Aspirin/Caffeine |
| 16611 | 73337020 | Anadin Tablet (Wyeth Consumer Healthcare) | Aspirin/Caffeine |
| 11951 | 73345020 | Original Phensic Aspirin tablets (Merck Consumer Health Products) | Aspirin/Caffeine |
| 7539 | 73352020 | Beechams Powders oral powder sachets (GlaxoSmithKline Consumer Healthcare) | Aspirin/Caffeine |
| 31001 | 82098020 | Cullens headache powders Sachets (Cullen and Davidson) | Aspirin/Caffeine |
| 25211 | 89845020 | Anadin Original tablets (Pfizer Consumer Healthcare Ltd) | Aspirin/Caffeine |
| 26967 | 79858020 | Alka-Seltzer XS effervescent tablets (Bayer Plc) | Aspirin/Caffeine/Citric acid/Sodium bicarbonate/Paracetamol |
| 52856 | 29961020 | Co-codaprin 8mg/400mg tablets | Aspirin/Codeine phosphate |
| 9044 | 48725020 | Codis 500 dispersible tablets (Reckitt Benckiser Healthcare (UK) Ltd) | Aspirin/Codeine phosphate |
| 12992 | 69049020 | Aspirin 500mg / Codeine 8mg dispersible tablets sugar free | Aspirin/Codeine phosphate |
| 22776 | 68537020 | Aspirin 500mg with Cyclizine 25mg effervescent tablets | Aspirin/Cyclizine Hydrochloride |
| 484 | 49285020 | Equagesic Tablet (Wyeth Pharmaceuticals) | Aspirin/ethoheptazine Citrate/meprobamate |
| 11326 | 64412020 | Meprobamate with ethoheptazine citrate and aspirin Tablet | Aspirin/ethoheptazine Citrate/meprobamate |
| 14517 | 51418020 | Robaxisal forte Tablet (Shire Pharmaceuticals Ltd) | Aspirin/Methocarbamol |
| 29054 | 64485020 | Methocarbamol with aspirin Tablet | Aspirin/Methocarbamol |
| 685 | 56280020 | Aspav dispersible tablets (Actavis UK Ltd) | Aspirin/Papaveretum |
| 18261 | 70309020 | Aspirin 500mg with Papaveretum 7.71mg dispersible tablets | Aspirin/Papaveretum |
| 6226 | 70312020 | Aspirin 500mg / Papaveretum 7.71mg dispersible tablets sugar free | Aspirin/Papaveretum |
| 31499 | 65980020 | Paracetamol with aspirin tablet | Aspirin/Paracetamol |
| 21770 | 65981020 | Paracetamol 200mg with aspirin 300mg dispersible tablet | Aspirin/Paracetamol |
| 23841 | 66025020 | Safapryn Tablet (Pfizer Ltd) | Aspirin/Paracetamol |
| 20650 | 73522020 | Aspirin 300mg / Paracetamol 200mg dispersible tablets sugar free | Aspirin/Paracetamol |
| 22305 | 73531020 | Disprin Extra dispersible tablets (Reckitt Benckiser Healthcare (UK) Ltd) | Aspirin/Paracetamol |
| 54353 | 14651020 | Generic Anadin Extra soluble tablets sugar free | Aspirin/Paracetamol/Caffeine |
| 7520 | 73479020 | Anadin Extra soluble tablets (Pfizer Consumer Healthcare Ltd) | Aspirin/Paracetamol/Caffeine |
| 32154 | 61337020 | Anadin Soluble tablet (Wyeth Consumer Healthcare) | Caffeine/Aspirin |
| 430 | 73331020 | Aspirin 500mg / Caffeine 32mg capsules | Caffeine/Aspirin |
| 33139 | 73482020 | Anadin maximum strength Capsule (Wyeth Consumer Healthcare) | Caffeine/Aspirin |
| 32314 | 72355020 | Aspirin with aloxiprin and caffeine powder | Caffeine/Aspirin/Aloxiprin |
| 34233 | 72356020 | Aspirin with aloxiprin and caffeine capsules | Caffeine/Aspirin/Aloxiprin |
| 32178 | 82242020 | Paracetamol 133mg with aspirin 267mg & caffeine 40mg effervescent tablet | Caffeine/Aspirin/Paracetamol |
| 19255 | 82243020 | Paracetamol 200mg with aspirin 300mg & caffeine 45mg tablet | Caffeine/Aspirin/Paracetamol |
| 27435 | 82244020 | Paracetamol 200mg with aspirin 300mg & caffeine 45mg soluble tablet | Caffeine/Aspirin/Paracetamol |
| 24309 | 83115020 | Paracetamol 120mg with aspirin 165.3mg & caffeine 60mg powder | Caffeine/Aspirin/Paracetamol |
| 30022 | 81796020 | Fynnon calcium aspirin 500mg Soluble tablet (SSL International Plc) | Calcium Carbonate/Aspirin |
| 66757 | 8508020 | Celebrex 200mg capsules (Waymade Healthcare Plc) | Celecoxib |
| 55582 | 8512020 | Celebrex 200mg capsules (Lexon (UK) Ltd) | Celecoxib |
| 62840 | 8517020 | Celebrex 100mg capsules (DE Pharmaceuticals) | Celecoxib |
| 52420 | 8519020 | Celebrex 100mg capsules (Mawdsley-Brooks & Company Ltd) | Celecoxib |
| 50059 | 8520020 | Celebrex 100mg capsules (Necessity Supplies Ltd) | Celecoxib |
| 65016 | 40716021 | Celecoxib 200mg capsules (Alliance Healthcare (Distribution) Ltd) | Celecoxib |
| 66571 | 43103021 | Celecoxib 200mg capsules (Actavis UK Ltd) | Celecoxib |
| 64245 | 43104021 | Celecoxib 200mg capsules (A A H Pharmaceuticals Ltd) | Celecoxib |
| 64935 | 52306021 | Celecoxib 200mg capsules (Sigma Pharmaceuticals Plc) | Celecoxib |
| 474 | 79023020 | Celecoxib 100mg capsules | Celecoxib |
| 5254 | 79024020 | Celecoxib 200mg capsules | Celecoxib |
| 5175 | 81597020 | Celebrex 100mg capsules (Pfizer Ltd) | Celecoxib |
| 5080 | 81598020 | Celebrex 200mg capsules (Pfizer Ltd) | Celecoxib |
| 43616 | 98663020 | Celecoxib 400mg capsules | Celecoxib |
| 33317 | 81954020 | Aspirin with Sodium bicarbonate with Citric acid effervescent tablets | Citric Acid/Sodium Bicarbonate/Aspirin |
| 16184 | 81957020 | Alka-seltzer Tablet (Bayer Plc) | Citric Acid/Sodium Bicarbonate/Aspirin |
| 63683 | 51016020 | Aspirin with codeine Dispersible tablet (Actavis UK Ltd) | Codeine phosphate/Aspirin |
| 46925 | 53767020 | Co-codaprin 8mg/400mg dispersible tablets (Actavis UK Ltd) | Codeine phosphate/Aspirin |
| 63658 | 53770020 | Co-codaprin 400/8 Tablet (Hillcross Pharmaceuticals Ltd) | Codeine Phosphate/Aspirin |
| 2047 | 55578020 | Co-codaprin 8mg with 400mg tablets | Codeine Phosphate/Aspirin |
| 2986 | 55579020 | Co-codaprin 8mg/400mg dispersible tablets | Codeine phosphate/Aspirin |
| 17926 | 63231020 | Aspirin 400mg with Codeine 8mg tablets | Codeine Phosphate/Aspirin |
| 7518 | 63232020 | Aspirin 400mg with Codeine 8mg dispersible tablets | Codeine Phosphate/Aspirin |
| 9432 | 63233020 | Aspirin 500mg / Codeine 8mg soluble tablets | Codeine Phosphate/Aspirin |
| 42218 | 74305020 | Co-codaprin 8mg/400mg dispersible tablets (A A H Pharmaceuticals Ltd) | Codeine phosphate/Aspirin |
| 20127 | 77403020 | Codeine phosphate 8mg with aspirin 400mg with caffeine dispersible tablets | Codeine Phosphate/Aspirin |
| 15779 | 82636020 | Codeine 8mg with aspirin 500mg soluble tablets | Codeine Phosphate/Aspirin |
| 28784 | 82882020 | Codeine phosphate 8mg with aspirin 400mg tablets | Codeine Phosphate/Aspirin |
| 9129 | 65984020 | Paracetamol 250mg with aspirin 250mg & codeine 6.8mg tablet | Codeine Phosphate/Aspirin/Paracetamol |
| 31894 | 66028020 | Safapryn -Co Tablet (Pfizer Ltd) | Codeine Phosphate/Aspirin/Paracetamol |
| 11961 | 66031020 | Veganin Tablet (Pfizer Consumer Healthcare Ltd) | Codeine Phosphate/Aspirin/Paracetamol |
| 24828 | 71656020 | Hypon Tablet (Wellcome Medical Division) | Codeine Phosphate/Caffeine Citrate/Citric Acid/Calcium Carbonate/Aspirin |
| 306 | 69988020 | Aspirin with codeine phosphate and caffeine tablets | Codeine Phosphate/Caffeine/Aspirin |
| 12709 | 77459020 | Ibuprofen and codeine 200mg + 12.5mg Tablet | Codeine Phosphate/Ibuprofen |
| 10325 | 89560020 | Dexibuprofen 300mg tablets | Dexibuprofen |
| 11907 | 89572020 | Dexibuprofen 400mg tablets | Dexibuprofen |
| 21419 | 89574020 | Seractil 300mg tablets (Thornton & Ross Ltd) | Dexibuprofen |
| 21421 | 89582020 | Seractil 400mg tablets (Thornton & Ross Ltd) | Dexibuprofen |
| 5173 | 67445020 | Dexketoprofen 25mg tablets | Dexketoprofen trometamol |
| 9637 | 79359020 | Keral 25mg tablets (A. Menarini Farmaceutica Internazionale SRL) | Dexketoprofen trometamol |
| 21067 | 49085020 | Doloxene Capsule (Eli Lilly and Company Ltd) | Dextropropoxyphene Napsylate/Caffeine/Aspirin |
| 47820 | 164021 | Voltarol Pain-eze Extra Strength 25mg tablets (Novartis Consumer Health UK Ltd) | Diclofenac potassium |
| 58572 | 8370020 | Diclofenac potassium 25mg tablets (A A H Pharmaceuticals Ltd) | Diclofenac potassium |
| 58071 | 8371020 | Voltarol Rapid 50mg tablets (Waymade Healthcare Plc) | Diclofenac potassium |
| 69477 | 8373020 | Voltarol Rapid 50mg tablets (DE Pharmaceuticals) | Diclofenac potassium |
| 70468 | 8374020 | Voltarol Rapid 50mg tablets (Sigma Pharmaceuticals Plc) | Diclofenac potassium |
| 53345 | 8375020 | Voltarol Rapid 50mg tablets (Lexon (UK) Ltd) | Diclofenac potassium |
| 70145 | 8376020 | Voltarol Rapid 50mg tablets (Stephar (U.K.) Ltd) | Diclofenac potassium |
| 51099 | 8377020 | Voltarol Rapid 50mg tablets (Mawdsley-Brooks & Company Ltd) | Diclofenac potassium |
| 52338 | 8378020 | Diclofenac potassium 50mg tablets (Focus Pharmaceuticals Ltd) | Diclofenac potassium |
| 50602 | 8379020 | Diclofenac potassium 50mg tablets (Alliance Healthcare (Distribution) Ltd) | Diclofenac potassium |
| 51293 | 8380020 | Diclofenac potassium 50mg tablets (Phoenix Healthcare Distribution Ltd) | Diclofenac potassium |
| 51343 | 39330020 | Voltarol Rapid 25mg tablets (DE Pharmaceuticals) | Diclofenac potassium |
| 65783 | 53640021 | Diclofenac potassium 50mg tablets (DE Pharmaceuticals) | Diclofenac potassium |
| 48871 | 77023020 | Diclofenac potassium 25mg tablets (Actavis UK Ltd) | Diclofenac potassium |
| 43045 | 77025020 | Diclofenac potassium 50mg tablets (Actavis UK Ltd) | Diclofenac potassium |
| 48059 | 77150020 | Diclofenac potassium 50mg tablets (A A H Pharmaceuticals Ltd) | Diclofenac potassium |
| 628 | 79361020 | Diclofenac potassium 25mg tablets | Diclofenac potassium |
| 597 | 79362020 | Diclofenac potassium 50mg tablets | Diclofenac potassium |
| 5401 | 82738020 | Voltarol Rapid 25mg tablets (Novartis Pharmaceuticals UK Ltd) | Diclofenac potassium |
| 5085 | 82739020 | Voltarol Rapid 50mg tablets (Novartis Pharmaceuticals UK Ltd) | Diclofenac potassium |
| 38817 | 95565020 | Diclofenac potassium 12.5mg tablets | Diclofenac potassium |
| 39722 | 95573020 | Voltarol Pain-eze 12.5mg tablets (Novartis Consumer Health UK Ltd) | Diclofenac potassium |
| 44112 | 98747020 | Voltarol Joint Pain 12.5mg tablets (Novartis Consumer Health UK Ltd) | Diclofenac potassium |
| 45814 | 99265020 | First Resort Double Action Pain Relief 12.5mg tablets (Actavis UK Ltd) | Diclofenac potassium |
| 47501 | 59021 | Rhumalgan SR 75mg capsules (Almus Pharmaceuticals Ltd) | Diclofenac sodium |
| 56078 | 61021 | Rhumalgan XL 100mg capsules (Almus Pharmaceuticals Ltd) | Diclofenac sodium |
| 48218 | 629021 | Dicloflex sr 100mg Tablet (Teva UK Ltd) | Diclofenac sodium |
| 71064 | 8045020 | Diclofenac sodium 75mg modified-release capsules (Waymade Healthcare Plc) | Diclofenac sodium |
| 71117 | 8046020 | Diclomax SR 75mg capsules (DE Pharmaceuticals) | Diclofenac sodium |
| 52389 | 8049020 | Voltarol 50mg suppositories (Sigma Pharmaceuticals Plc) | Diclofenac sodium |
| 69582 | 8061020 | Voltarol 100mg suppositories (Lexon (UK) Ltd) | Diclofenac sodium |
| 54021 | 8069020 | Voltarol Retard 100mg tablets (Sigma Pharmaceuticals Plc) | Diclofenac sodium |
| 57006 | 8075020 | Diclofenac sodium 25mg gastro-resistant tablets (Phoenix Healthcare Distribution Ltd) | Diclofenac sodium |
| 54518 | 8079020 | Diclofenac sodium 50mg gastro-resistant tablets (Phoenix Healthcare Distribution Ltd) | Diclofenac sodium |
| 71100 | 8080020 | Diclofenac sodium 50mg gastro-resistant tablets (Sigma Pharmaceuticals Plc) | Diclofenac sodium |
| 57045 | 8084020 | Voltarol 50mg dispersible tablets (Waymade Healthcare Plc) | Diclofenac sodium |
| 50058 | 8086020 | Voltarol 50mg dispersible tablets (DE Pharmaceuticals) | Diclofenac sodium |
| 59595 | 8087020 | Diclofenac 50mg dispersible tablets sugar free (Sigma Pharmaceuticals Plc) | Diclofenac sodium |
| 60786 | 8088020 | Voltarol 50mg dispersible tablets (Sigma Pharmaceuticals Plc) | Diclofenac sodium |
| 49059 | 8090020 | Voltarol 50mg dispersible tablets (Lexon (UK) Ltd) | Diclofenac sodium |
| 54075 | 8091020 | Voltarol 50mg dispersible tablets (Stephar (U.K.) Ltd) | Diclofenac sodium |
| 53384 | 8092020 | Voltarol 50mg dispersible tablets (Mawdsley-Brooks & Company Ltd) | Diclofenac sodium |
| 57162 | 8093020 | Diclofenac 50mg dispersible tablets sugar free (DE Pharmaceuticals) | Diclofenac sodium |
| 65877 | 8113020 | Diclofenac sodium 75mg modified-release tablets (Mawdsley-Brooks & Company Ltd) | Diclofenac sodium |
| 56898 | 15708021 | Rhumalgan SR 75mg capsules (Actavis UK Ltd) | Diclofenac sodium |
| 59289 | 15710021 | Diclofenac sodium 100mg modified-release tablets (AM Distributions (Yorkshire) Ltd) | Diclofenac sodium |
| 60368 | 19739020 | Diclofenac 10mg/5ml oral solution | Diclofenac sodium |
| 61762 | 19741020 | Diclofenac 10mg/5ml oral suspension | Diclofenac sodium |
| 59880 | 20255021 | Diclofenac sodium 75mg modified-release capsules (Waymade Healthcare Plc) | Diclofenac sodium |
| 61596 | 21425021 | Diclofenac sodium 75mg modified-release capsules (Phoenix Healthcare Distribution Ltd) | Diclofenac sodium |
| 67220 | 21427021 | Diclofenac sodium 100mg modified-release tablets (Phoenix Healthcare Distribution Ltd) | Diclofenac sodium |
| 64303 | 21428021 | Diclofenac sodium 25mg gastro-resistant tablets (DE Pharmaceuticals) | Diclofenac sodium |
| 60443 | 21834021 | Diclofenac sodium 75mg modified-release capsules (DE Pharmaceuticals) | Diclofenac sodium |
| 66123 | 23762021 | Diclofenac sodium 100mg modified-release tablets (Ethigen Ltd) | Diclofenac sodium |
| 60666 | 25523021 | Diclofenac sodium 75mg modified-release capsules (Actavis UK Ltd) | Diclofenac sodium |
| 68354 | 25525021 | Diclofenac sodium 100mg modified-release capsules (Actavis UK Ltd) | Diclofenac sodium |
| 66577 | 26767021 | Diclofenac sodium 75mg modified-release capsules (Sigma Pharmaceuticals Plc) | Diclofenac sodium |
| 69584 | 26768021 | Diclofenac sodium 100mg modified-release tablets (Sigma Pharmaceuticals Plc) | Diclofenac sodium |
| 54906 | 29442020 | Diclofenac 50mg/5ml oral suspension | Diclofenac sodium |
| 51808 | 29485020 | Diclofenac 12.5mg/5ml oral solution | Diclofenac sodium |
| 68849 | 29487020 | Diclofenac 12.5mg/5ml oral suspension | Diclofenac sodium |
| 64759 | 29493020 | Diclofenac 50mg/5ml oral solution | Diclofenac sodium |
| 55913 | 39886020 | Voltarol 50mg suppositories (Lexon (UK) Ltd) | Diclofenac sodium |
| 50317 | 39887020 | Voltarol 75mg SR tablets (Lexon (UK) Ltd) | Diclofenac sodium |
| 50785 | 40590020 | Diclofenac sodium 50mg gastro-resistant tablets (Genesis Pharmaceuticals Ltd) | Diclofenac sodium |
| 58048 | 47557020 | Diclofenac sodium 50mg gastro-resistant tablets (Waymade Healthcare Plc) | Diclofenac sodium |
| 10792 | 49459020 | Voltarol 50mg Suppository (Novartis Pharmaceuticals UK Ltd) | Diclofenac sodium |
| 32536 | 49461020 | Diclofenac 25mg Tablet (Berk Pharmaceuticals Ltd) | Diclofenac sodium |
| 28256 | 49462020 | Diclofenac 50mg Tablet (Berk Pharmaceuticals Ltd) | Diclofenac sodium |
| 612 | 50464020 | Dicloflex 25mg gastro-resistant tablets (Dexcel-Pharma Ltd) | Diclofenac sodium |
| 4692 | 50465020 | Dicloflex 50mg gastro-resistant tablets (Dexcel-Pharma Ltd) | Diclofenac sodium |
| 8789 | 50466020 | Dicloflex retard tabs 100 100mg Modified-release tablet (Dexcel-Pharma Ltd) | Diclofenac sodium |
| 33559 | 51185020 | Diclofenac 50mg Tablet (C P Pharmaceuticals Ltd) | Diclofenac sodium |
| 24128 | 51193020 | Diclofenac sodium 25mg gastro-resistant tablets (A A H Pharmaceuticals Ltd) | Diclofenac sodium |
| 26165 | 51194020 | Diclofenac sodium 50mg gastro-resistant tablets (A A H Pharmaceuticals Ltd) | Diclofenac sodium |
| 31589 | 51195020 | Diclofenac sodium 75mg modified-release tablets (A A H Pharmaceuticals Ltd) | Diclofenac sodium |
| 34091 | 51270020 | Diclofenac sodium 25mg gastro-resistant tablets (Sandoz Ltd) | Diclofenac sodium |
| 29330 | 51271020 | Diclofenac sodium 50mg gastro-resistant tablets (Sandoz Ltd) | Diclofenac sodium |
| 447 | 52256020 | Diclofenac sodium 75mg modified-release capsules | Diclofenac sodium |
| 1139 | 53288020 | Voltarol 25mg Tablet (Novartis Pharmaceuticals UK Ltd) | Diclofenac sodium |
| 1446 | 53289020 | Voltarol 50mg Tablet (Novartis Pharmaceuticals UK Ltd) | Diclofenac sodium |
| 157 | 53290020 | Voltarol 100mg Suppository (Novartis Pharmaceuticals UK Ltd) | Diclofenac sodium |
| 2293 | 53293020 | Voltarol 25mg/ml Injection (Novartis Pharmaceuticals UK Ltd) | Diclofenac sodium |
| 1766 | 53294020 | Voltarol sr 75mg Modified-release tablet (Novartis Pharmaceuticals UK Ltd) | Diclofenac sodium |
| 10978 | 53295020 | Voltarol 25mg Suppository (Novartis Pharmaceuticals UK Ltd) | Diclofenac sodium |
| 2386 | 53298020 | Voltarol Retard 100mg tablets (Novartis Pharmaceuticals UK Ltd) | Diclofenac sodium |
| 4095 | 53301020 | Voltarol 12.5mg Suppository (Novartis Pharmaceuticals UK Ltd) | Diclofenac sodium |
| 33994 | 53791020 | Diclofenac sodium 25mg gastro-resistant tablets (IVAX Pharmaceuticals UK Ltd) | Diclofenac sodium |
| 34487 | 53792020 | Diclofenac sodium 50mg gastro-resistant tablets (IVAX Pharmaceuticals UK Ltd) | Diclofenac sodium |
| 33645 | 53793020 | Diclofenac 75mg Modified-release tablet (IVAX Pharmaceuticals UK Ltd) | Diclofenac sodium |
| 54463 | 54642020 | Diclofenac 50mg Tablet (Approved Prescription Services Ltd) | Diclofenac sodium |
| 17126 | 55714020 | Fenactol SR 75mg tablets (Discovery Pharmaceuticals) | Diclofenac sodium |
| 27677 | 56735020 | Diclofenac 75mg/3ml Injection (Antigen Pharmaceuticals) | Diclofenac sodium |
| 24121 | 56925020 | Diclofenac sodium 25mg gastro-resistant tablets (Actavis UK Ltd) | Diclofenac sodium |
| 24122 | 56926020 | Diclofenac sodium 50mg gastro-resistant tablets (Actavis UK Ltd) | Diclofenac sodium |
| 27362 | 56927020 | Diclofenac 100mg Modified-release tablet (Actavis UK Ltd) | Diclofenac sodium |
| 16221 | 57190020 | Diclozip 25mg gastro-resistant tablets (Ashbourne Pharmaceuticals Ltd) | Diclofenac sodium |
| 16222 | 57191020 | Diclozip 50mg gastro-resistant tablets (Ashbourne Pharmaceuticals Ltd) | Diclofenac sodium |
| 25362 | 57275020 | Defanac 25mg gastro-resistant tablets (Ranbaxy (UK) Ltd) | Diclofenac sodium |
| 30297 | 59231020 | Diclofenac 50mg Gastro-resistant tablet (Pharmacia Ltd) | Diclofenac sodium |
| 34218 | 59232020 | Diclofenac 25mg Gastro-resistant tablet (Pharmacia Ltd) | Diclofenac sodium |
| 30282 | 59264020 | Diclofenac 75mg Modified-release tablet (Galen Ltd) | Diclofenac sodium |
| 42905 | 59298020 | Diclofenac 75mg Modified-release tablet (Actavis UK Ltd) | Diclofenac sodium |
| 30942 | 59698020 | Diclofenac 50mg Tablet (Regent Laboratories Ltd) | Diclofenac sodium |
| 32108 | 59804020 | Diclofenac sodium 25mg gastro-resistant tablets (Teva UK Ltd) | Diclofenac sodium |
| 28553 | 59805020 | Diclofenac sodium 50mg gastro-resistant tablets (Teva UK Ltd) | Diclofenac sodium |
| 62636 | 60043020 | Diclofenac sodium 25mg gastro-resistant tablets (Sterwin Medicines) | Diclofenac sodium |
| 31950 | 60044020 | Diclofenac sodium 50mg gastro-resistant tablets (Sterwin Medicines) | Diclofenac sodium |
| 31944 | 60370020 | Diclofenac sodium 25mg gastro-resistant tablets (Mylan) | Diclofenac sodium |
| 21387 | 60371020 | Diclofenac sodium 50mg gastro-resistant tablets (Mylan) | Diclofenac sodium |
| 34271 | 60405020 | Diclofenac sodium 100mg modified-release tablets (A A H Pharmaceuticals Ltd) | Diclofenac sodium |
| 42793 | 61462020 | Diclofenac 100mg Modified-release tablet (IVAX Pharmaceuticals UK Ltd) | Diclofenac sodium |
| 34362 | 61922020 | Diclofenac 25mg Gastro-resistant tablet (Genus Pharmaceuticals Ltd) | Diclofenac sodium |
| 33669 | 61923020 | Diclofenac 50mg Gastro-resistant tablet (Genus Pharmaceuticals Ltd) | Diclofenac sodium |
| 34212 | 61924020 | Diclofenac 75mg Modified-release tablet (Genus Pharmaceuticals Ltd) | Diclofenac sodium |
| 928 | 62455020 | Diclofenac sodium 25mg tablets | Diclofenac Sodium |
| 917 | 62456020 | Diclofenac sodium 50mg tablets | Diclofenac Sodium |
| 1984 | 62457020 | Diclofenac sodium 100mg modified-release tablets | Diclofenac Sodium |
| 1116 | 62460020 | Diclofenac 100mg suppositories | Diclofenac sodium |
| 7667 | 62461020 | Diclofenac 12.5mg suppositories | Diclofenac sodium |
| 1233 | 62462020 | Diclofenac sodium 75mg modified-release tablets | Diclofenac Sodium |
| 27055 | 63521020 | Diclofenac sodium 50mg gastro-resistant tablets (Kent Pharmaceuticals Ltd) | Diclofenac sodium |
| 30923 | 64059020 | Diclofenac 100mg suppositories (A A H Pharmaceuticals Ltd) | Diclofenac sodium |
| 53164 | 68114020 | Diclofenac sodium 25mg gastro-resistant tablets (Kent Pharmaceuticals Ltd) | Diclofenac sodium |
| 33457 | 68415020 | Isclofen 50mg Gastro-resistant tablet (Isis Products Ltd) | Diclofenac sodium |
| 25790 | 68882020 | Rhumalgan 25mg Tablet (Lagap) | Diclofenac sodium |
| 30806 | 68883020 | Rhumalgan 50mg Tablet (Lagap) | Diclofenac sodium |
| 32916 | 68901020 | Diclofenac 75mg Modified-release capsule (Sandoz Ltd) | Diclofenac sodium |
| 34744 | 68904020 | Diclofenac 100mg Modified-release capsule (Sandoz Ltd) | Diclofenac sodium |
| 32854 | 69320020 | Diclofenac sodium 75mg modified-release capsules (A A H Pharmaceuticals Ltd) | Diclofenac sodium |
| 676 | 72315020 | Diclofenac 75mg/3ml solution for injection ampoules | Diclofenac sodium |
| 589 | 72769020 | Voltarol 50mg dispersible tablets (Novartis Pharmaceuticals UK Ltd) | Diclofenac sodium |
| 417 | 72775020 | Diclofenac 50mg dispersible tablets sugar free | Diclofenac sodium |
| 42406 | 73414020 | Diclofenac 50mg Gastro-resistant tablet (Almus Pharmaceuticals Ltd) | Diclofenac sodium |
| 1096 | 73892020 | Diclofenac sodium 25mg gastro-resistant tablets | Diclofenac Sodium |
| 1075 | 73893020 | Diclofenac sodium 50mg gastro-resistant tablets | Diclofenac Sodium |
| 1115 | 73894020 | Diclofenac sodium 100mg modified-release capsules | Diclofenac sodium |
| 28390 | 73897020 | Valenac ec 25mg Gastro-resistant tablet (Shire Pharmaceuticals Ltd) | Diclofenac sodium |
| 25283 | 73898020 | Valenac ec 50mg Gastro-resistant tablet (Shire Pharmaceuticals Ltd) | Diclofenac sodium |
| 21444 | 74266020 | Volraman 25mg gastro-resistant tablets (LPC Medical (UK) Ltd) | Diclofenac sodium |
| 15201 | 74267020 | Volraman 50mg gastro-resistant tablets (LPC Medical (UK) Ltd) | Diclofenac sodium |
| 3852 | 74400020 | Diclomax 100mg Modified-release capsule (Provalis Healthcare Ltd) | Diclofenac sodium |
| 3421 | 74401020 | Diclomax sr 75mg Modified-release capsule (Provalis Healthcare Ltd) | Diclofenac sodium |
| 21807 | 74801020 | Flamrase 25 EC tablets (Teva UK Ltd) | Diclofenac sodium |
| 21824 | 74802020 | Flamrase 50 EC tablets (Teva UK Ltd) | Diclofenac sodium |
| 10917 | 74803020 | Flamrase SR 100mg tablets (Teva UK Ltd) | Diclofenac sodium |
| 8062 | 75820020 | Motifene 75mg modified-release capsules (Daiichi Sankyo UK Ltd) | Diclofenac sodium |
| 2904 | 75877020 | Diclofenac sodium 75mg gastro-resistant modified-release capsules | Diclofenac sodium |
| 14672 | 75883020 | Defanac 75mg SR tablets (Ranbaxy (UK) Ltd) | Diclofenac sodium |
| 14678 | 75884020 | Defanac sr 100mg Modified-release tablet (Ranbaxy (UK) Ltd) | Diclofenac sodium |
| 3958 | 76044020 | Diclofenac 25mg suppositories | Diclofenac sodium |
| 499 | 76045020 | Diclofenac 50mg suppositories | Diclofenac sodium |
| 17491 | 76698020 | Dicloflex sr 75mg Tablet (Ratiopharm UK Ltd) | Diclofenac sodium |
| 17128 | 77273020 | Fenactol 50mg gastro-resistant tablets (Discovery Pharmaceuticals) | Diclofenac sodium |
| 18921 | 77276020 | Fenactol 25mg gastro-resistant tablets (Discovery Pharmaceuticals) | Diclofenac sodium |
| 25358 | 78548020 | Defanac 50mg gastro-resistant tablets (Ranbaxy (UK) Ltd) | Diclofenac sodium |
| 17525 | 78919020 | Fenactol Retard 100mg tablets (Discovery Pharmaceuticals) | Diclofenac sodium |
| 9222 | 79017020 | Dicloflex 75mg SR tablets (Dexcel-Pharma Ltd) | Diclofenac sodium |
| 14707 | 79162020 | Defanac Retard 100mg tablets (Ranbaxy (UK) Ltd) | Diclofenac sodium |
| 32601 | 79375020 | Econac 100mg suppositories (AMCo) | Diclofenac sodium |
| 25329 | 79838020 | Lofensaid 25mg gastro-resistant tablets (Opus Pharmaceuticals Ltd) | Diclofenac sodium |
| 18798 | 79839020 | Lofensaid 50mg gastro-resistant tablets (Opus Pharmaceuticals Ltd) | Diclofenac sodium |
| 17029 | 80210020 | Rhumalgan CR 75 tablets (Sandoz Ltd) | Diclofenac sodium |
| 21610 | 80211020 | Rhumalgan CR 100 tablets (Sandoz Ltd) | Diclofenac sodium |
| 4506 | 80722020 | Volsaid Retard 75 tablets (Chiesi Ltd) | Diclofenac sodium |
| 11168 | 80723020 | Volsaid Retard 100 tablets (Chiesi Ltd) | Diclofenac sodium |
| 30849 | 81084020 | Valdic 75 Retard tablets (Fannin UK Ltd) | Diclofenac sodium |
| 29037 | 81085020 | Valdic 100 Retard tablets (Fannin UK Ltd) | Diclofenac sodium |
| 20105 | 81306020 | Dicloflex 25mg Gastro-resistant tablet (Ratiopharm UK Ltd) | Diclofenac sodium |
| 9886 | 82072020 | Dicloflex 50mg Gastro-resistant tablet (Ratiopharm UK Ltd) | Diclofenac sodium |
| 16286 | 82237020 | Lofensaid Retard 75 tablets (Opus Pharmaceuticals Ltd) | Diclofenac sodium |
| 16272 | 82238020 | Lofensaid Retard 100 tablets (Opus Pharmaceuticals Ltd) | Diclofenac sodium |
| 26351 | 83497020 | Rheumatac Retard 75 tablets (AMCo) | Diclofenac sodium |
| 18371 | 83511020 | Digenac xl 100mg Modified-release tablet (Genus Pharmaceuticals Ltd) | Diclofenac sodium |
| 497 | 83709020 | Voltarol 25mg gastro-resistant tablets (Novartis Pharmaceuticals UK Ltd) | Diclofenac sodium |
| 4631 | 83710020 | Voltarol 50mg gastro-resistant tablets (Novartis Pharmaceuticals UK Ltd) | Diclofenac sodium |
| 18448 | 83740020 | Voltarol 12.5mg suppositories (Novartis Pharmaceuticals UK Ltd) | Diclofenac sodium |
| 11215 | 83741020 | Voltarol 25mg suppositories (Novartis Pharmaceuticals UK Ltd) | Diclofenac sodium |
| 5200 | 83742020 | Voltarol 50mg suppositories (Novartis Pharmaceuticals UK Ltd) | Diclofenac sodium |
| 4806 | 83744020 | Voltarol 100mg suppositories (Novartis Pharmaceuticals UK Ltd) | Diclofenac sodium |
| 4713 | 83756020 | Voltarol 75mg/3ml solution for injection ampoules (Novartis Pharmaceuticals UK Ltd) | Diclofenac sodium |
| 11322 | 83762020 | Flamrase sr 75mg Modified-release tablet (APS Berk) | Diclofenac sodium |
| 4625 | 83774020 | Voltarol 75mg SR tablets (Novartis Pharmaceuticals UK Ltd) | Diclofenac sodium |
| 580 | 83822020 | Diclofenac sodium 75mg modified-release tablets | Diclofenac sodium |
| 3416 | 83823020 | Diclofenac sodium 100mg modified-release tablets | Diclofenac sodium |
| 649 | 83871020 | Diclofenac sodium 25mg gastro-resistant tablets | Diclofenac sodium |
| 40 | 83872020 | Diclofenac sodium 50mg gastro-resistant tablets | Diclofenac sodium |
| 19382 | 83888020 | Slofenac 75mg SR tablets (Sterwin Medicines) | Diclofenac sodium |
| 24236 | 83889020 | Slofenac 100mg Modified-release tablet (Sterwin Medicines) | Diclofenac sodium |
| 9500 | 84230020 | Diclotard 75mg modified-release tablets (Galen Ltd) | Diclofenac sodium |
| 9465 | 84244020 | Diclotard 100 100mg Modified-release tablet (Galen Ltd) | Diclofenac sodium |
| 29455 | 84251020 | Flexotard MR 100mg tablets (Pfizer Ltd) | Diclofenac sodium |
| 16225 | 84262020 | Dexomon retard 100mg Modified-release tablet (Hillcross Pharmaceuticals Ltd) | Diclofenac sodium |
| 30790 | 84507020 | Dicloflex sr 75mg Tablet (Genus Pharmaceuticals Ltd) | Diclofenac sodium |
| 20621 | 84866020 | Dicloflex 75mg SR tablets (Kent Pharmaceuticals Ltd) | Diclofenac sodium |
| 17532 | 84868020 | Dicloflex Retard 100mg tablets (Kent Pharmaceuticals Ltd) | Diclofenac sodium |
| 28764 | 84951020 | Closteril 100mg Modified-release tablet (Pharmalife Healthcare Services Ltd) | Diclofenac sodium |
| 20384 | 85672020 | Flamatak MR 100mg tablets (Actavis UK Ltd) | Diclofenac sodium |
| 20395 | 85673020 | Flamatak MR 75mg tablets (Actavis UK Ltd) | Diclofenac sodium |
| 26888 | 85826020 | Difenor xl 100mg Modified-release tablet (IVAX Pharmaceuticals UK Ltd) | Diclofenac sodium |
| 20805 | 85934020 | Dicloflex 75mg SR tablets (Teva UK Ltd) | Diclofenac sodium |
| 17124 | 85935020 | Dicloflex sr 100mg Tablet (IVAX Pharmaceuticals UK Ltd) | Diclofenac sodium |
| 31383 | 86166020 | Dexomon 75mg SR tablets (Hillcross Pharmaceuticals Ltd) | Diclofenac sodium |
| 25361 | 86198020 | Diclovol 25mg gastro-resistant tablets (Arun Pharmaceuticals Ltd) | Diclofenac sodium |
| 15732 | 86199020 | Diclovol 50mg gastro-resistant tablets (Arun Pharmaceuticals Ltd) | Diclofenac sodium |
| 14084 | 86201020 | Diclovol 75mg SR tablets (Arun Pharmaceuticals Ltd) | Diclofenac sodium |
| 14085 | 86203020 | Diclovol Retard 100mg tablets (Arun Pharmaceuticals Ltd) | Diclofenac sodium |
| 40086 | 86647020 | Acoflam 50mg gastro-resistant tablets (Mercury Pharma Group Ltd) | Diclofenac sodium |
| 55099 | 86649020 | Acoflam 100mg Retard tablets (Mercury Pharma Group Ltd) | Diclofenac sodium |
| 9688 | 86796020 | Diclovol 75mg SR tablets (Mylan) | Diclofenac sodium |
| 27200 | 86798020 | Diclovol Retard 100mg tablets (Mylan) | Diclofenac sodium |
| 31787 | 87660020 | Econac SR 75mg tablets (AMCo) | Diclofenac sodium |
| 36486 | 87664020 | Econac XL 100mg tablets (AMCo) | Diclofenac sodium |
| 17030 | 88558020 | Rhumalgan SR 75mg capsules (Sandoz Ltd) | Diclofenac sodium |
| 26631 | 88560020 | Rhumalgan XL 100mg capsules (Sandoz Ltd) | Diclofenac sodium |
| 35893 | 91115020 | Dicloflex Retard 100mg tablets (Almus Pharmaceuticals Ltd) | Diclofenac sodium |
| 29181 | 91117020 | Dicloflex 75mg SR tablets (Almus Pharmaceuticals Ltd) | Diclofenac sodium |
| 54660 | 92313020 | Diclofenac sodium 50mg capsules | Diclofenac Sodium |
| 35711 | 92493020 | Dicloflex 25mg gastro-resistant tablets (Teva UK Ltd) | Diclofenac sodium |
| 45213 | 92541020 | Diclofenac 10mg dispersible tablets | Diclofenac sodium |
| 37763 | 94477020 | Diclofenac 75mg/2ml solution for injection vials | Diclofenac sodium |
| 38881 | 95549020 | Diclomax SR 75mg capsules (Galen Ltd) | Diclofenac sodium |
| 38992 | 95555020 | Flamrase 75mg SR tablets (Teva UK Ltd) | Diclofenac sodium |
| 39264 | 95833020 | Dicloflex Retard 100mg tablets (Dexcel-Pharma Ltd) | Diclofenac sodium |
| 38948 | 95891020 | Diclomax Retard 100mg capsules (Galen Ltd) | Diclofenac sodium |
| 40756 | 96608020 | Dicloflex 25mg gastro-resistant tablets (Almus Pharmaceuticals Ltd) | Diclofenac sodium |
| 39823 | 96610020 | Dicloflex 50mg gastro-resistant tablets (Almus Pharmaceuticals Ltd) | Diclofenac sodium |
| 42455 | 97700020 | Dicloflex Retard 100mg tablets (Teva UK Ltd) | Diclofenac sodium |
| 46844 | 99460020 | Dicloflex 75mg SR tablets (Actavis UK Ltd) | Diclofenac sodium |
| 2363 | 49076020 | Dolobid 250mg tablets (Merck Sharp & Dohme Ltd) | Diflunisal |
| 3899 | 49077020 | Dolobid 500mg tablets (Merck Sharp & Dohme Ltd) | Diflunisal |
| 560 | 62515020 | Diflunisal 250mg tablets | Diflunisal |
| 3492 | 62516020 | Diflunisal 500mg tablets | Diflunisal |
| 52714 | 8117020 | Etodolac 600mg modified-release tablets (Alliance Healthcare (Distribution) Ltd) | Etodolac |
| 4368 | 58036020 | Lodine 200mg Capsule (Shire Pharmaceuticals Ltd) | Etodolac |
| 16194 | 58037020 | Lodine 200mg Tablet (Shire Pharmaceuticals Ltd) | Etodolac |
| 8969 | 58038020 | Lodine 300mg Capsule (Shire Pharmaceuticals Ltd) | Etodolac |
| 3311 | 60932020 | Etodolac 200mg capsules | Etodolac |
| 8451 | 60933020 | Etodolac 200mg Tablet | Etodolac |
| 10033 | 60934020 | Etodolac 300mg capsules | Etodolac |
| 20386 | 60937020 | Ramodar 200mg Tablet (Wyeth Pharmaceuticals) | Etodolac |
| 5266 | 75111020 | Lodine sr 600mg Modified-release tablet (Shire Pharmaceuticals Ltd) | Etodolac |
| 5455 | 75114020 | Etodolac 600mg modified-release tablets | Etodolac |
| 24356 | 83390020 | Eccoxolac 300mg capsules (Meda Pharmaceuticals Ltd) | Etodolac |
| 66323 | 86577020 | Ebretin 300mg capsules (Ranbaxy (UK) Ltd) | Etodolac |
| 35653 | 91953020 | Etopan XL 600mg tablets (Sun Pharmaceuticals UK Ltd) | Etodolac |
| 38770 | 95557020 | Lodine SR 600mg tablets (Almirall Ltd) | Etodolac |
| 51284 | 10595020 | Arcoxia 60mg tablets (Sigma Pharmaceuticals Plc) | Etoricoxib |
| 56584 | 10596020 | Arcoxia 60mg tablets (Lexon (UK) Ltd) | Etoricoxib |
| 66486 | 10601020 | Arcoxia 90mg tablets (DE Pharmaceuticals) | Etoricoxib |
| 62251 | 10602020 | Arcoxia 90mg tablets (Sigma Pharmaceuticals Plc) | Etoricoxib |
| 62843 | 10603020 | Arcoxia 90mg tablets (Lexon (UK) Ltd) | Etoricoxib |
| 64521 | 10604020 | Arcoxia 90mg tablets (Mawdsley-Brooks & Company Ltd) | Etoricoxib |
| 62658 | 10606020 | Arcoxia 120mg tablets (Waymade Healthcare Plc) | Etoricoxib |
| 53576 | 10607020 | Arcoxia 120mg tablets (DE Pharmaceuticals) | Etoricoxib |
| 51874 | 39400020 | Arcoxia 30mg tablets (Lexon (UK) Ltd) | Etoricoxib |
| 6498 | 77845020 | Arcoxia 90mg tablets (Grunenthal Ltd) | Etoricoxib |
| 9822 | 77848020 | Arcoxia 120mg tablets (Grunenthal Ltd) | Etoricoxib |
| 650 | 77851020 | Etoricoxib 60mg tablets | Etoricoxib |
| 5812 | 77854020 | Etoricoxib 90mg tablets | Etoricoxib |
| 5938 | 84667020 | Etoricoxib 120mg tablets | Etoricoxib |
| 6464 | 84678020 | Arcoxia 60mg tablets (Grunenthal Ltd) | Etoricoxib |
| 37587 | 94522020 | Etoricoxib 30mg tablets | Etoricoxib |
| 37562 | 94524020 | Arcoxia 30mg tablets (Grunenthal Ltd) | Etoricoxib |
| 7522 | 50134020 | Lederfen 300mg Tablet (Wyeth Pharmaceuticals) | Fenbufen |
| 7426 | 50135020 | Lederfen 300mg Capsule (Wyeth Pharmaceuticals) | Fenbufen |
| 7481 | 50136020 | Lederfen 450mg Tablet (Wyeth Pharmaceuticals) | Fenbufen |
| 61469 | 53556020 | Fenbufen 300mg capsules (A A H Pharmaceuticals Ltd) | Fenbufen |
| 10481 | 54744020 | Lederfen f 450mg Tablet (Wyeth Pharmaceuticals) | Fenbufen |
| 17131 | 57844020 | Lederfen 300mg tablets (Mercury Pharma Group Ltd) | Fenbufen |
| 16170 | 59106020 | Fenbufen 300mg capsules (Genus Pharmaceuticals Ltd) | Fenbufen |
| 7424 | 61799020 | Fenbufen 300mg capsules | Fenbufen |
| 8145 | 61800020 | Fenbufen 300mg tablets | Fenbufen |
| 8544 | 61801020 | Fenbufen 450mg tablets | Fenbufen |
| 14422 | 61806020 | Fenbufen 450mg Effervescent tablet | Fenbufen |
| 26994 | 74757020 | Fenbuzip 300mg Tablet (Ashbourne Pharmaceuticals Ltd) | Fenbufen |
| 26214 | 74758020 | Fenbuzip 450mg Tablet (Ashbourne Pharmaceuticals Ltd) | Fenbufen |
| 26205 | 74759020 | Fenbuzip 300mg Capsule (Ashbourne Pharmaceuticals Ltd) | Fenbufen |
| 16176 | 77247020 | Lederfen 450mg tablets (Mercury Pharma Group Ltd) | Fenbufen |
| 14380 | 84600020 | Lederfen 300mg capsules (Mercury Pharma Group Ltd) | Fenbufen |
| 10678 | 49424020 | Fenopron 300 tablets (Typharm Ltd) | Fenoprofen calcium |
| 10589 | 49425020 | Fenopron 600 tablets (Typharm Ltd) | Fenoprofen calcium |
| 17754 | 51288020 | Progesic 200mg Tablet (Eli Lilly and Company Ltd) | Fenoprofen Calcium |
| 4564 | 61812020 | Fenoprofen 200mg Tablet | Fenoprofen Calcium |
| 4469 | 61813020 | Fenoprofen 300mg tablets | Fenoprofen calcium |
| 4565 | 61814020 | Fenoprofen 600mg tablets | Fenoprofen calcium |
| 3182 | 49559020 | Froben 50mg tablets (Abbott Laboratories Ltd) | Flurbiprofen |
| 6249 | 49560020 | Froben 100mg tablets (Abbott Laboratories Ltd) | Flurbiprofen |
| 7490 | 49561020 | Froben 100mg suppositories (Abbott Laboratories Ltd) | Flurbiprofen |
| 34725 | 55428020 | Flurbiprofen 50mg Tablet (Bristol-Myers Squibb Pharmaceuticals Ltd) | Flurbiprofen |
| 3266 | 62061020 | Flurbiprofen 50mg tablets | Flurbiprofen |
| 2366 | 62062020 | Flurbiprofen 100mg tablets | Flurbiprofen |
| 15501 | 62063020 | Flurbiprofen 100mg suppositories | Flurbiprofen |
| 4043 | 68387020 | Froben sr 200mg Modified-release capsule (Abbott Laboratories Ltd) | Flurbiprofen |
| 9439 | 68435020 | Flurbiprofen 200mg modified-release capsules | Flurbiprofen |
| 38944 | 95551020 | Froben SR 200mg capsules (Abbott Laboratories Ltd) | Flurbiprofen |
| 59553 | 8133020 | Ibuprofen 200mg tablets (Alliance Healthcare (Distribution) Ltd) | Ibuprofen |
| 51614 | 8135020 | Ibuprofen 200mg caplets (Lloyds Pharmacy Ltd) | Ibuprofen |
| 50266 | 8136020 | Ibuprofen 200mg caplets (The Boots Company Plc) | Ibuprofen |
| 68582 | 8138020 | Ibuprofen 200mg tablets (DE Pharmaceuticals) | Ibuprofen |
| 65514 | 8139020 | Ibuprofen 200mg tablets (Boston Healthcare Ltd) | Ibuprofen |
| 49277 | 8143020 | Ibuprofen 200mg caplets (Bristol Laboratories Ltd) | Ibuprofen |
| 56213 | 8145020 | Ibuprofen 400mg tablets sugar coated (Kent Pharmaceuticals Ltd) | Ibuprofen |
| 57112 | 8146020 | Ibuprofen 400mg tablets (Alliance Healthcare (Distribution) Ltd) | Ibuprofen |
| 48644 | 8148020 | Ibuprofen 400mg caplets (Lloyds Pharmacy Ltd) | Ibuprofen |
| 50628 | 8149020 | Ibuprofen 400mg caplets (The Boots Company Plc) | Ibuprofen |
| 66544 | 8152020 | Ibuprofen 400mg tablets (DE Pharmaceuticals) | Ibuprofen |
| 55434 | 8154020 | Ibuprofen 400mg tablets (Bristol Laboratories Ltd) | Ibuprofen |
| 48546 | 8156020 | Ibuprofen 400mg caplets (Bristol Laboratories Ltd) | Ibuprofen |
| 59562 | 8162020 | Ibuprofen 600mg tablets (Boston Healthcare Ltd) | Ibuprofen |
| 58652 | 8163020 | Ibuprofen 600mg tablets (Sigma Pharmaceuticals Plc) | Ibuprofen |
| 55009 | 8167020 | Brufen 600mg effervescent granules sachets (Necessity Supplies Ltd) | Ibuprofen |
| 50314 | 8169020 | Brufen 600mg effervescent granules sachets (DE Pharmaceuticals) | Ibuprofen |
| 48326 | 8170020 | Ibuprofen 100mg/5ml oral suspension sugar free | Ibuprofen |
| 53331 | 8171020 | Ibuprofen 100mg/5ml oral suspension sugar free (Alliance Healthcare (Distribution) Ltd) | Ibuprofen |
| 51943 | 8172020 | Orbifen For Children 100mg/5ml oral suspension (Orbis Consumer Products Ltd) | Ibuprofen |
| 50652 | 8173020 | Junior Ibuprofen 100mg/5ml oral suspension (Numark Ltd) | Ibuprofen |
| 48738 | 8174020 | Nurofen for Children 100mg/5ml oral suspension orange (Reckitt Benckiser Healthcare (UK) Ltd) | Ibuprofen |
| 51828 | 8175020 | Ibuprofen 100mg/5ml oral suspension sugar free (Kent Pharmaceuticals Ltd) | Ibuprofen |
| 65121 | 8176020 | Fenpaed 100mg/5ml oral suspension (Pinewood Healthcare) | Ibuprofen |
| 49432 | 8177020 | Calprofen 100mg/5ml oral suspension (McNeil Products Ltd) | Ibuprofen |
| 49133 | 8180020 | Nurofen for Children 100mg/5ml oral suspension strawberry (Reckitt Benckiser Healthcare (UK) Ltd) | Ibuprofen |
| 49266 | 8183020 | Ibuprofen for Children 100mg/5ml oral suspension (Galpharm International Ltd) | Ibuprofen |
| 52617 | 8184020 | Ibuprofen 100mg/5ml oral suspension sugar free (Sigma Pharmaceuticals Plc) | Ibuprofen |
| 71072 | 8185020 | Ibuprofen 100mg/5ml oral suspension sugar free (Phoenix Healthcare Distribution Ltd) | Ibuprofen |
| 59203 | 8188020 | Brufen 100mg/5ml syrup (Sigma Pharmaceuticals Plc) | Ibuprofen |
| 53604 | 9766020 | Ibuprofen 200mg capsules (Numark Ltd) | Ibuprofen |
| 53803 | 9767020 | Ibuprofen 200mg capsules (Kent Pharmaceuticals Ltd) | Ibuprofen |
| 52009 | 9769020 | Ibuprofen 200mg capsules (Galpharm International Ltd) | Ibuprofen |
| 48562 | 14177020 | Ibuprofen 100mg/5ml oral suspension 5ml sachets sugar free | Ibuprofen |
| 51769 | 14178020 | Nurofen for Children Singles 100mg/5ml oral suspension 5ml sachets orange (Reckitt Benckiser Healthcare (UK) Ltd) | Ibuprofen |
| 50363 | 14181020 | Nurofen for Children Singles 100mg/5ml oral suspension 5ml sachets strawberry (Reckitt Benckiser Healthcare (UK) Ltd) | Ibuprofen |
| 56441 | 14184020 | Calprofen 100mg/5ml oral suspension 5ml sachets (McNeil Products Ltd) | Ibuprofen |
| 63079 | 14563021 | Ibuprofen 400mg tablets (Waymade Healthcare Plc) | Ibuprofen |
| 56039 | 14564021 | Ibuprofen 600mg tablets (Waymade Healthcare Plc) | Ibuprofen |
| 63036 | 15908021 | Ibuprofen 400mg capsules (AM Distributions (Yorkshire) Ltd) | Ibuprofen |
| 59067 | 17816021 | Ibuprofen 200mg capsules (AM Distributions (Yorkshire) Ltd) | Ibuprofen |
| 59502 | 21430021 | Nurofen for Children Cold, Pain and Fever Strawberry Flavour 100mg/5ml oral suspension (Reckitt Benckiser Healthcare (UK) Ltd) | Ibuprofen |
| 60510 | 21431021 | Nurofen for Children Cold, Pain and Fever Orange Flavour 100mg/5ml oral suspension (Reckitt Benckiser Healthcare (UK) Ltd) | Ibuprofen |
| 62238 | 26783021 | Ibuprofen 200mg capsules (Colorama Pharmaceuticals Ltd) | Ibuprofen |
| 61953 | 29732021 | Ibuprofen 200mg caplets (Wockhardt UK Ltd) | Ibuprofen |
| 53397 | 38583020 | Brufen 100mg/5ml syrup (Mawdsley-Brooks & Company Ltd) | Ibuprofen |
| 50117 | 39329020 | Brufen 100mg/5ml syrup (Lexon (UK) Ltd) | Ibuprofen |
| 71041 | 43872020 | Ibuprofen 600mg tablets (Bristol Laboratories Ltd) | Ibuprofen |
| 61878 | 45498020 | Nurofen Express Period Pain 200mg capsules (Reckitt Benckiser Healthcare (UK) Ltd) | Ibuprofen |
| 65591 | 48064021 | Ibuprofen 200mg tablets (Crescent Pharma Ltd) | Ibuprofen |
| 65025 | 48065021 | Ibuprofen 400mg tablets (Crescent Pharma Ltd) | Ibuprofen |
| 1621 | 48493020 | Brufen 200mg tablets (Abbott Laboratories Ltd) | Ibuprofen |
| 360 | 48494020 | Brufen 100mg/5ml syrup (Mylan) | Ibuprofen |
| 66194 | 49091021 | Care Ibuprofen for Children 100mg/5ml oral suspension (Thornton & Ross Ltd) | Ibuprofen |
| 34911 | 49827020 | Ibuprofen 200mg Tablet (Celltech Pharma Europe Ltd) | Ibuprofen |
| 45842 | 49828020 | Ibuprofen 600mg Tablet (Celltech Pharma Europe Ltd) | Ibuprofen |
| 34889 | 49829020 | Ibuprofen 400mg Tablet (Celltech Pharma Europe Ltd) | Ibuprofen |
| 16001 | 49832020 | Ibuprofen 200mg tablets (A A H Pharmaceuticals Ltd) | Ibuprofen |
| 19046 | 49833020 | Ibuprofen 400mg tablets (A A H Pharmaceuticals Ltd) | Ibuprofen |
| 32100 | 49834020 | Ibuprofen 600mg tablets (A A H Pharmaceuticals Ltd) | Ibuprofen |
| 14385 | 50220020 | Cuprofen 200mg Tablet (SSL International Plc) | Ibuprofen |
| 11980 | 50221020 | Cuprofen 400mg Tablet (SSL International Plc) | Ibuprofen |
| 1392 | 50928020 | Ibuprofen 800mg modified-release tablets | Ibuprofen |
| 3599 | 50929020 | Ibuprofen 600mg effervescent granules sachets | Ibuprofen |
| 30243 | 50930020 | Ibuprofen 200mg effervescent tablets | Ibuprofen |
| 392 | 53037020 | Ibuprofen 200mg modified-release capsules | Ibuprofen |
| 34850 | 53364020 | Ibuprofen 600mg tablets (Teva UK Ltd) | Ibuprofen |
| 27782 | 53365020 | Ibuprofen 400mg tablets (Teva UK Ltd) | Ibuprofen |
| 28348 | 53366020 | Ibuprofen 200mg tablets (Teva UK Ltd) | Ibuprofen |
| 68018 | 53630021 | Ibuprofen 200mg tablets (Mawdsley-Brooks & Company Ltd) | Ibuprofen |
| 10785 | 53757020 | Fenbid 300mg Spansules (Mercury Pharma Group Ltd) | Ibuprofen |
| 34425 | 53852020 | Ibuprofen 400mg Tablet (Family Health) | Ibuprofen |
| 34980 | 53948020 | Ibuprofen 200mg tablets sugar coated (Actavis UK Ltd) | Ibuprofen |
| 27783 | 53949020 | Ibuprofen 400mg tablets sugar coated (Actavis UK Ltd) | Ibuprofen |
| 41701 | 53950020 | Ibuprofen 600mg tablets (Actavis UK Ltd) | Ibuprofen |
| 30382 | 53953020 | Ibuprofen 200mg Tablet (C P Pharmaceuticals Ltd) | Ibuprofen |
| 45216 | 53954020 | Ibuprofen 400mg Tablet (C P Pharmaceuticals Ltd) | Ibuprofen |
| 43911 | 53955020 | Ibuprofen 600mg Tablet (C P Pharmaceuticals Ltd) | Ibuprofen |
| 1739 | 53998020 | Brufen 400mg tablets (Mylan) | Ibuprofen |
| 4216 | 54001020 | Brufen 600mg tablets (Mylan) | Ibuprofen |
| 46942 | 55366020 | Ibuprofen 600mg tablets (IVAX Pharmaceuticals UK Ltd) | Ibuprofen |
| 25800 | 55616020 | Feverfen 100mg/5ml oral suspension (Wise Pharmaceuticals Ltd) | Ibuprofen |
| 34359 | 56114020 | Ibuprofen 400mg tablets (Vantage) | Ibuprofen |
| 34354 | 56266020 | Ibuprofen 200mg tablets (Vantage) | Ibuprofen |
| 16192 | 56328020 | Motrin 200mg Tablet (Pharmacia Ltd) | Ibuprofen |
| 8401 | 56329020 | Motrin 400mg tablets (Pfizer Ltd) | Ibuprofen |
| 17201 | 56330020 | Motrin 600mg tablets (Pfizer Ltd) | Ibuprofen |
| 25794 | 56702020 | Isisfen 400mg Tablet (Isis Products Ltd) | Ibuprofen |
| 34447 | 57304020 | Ibuprofen 200mg tablets (Thornton & Ross Ltd) | Ibuprofen |
| 33589 | 57305020 | Ibuprofen 400mg tablets (Thornton & Ross Ltd) | Ibuprofen |
| 31482 | 57638020 | Apsifen 200mg Tablet (Approved Prescription Services Ltd) | Ibuprofen |
| 27968 | 57639020 | Apsifen 400mg Tablet (Approved Prescription Services Ltd) | Ibuprofen |
| 31469 | 57645020 | Apsifen -f 600mg Tablet (Approved Prescription Services Ltd) | Ibuprofen |
| 41513 | 57811020 | Ibuprofen 200mg tablets (IVAX Pharmaceuticals UK Ltd) | Ibuprofen |
| 34536 | 57812020 | Ibuprofen 400mg tablets (IVAX Pharmaceuticals UK Ltd) | Ibuprofen |
| 32136 | 58455020 | Ibular 200mg Tablet (Lagap) | Ibuprofen |
| 18364 | 58456020 | Ibular 400mg Tablet (Lagap) | Ibuprofen |
| 416 | 59354020 | Ibuprofen 200mg tablets | Ibuprofen |
| 15 | 59355020 | Ibuprofen 400mg tablets | Ibuprofen |
| 1086 | 59356020 | Ibuprofen 600mg tablets | Ibuprofen |
| 29587 | 59451020 | Ebufac 400mg Tablet (DDSA Pharmaceuticals Ltd) | Ibuprofen |
| 21045 | 59456020 | Ibumetin 400mg Tablet (Alfred Benzon (UK) Ltd) | Ibuprofen |
| 29704 | 59460020 | Paxofen 200mg Tablet (M A Steinhard Ltd) | Ibuprofen |
| 45320 | 59470020 | Ibuprofen 200mg tablets (Sandoz Ltd) | Ibuprofen |
| 32875 | 59471020 | Ibuprofen 400mg tablets (Sandoz Ltd) | Ibuprofen |
| 34961 | 59472020 | Ibuprofen 600mg tablets (Sandoz Ltd) | Ibuprofen |
| 21811 | 59661020 | Lidifen 200mg Tablet (Berk Pharmaceuticals Ltd) | Ibuprofen |
| 21813 | 59662020 | Lidifen 400mg Tablet (Berk Pharmaceuticals Ltd) | Ibuprofen |
| 21821 | 59663020 | Lidifen f 600mg Tablet (Berk Pharmaceuticals Ltd) | Ibuprofen |
| 34757 | 59877020 | Ibuprofen 400mg Tablet (Unichem) | Ibuprofen |
| 34527 | 59965020 | Ibuprofen 200mg tablets (Zentiva) | Ibuprofen |
| 32242 | 59966020 | Ibuprofen 400mg tablets (Sterwin Medicines) | Ibuprofen |
| 29749 | 60159020 | Ibuprofen 200mg tablets (Ranbaxy (UK) Ltd) | Ibuprofen |
| 46921 | 60160020 | Ibuprofen 400mg tablets (Ranbaxy (UK) Ltd) | Ibuprofen |
| 70878 | 60950021 | Nurofen Joint & Back Pain Relief 200mg capsules (Reckitt Benckiser Healthcare (UK) Ltd) | Ibuprofen |
| 68220 | 61380021 | Ibuprofen 200mg capsules (Ennogen Healthcare Ltd) | Ibuprofen |
| 67594 | 61805020 | Ibuprofen 400mg tablets (Kent Pharmaceuticals Ltd) | Ibuprofen |
| 40253 | 62183020 | Ibuprofen 600mg Tablet (Sovereign Medical Ltd) | Ibuprofen |
| 42108 | 62385020 | Ibuprofen 200mg tablets (OBG Pharmaceuticals Ltd) | Ibuprofen |
| 34729 | 62386020 | Ibuprofen 400mg tablets (OBG Pharmaceuticals Ltd) | Ibuprofen |
| 19036 | 62435020 | Arthrofen 200 tablets (Ashbourne Pharmaceuticals Ltd) | Ibuprofen |
| 15068 | 62436020 | Arthrofen 400 tablets (Ashbourne Pharmaceuticals Ltd) | Ibuprofen |
| 21815 | 62437020 | Arthrofen 600 tablets (Ashbourne Pharmaceuticals Ltd) | Ibuprofen |
| 45331 | 62584020 | Ibuprofen 200mg Tablet (Co-Pharma Ltd) | Ibuprofen |
| 29332 | 62712020 | Ibuprofen 100mg/5ml oral suspension sugar free (Sandoz Ltd) | Ibuprofen |
| 34931 | 62843020 | Ibuprofen 200mg Tablet (Regent Laboratories Ltd) | Ibuprofen |
| 19575 | 63156020 | Proflex 200mg Tablet (Novartis Consumer Health UK Ltd) | Ibuprofen |
| 30811 | 63157020 | Proflex 300mg Modified-release capsule (Novartis Consumer Health UK Ltd) | Ibuprofen |
| 784 | 63501020 | Ibuprofen 300mg modified-release capsules | Ibuprofen |
| 647 | 63502020 | Ibuprofen 100mg/5ml oral suspension | Ibuprofen |
| 2938 | 63503020 | Ibuprofen 100mg/5ml Oral suspension | Ibuprofen |
| 33704 | 63505020 | Ibuprofen 100mg/5ml oral suspension sugar free (A A H Pharmaceuticals Ltd) | Ibuprofen |
| 29524 | 63506020 | Ibumetin 600mg Tablet (Alfred Benzon (UK) Ltd) | Ibuprofen |
| 33357 | 64641020 | Pacifene 200mg tablets (Sussex Pharmaceutical Ltd) | Ibuprofen |
| 29345 | 65371020 | Ibuprofen 100mg/5ml Oral suspension (Hillcross Pharmaceuticals Ltd) | Ibuprofen |
| 66247 | 65964021 | Ibuprofen 100mg chewable capsules | Ibuprofen |
| 70299 | 66077021 | Ibuprofen 200mg capsules (Bell,Sons & Co (Druggists) Ltd) | Ibuprofen |
| 34621 | 66405020 | Ibuprofen 200mg Tablet (Nucare Plc) | Ibuprofen |
| 55233 | 66410020 | Ibuprofen 400mg Tablet (Nucare Plc) | Ibuprofen |
| 52154 | 67026020 | Ibuprofen 200mg tablets (Galpharm International Ltd) | Ibuprofen |
| 29352 | 68023020 | Ibuprofen 100mg/5ml oral suspension sugar free (Vantage) | Ibuprofen |
| 11550 | 68069020 | Nurofen Meltlets 200mg tablets (Reckitt Benckiser Healthcare (UK) Ltd) | Ibuprofen |
| 34663 | 68074020 | Ibuprofen 100mg/5ml Oral suspension (Neo Laboratories Ltd) | Ibuprofen |
| 2622 | 68109020 | Ibuprofen 800mg tablets | Ibuprofen |
| 16193 | 68112020 | Motrin 800mg tablets (Pfizer Ltd) | Ibuprofen |
| 26970 | 68245020 | Ibuprofen 100mg/5ml oral suspension sugar free (Teva UK Ltd) | Ibuprofen |
| 2129 | 68365020 | Brufen retard tabs 800mg Modified-release tablet (Abbott Laboratories Ltd) | Ibuprofen |
| 407 | 68366020 | Brufen 600mg effervescent granules sachets (Mylan) | Ibuprofen |
| 48138 | 68817020 | Ibuprofen 200mg tablets (Aspar Pharmaceuticals Ltd) | Ibuprofen |
| 54137 | 68820020 | Ibuprofen 400mg tablets (Aspar Pharmaceuticals Ltd) | Ibuprofen |
| 60035 | 69002020 | Ibuprofen 200mg tablets film coated (Actavis UK Ltd) | Ibuprofen |
| 34550 | 69005020 | Ibuprofen 400mg tablets film coated (Actavis UK Ltd) | Ibuprofen |
| 65471 | 69610020 | Ibuprofen 200mg tablets (Almus Pharmaceuticals Ltd) | Ibuprofen |
| 66648 | 69615020 | Ibuprofen 400mg tablets (Almus Pharmaceuticals Ltd) | Ibuprofen |
| 32862 | 71142020 | Ibuprofen 100mg/5ml oral suspension sugar free (Thornton & Ross Ltd) | Ibuprofen |
| 1468 | 71645020 | Ibuprofen 200mg Soluble tablet | Ibuprofen |
| 1030 | 72737020 | Junifen 100mg/5ml Oral suspension (Crookes Healthcare Ltd) | Ibuprofen |
| 67740 | 73278020 | Ibuprofen 600mg tablets (Fannin UK Ltd) | Ibuprofen |
| 4298 | 73681020 | Nurofen 200mg Tablet (Crookes Healthcare Ltd) | Ibuprofen |
| 3597 | 73682020 | Nurofen 200mg Soluble tablet (Crookes Healthcare Ltd) | Ibuprofen |
| 25619 | 73683020 | Nurofen 400mg Tablet (Crookes Healthcare Ltd) | Ibuprofen |
| 24469 | 73716020 | Cuprofen for Children 100mg/5ml oral suspension (SSL International Plc) | Ibuprofen |
| 32509 | 73886020 | Anadin Ibuprofen 200mg tablets (Pfizer Consumer Healthcare Ltd) | Ibuprofen |
| 849 | 74754020 | Ibumed 400mg Tablet (Medipharma Ltd) | Ibuprofen |
| 18812 | 76985020 | Nurofen meltlets lemon 200mg Orodispersible tablet (Reckitt Benckiser Healthcare (UK) Ltd) | Ibuprofen |
| 62892 | 77148020 | Ibuprofen 200mg tablets (Wockhardt UK Ltd) | Ibuprofen |
| 20978 | 77321020 | Anadin Ultra liquid capsules (Wyeth Consumer Healthcare) | Ibuprofen |
| 55313 | 77645020 | Ibuprofen 400mg tablets (Boston Healthcare Ltd) | Ibuprofen |
| 48062 | 78798020 | Ibuprofen 200mg Tablet (Wockhardt UK Ltd) | Ibuprofen |
| 10209 | 80061020 | Ibufem 200mg tablets (Galpharm International Ltd) | Ibuprofen |
| 4911 | 80143020 | Ibuprofen 400mg Granules | Ibuprofen |
| 586 | 80144020 | Ibuprofen 200mg Capsule | Ibuprofen |
| 5648 | 80145020 | Ibuprofen 200mg orodispersible tablets sugar free | Ibuprofen |
| 24305 | 80560020 | Ibufac 400mg Tablet (DDSA Pharmaceuticals Ltd) | Ibuprofen |
| 18196 | 81617020 | Orbifen for children 100mg/5ml Oral suspension (Orbis Consumer Products Ltd) | Ibuprofen |
| 36597 | 82132020 | Hedex Ibuprofen 200mg tablets (Omega Pharma Ltd) | Ibuprofen |
| 32366 | 82148020 | Relcofen 200mg Tablet (Actavis UK Ltd) | Ibuprofen |
| 32365 | 82149020 | Relcofen 400mg tablets (Actavis UK Ltd) | Ibuprofen |
| 37053 | 82155020 | Migrafen 200mg tablets (Chatfield Laboratories) | Ibuprofen |
| 22206 | 82951020 | Nurofen Long Lasting 300mg capsules (Crookes Healthcare Ltd) | Ibuprofen |
| 25257 | 83095020 | Advil 200mg tablets (Wyeth Consumer Healthcare) | Ibuprofen |
| 40394 | 83096020 | Advil 400mg Tablet (Wyeth Consumer Healthcare) | Ibuprofen |
| 28168 | 83270020 | Nurofen Recovery 200mg orodispersible tablets (Reckitt Benckiser Healthcare (UK) Ltd) | Ibuprofen |
| 36606 | 83343020 | Manorfen 400mg tablets (The Manor Drug Company (Nottingham) Ltd) | Ibuprofen |
| 33785 | 83372020 | Galprofen 200mg tablets (Galpharm International Ltd) | Ibuprofen |
| 7058 | 83707020 | Calprofen 100mg/5ml Oral suspension (McNeil Products Ltd) | Ibuprofen |
| 18820 | 84269020 | Fenpaed 100mg/5ml Oral suspension (Pinewood Healthcare) | Ibuprofen |
| 43032 | 84356020 | Inoven 200mg Tablet (Janssen-Cilag Ltd) | Ibuprofen |
| 28888 | 84592020 | Galprofen Long Lasting 200mg capsules (Galpharm International Ltd) | Ibuprofen |
| 30892 | 84841020 | Mandafen for Children 100mg/5ml oral suspension sugar free (M & A Pharmachem Ltd) | Ibuprofen |
| 402 | 85651020 | Nurofen 200mg Tablet (Crookes Healthcare Ltd) | Ibuprofen |
| 7535 | 85652020 | Nurofen 200mg Capsule (Crookes Healthcare Ltd) | Ibuprofen |
| 18527 | 86224020 | Mandafen 400mg tablets (M & A Pharmachem Ltd) | Ibuprofen |
| 10149 | 86472020 | Ibuprofen 200mg capsules | Ibuprofen |
| 29316 | 86537020 | Care ibuprofen 400mg Tablet (Thornton & Ross Ltd) | Ibuprofen |
| 4731 | 86753020 | Nurofen for children 100mg/5ml Oral suspension (Reckitt Benckiser Healthcare (UK) Ltd) | Ibuprofen |
| 30724 | 86821020 | Galprofen 100mg/5ml oral suspension (Galpharm International Ltd) | Ibuprofen |
| 28479 | 88706020 | Nurofen Back Pain SR 300mg capsules (Reckitt Benckiser Healthcare (UK) Ltd) | Ibuprofen |
| 40083 | 88937020 | Ibuprofen 200mg caplets (Galpharm International Ltd) | Ibuprofen |
| 25205 | 89068020 | Ibuprofen 100mg/5ml oral suspension 5ml sachets sugar free (Thornton & Ross Ltd) | Ibuprofen |
| 39354 | 90397020 | Galpharm ibuprofen for children 100mg/5ml Oral suspension (Galpharm International Ltd) | Ibuprofen |
| 14333 | 91245020 | Ibuprofen 400mg capsules | Ibuprofen |
| 29068 | 91247020 | Nurofen Extra Strength 400mg capsules (Reckitt Benckiser Healthcare (UK) Ltd) | Ibuprofen |
| 37253 | 91330020 | Anadin ultra double strength 400mg Capsule (Wyeth Consumer Healthcare) | Ibuprofen |
| 35265 | 93625020 | Nurofen for children 3 months to 9 years 100mg/5ml Oral suspension (Reckitt Benckiser Healthcare (UK) Ltd) | Ibuprofen |
| 36650 | 93686020 | Nurofen 200mg tablets (Reckitt Benckiser Healthcare (UK) Ltd) | Ibuprofen |
| 35890 | 93688020 | Nurofen 200mg caplets (Reckitt Benckiser Healthcare (UK) Ltd) | Ibuprofen |
| 35292 | 93690020 | Nurofen 200mg liquid capsules (Reckitt Benckiser Healthcare (UK) Ltd) | Ibuprofen |
| 37094 | 93812020 | Cuprofen 200mg tablets (SSL International Plc) | Ibuprofen |
| 37648 | 93920020 | Nurofen Express 400mg liquid capsules (Reckitt Benckiser Healthcare (UK) Ltd) | Ibuprofen |
| 37002 | 93924020 | Nurofen Express 200mg liquid capsules (Reckitt Benckiser Healthcare (UK) Ltd) | Ibuprofen |
| 38493 | 94461020 | Anadin Joint Pain 200mg tablets (Pfizer Consumer Healthcare Ltd) | Ibuprofen |
| 38332 | 94471020 | Ibucalm 200mg tablets (Aspar Pharmaceuticals Ltd) | Ibuprofen |
| 37553 | 94475020 | Ibucalm 400mg tablets (Aspar Pharmaceuticals Ltd) | Ibuprofen |
| 48084 | 94572020 | Ibuprofen 200mg/5ml oral suspension | Ibuprofen |
| 39019 | 95831020 | Brufen Retard 800mg tablets (Mylan) | Ibuprofen |
| 39873 | 95907020 | Cuprofen Maximum Strength 400mg tablets (SSL International Plc) | Ibuprofen |
| 40516 | 96455020 | Anadin Ultra 200mg capsules (Pfizer Consumer Healthcare Ltd) | Ibuprofen |
| 43456 | 96457020 | Anadin LiquiFast 400mg capsules (Pfizer Consumer Healthcare Ltd) | Ibuprofen |
| 44233 | 98805020 | Nurofen for children baby 100mg/5ml Oral suspension (Reckitt Benckiser Healthcare (UK) Ltd) | Ibuprofen |
| 46342 | 99120020 | Medifen 3with months 100mg/5ml Oral suspension (SSL International Plc) | Ibuprofen |
| 46860 | 99382020 | Anadin LiquiFast 200mg effervescent tablets (Pfizer Consumer Healthcare Ltd) | Ibuprofen |
| 48568 | 14108020 | Boots Rapid Ibuprofen lysine 342mg tablets (The Boots Company Plc) | Ibuprofen lysine |
| 54514 | 46754020 | Ibuprofen lysine 400mg oral powder sachets | Ibuprofen lysine |
| 55153 | 46755020 | Nurofen Express Soluble 400mg oral powder sachets (Reckitt Benckiser Healthcare (UK) Ltd) | Ibuprofen lysine |
| 23425 | 82652020 | Nurofen Migraine Pain 342mg tablets (Reckitt Benckiser Healthcare (UK) Ltd) | Ibuprofen lysine |
| 4309 | 86342020 | Ibuprofen lysine 200mg tablets | Ibuprofen lysine |
| 24887 | 86353020 | Nurofen Advance 200mg tablets (Crookes Healthcare Ltd) | Ibuprofen lysine |
| 46141 | 89638020 | Nurofen Tension Headache 342mg caplets (Reckitt Benckiser Healthcare (UK) Ltd) | Ibuprofen lysine |
| 26095 | 90473020 | Ibuprofen lysine 400mg tablets | Ibuprofen lysine |
| 33935 | 90475020 | Nurofen Maximum Strength Migraine Pain 684mg caplets (Reckitt Benckiser Healthcare (UK) Ltd) | Ibuprofen lysine |
| 37731 | 93916020 | Nurofen Express 342mg caplets (Reckitt Benckiser Healthcare (UK) Ltd) | Ibuprofen lysine |
| 36787 | 93922020 | Nurofen Express 684mg caplets (Reckitt Benckiser Healthcare (UK) Ltd) | Ibuprofen lysine |
| 43904 | 98003020 | Feminax Express 342mg tablets (Bayer Plc) | Ibuprofen lysine |
| 69018 | 61136021 | Nurofen Joint & Back Pain Relief 256mg tablets (Reckitt Benckiser Healthcare (UK) Ltd) | Ibuprofen sodium dihydrate |
| 39502 | 95773020 | Ibuprofen sodium dihydrate 200mg tablets | Ibuprofen sodium dihydrate |
| 66567 | 95775020 | Ibuprofen sodium dihydrate 400mg tablets | Ibuprofen sodium dihydrate |
| 39758 | 95779020 | Nurofen Express 256mg caplets (Reckitt Benckiser Healthcare (UK) Ltd) | Ibuprofen sodium dihydrate |
| 44483 | 95783020 | Nurofen Express 512mg tablets (Reckitt Benckiser Healthcare (UK) Ltd) | Ibuprofen sodium dihydrate |
| 42397 | 95785020 | Nurofen Express 256mg tablets (Reckitt Benckiser Healthcare (UK) Ltd) | Ibuprofen sodium dihydrate |
| 1708 | 54315020 | Codafen Continus tablets (Napp Pharmaceuticals Ltd) | Ibuprofen/Codeine phosphate |
| 11461 | 59563020 | Ibuprofen 300mg modified-release / Codeine 20mg tablets | Ibuprofen/Codeine phosphate |
| 69285 | 60686021 | Boots Ibuprofen and Codeine 200mg/12.8mg tablets (The Boots Company Plc) | Ibuprofen/Codeine phosphate |
| 13893 | 77456020 | Nurofen Plus tablets (Reckitt Benckiser Healthcare (UK) Ltd) | Ibuprofen/Codeine phosphate |
| 11554 | 77460020 | Ibuprofen 200mg / Codeine 12.8mg tablets | Ibuprofen/Codeine phosphate |
| 53617 | 78855020 | Ibuprofen and codeine 200mg+12.8mg Tablet (Almus Pharmaceuticals Ltd) | Ibuprofen/Codeine phosphate |
| 25330 | 82982020 | Solpaflex tablets (GlaxoSmithKline Consumer Healthcare) | Ibuprofen/Codeine phosphate |
| 37816 | 87860020 | Cuprofen PLUS tablets (SSL International Plc) | Ibuprofen/Codeine phosphate |
| 39461 | 90563020 | Solpadeine Migraine Ibuprofen & Codeine tablets (Omega Pharma Ltd) | Ibuprofen/Codeine phosphate |
| 28522 | 75899020 | Ibuprofen 200mg / Pseudoephedrine hydrochloride 30mg tablets | Ibuprofen/Pseudoephedrine hydrochloride |
| 15363 | 75904020 | Nurofen Cold and Flu tablets (Reckitt Benckiser Healthcare (UK) Ltd) | Ibuprofen/Pseudoephedrine hydrochloride |
| 20907 | 82037020 | Sudafed Sinus Pressure & Pain tablets (McNeil Products Ltd) | Ibuprofen/Pseudoephedrine hydrochloride |
| 37235 | 94243020 | Ibuprofen 100mg/5ml / Pseudoephedrine 15mg/5ml oral suspension sugar free | Ibuprofen/Pseudoephedrine hydrochloride |
| 38182 | 94245020 | Orbifen Cold & Flu oral suspension (Orbis Consumer Products Ltd) | Ibuprofen/Pseudoephedrine hydrochloride |
| 60930 | 8191020 | Indometacin 25mg capsules (Alliance Healthcare (Distribution) Ltd) | Indometacin |
| 60916 | 8210020 | Indometacin 75mg modified-release capsules (Kent Pharmaceuticals Ltd) | Indometacin |
| 60772 | 20162020 | Indometacin 25mg/5ml oral solution | Indometacin |
| 68708 | 21838021 | Indometacin 75mg modified-release capsules (DE Pharmaceuticals) | Indometacin |
| 51339 | 40591020 | Indometacin 25mg capsules (Genesis Pharmaceuticals Ltd) | Indometacin |
| 41817 | 49850020 | Indometacin sr 75mg Modified-release capsule (C P Pharmaceuticals Ltd) | Indometacin |
| 24137 | 49854020 | Indometacin 25mg capsules (Actavis UK Ltd) | Indometacin |
| 33321 | 49855020 | Indometacin 50mg capsules (Actavis UK Ltd) | Indometacin |
| 34199 | 49856020 | Indometacin 100mg suppositories (Actavis UK Ltd) | Indometacin |
| 32641 | 49864020 | Indometacin 25mg capsules (A A H Pharmaceuticals Ltd) | Indometacin |
| 31959 | 49865020 | Indometacin 50mg capsules (A A H Pharmaceuticals Ltd) | Indometacin |
| 24193 | 49913020 | Imbrilon 25mg Capsule (Berk Pharmaceuticals Ltd) | Indometacin |
| 24212 | 49914020 | Imbrilon 50mg Capsule (Berk Pharmaceuticals Ltd) | Indometacin |
| 23795 | 49915020 | Imbrilon 100mg Suppository (Berk Pharmaceuticals Ltd) | Indometacin |
| 120 | 49932020 | Indocid 25mg capsules (Merck Sharp & Dohme Ltd) | Indometacin |
| 1688 | 49933020 | Indocid 50mg capsules (Merck Sharp & Dohme Ltd) | Indometacin |
| 10625 | 49934020 | Indocid 5mg/ml oral suspension (Merck Sharp & Dohme Ltd) | Indometacin |
| 41615 | 53369020 | Indometacin 50mg Capsule (Approved Prescription Services Ltd) | Indometacin |
| 41521 | 53370020 | Indometacin 25mg Capsule (Approved Prescription Services Ltd) | Indometacin |
| 41823 | 53374020 | Indometacin sr 75mg Modified-release capsule (Generics (UK) Ltd) | Indometacin |
| 28900 | 53375020 | Indometacin 25mg Capsule (Generics (UK) Ltd) | Indometacin |
| 33318 | 53376020 | Indometacin 50mg Capsule (Generics (UK) Ltd) | Indometacin |
| 920 | 54426020 | Indocid 100mg suppositories (Aspen Pharma Trading Ltd) | Indometacin |
| 1496 | 54429020 | Indocid R 75mg capsules (Merck Sharp & Dohme Ltd) | Indometacin |
| 62643 | 55788020 | Indometacin 50mg Capsule (Carter Wallace Ltd) | Indometacin |
| 24320 | 58460020 | Indolar 50mg Capsule (Lagap) | Indometacin |
| 26083 | 58461020 | Indolar 100mg Suppository (Lagap) | Indometacin |
| 14476 | 58464020 | Indolar SR 75mg capsules (Sandoz Ltd) | Indometacin |
| 177 | 58913020 | Indometacin 25mg capsules | Indometacin |
| 736 | 58914020 | Indometacin 50mg capsules | Indometacin |
| 1210 | 58915020 | Indometacin 75mg modified-release capsules | Indometacin |
| 919 | 58918020 | Indometacin 100mg suppositories | Indometacin |
| 33113 | 58999020 | Artracin 50mg Capsule (DDSA Pharmaceuticals Ltd) | Indometacin |
| 44313 | 59004020 | Indoflex 25mg Capsule (Unimed Pharmaceuticals Ltd) | Indometacin |
| 52141 | 59007020 | Mobilan 25mg Capsule (Galen Ltd) | Indometacin |
| 24308 | 59011020 | Slo-Indo 75mg capsules (Mylan) | Indometacin |
| 42003 | 59423020 | Indometacin sr 75mg Capsule (Lagap) | Indometacin |
| 34190 | 59891020 | Indometacin 75mg modified-release capsules (A A H Pharmaceuticals Ltd) | Indometacin |
| 32097 | 59898020 | Indometacin 75mg Modified-release capsule (Actavis UK Ltd) | Indometacin |
| 45256 | 62429020 | Indometacin 25mg Capsule (Meridian Healthcare (UK) Ltd) | Indometacin |
| 36577 | 62430020 | Indometacin 50mg Capsule (Meridian Healthcare (UK) Ltd) | Indometacin |
| 3168 | 63544020 | Indometacin 25mg/5ml oral suspension sugar free | Indometacin |
| 1051 | 63545020 | Indometacin 75mg modified-release tablets | Indometacin |
| 2671 | 63546020 | Indometacin 50mg modified-release tablets | Indometacin |
| 15005 | 63549020 | Indomod 25mg modified-release capsules (Pfizer Ltd) | Indometacin |
| 18662 | 63550020 | Indomod 75mg modified-release capsules (Pfizer Ltd) | Indometacin |
| 18234 | 63553020 | Rheumacin LA 75mg capsules (Hillcross Pharmaceuticals Ltd) | Indometacin |
| 3216 | 67393020 | Indometacin 25mg modified-release tablets | Indometacin |
| 10558 | 68265020 | Flexin-75 Continus tablets (Napp Pharmaceuticals Ltd) | Indometacin |
| 13639 | 68266020 | Flexin-50 Continus tablets (Napp Pharmaceuticals Ltd) | Indometacin |
| 13606 | 68267020 | Flexin-25 Continus tablets (Napp Pharmaceuticals Ltd) | Indometacin |
| 58523 | 72599020 | Indometacin 50mg capsules (Almus Pharmaceuticals Ltd) | Indometacin |
| 67756 | 73417020 | Indometacin 25mg capsules (Almus Pharmaceuticals Ltd) | Indometacin |
| 70904 | 73878021 | Indometacin 25mg capsules (Crescent Pharma Ltd) | Indometacin |
| 17750 | 74793020 | Indomax 25mg Capsule (Ashbourne Pharmaceuticals Ltd) | Indometacin |
| 17680 | 74794020 | Indomax 75 SR capsules (Ashbourne Pharmaceuticals Ltd) | Indometacin |
| 23026 | 77482020 | Artracin sr 75mg Modified-release capsule (Trinity Pharmaceuticals Ltd) | Indometacin |
| 2200 | 80094020 | Indometacin 25mg modified-release capsules | Indometacin |
| 23204 | 85828020 | Pardelprin MR 75mg capsules (Actavis UK Ltd) | Indometacin |
| 71127 | 8217020 | Oruvail 100 modified-release capsules (Waymade Healthcare Plc) | Ketoprofen |
| 71104 | 8221020 | Oruvail 200 modified-release capsules (Waymade Healthcare Plc) | Ketoprofen |
| 67803 | 8225020 | Oruvail 200 modified-release capsules (Lexon (UK) Ltd) | Ketoprofen |
| 13347 | 48091020 | Alrheumat 50mg Capsule (Bayer Plc) | Ketoprofen |
| 12122 | 50919020 | Orudis 50mg Capsule (Hawgreen Ltd) | Ketoprofen |
| 11999 | 50920020 | Orudis 100mg Capsule (Hawgreen Ltd) | Ketoprofen |
| 3326 | 50923020 | Oruvail 100mg Modified-release capsule (Hawgreen Ltd) | Ketoprofen |
| 838 | 50924020 | Oruvail 200mg Modified-release capsule (Hawgreen Ltd) | Ketoprofen |
| 7840 | 50925020 | Oruvail 150mg Modified-release capsule (Hawgreen Ltd) | Ketoprofen |
| 11995 | 52982020 | Orudis 100mg Suppository (Hawgreen Ltd) | Ketoprofen |
| 21955 | 53056020 | Ketozip 200 XL capsules (Ashbourne Pharmaceuticals Ltd) | Ketoprofen |
| 42500 | 53591020 | Ketoprofen sr 100mg Capsule (Approved Prescription Services Ltd) | Ketoprofen |
| 46919 | 53592020 | Ketoprofen sr 200mg Capsule (Approved Prescription Services Ltd) | Ketoprofen |
| 33180 | 53674020 | Ketoprofen cr 200mg Capsule (Bristol-Myers Squibb Pharmaceuticals Ltd) | Ketoprofen |
| 40141 | 55793020 | Ketoprofen 100mg capsules (A A H Pharmaceuticals Ltd) | Ketoprofen |
| 46920 | 56131020 | Ketoprofen 200mg Modified-release capsule (Generics (UK) Ltd) | Ketoprofen |
| 33568 | 57010020 | Ketoprofen 200mg Modified-release capsule (Actavis UK Ltd) | Ketoprofen |
| 46940 | 59918020 | Ketoprofen 100mg capsules (Mylan) | Ketoprofen |
| 389 | 63883020 | Ketoprofen 50mg capsules | Ketoprofen |
| 1231 | 63884020 | Ketoprofen 100mg capsules | Ketoprofen |
| 12000 | 63885020 | Ketoprofen 100mg suppositories | Ketoprofen |
| 1571 | 69511020 | Ketoprofen 100mg modified-release capsules | Ketoprofen |
| 3043 | 69512020 | Ketoprofen 200mg modified-release capsules | Ketoprofen |
| 8385 | 69513020 | Ketoprofen 150mg modified-release capsules | Ketoprofen |
| 10336 | 70001020 | Ketoprofen 100mg/2ml solution for injection ampoules | Ketoprofen |
| 7432 | 74128020 | Oruvail IM 100mg/2ml solution for injection ampoules (Sanofi) | Ketoprofen |
| 17818 | 74336020 | Ketovail 100mg modified-release capsules (Teva UK Ltd) | Ketoprofen |
| 25701 | 74337020 | Ketovail 200mg modified-release capsules (Teva UK Ltd) | Ketoprofen |
| 21050 | 74798020 | Ketonal 100mg Capsule (Lagap) | Ketoprofen |
| 15286 | 77485020 | Ketocid 200 modified-release capsules (Chiesi Ltd) | Ketoprofen |
| 18647 | 78164020 | Fenoket 200mg modified-release capsules (Opus Pharmaceuticals Ltd) | Ketoprofen |
| 32227 | 80610020 | Larafen CR 200mg capsules (Ennogen Pharma Ltd) | Ketoprofen |
| 29772 | 82614020 | Ketotard XL 200mg capsules (Galen Ltd) | Ketoprofen |
| 27082 | 84317020 | Ketpron XL 100mg capsules (Mercury Pharma Group Ltd) | Ketoprofen |
| 31962 | 84318020 | Ketpron XL 200mg capsules (Mercury Pharma Group Ltd) | Ketoprofen |
| 30327 | 84738020 | Jomethid XL 200mg capsules (Actavis UK Ltd) | Ketoprofen |
| 31916 | 86741020 | Tiloket CR 100mg capsules (Tillomed Laboratories Ltd) | Ketoprofen |
| 27013 | 86742020 | Tiloket 200mg Modified-release capsule (Tillomed Laboratories Ltd) | Ketoprofen |
| 40336 | 96897020 | Orudis 50mg capsules (Sanofi) | Ketoprofen |
| 40484 | 96899020 | Orudis 100mg capsules (Sanofi) | Ketoprofen |
| 41450 | 96901020 | Orudis 100mg suppositories (Sanofi) | Ketoprofen |
| 40215 | 96903020 | Oruvail 100 modified-release capsules (Sanofi) | Ketoprofen |
| 40185 | 96905020 | Oruvail 200 modified-release capsules (Sanofi) | Ketoprofen |
| 40664 | 96907020 | Oruvail 150 modified-release capsules (Sanofi) | Ketoprofen |
| 57943 | 98801020 | Valket 200 Retard capsules (Tillomed Laboratories Ltd) | Ketoprofen |
| 41364 | 97642020 | Ketoprofen 100mg / Omeprazole 20mg modified-release capsules | Ketoprofen/Omeprazole |
| 41367 | 97644020 | Ketoprofen 200mg / Omeprazole 20mg modified-release capsules | Ketoprofen/Omeprazole |
| 41366 | 97646020 | Axorid 100mg/20mg modified-release capsules (Meda Pharmaceuticals Ltd) | Ketoprofen/Omeprazole |
| 41365 | 97648020 | Axorid 200mg/20mg modified-release capsules (Meda Pharmaceuticals Ltd) | Ketoprofen/Omeprazole |
| 29110 | 83611020 | Lornoxicam 4mg tablets | Lornoxicam |
| 30122 | 83613020 | Lornoxicam 8mg tablets | Lornoxicam |
| 10212 | 88352020 | Lumiracoxib 100mg tablets | Lumiracoxib |
| 28171 | 88356020 | Lumiracoxib 400mg tablets | Lumiracoxib |
| 7118 | 88358020 | Prexige 100mg tablets (Novartis Pharmaceuticals UK Ltd) | Lumiracoxib |
| 28383 | 88362020 | Prexige 400mg tablets (Novartis Pharmaceuticals UK Ltd) | Lumiracoxib |
| 70221 | 8228020 | Mefenamic acid 250mg capsules (Alliance Healthcare (Distribution) Ltd) | Mefenamic acid |
| 48810 | 8229020 | Dysman 250 capsules (Ashbourne Pharmaceuticals Ltd) | Mefenamic acid |
| 57297 | 8234020 | Mefenamic acid 500mg tablets (Alliance Healthcare (Distribution) Ltd) | Mefenamic acid |
| 51827 | 8236020 | Mefenamic acid 500mg tablets (Sigma Pharmaceuticals Plc) | Mefenamic acid |
| 66452 | 47561020 | Mefenamic acid 500mg tablets (Waymade Healthcare Plc) | Mefenamic acid |
| 34898 | 49994020 | Mefenamic acid 250mg Capsule (Berk Pharmaceuticals Ltd) | Mefenamic acid |
| 34910 | 49995020 | Mefenamic acid 500mg Tablet (Berk Pharmaceuticals Ltd) | Mefenamic acid |
| 4710 | 49999020 | Mefenamic acid 250mg Capsule (Actavis UK Ltd) | Mefenamic acid |
| 32090 | 50000020 | Mefenamic acid 500mg tablets (Actavis UK Ltd) | Mefenamic acid |
| 34438 | 50007020 | Mefenamic acid 250mg capsules (A A H Pharmaceuticals Ltd) | Mefenamic acid |
| 32105 | 50008020 | Mefenamic acid 500mg tablets (A A H Pharmaceuticals Ltd) | Mefenamic acid |
| 296 | 51182020 | Ponstan Forte 500mg tablets (Chemidex Pharma Ltd) | Mefenamic acid |
| 126 | 51186020 | Ponstan 250mg capsules (Chemidex Pharma Ltd) | Mefenamic acid |
| 1246 | 51187020 | Ponstan 250mg Dispersible tablet (Chemidex Pharma Ltd) | Mefenamic Acid |
| 14541 | 53445020 | Ponstan 50mg/5ml paediatric Liquid (Chemidex Pharma Ltd) | Mefenamic acid |
| 41677 | 54078020 | Mefenamic acid 250mg Capsule (IVAX Pharmaceuticals UK Ltd) | Mefenamic acid |
| 32234 | 54079020 | Mefenamic acid 500mg tablets (IVAX Pharmaceuticals UK Ltd) | Mefenamic acid |
| 34924 | 55454020 | Mefenamic acid 250mg Capsule (Teva UK Ltd) | Mefenamic acid |
| 41524 | 55455020 | Mefenamic acid 500mg tablets (Teva UK Ltd) | Mefenamic acid |
| 21831 | 56666020 | Dysman 250mg Capsule (Ashbourne Pharmaceuticals Ltd) | Mefenamic acid |
| 13459 | 56667020 | Dysman 500 tablets (Ashbourne Pharmaceuticals Ltd) | Mefenamic acid |
| 46967 | 59436020 | Mefenamic acid 250mg Capsule (Sandoz Ltd) | Mefenamic acid |
| 259 | 59793020 | Mefenamic acid 250mg capsules | Mefenamic acid |
| 34793 | 60065020 | Mefenamic acid 250mg capsules (Zentiva) | Mefenamic acid |
| 34595 | 60066020 | Mefenamic acid 500mg tablets (Zentiva) | Mefenamic acid |
| 46968 | 60540020 | Mefenamic acid 250mg capsules (Mylan) | Mefenamic acid |
| 1983 | 64363020 | Mefenamic acid 250mg Dispersible tablet | Mefenamic Acid |
| 1073 | 64364020 | Mefenamic acid 500mg tablets | Mefenamic acid |
| 9736 | 64365020 | Mefenamic acid 50mg/5ml oral suspension | Mefenamic acid |
| 57007 | 76223020 | Mefenamic acid 250mg capsules (Essential Generics Ltd) | Mefenamic acid |
| 61581 | 76226020 | Mefenamic acid 500mg tablets (Essential Generics Ltd) | Mefenamic acid |
| 22230 | 78062020 | Meflam 250mg Capsule (Trinity Pharmaceuticals Ltd) | Mefenamic acid |
| 26522 | 78063020 | Meflam 500mg Tablet (Trinity Pharmaceuticals Ltd) | Mefenamic acid |
| 33801 | 78352020 | Opustan 250mg Capsule (Opus Pharmaceuticals Ltd) | Mefenamic acid |
[truncated: 243,874 more chars]
