# Supplementary material for: Real-World Effectiveness of Beta-Blockers versus Other Antihypertensives in Reducing All-Cause Mortality and Cardiovascular Events
Source: Int J Clin Pract. 2022 Jul 30;2022:6124559. doi: 10.1155/2022/6124559 (PMC9356871; doi:10.1155/2022/6124559)
Supplement: Supplementary Materials — Supplementary Table 1. Antihypertensive drugs considered for each treatment of interest. Supplementary Tables 5–31. All code lists for exposure, covariates, and outcomes. Supplementary Figure 1. Patient attrition. Supplementary Table 2. Sensitivity analysis results for all-cause death and cardiovascular mortality with IPTW and Fine and Gray model for the event of cardiovascular mortality. Supplementary Table 3. Sensitivity analysis results for myocardial infarction with IPTW and fine and gray model. Supplementary Table 4. Sensitivity analysis results for cerebrovascular outcome with IPTW and fine and gray model. Supplementary Figure 2. Cumulative incidence curves for cerebrocardiovascular mortality with only death from cerebrocardiovascular causes as event. Supplementary Figure 3. Cumulative incidence curves for myocardial infarction. Supplementary Figure 4. Cumulative incidence curves for stroke, hemorrhagic stroke and ischemic stroke. [file 6124559.f1.zip › 6124559.f1/Supplementary file_Results (Sections 3.1; 3.2; 3.4.2).docx]

**Supplementary file: Results (Section 3.1)**

**Supplementary Figure 1. Patient attrition**


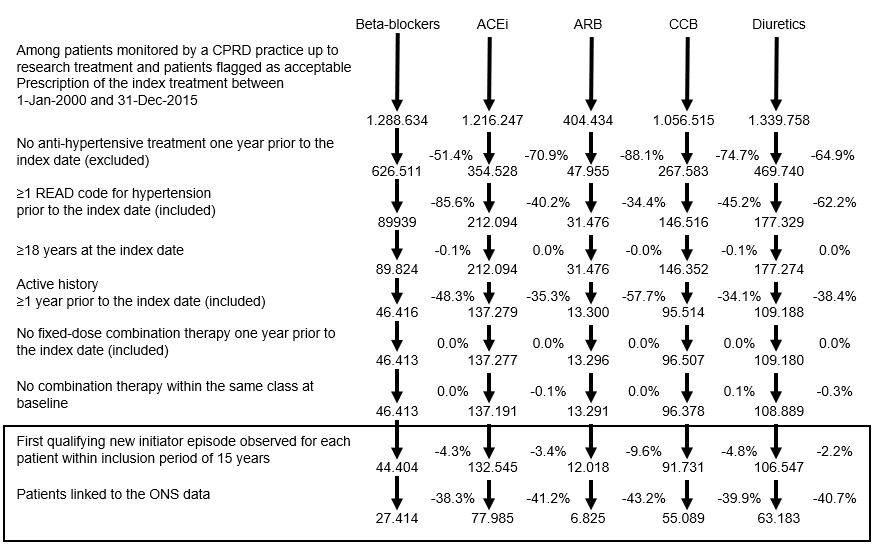


ACE, angiotensin-converting enzyme; ARB, angiotensin II receptor blockers; CCB, calcium channel blockers; CPRD, Clinical Practice Research Datalink; ONS, Office for National Statistics.

**Supplementary file: Results (Sections 3.2; 3.4.2)**

**Supplementary Tables 2–4: Results**

**Supplementary Table 2. Sensitivity analysis results for all-cause death and cardiovascular mortality with IPTW and Fine and Gray model for the event of cardiovascular mortality**

ACEi, angiotensin-converting enzyme inhibitor; ARB, angiotensin II receptor blockers; CCB, calcium channel blockers; CI, confidence interval; HR, hazard ratio; IPTW, inverse probability of treatment weighting; SHR, sub-distribution hazard ratio.

|  | **Beta-blockers** | | **ACEi** | **ARB** | | | **CCB** | | **Diuretics** | |
| --- | --- | --- | --- | --- | --- | --- | --- | --- | --- | --- |
|  | **n = 44,404** | | **n = 132,545** | **n = 12,018** | | | **n = 91,731** | | **n = 106,547** | |
| **All-cause mortality** | | |  |  | | |  | |  | |
| **HR (CI 98.75%)** |  | |  |  | | |  | |  | |
| Adjusted | 1 | | 0.71 (0.61,0.83) | 0.67 (0.51,0.88) | | | 0.76 (0.66,0.88) | | 1.06 (0.93,1.22) | |
| After IPTW | 1 | | 0.73 (0.63, 0.84) | 0.63 (0.49, 0.80) | | | 0.93 (0.82, 1.06) | | 1.32 (1.18, 1.49) | |
|  | | **Beta-blockers** | **ACEi** | | **ARB** | **CCB** | | **Diuretics** | |  |
|  | | **n = 27,414** | **n = 77,985** | | **n = 6,825** | **n = 55,089** | | **n = 63,183** | |  |
| **Cardiovascular mortality** | | |  | |  |  | |  | |  |
| **HR (95.75%)** | |  |  | |  |  | |  | |  |
| Adjusted | | 1 | 0.63 (0.43,0.91) | | 0.64 (0.32,1.28) | 0.71 (0.49,1.03) | | 0.97 (0.69,1.37) | |  |
| After IPTW | | 1 | 0.80 (0.56, 1.14) | | 0.64 (0.35, 1.16) | 0.96 (0.71, 1.30) | | 1.20 (0.90, 1.61) | |  |
| **SHR (98.75%)** | |  |  | |  |  | |  | |  |
| Adjusted | | 1 | 0.82 (0.57,1.18) | | 0.89 (0.45,1.77) | 0.81 (0.56,1.16) | | 0.93 (0.66,1.30) | |  |
| After IPTW | | 1 | 0.95 (0.67, 1.36) | | 0.87 (0.48, 1.58) | 1.01 (0.74, 1.37) | | 1.10 (0.82, 1.47) | |  |

**Supplementary Table 3. Sensitivity analysis results for myocardial infarction with IPTW and Fine and Gray model**

ACEi, angiotensin-converting enzyme inhibitor; ARB, angiotensin II receptor blockers; CCB, calcium channel blockers; CI, confidence interval; IPTW, inverse probability of treatment weighting; SHR, sub-distribution hazard ratio.

|  | **Beta-blockers** | **ACEi** | **ARB** | **CCB** | **Diuretics** |
| --- | --- | --- | --- | --- | --- |
| **Cardiovascular outcome HR  (CI 98.5%)** | **n = 44,404** | **n = 132,545** | **n = 12,018** | **n = 91,731** | **n = 106,547** |
| **Myocardial infarction** | |  |  |  |  |
| **HR (98.75%)** |  |  |  |  |  |
| Adjusted | 1 | 0.89 (0.69, 1.15) | 0.90 (0.59, 1.37) | 0.76 (0.58, 1.00) | 0.72 (0.54, 0.95) |
| After IPTW | 1 | 0.90 (0.71, 1.14) | 0.86 (0.60, 1.24) | 0.80 (0.63, 1.01) | 0.76 (0.60, 0.96) |
| **SHR (98.75%)** |  |  |  |  |  |
| Adjusted | 1 | 1.09 (0.85, 1.41) | 1.27 (0.83, 1.94) | 0.86 (0.66, 1.12) | 0.66 (0.50, 0.87) |
| After IPTW | 1 | 1.06 (0.83, 1.33) | 1.16 (0.80, 1.67) | 0.86 (0.68, 1.09) | 0.70 (0.55, 0.88) |

**Supplementary Table 4. Sensitivity analysis results for cerebrovascular outcome with IPTW and Fine and Gray model**

|  | **Beta-blockers** | **ACEi** | **ARB** | **CCB** | **Diuretics** |
| --- | --- | --- | --- | --- | --- |
| **Cerebrovascular outcomes**  **(CI 98.5%)** | **n = 44,404** | **n = 132,545** | **n = 12,018** | **n = 91,731** | **n = 106,547** |
| **Stroke** |  |  |  |  |  |
| **HR (98.75%)** |  |  |  |  |  |
| Adjusted | 1 | 0.86 (0.69,1.09) | 0.73 (0.49,1.10) | 0.83 (0.66,1.05) | 0.66 (0.52,0.84) |
| After IPTW | 1 | 0.95 (0.76, 1.18) | 0.67 (0.46, 0.96) | 0.93 (0.76, 1.13) | 0.72 (0.59, 0.87) |
| **SHR (98.75%)** |  |  |  |  |  |
| Adjusted | 1 | 1.08 (0.86,1.36) | 1.03 (0.68,1.54) | 0.97 (0.77,1.22) | 0.65 (0.51,0.83) |
| After IPTW | 1 | 1.11 (0.89, 1.37) | 0.90 (0.62, 1.29) | 0.99 (0.82, 1.21) | 0.66 (0.54, 0.80) |
| **Hemorrhagic stroke** | |  |  |  |  |
| **HR (98.75%)** |  |  |  |  |  |
| Adjusted | 1 | 0.73 (0.36, 1.45) | 1.54 (0.59, 4.04) | 0.91 (0.45, 1.82) | 0.46 (0.21, 1.00) |
| After IPTW | 1 | 0.88 (0.49, 1.57) | 1.55 (0.66, 3.60) | 1.31 (0.72, 2.38) | 0.56 (0.30, 1.05) |
| **SHR (98.75%)** |  |  |  |  |  |
| Adjusted | 1 | 0.94 (0.47, 1.87) | 2.24 (0.86, 5.82) | 1.06 (0.53, 2.11) | 0.46 (0.21, 1.00) |
| After IPTW | 1 | 1.07 (0.60, 1.92) | 2.14 (0.93, 4.91) | 1.40 (0.78, 2.52) | 0.52 (0.28, 0.97) |
| **Ischemic stroke** |  |  |  |  |  |
| **HR (98.75%)** |  |  |  |  |  |
| Adjusted | 1 | 0.78 (0.51, 1.20) | 0.35 (0.14, 0.92) | 0.76 (0.50, 1.17) | 0.59 (0.37, 0.92) |
| After IPTW | 1 | 0.88 (0.59, 1.29) | 0.22 (0.10, 0.48) | 0.80 (0.55, 1.15) | 0.65 (0.45, 0.94) |
| **SHR (98.75%)** |  |  |  |  |  |
| Adjusted | 1 | 0.98 (0.64, 1.49) | 0.50 (0.19, 1.31) | 0.88 (0.57, 1.34) | 0.58 (0.37, 0.90) |
| After IPTW | 1 | 1.02 (0.70, 1.51) | 0.28 (0.13, 0.62) | 0.86 (0.59, 1.23) | 0.60 (0.42, 0.87) |

ACEi, angiotensin-converting enzyme inhibitor; ARB, angiotensin II receptor blockers; BP, blood pressure; CCB, calcium channel blockers, HR, hazard ratio; IPTW, inverse probability of treatment weighting; SHR, sub-distribution hazard ratio.

**Supplementary Figures 2–4 : Results**

**Supplementary Figure 2. Cumulative incidence curves for cerebrocardiovascular mortality with only death from cerebrocardiovascular causes as event**


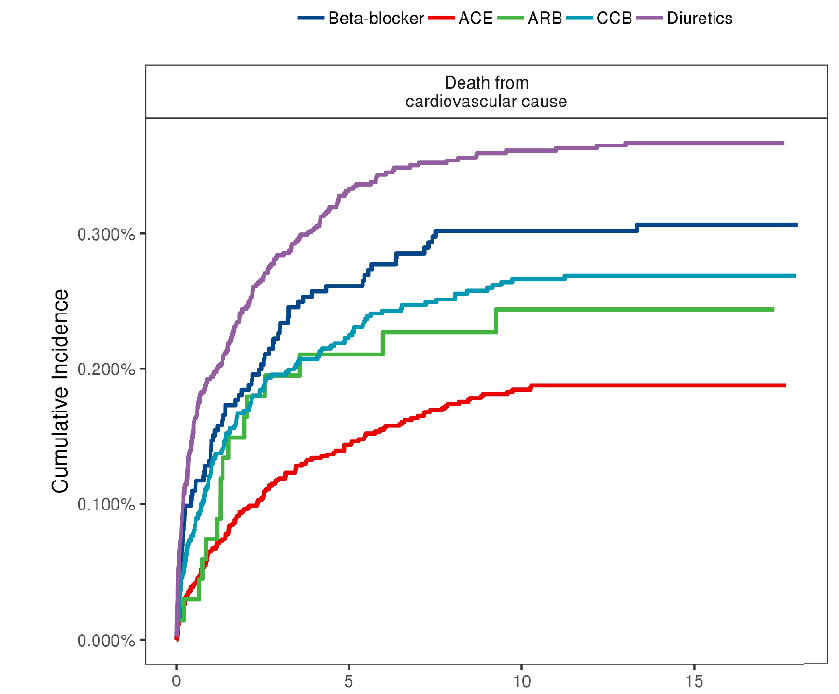


ACE, angiotensin-converting enzyme; ARB, angiotensin II receptor blockers; CCB, calcium channel blockers.

**Supplementary Figure 3. Cumulative incidence curves for myocardial infarction (beta-blocker in blue, ACE in red, ARB in green, CCB in light blue and diuretics in purple)**


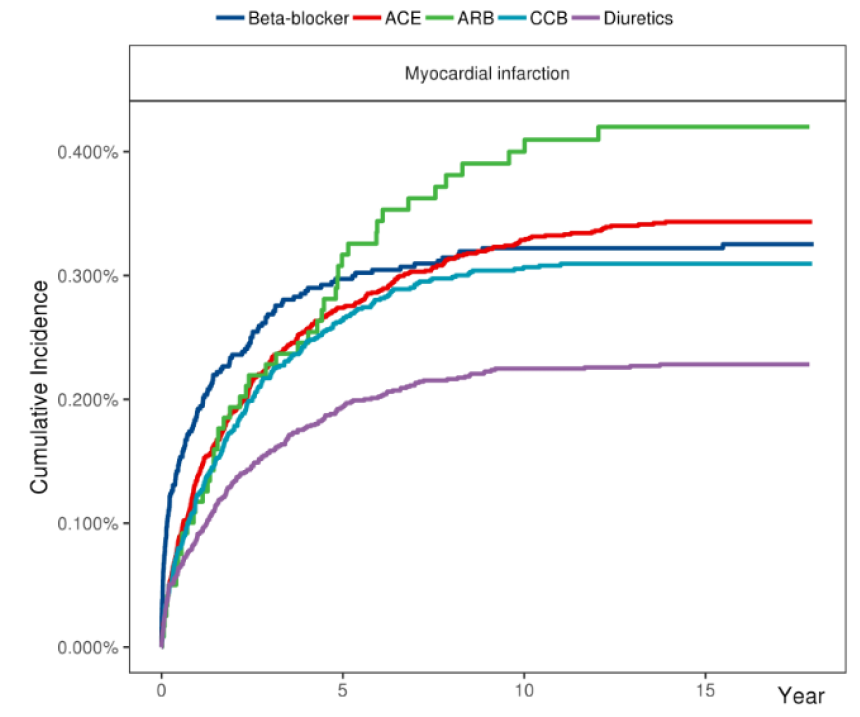


ACE, angiotensin-converting enzyme; ARB, angiotensin II receptor blockers; CCB, calcium channel blockers.

**Supplementary Figure 4. Cumulative incidence curves for stroke, hemorrhagic stroke and ischemic stroke (beta-blocker in blue, ACE in red, ARB in green, CCB in light blue and diuretics in purple)**


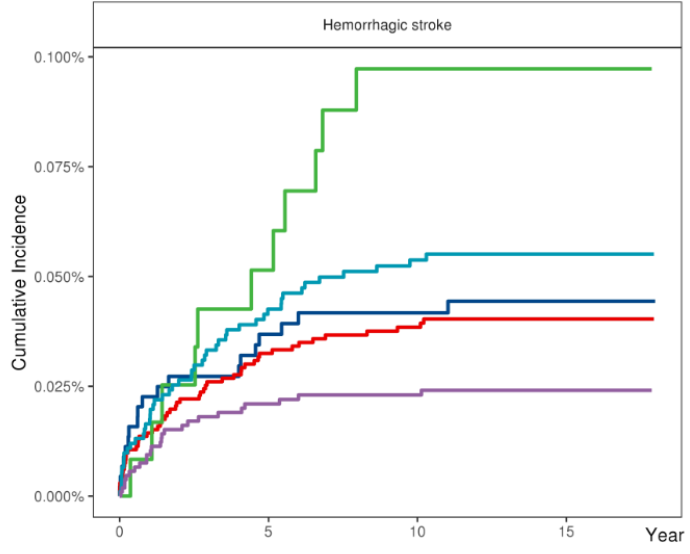


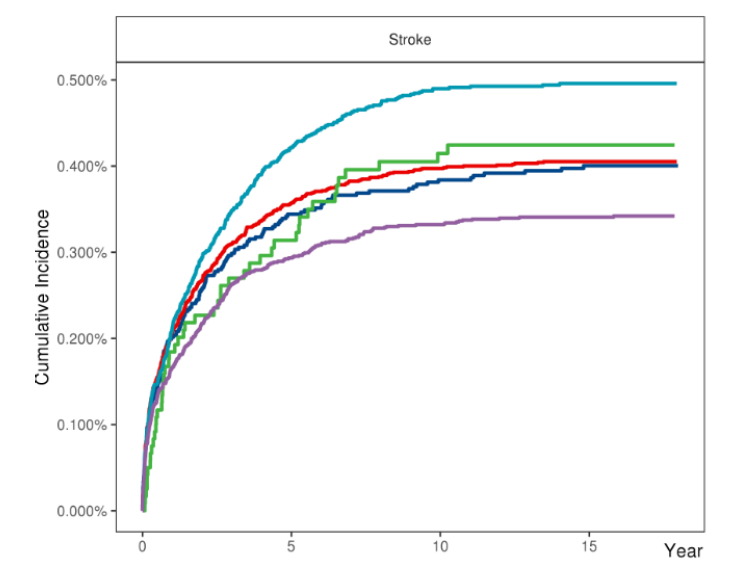


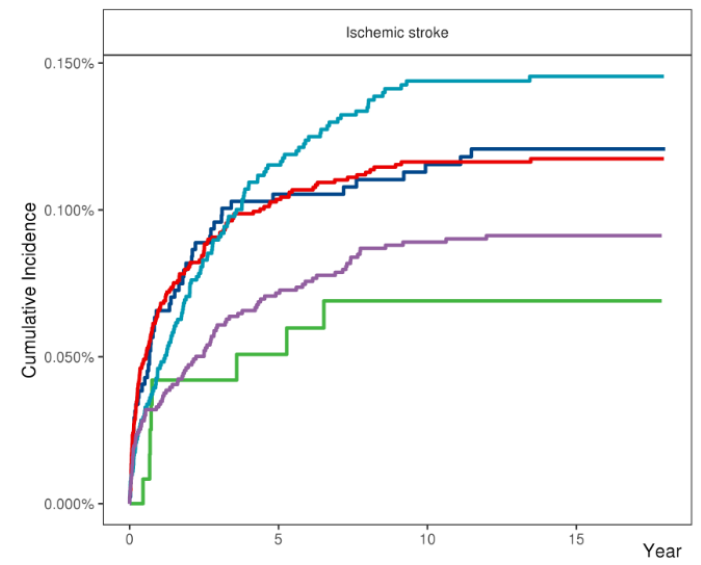


ACE, angiotensin-converting enzyme; ARB, angiotensin II receptor blockers; CCB, calcium channel blockers.
